# Supplementary figures and images for: Synergistic effects of notoginsenoside R1 and saikosaponin B2 in atherosclerosis: A novel approach targeting PI3K/AKT/mTOR pathway and macrophage autophagy
Source: PLoS One. 2025 Jun 27;20(6):e0326687. doi: 10.1371/journal.pone.0326687 (PMC12204583; doi:10.1371/journal.pone.0326687)

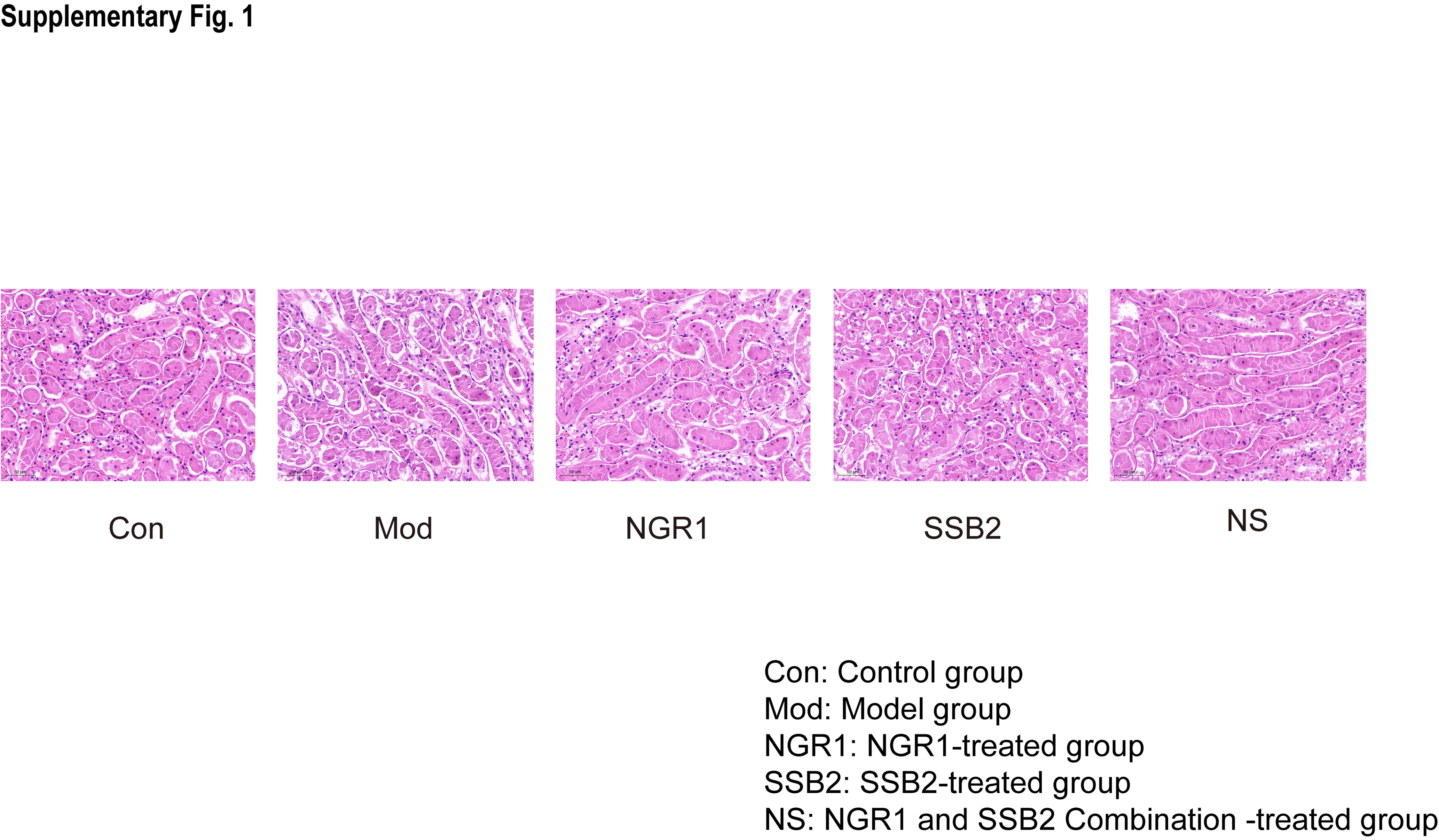

Supplement: S1 Fig — The HE-stained section of mouse kidney, scale bar = 50 μm. (TIF) [file pone.0326687.s001.tif]
