# Supplementary material for: Synergistic effects of notoginsenoside R1 and saikosaponin B2 in atherosclerosis: A novel approach targeting PI3K/AKT/mTOR pathway and macrophage autophagy
Source: PLoS One. 2025 Jun 27;20(6):e0326687. doi: 10.1371/journal.pone.0326687 (PMC12204583; doi:10.1371/journal.pone.0326687)
Supplement: S1 File — (PDF) [file pone.0326687.s002.pdf]

All experiments were performed in triplicate (n=3)
Representative raw images from each independent experiment are shown.

NLRP3-1-full

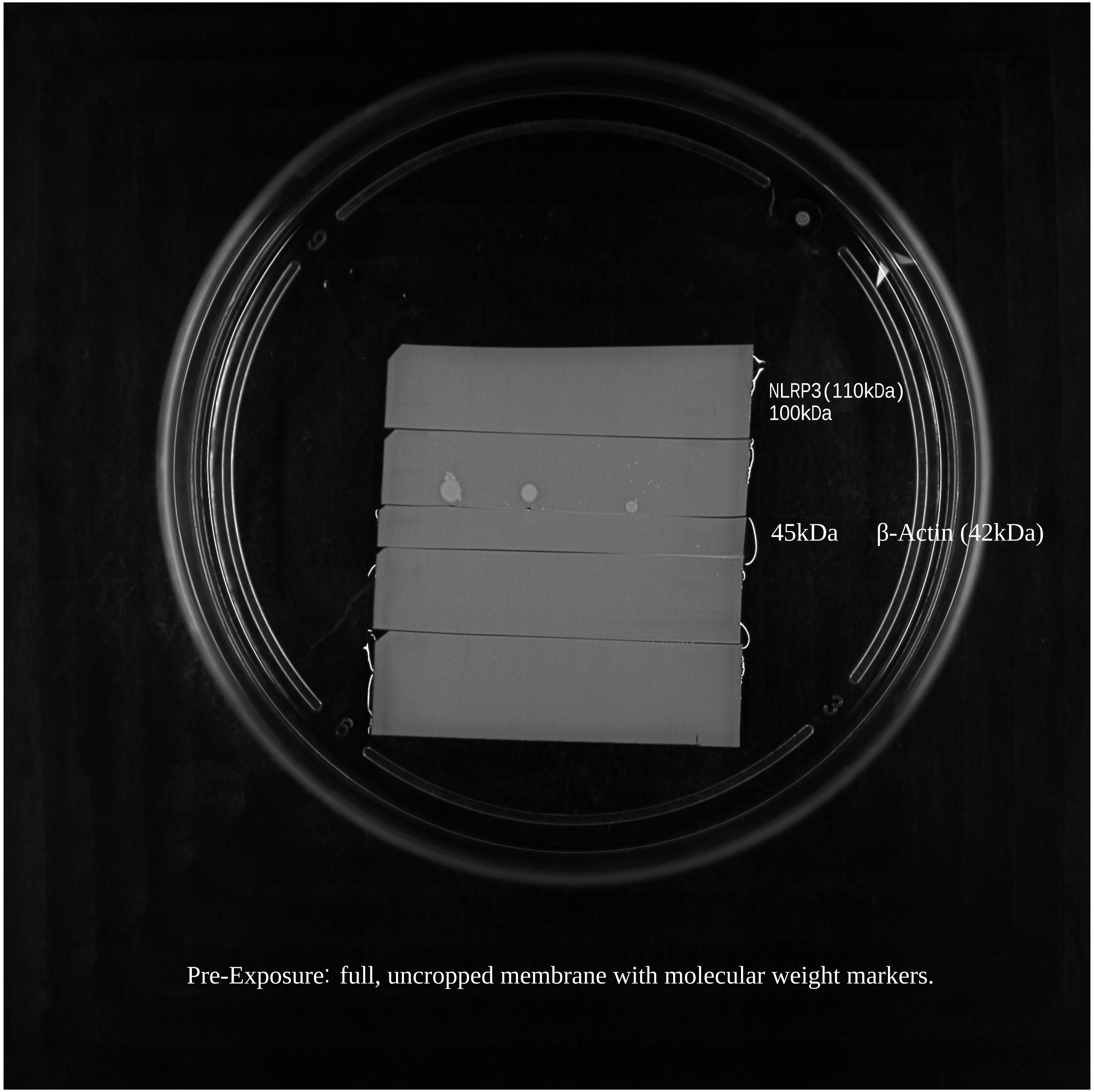

Con Mod NGR1 SSB2 NS

NLRP3-1

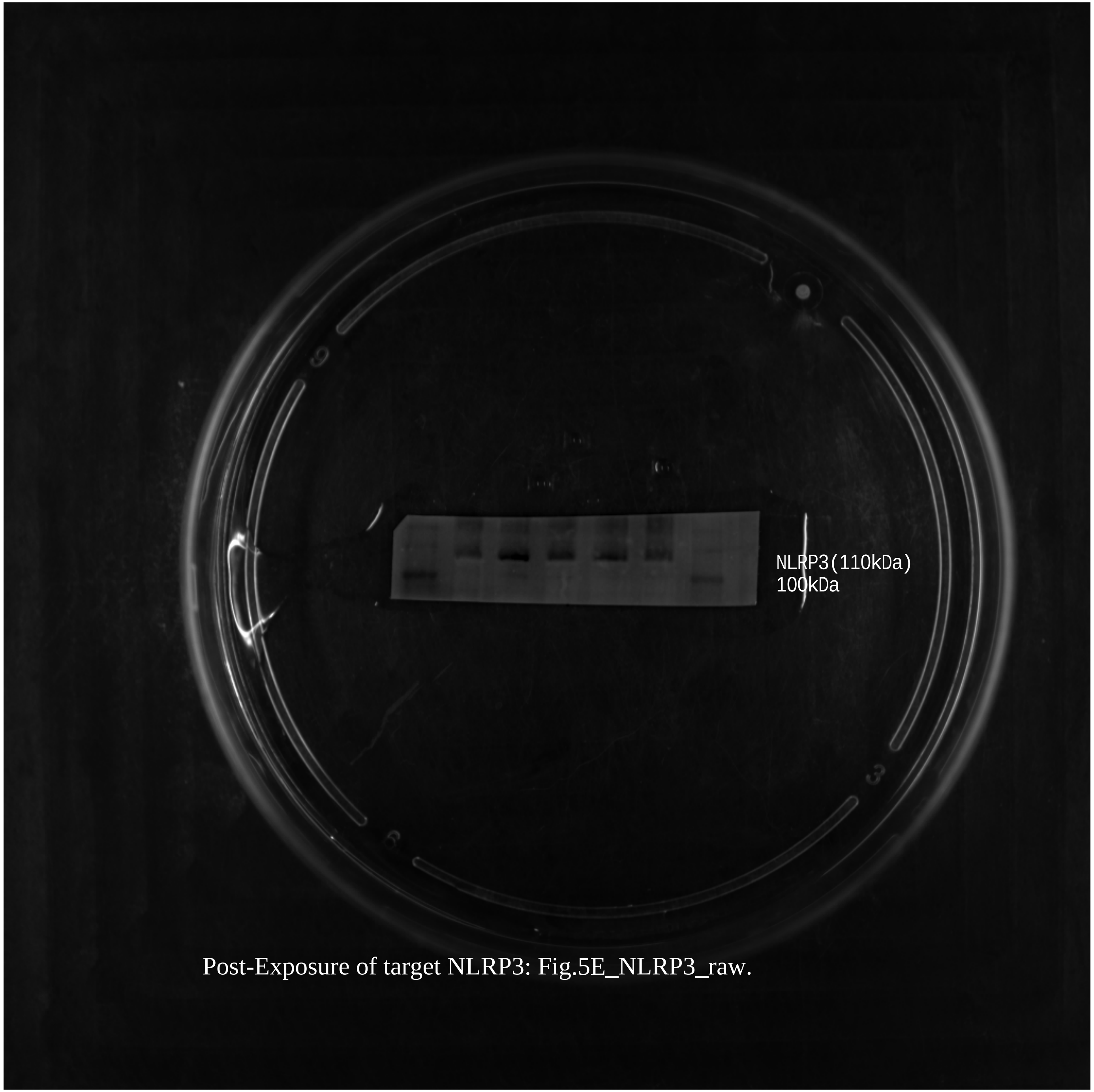

Con Mod NGR1 SSB2 NS

NLRP3-1-β-Actin

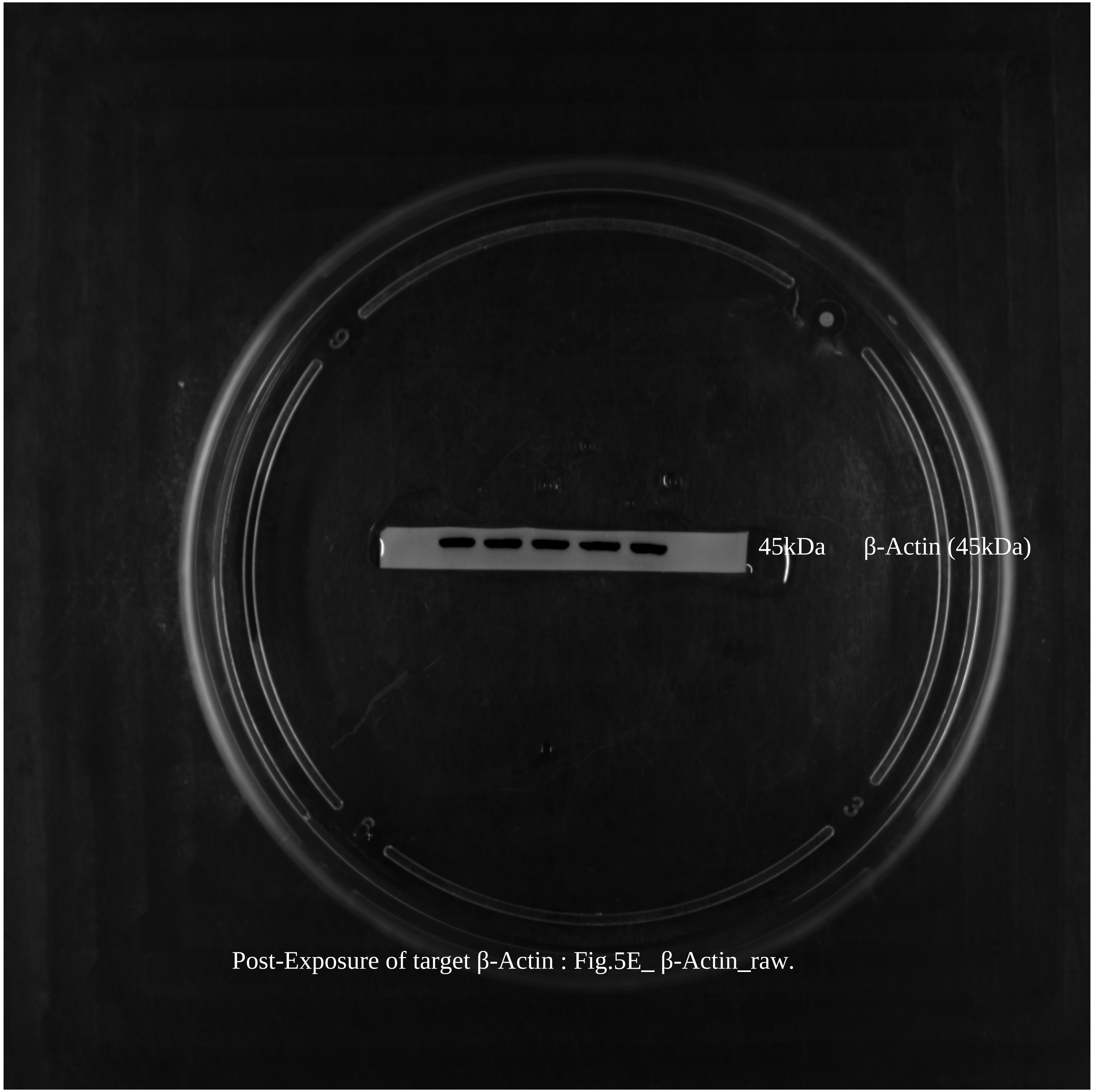

Con Mod NGR1 SSB2 NS

IL1β-1&2-full

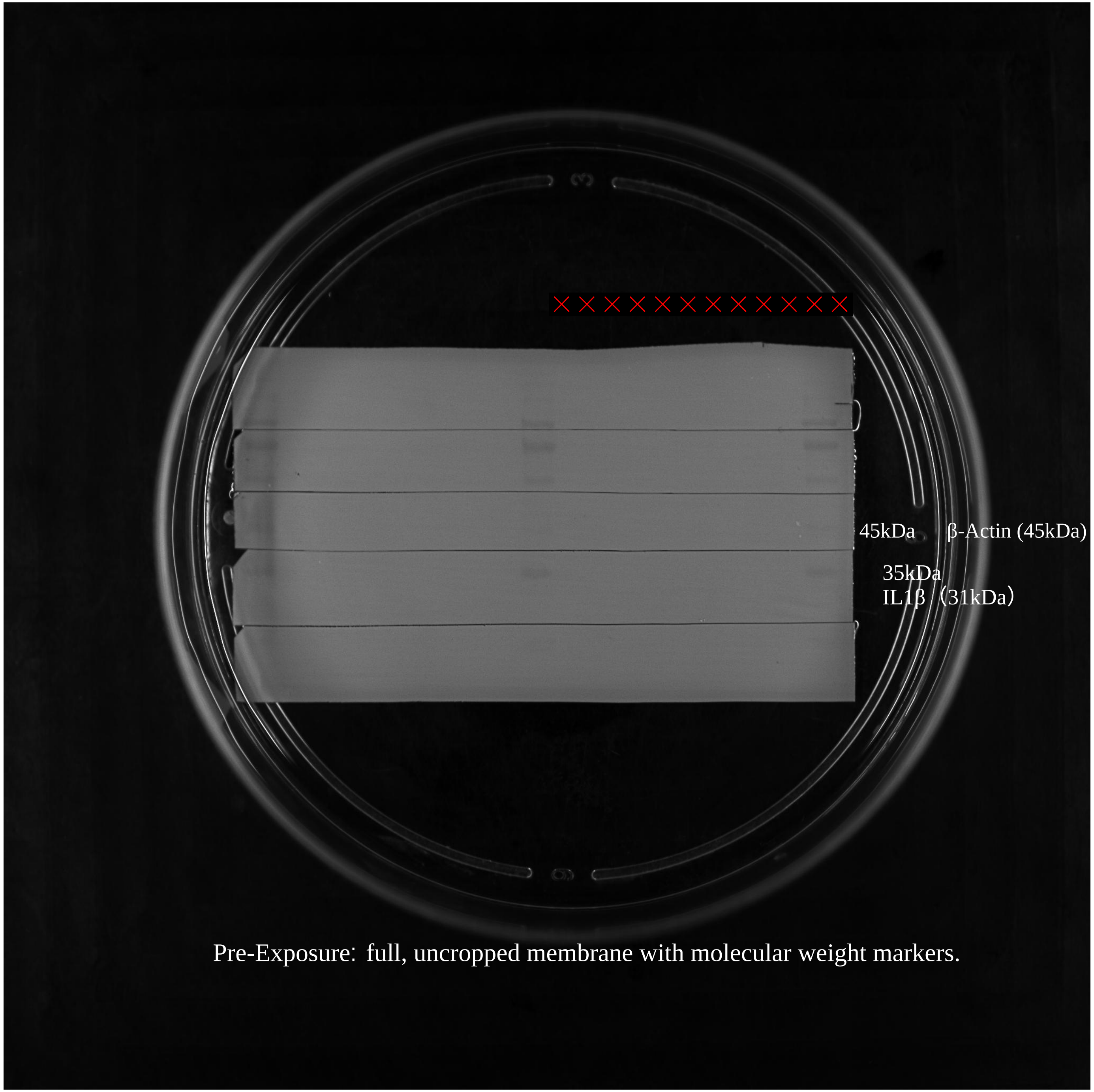

Con Mod NGR1 SSB2 NS   Con Mod NGR1 SSB2 NS

IL1β-1&2

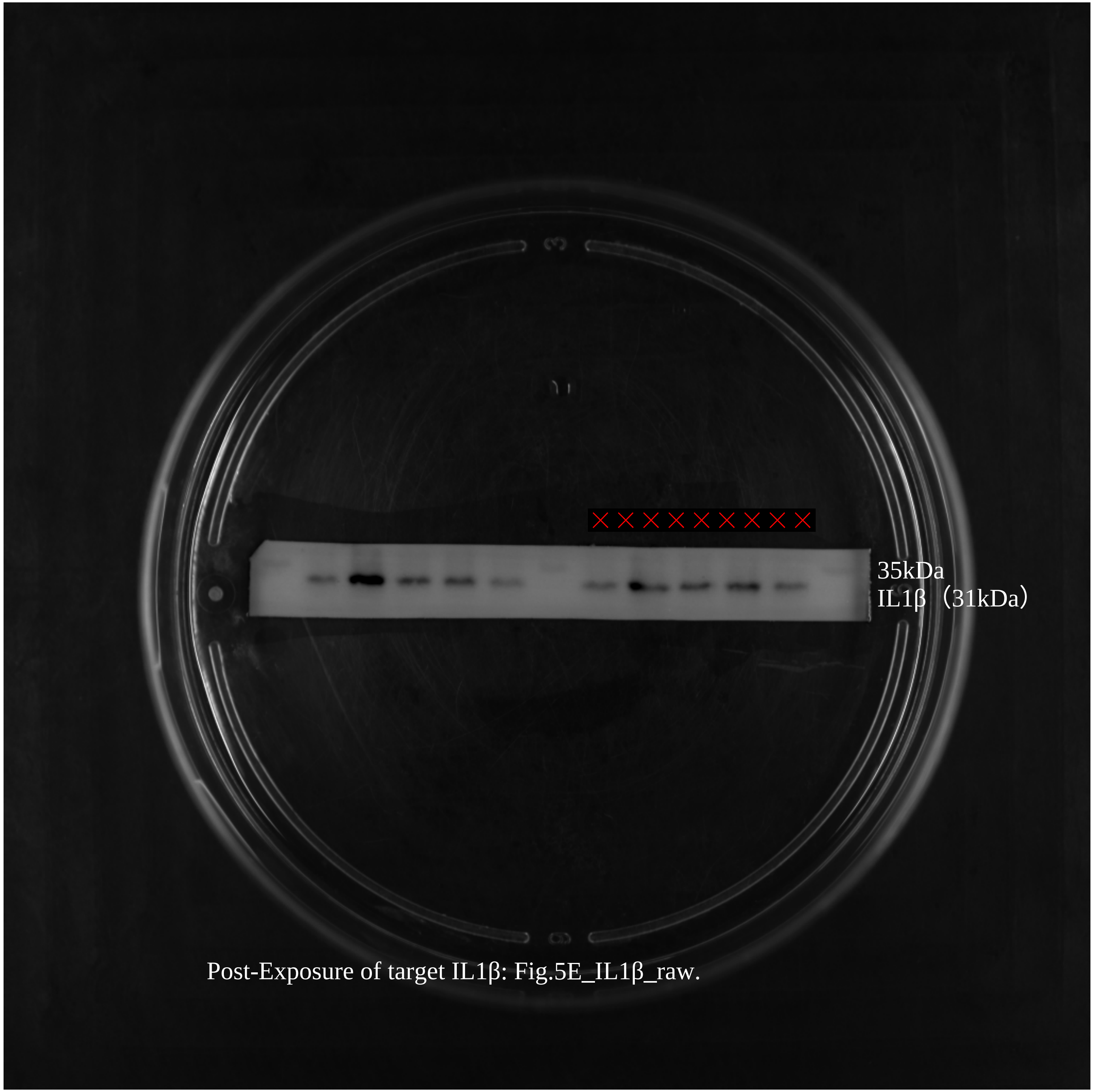

Con Mod NGR1 SSB2 NS   Con Mod NGR1 SSB2 NS

IL1β-1&2-β-Actin

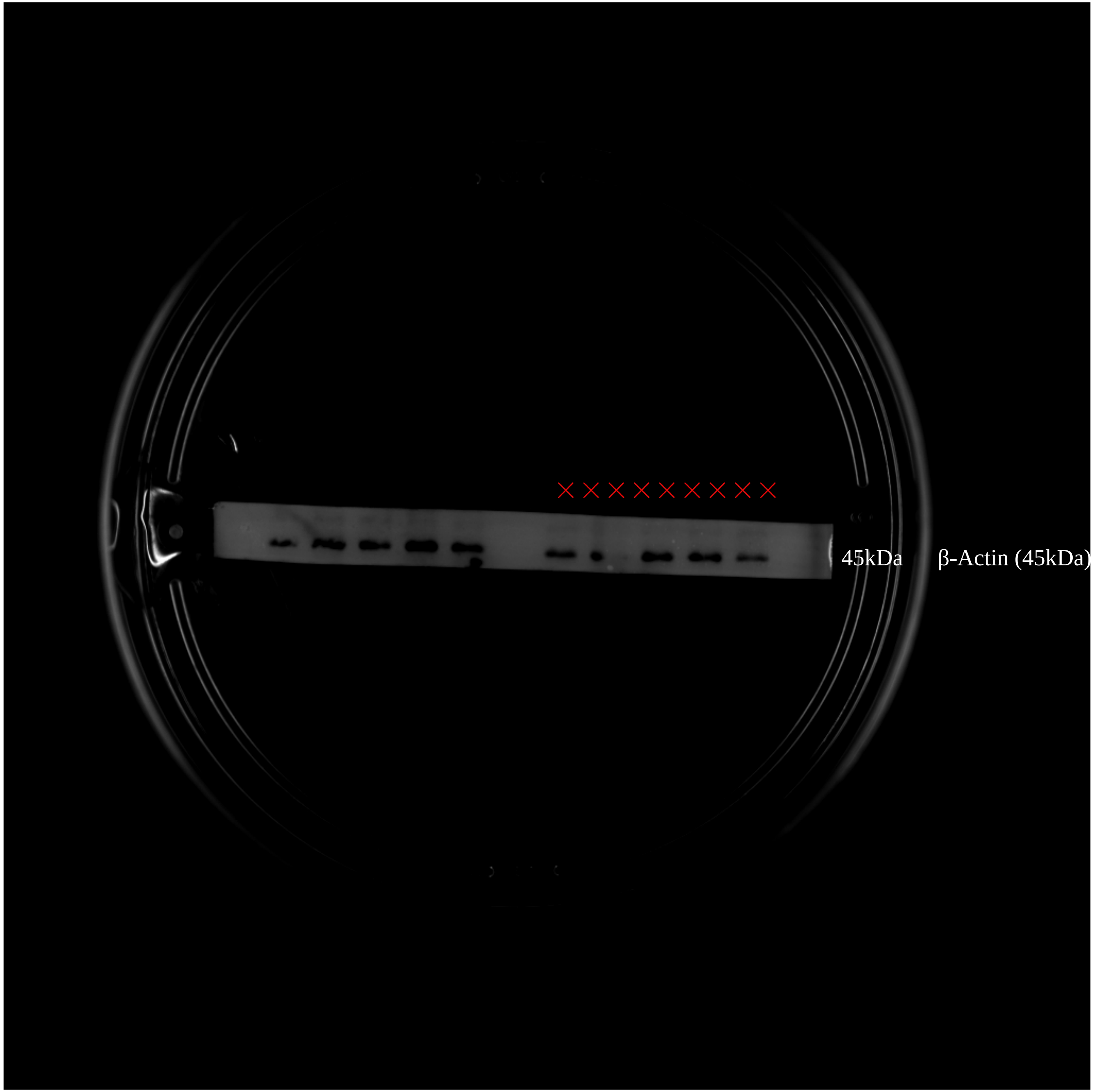

Con Mod NGR1 SSB2 NS Con Mod NGR1 SSB2 NS

IL18-1&2-full

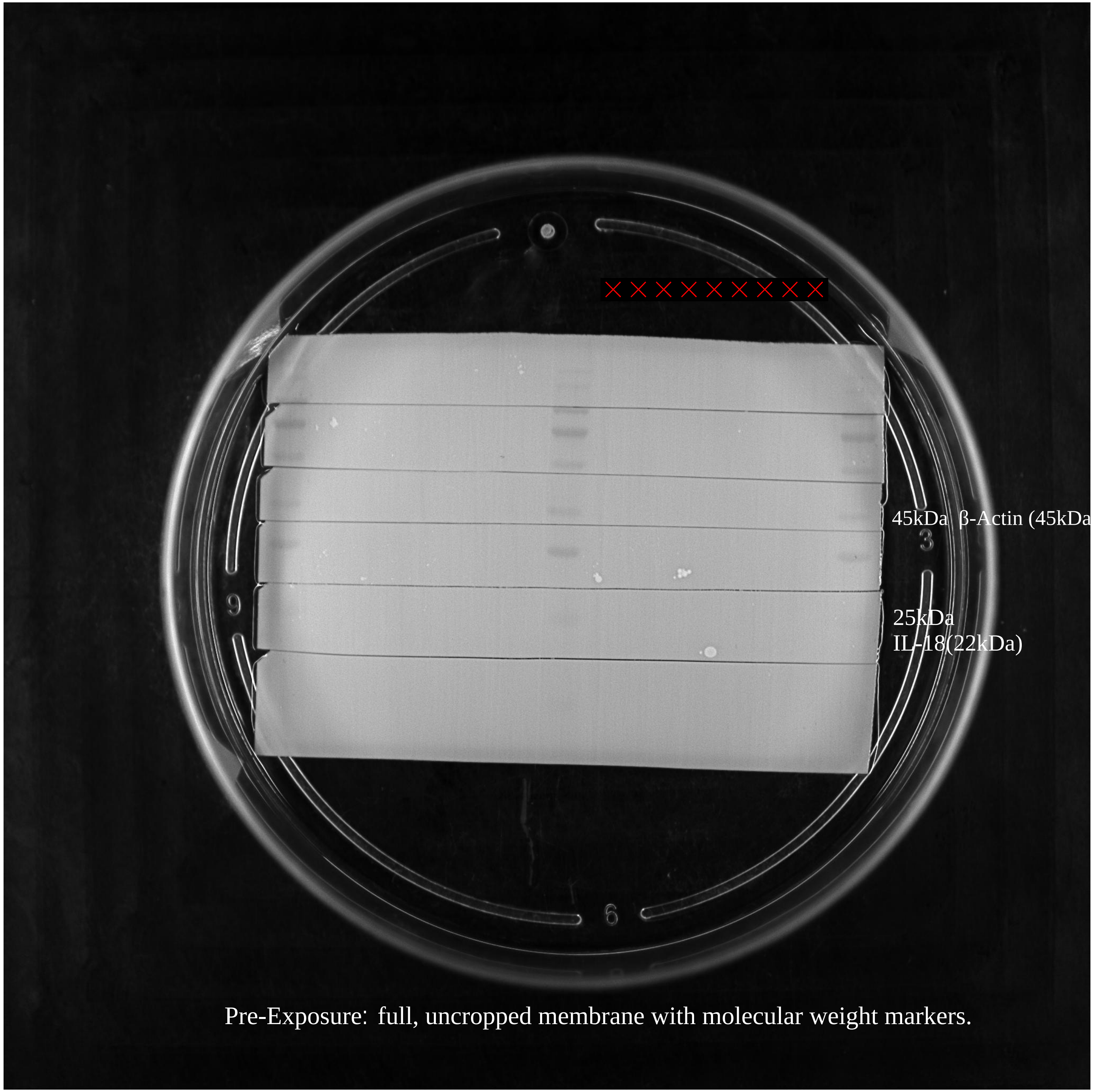

Con Mod NGR1 SSB2 NS Con Mod NGR1 SSB2 NS

IL18-1&2

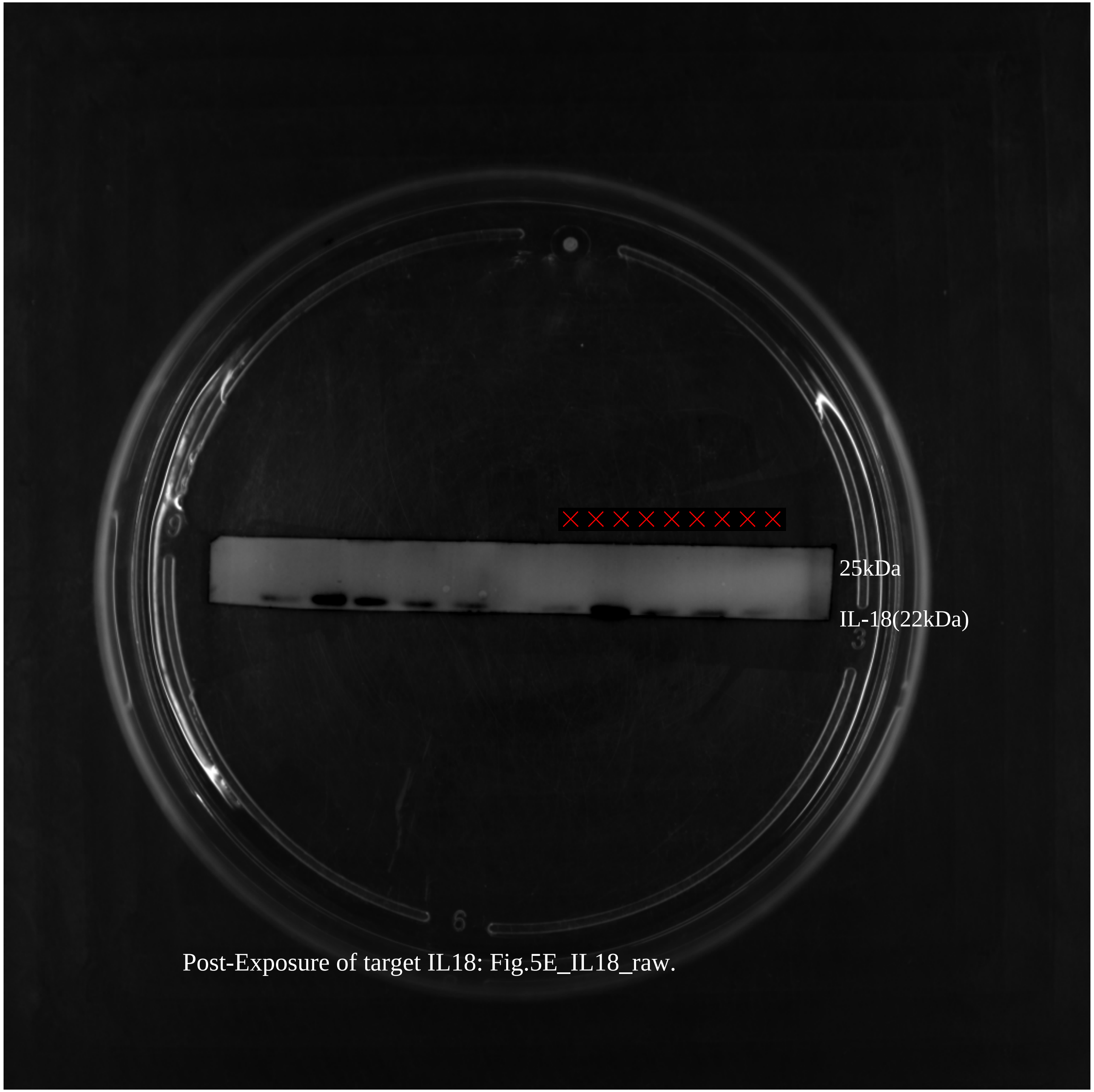

Con Mod NGR1 SSB2 NS Con Mod NGR1 SSB2 NS

IL18-1&2-β-Actin

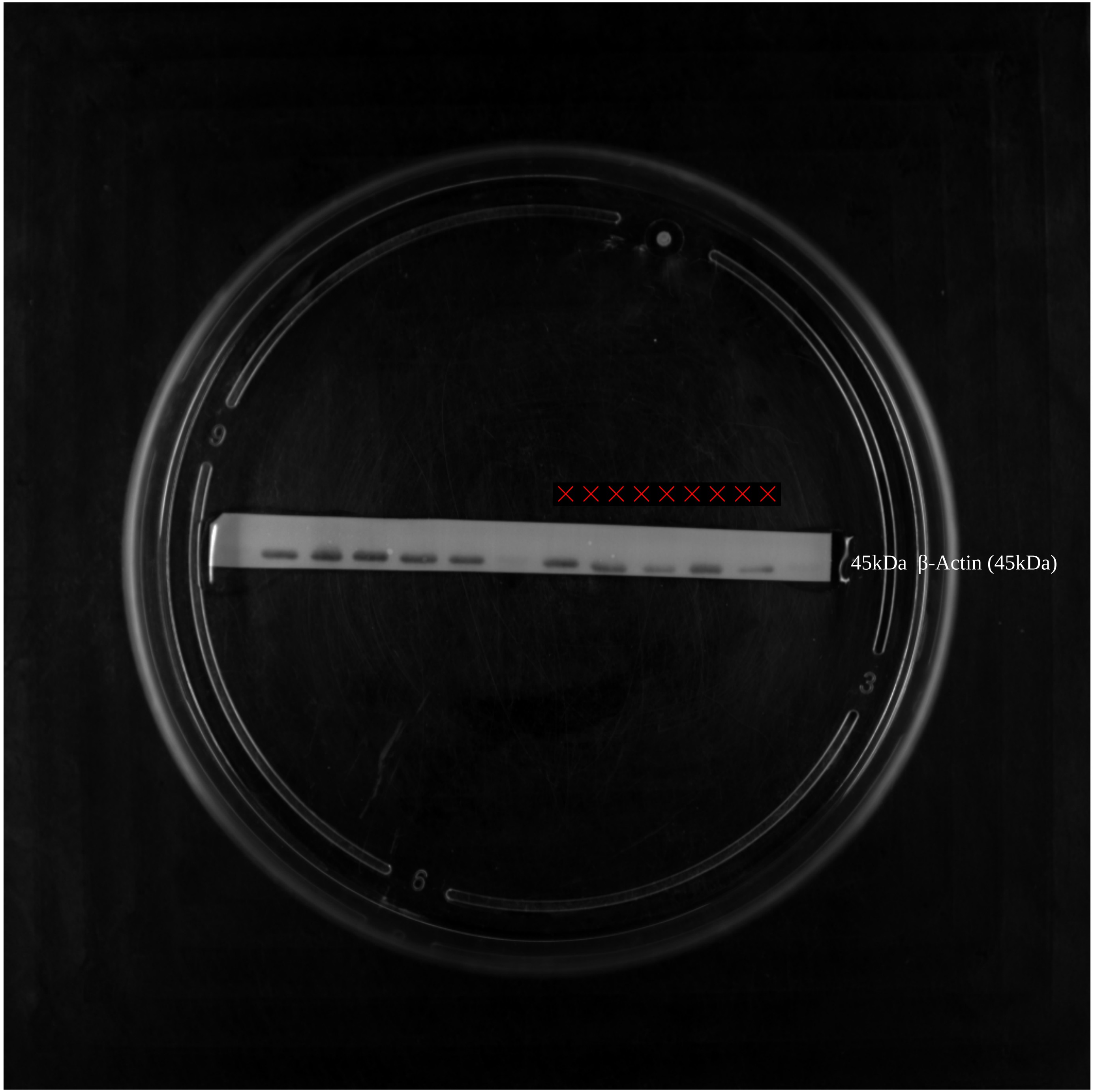

Con Mod NGR1 SSB2 NS Con Mod NGR1 SSB2 NS

BAX-1&2-full

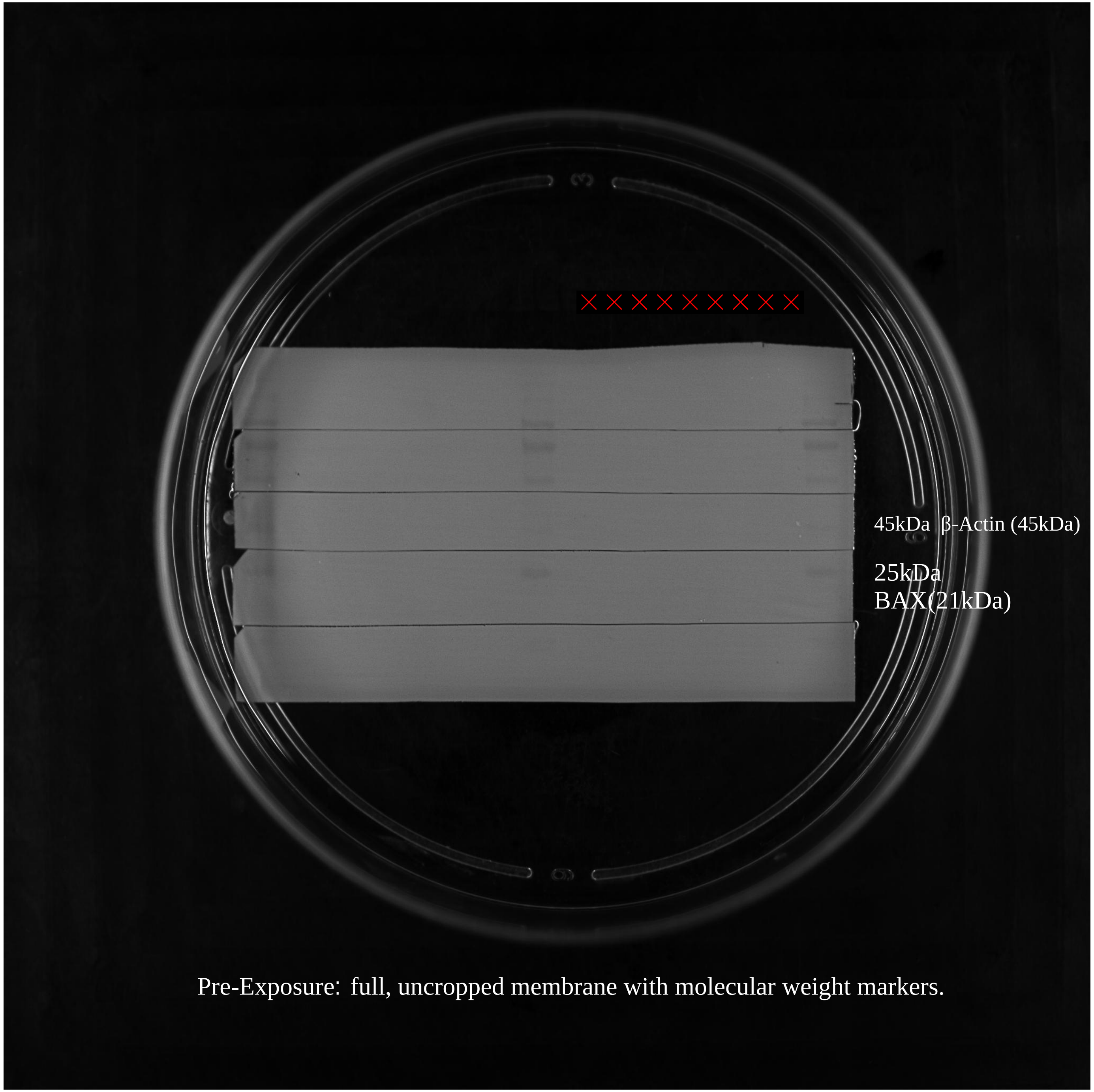

Con Mod NGR1 SSB2 NS Con Mod NGR1 SSB2 NS

BAX-1&2

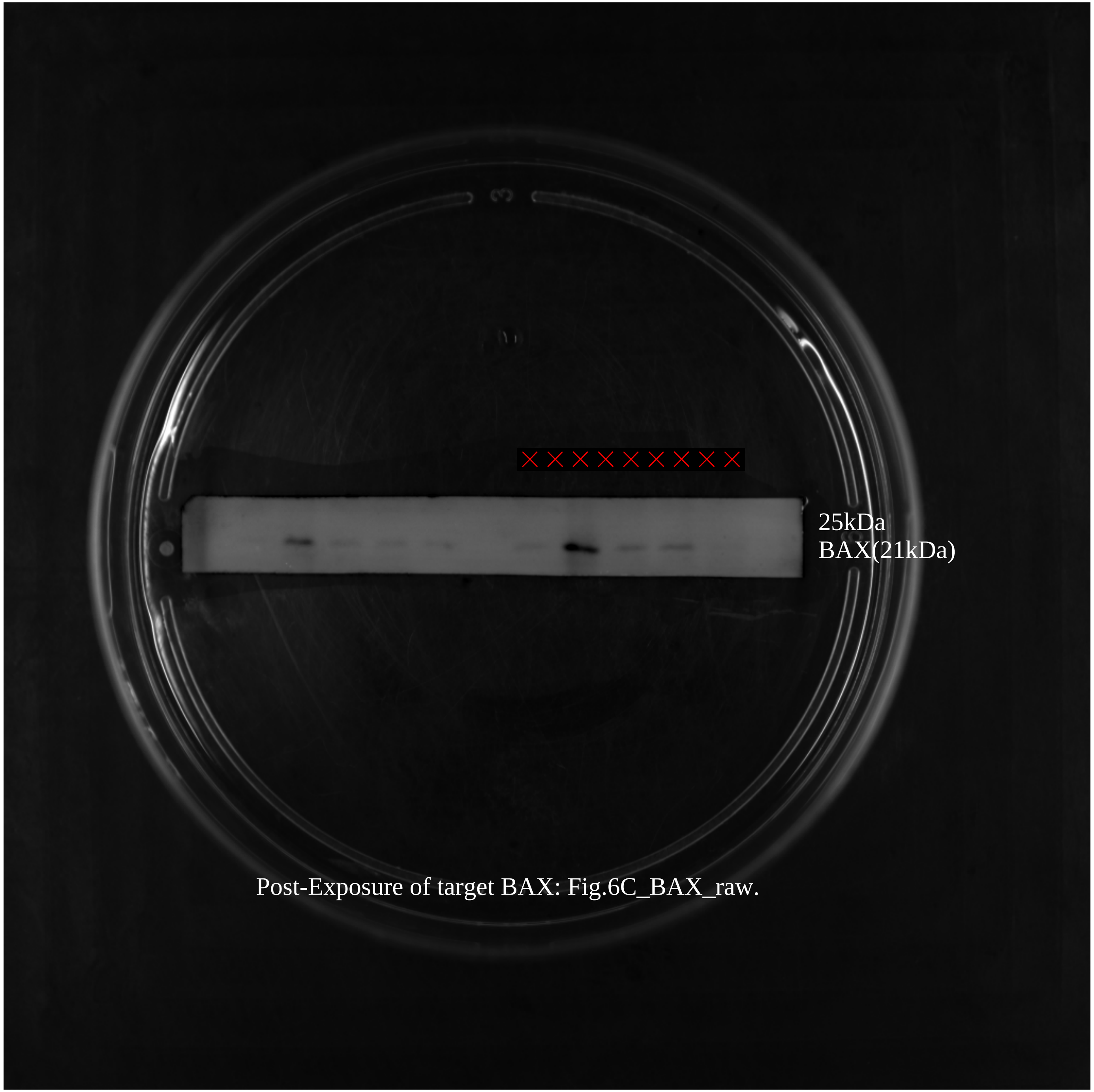

Con Mod NGR1 SSB2 NS Con Mod NGR1 SSB2 NS

BAX-1&2-β-Actin

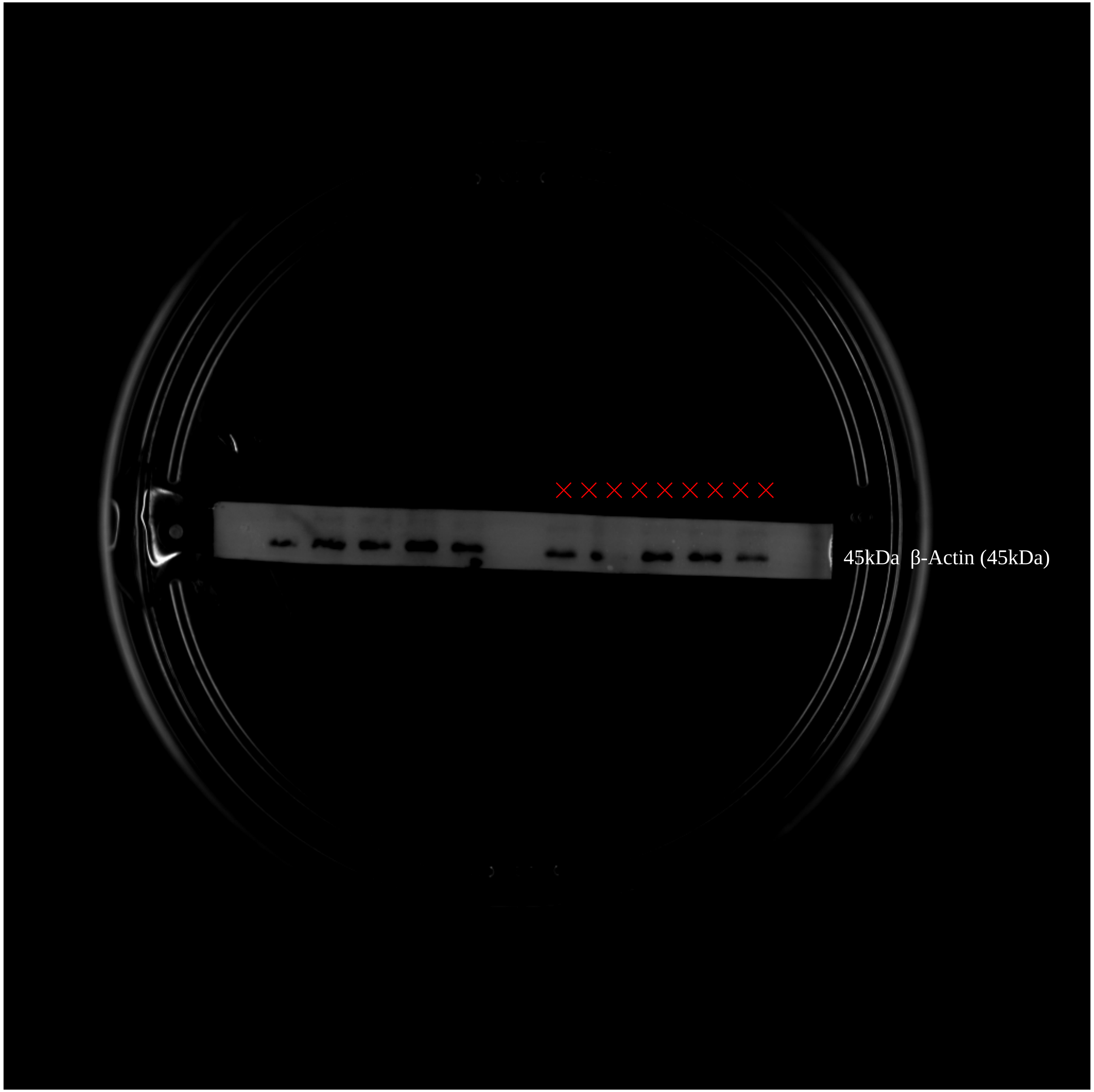

Con Mod NGR1 SSB2 NS Con Mod NGR1 SSB2 NS

BCL2-1-full

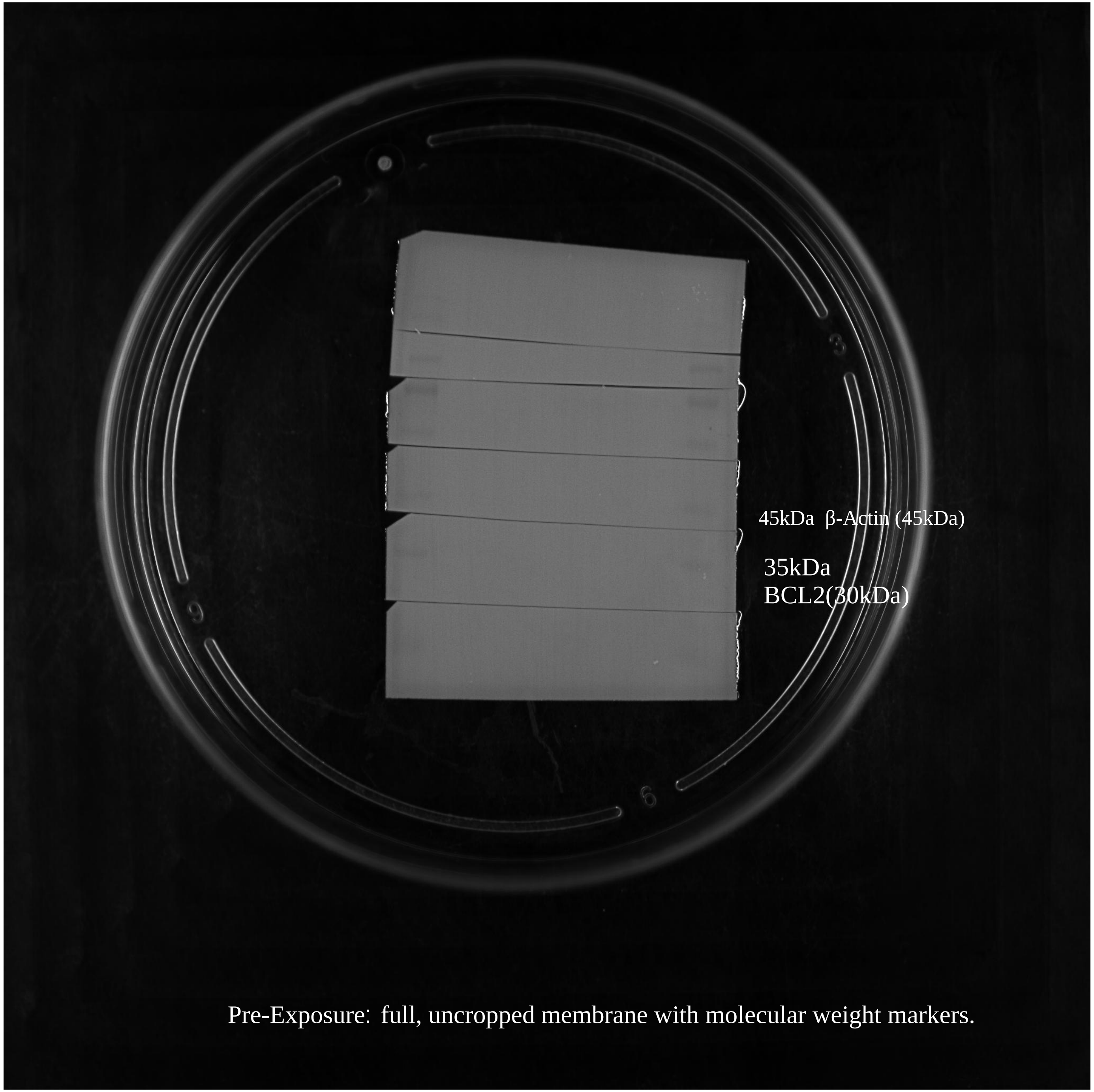

Con Mod NGR1 SSB2 NS

BCL2-1

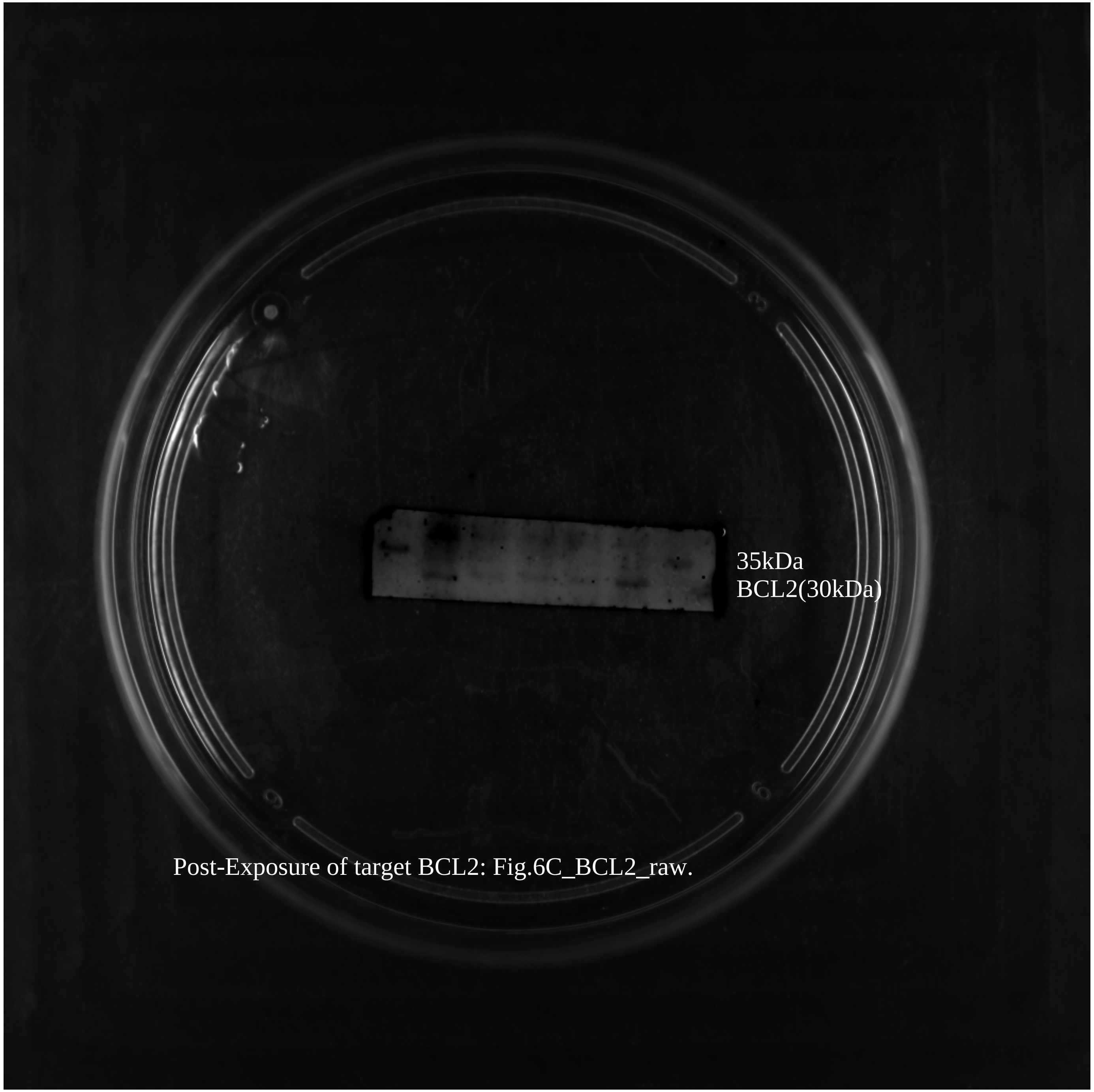

Con Mod NGR1 SSB2 NS

BCL2-1-β-Actin

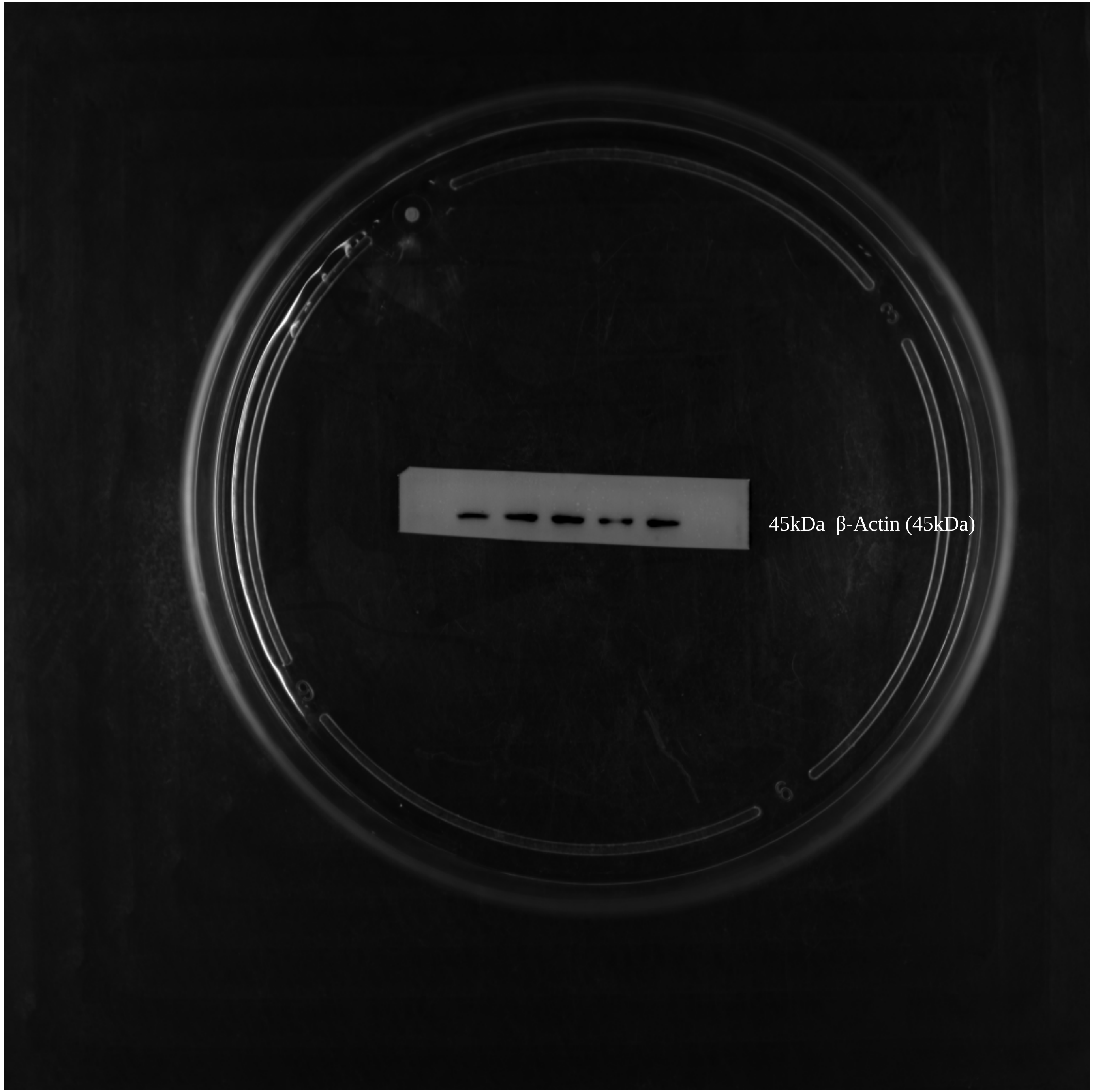

Con Mod NGR1 SSB2 NS

Caspase-3-1-full

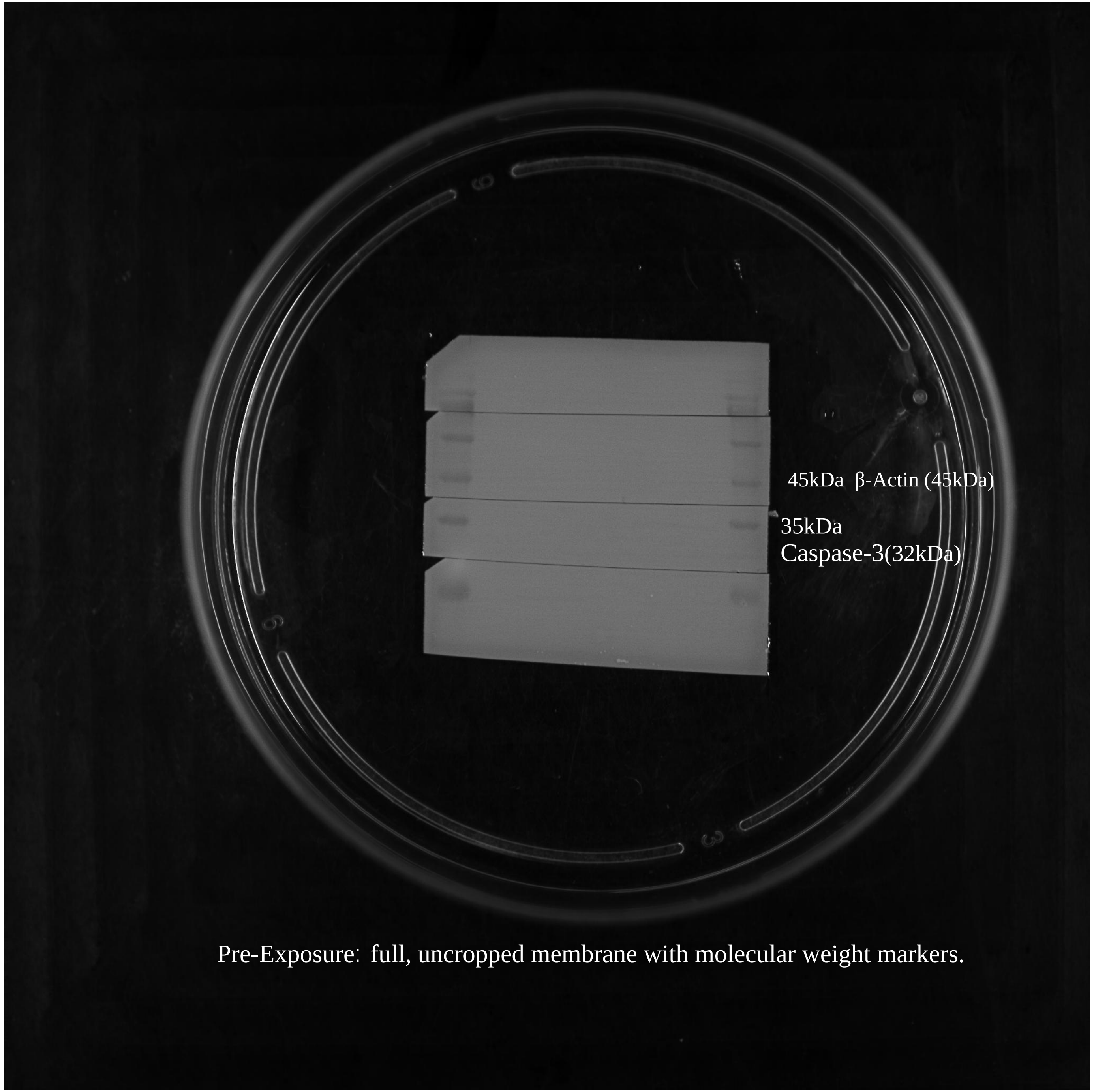

Con Mod NGR1 SSB2 NS

Caspase-3-1

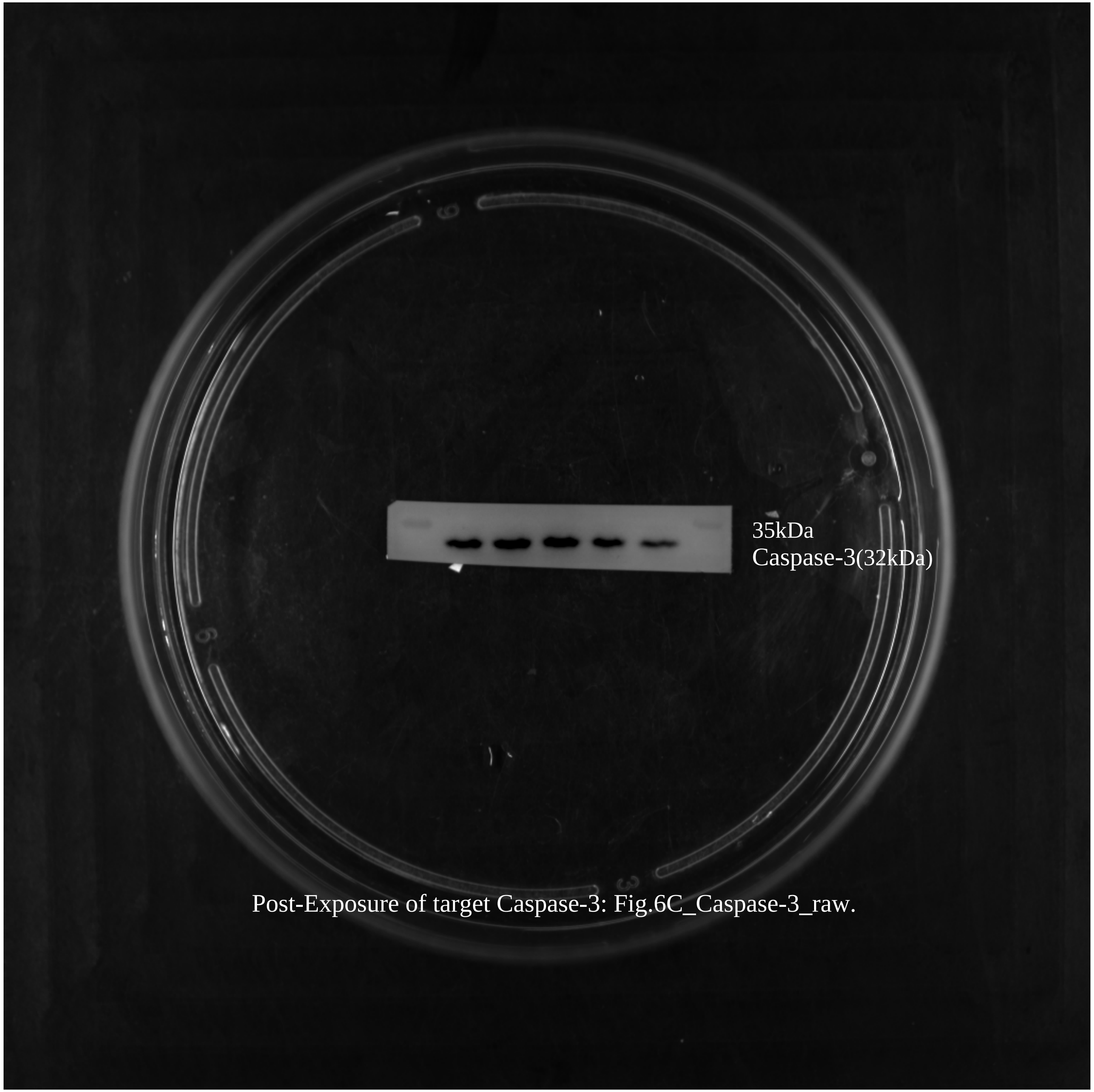

Con Mod NGR1 SSB2 NS

Caspase-3-1-β-Actin

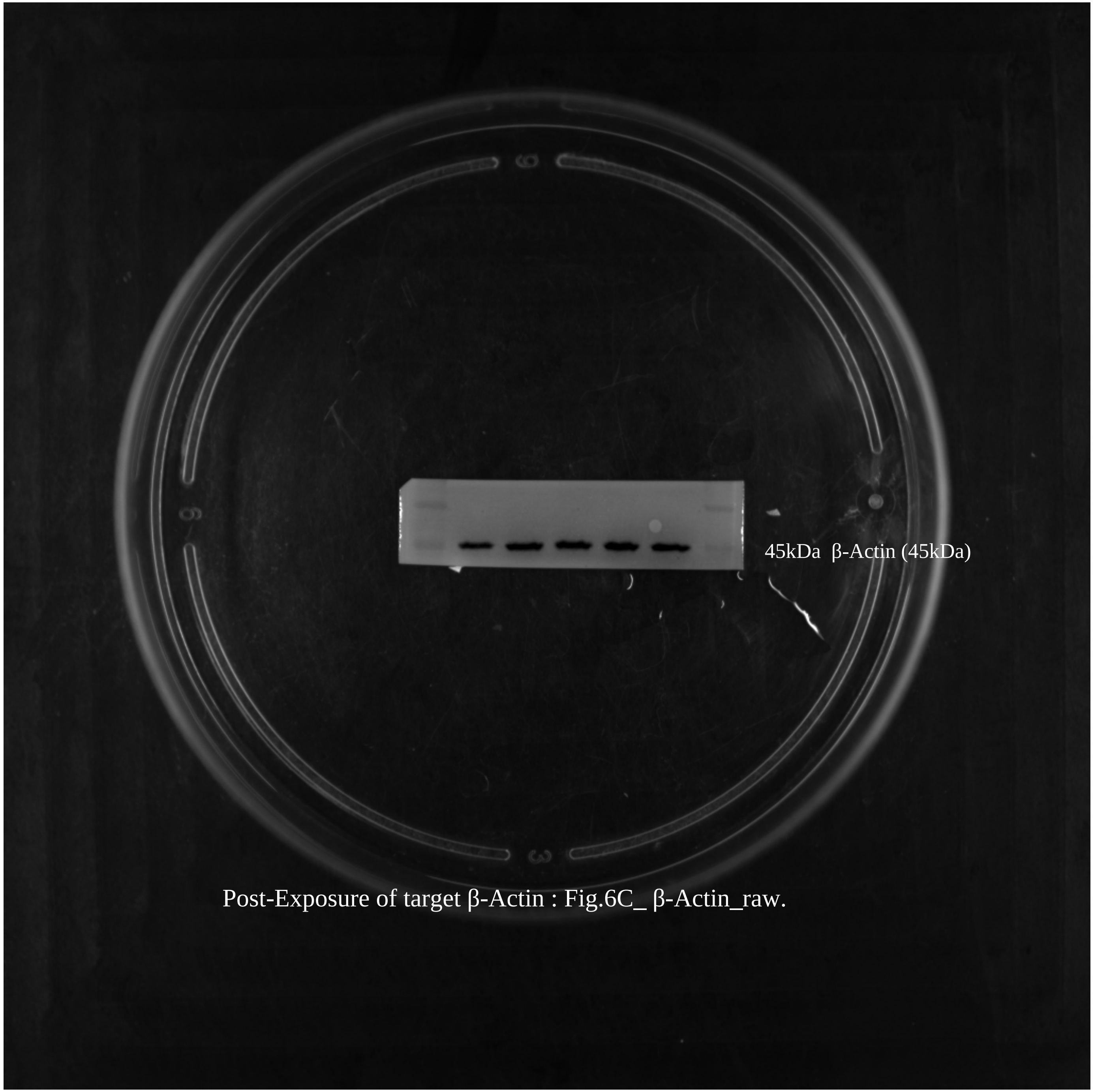

Con Mod NGR1 SSB2 NS

p-PI3K-1-full

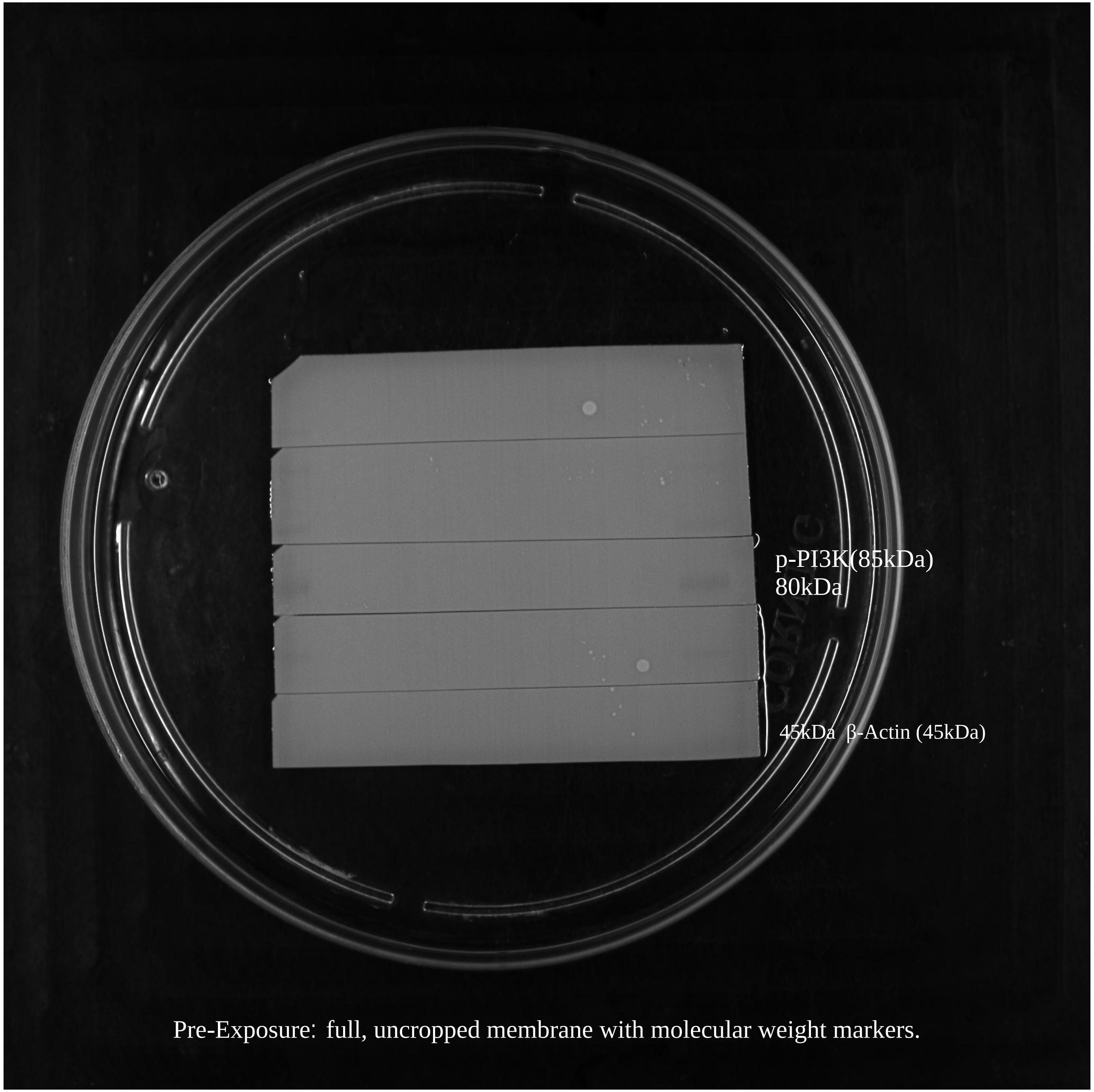

Con Mod NGR1 SSB2 NS

p-PI3K-1

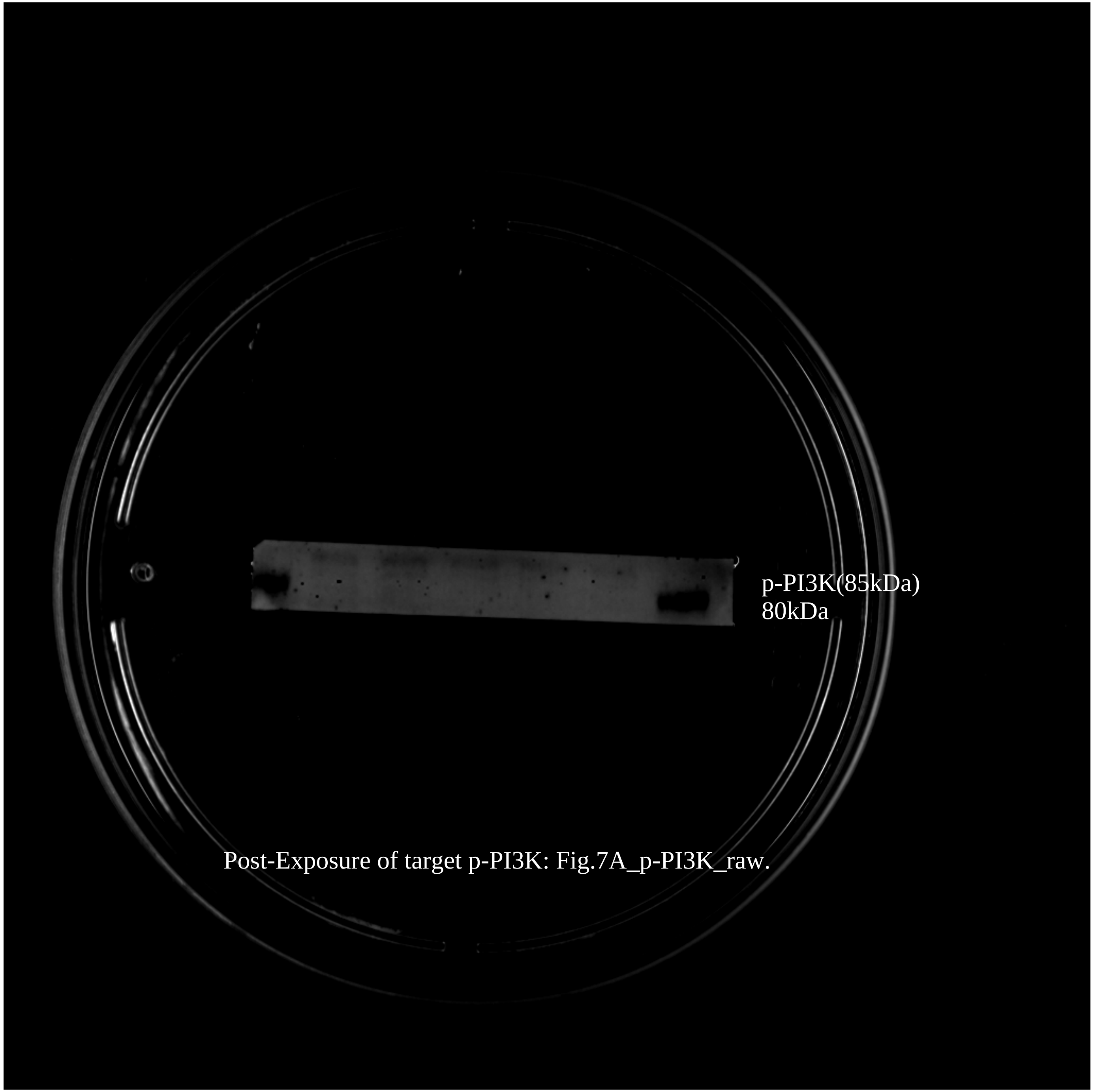

Con Mod NGR1 SSB2 NS

p-PI3K-1-β-Actin

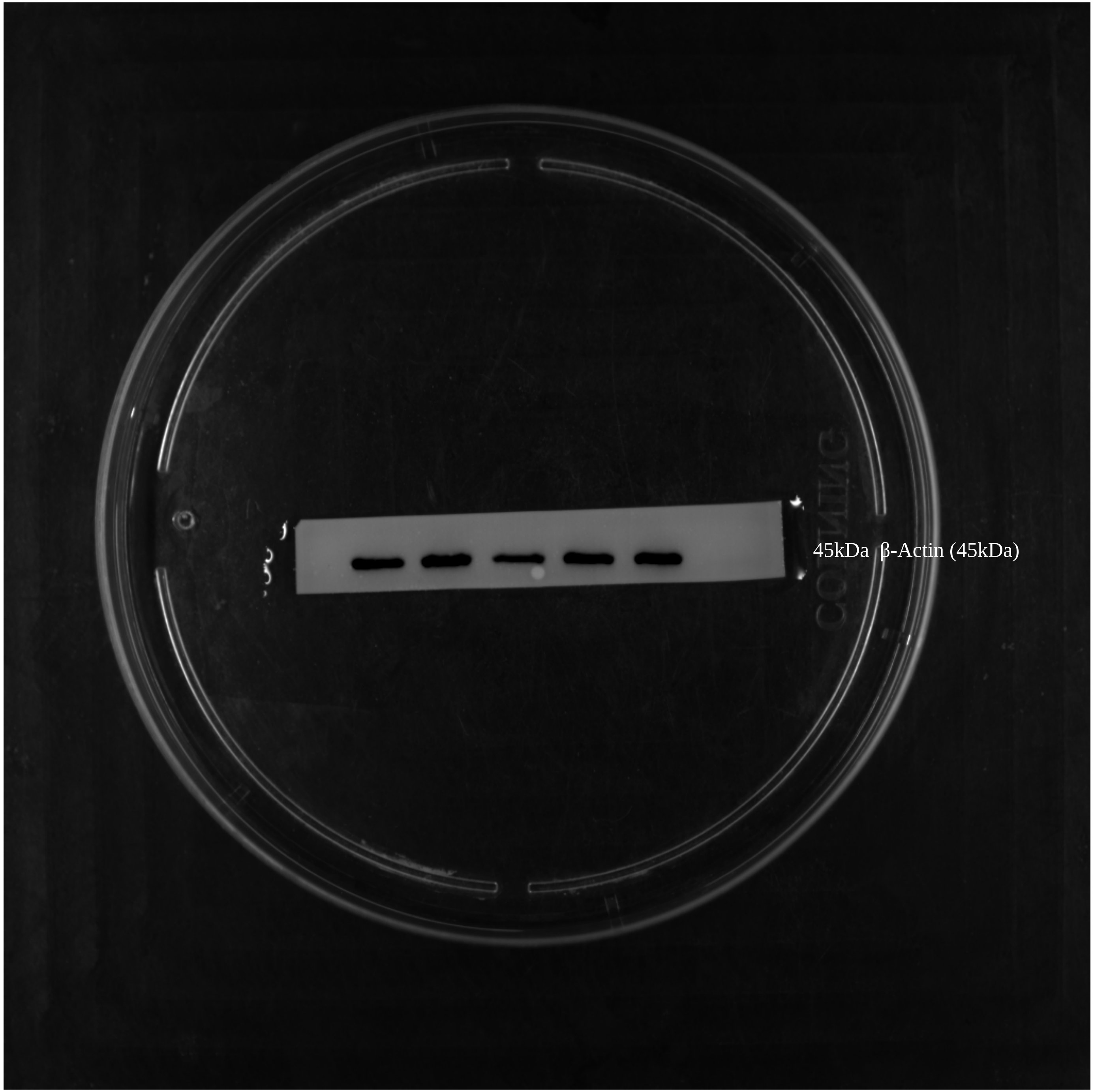

Con Mod NGR1 SSB2 NS

PI3K-1&2-full

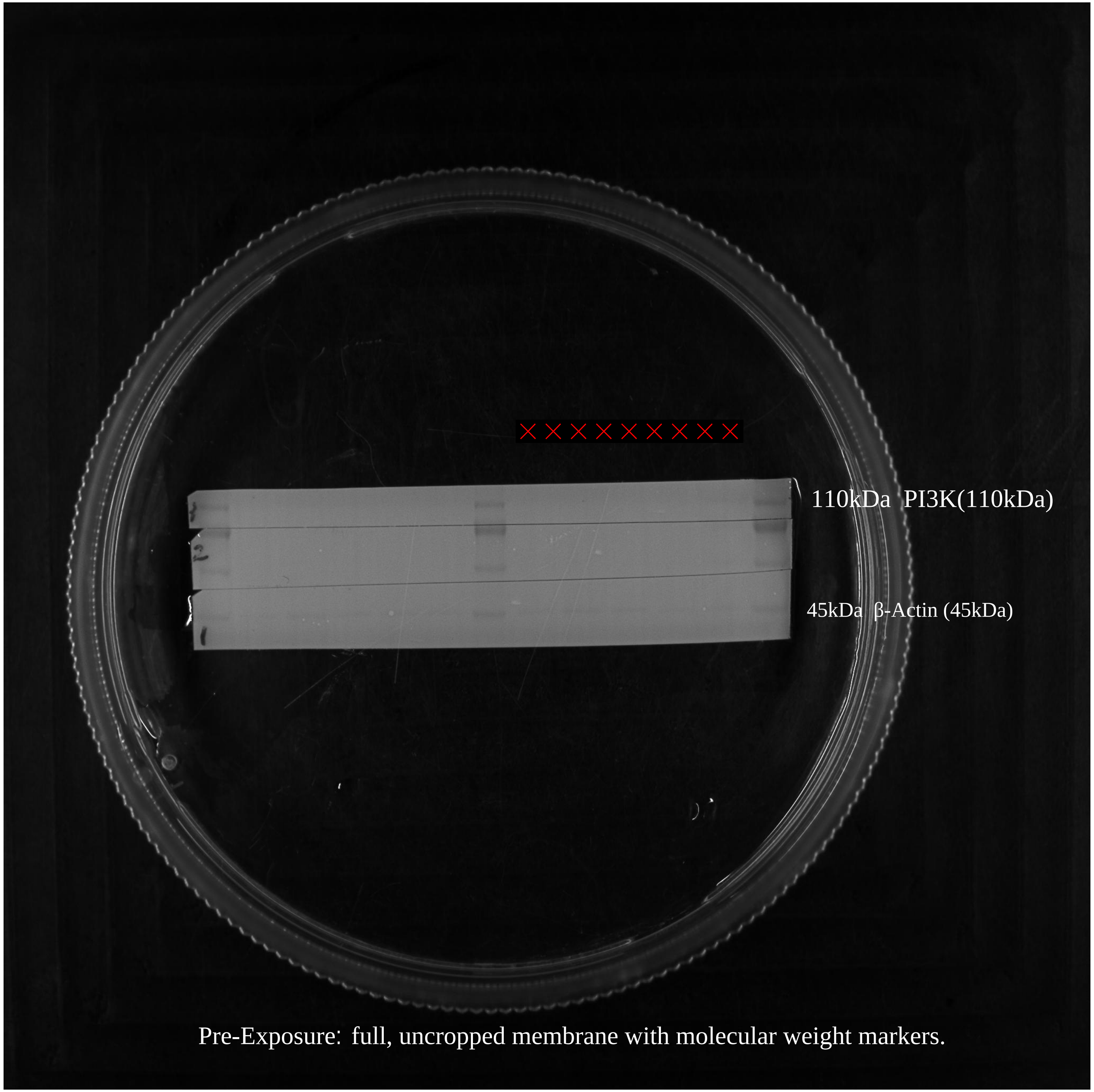

Con Mod NGR1 SSB2 NS Con Mod NGR1 SSB2 NS

PI3K-1&2

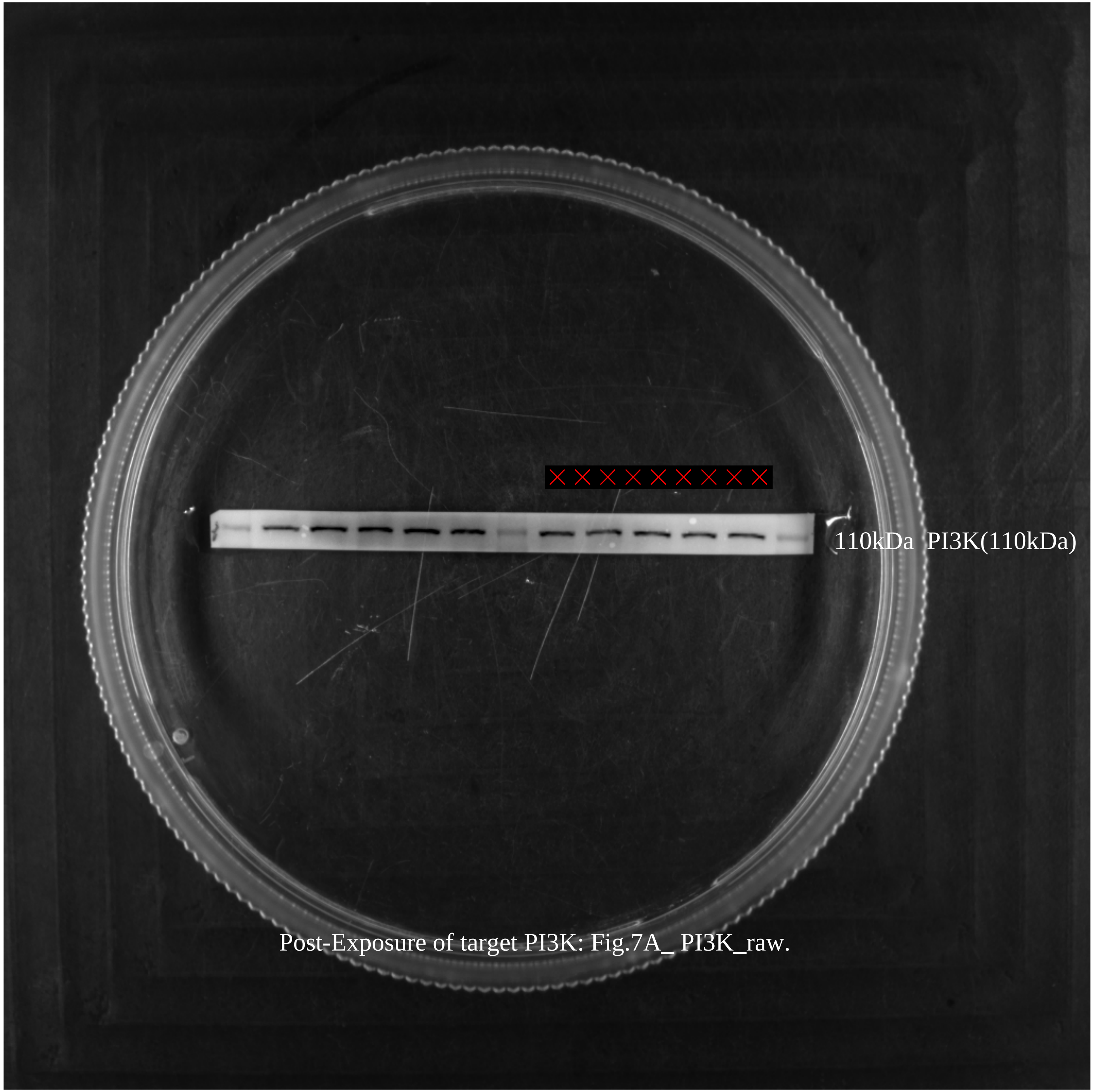

Con Mod NGR1 SSB2 NS Con Mod NGR1 SSB2 NS

PI3K-1&2-β-Actin

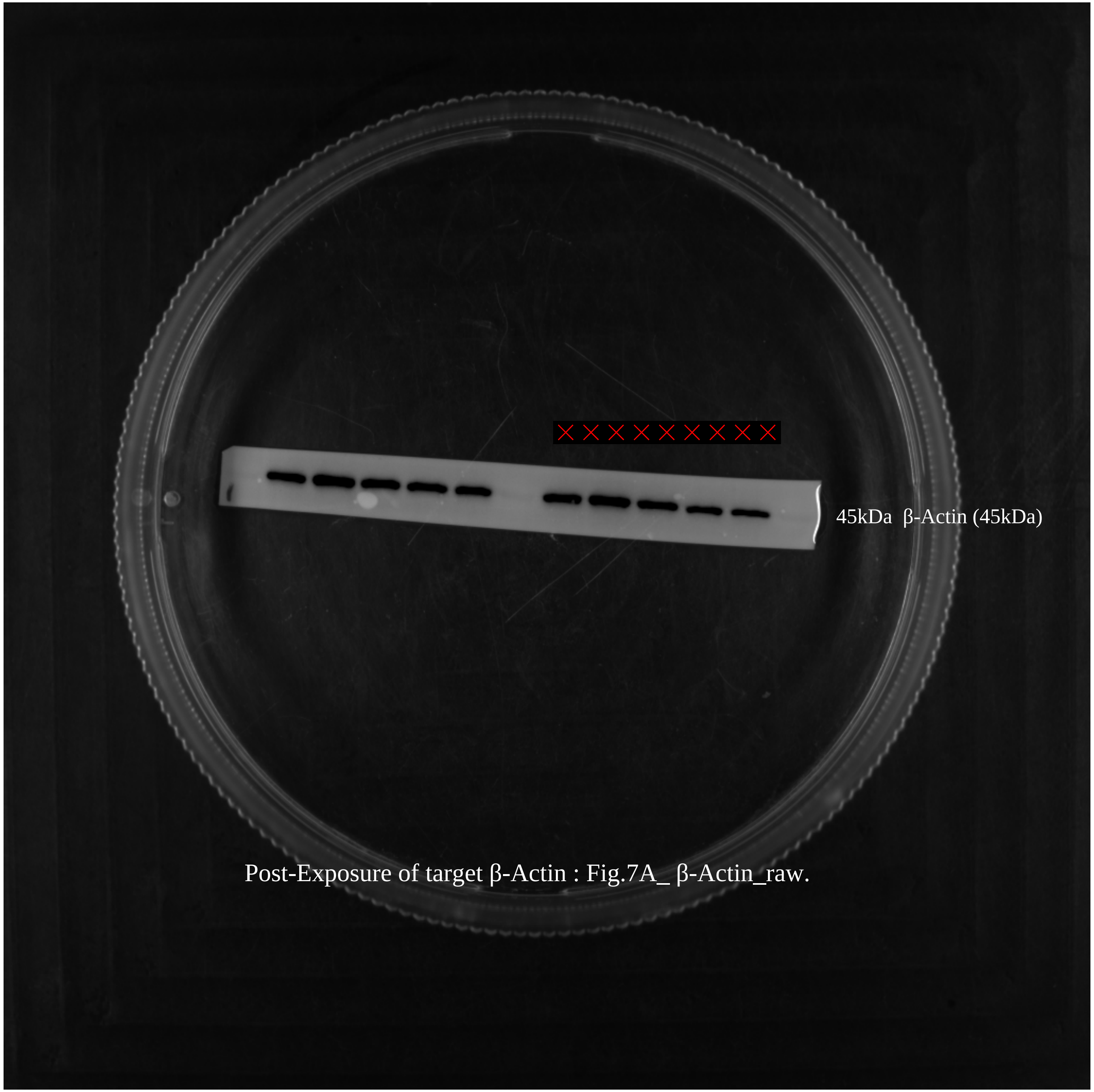

Con Mod NGR1 SSB2 NS Con Mod NGR1 SSB2 NS

p-AKT-1-full

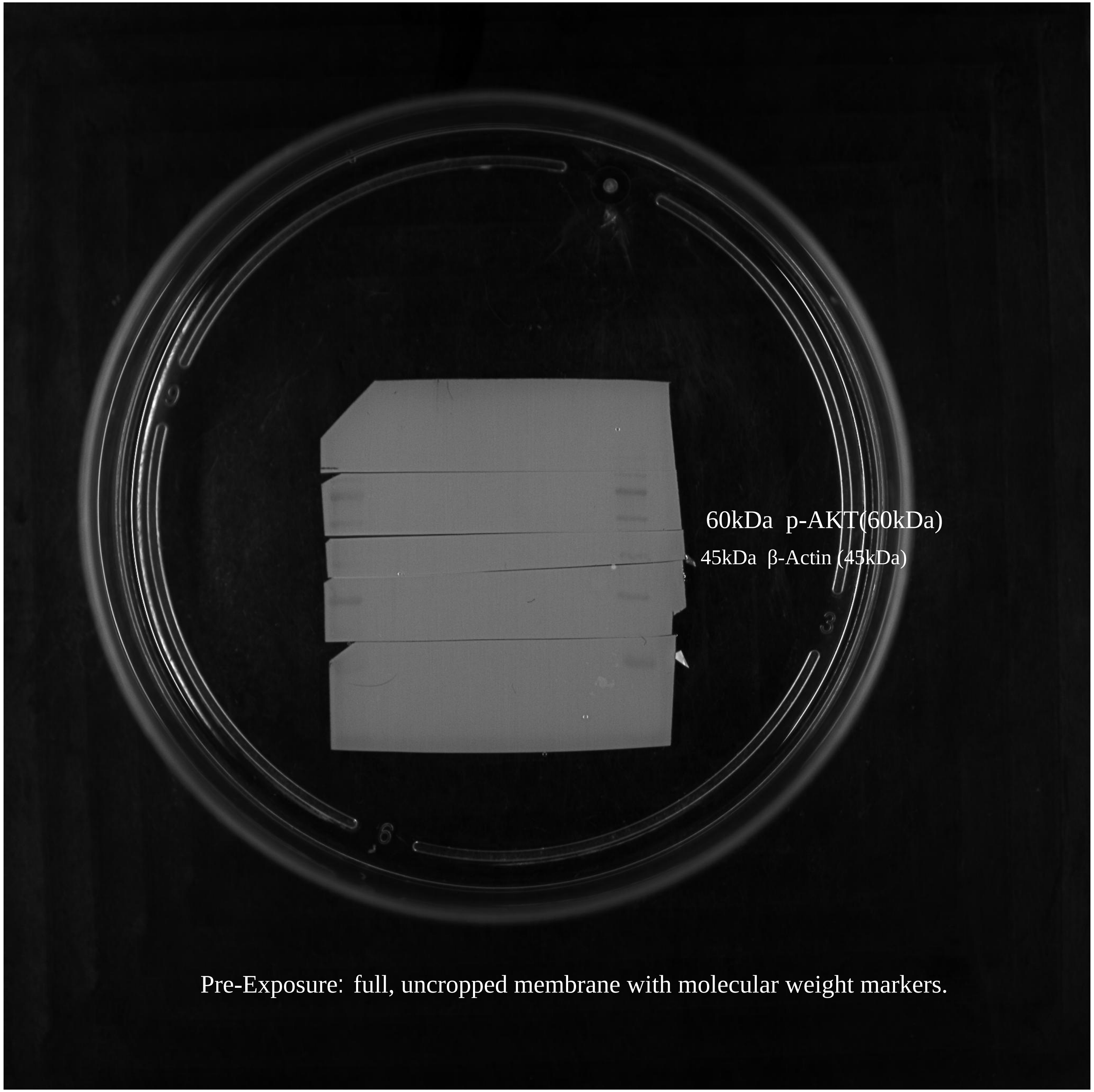

Con Mod NGR1 SSB2 NS

p-AKT-1

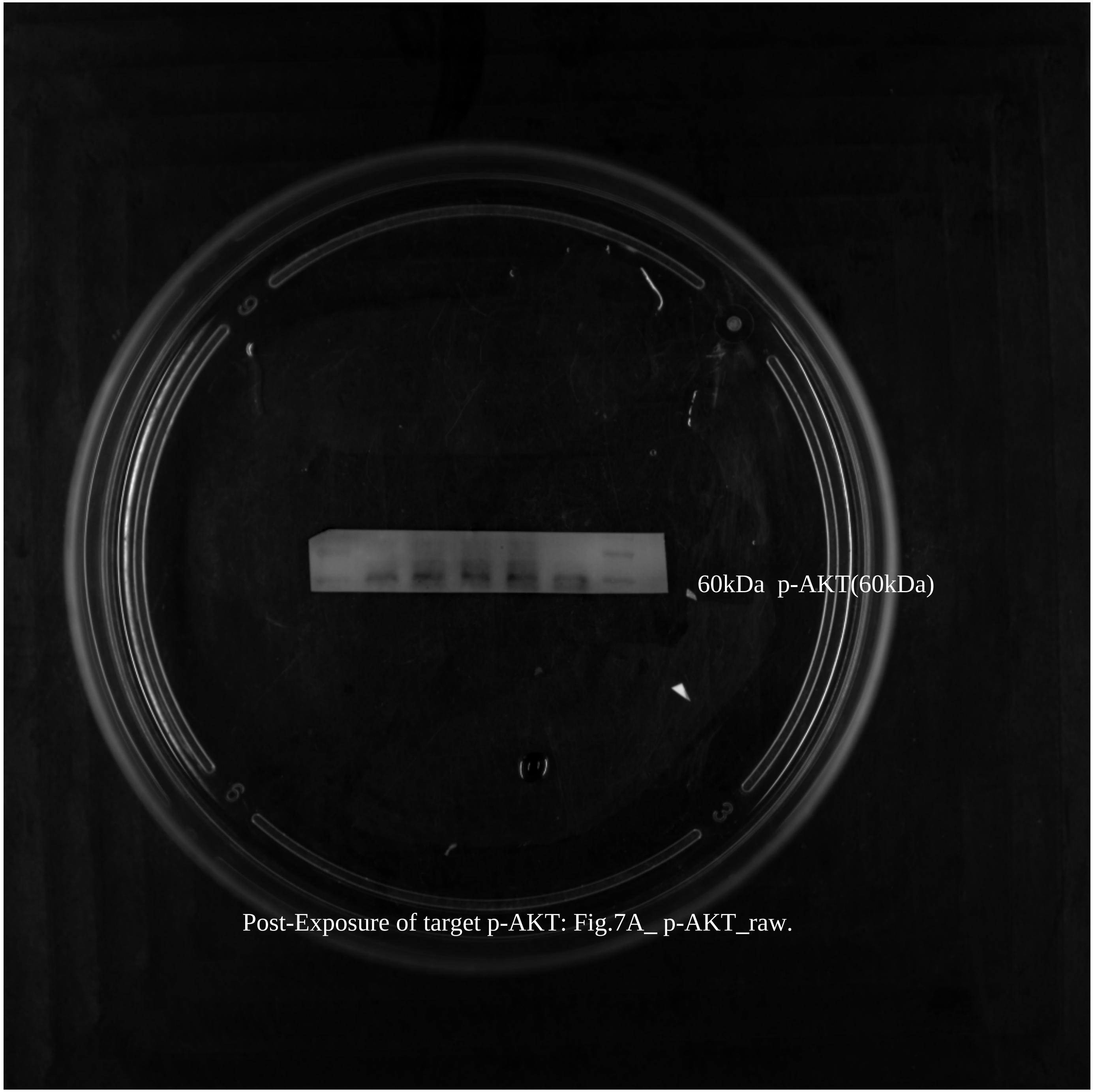

Con Mod NGR1 SSB2 NS

p-AKT-1-β-Actin

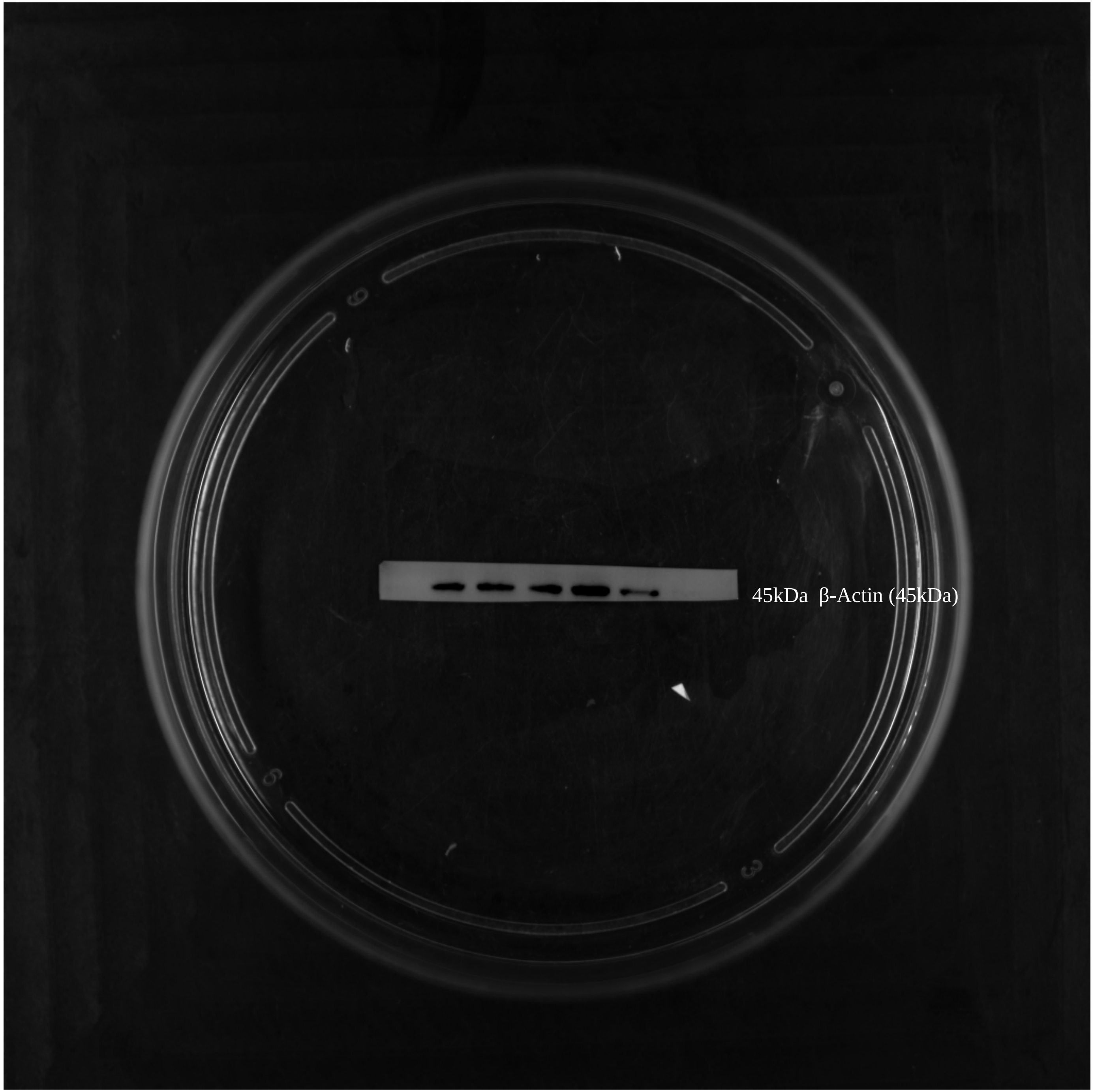

Con Mod NGR1 SSB2 NS

AKT-1&2-full

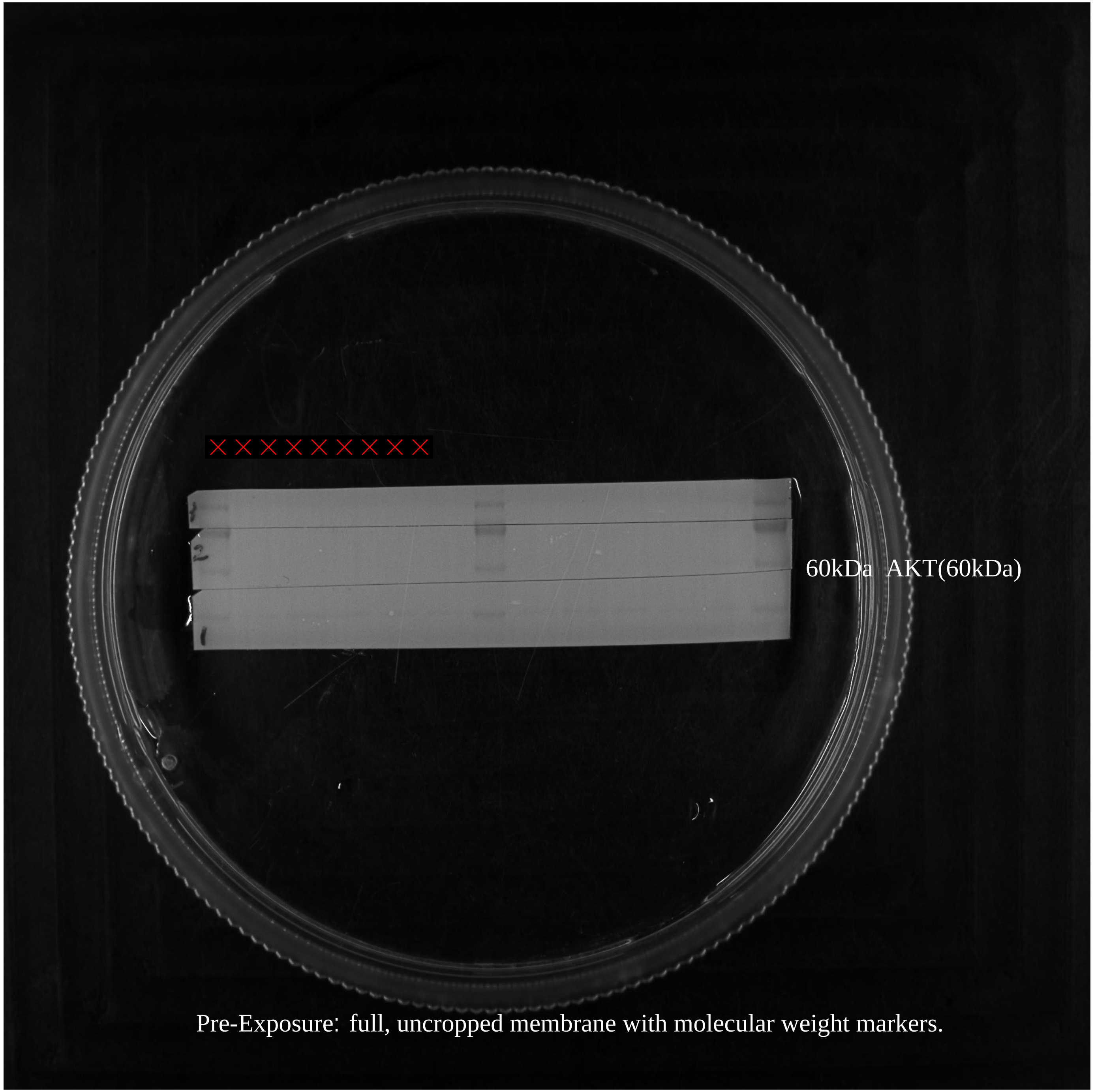

Con Mod NGR1 SSB2 NS Con Mod NGR1 SSB2 NS

AKT-1&2

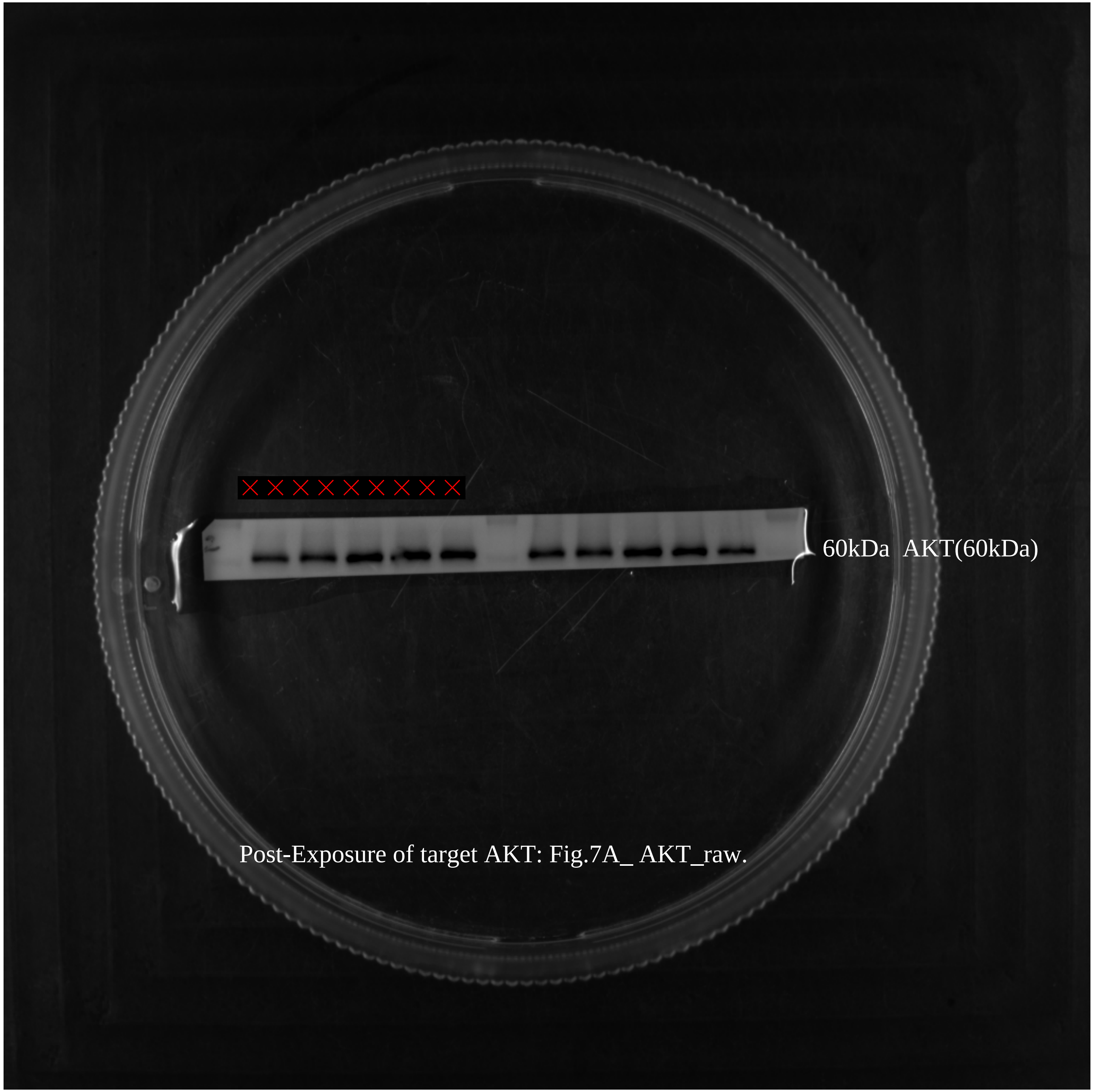

Con Mod NGR1 SSB2 NS   Con Mod NGR1 SSB2 NS

AKT-1&2-β-Actin

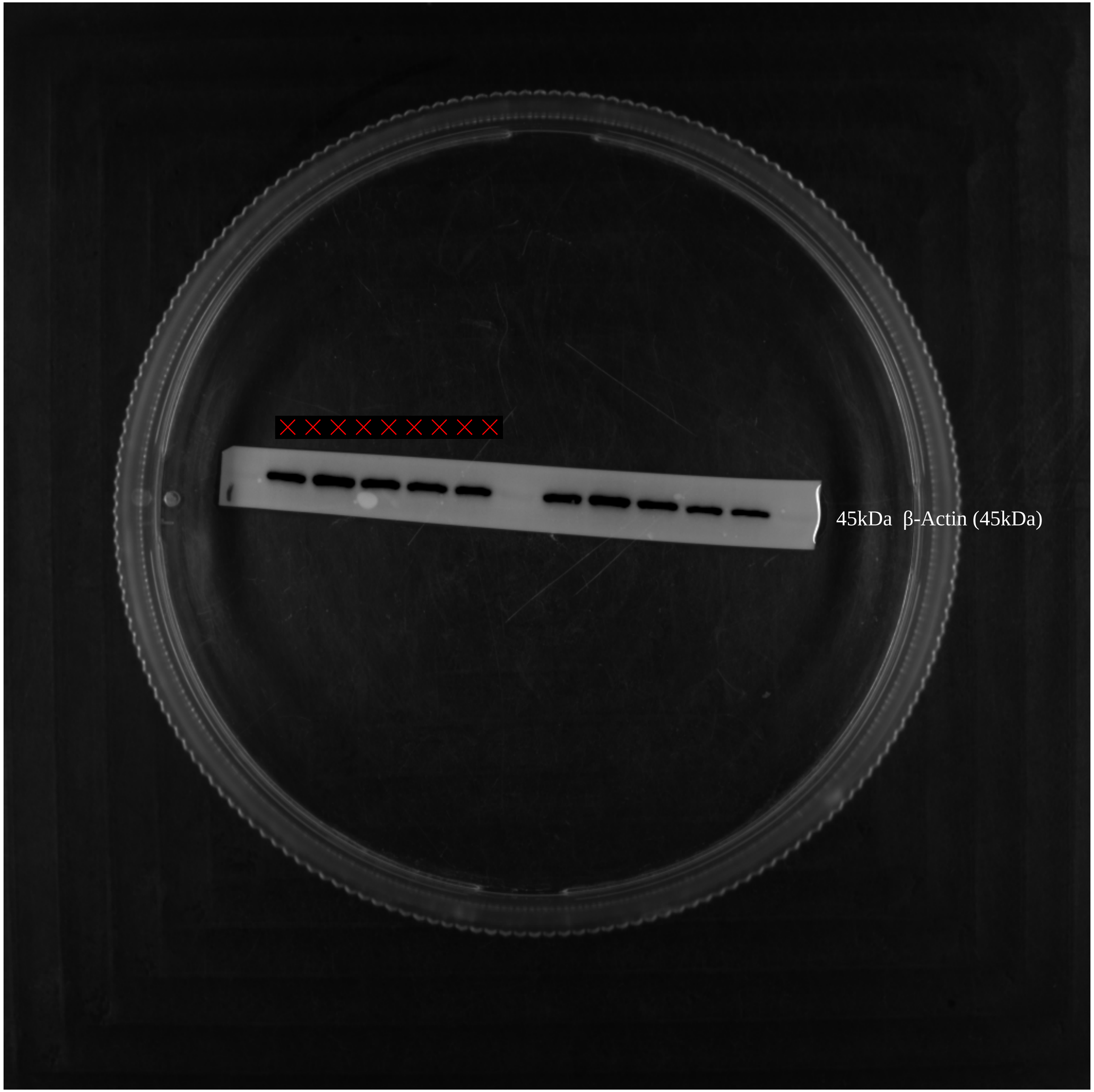

Con Mod NGR1 SSB2 NS Con Mod NGR1 SSB2 NS

p-mTOR-1&2-full

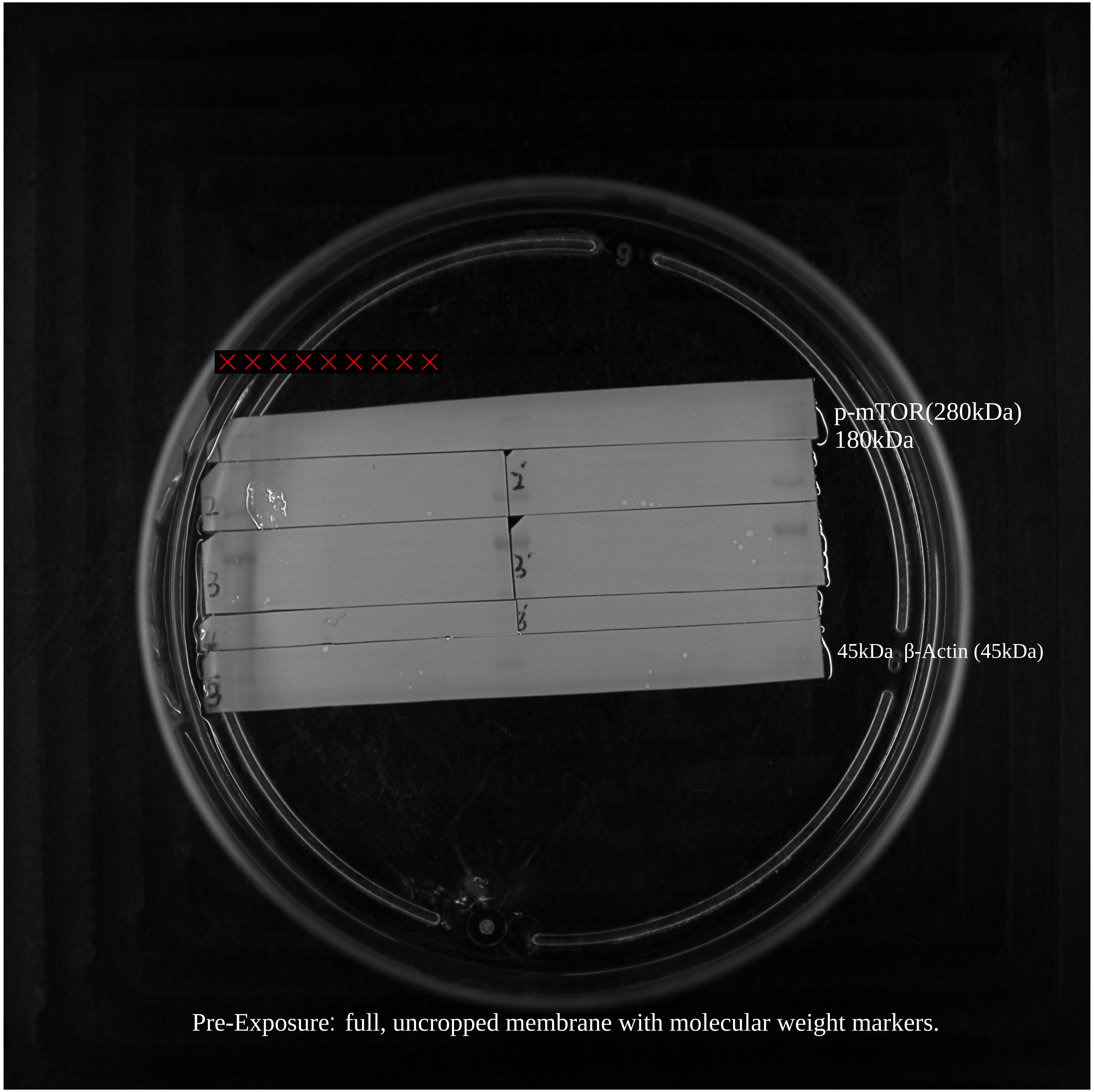

Con Mod NGR1 SSB2 NS Con Mod NGR1 SSB2 NS

p-mTOR-1&2

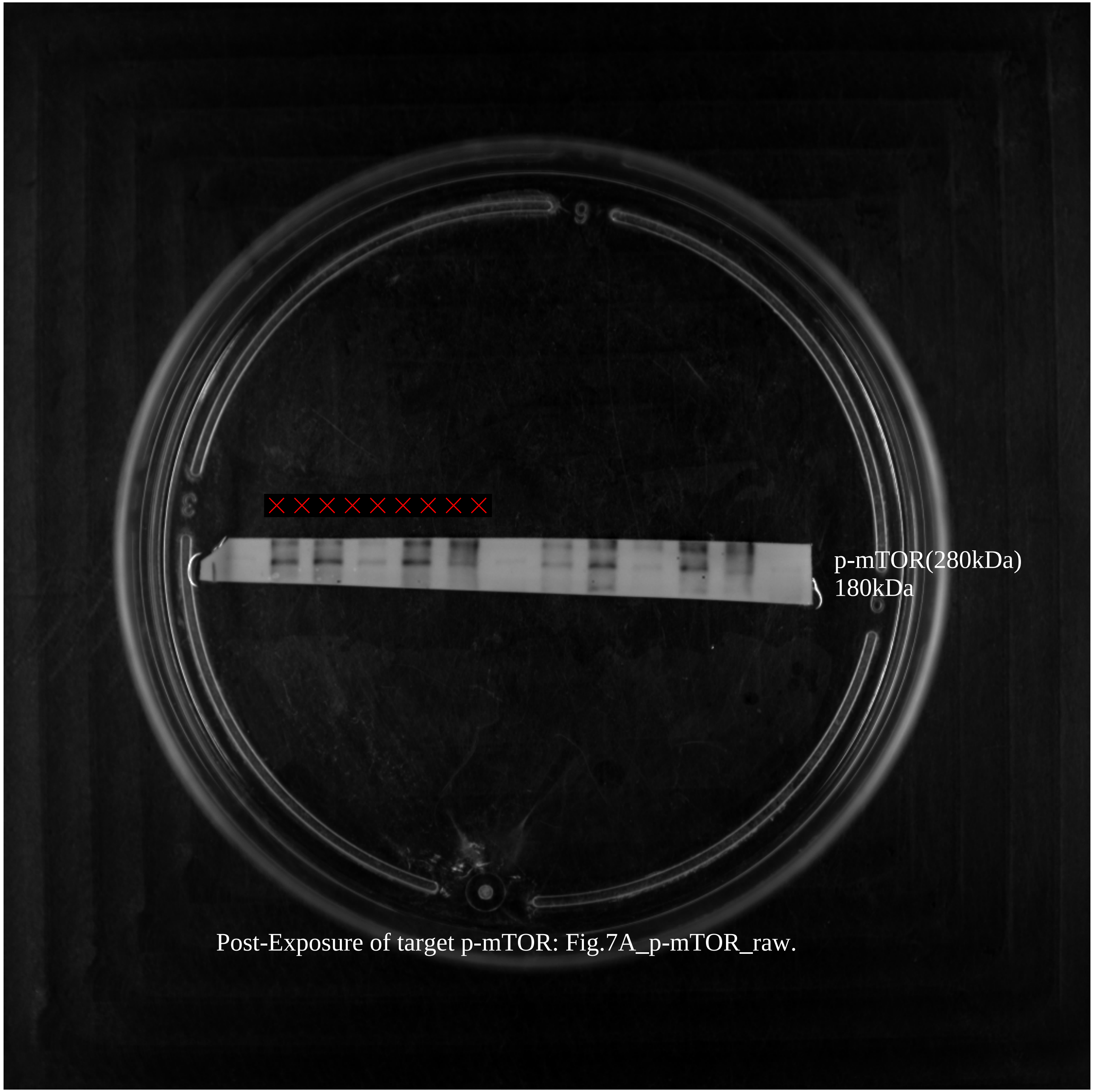

Con Mod NGR1 SSB2 NS Con Mod NGR1 SSB2 NS

p-mTOR-1&2-β-Actin

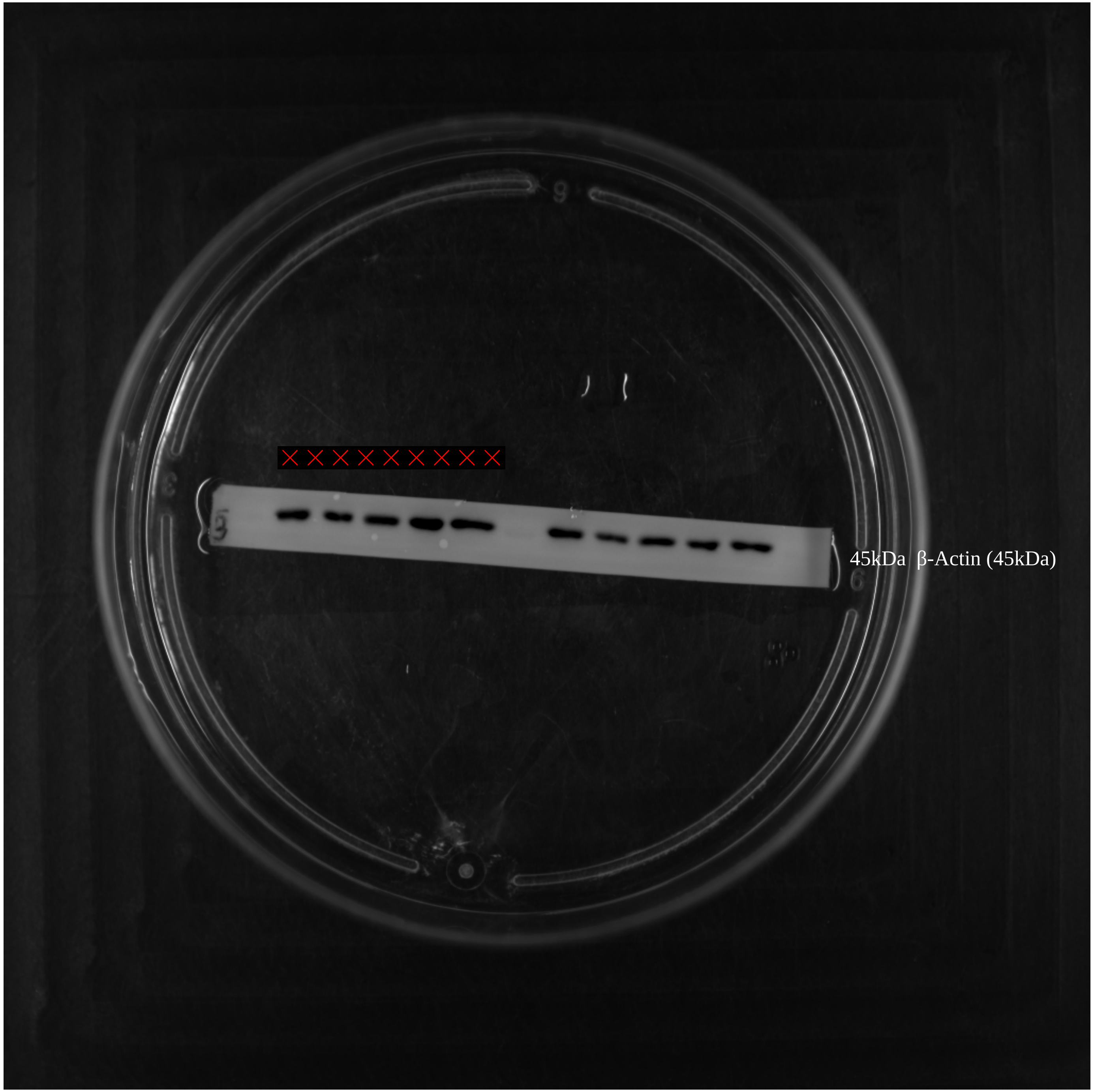

Con Mod NGR1 SSB2 NS   Con Mod NGR1 SSB2 NS

mTOR-1&2-full

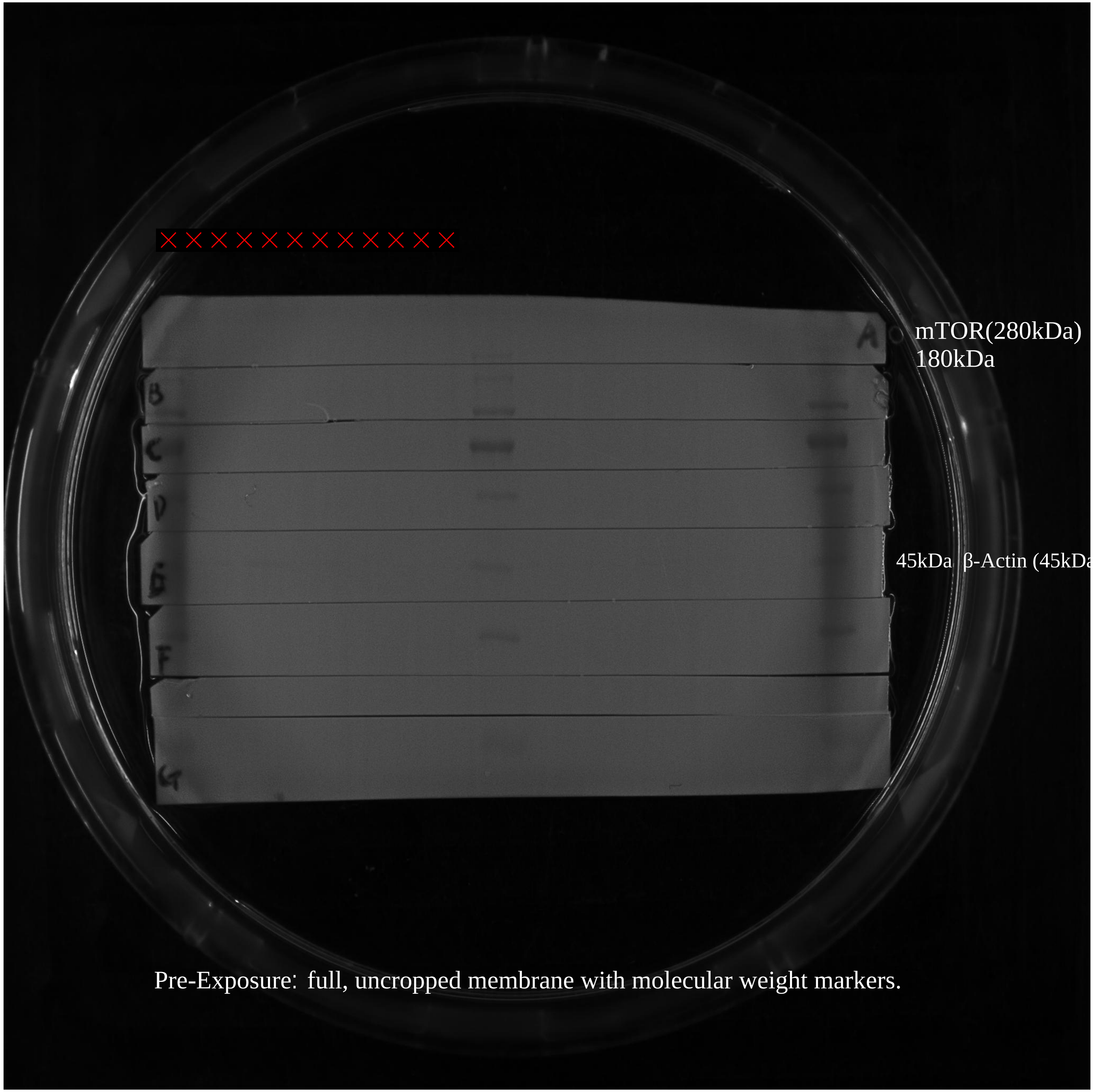

Pre-Exposure: full, uncropped membrane with molecular weight markers.

Con Mod NGR1 SSB2 NS   Con Mod NGR1 SSB2 NS

mTOR-1&2

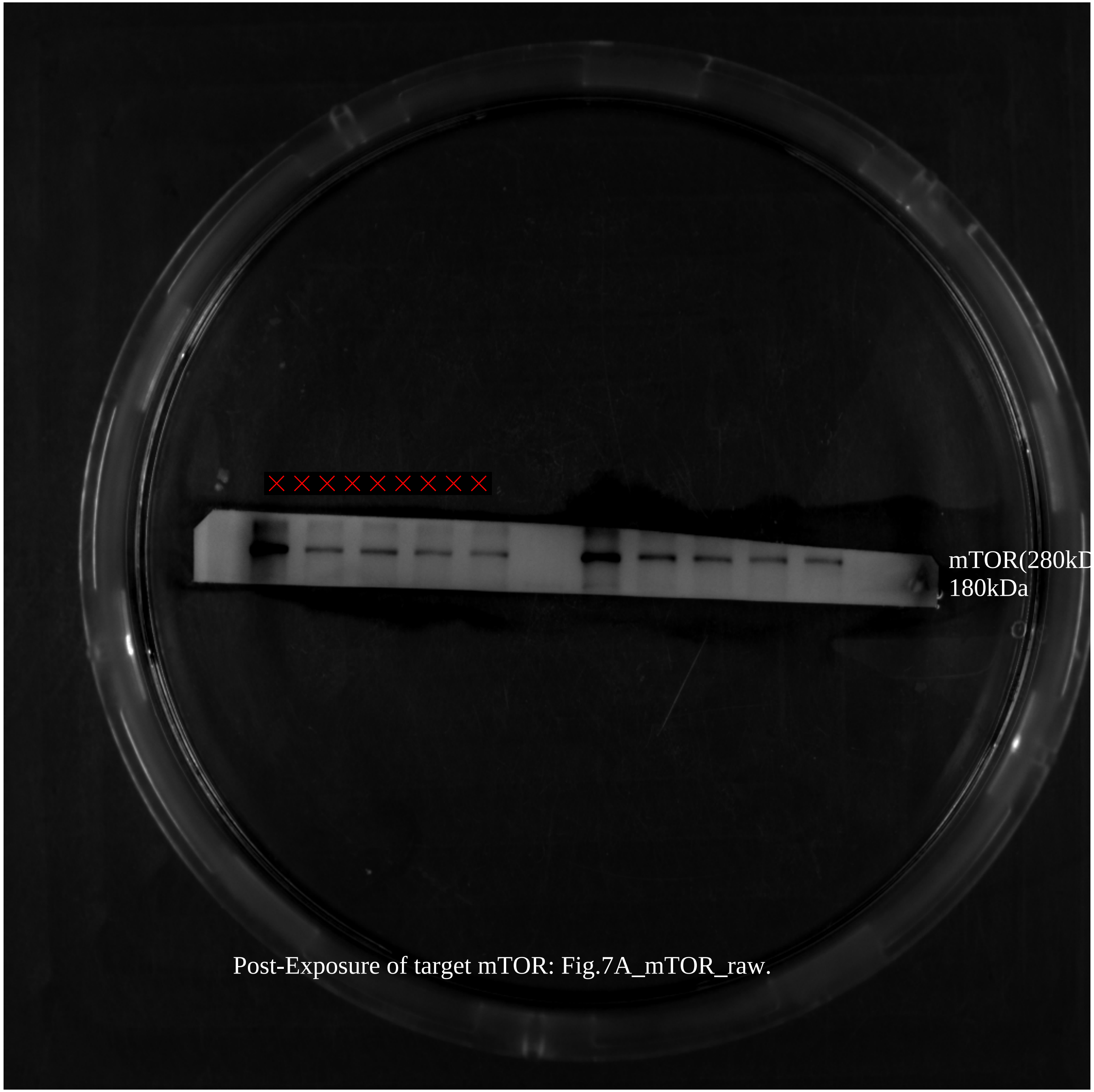

Con Mod NGR1 SSB2 NS Con Mod NGR1 SSB2 NS

mTOR-1&2-β-Actin

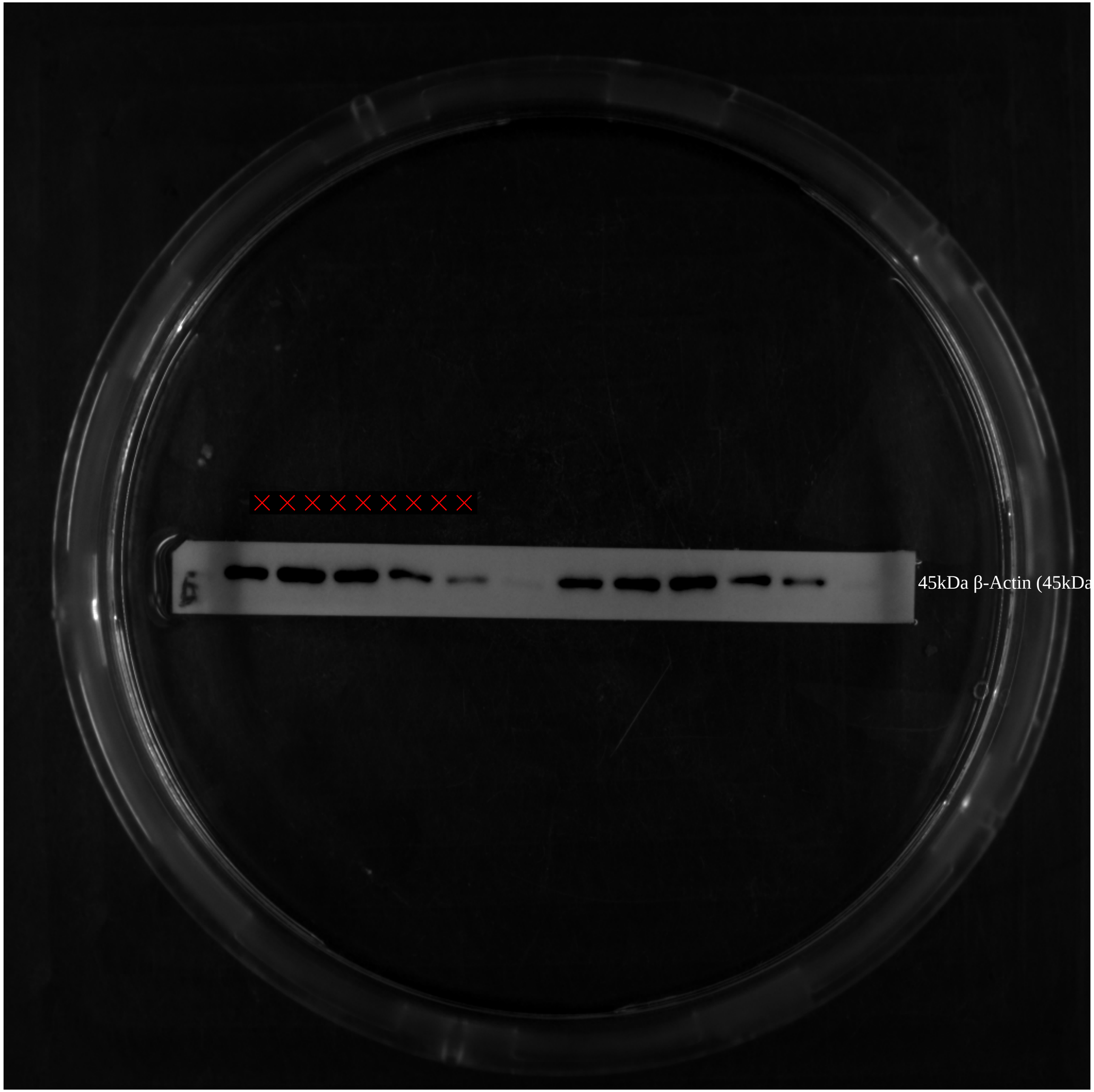

Con Mod NGR1 SSB2 NS Con Mod NGR1 SSB2 NS

Beclin1-1-full

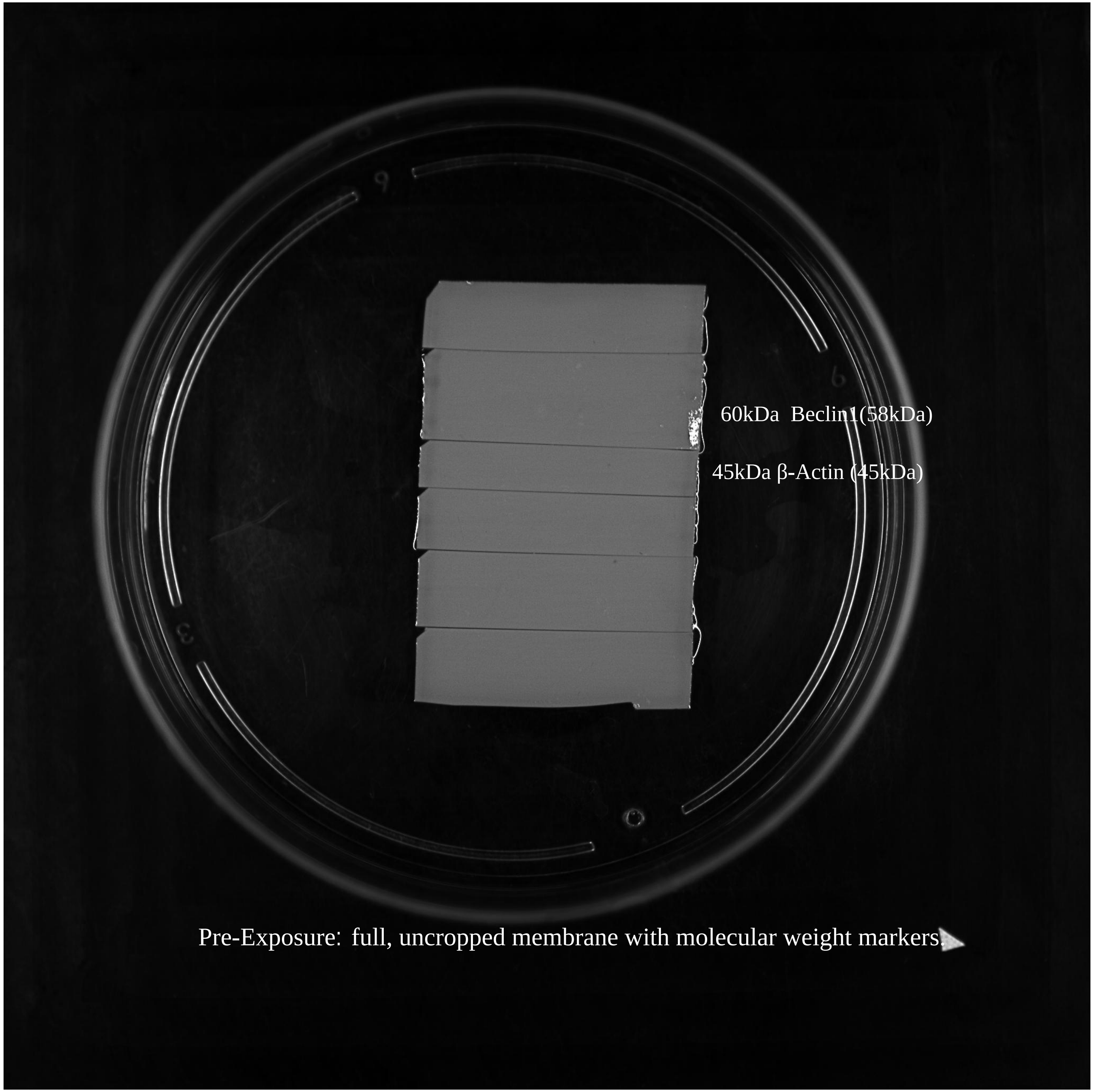

Con Mod NGR1 SSB2 NS

Beclin1-1

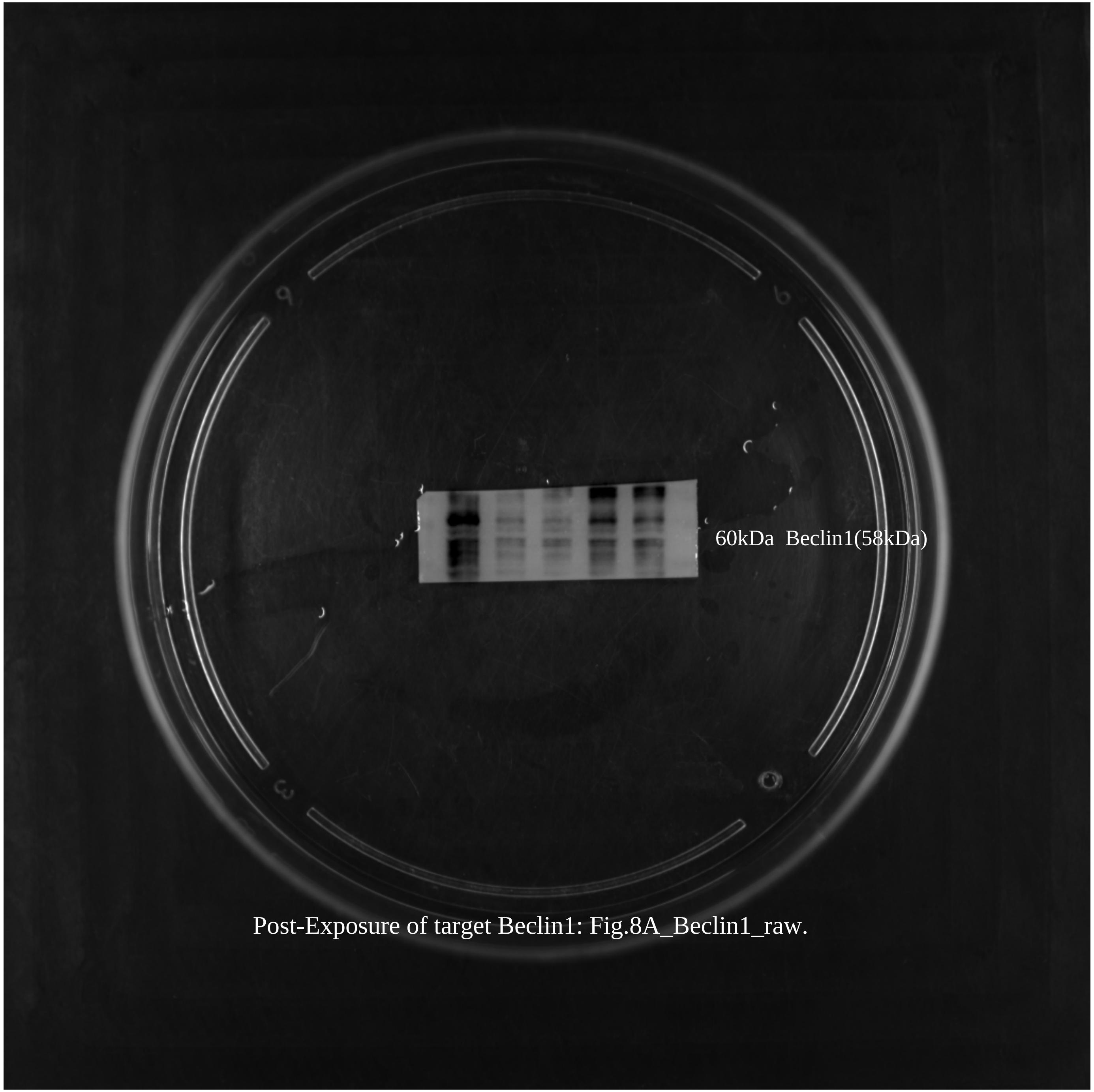

Con Mod NGR1 SSB2 NS

Beclin1-1-β-Actin

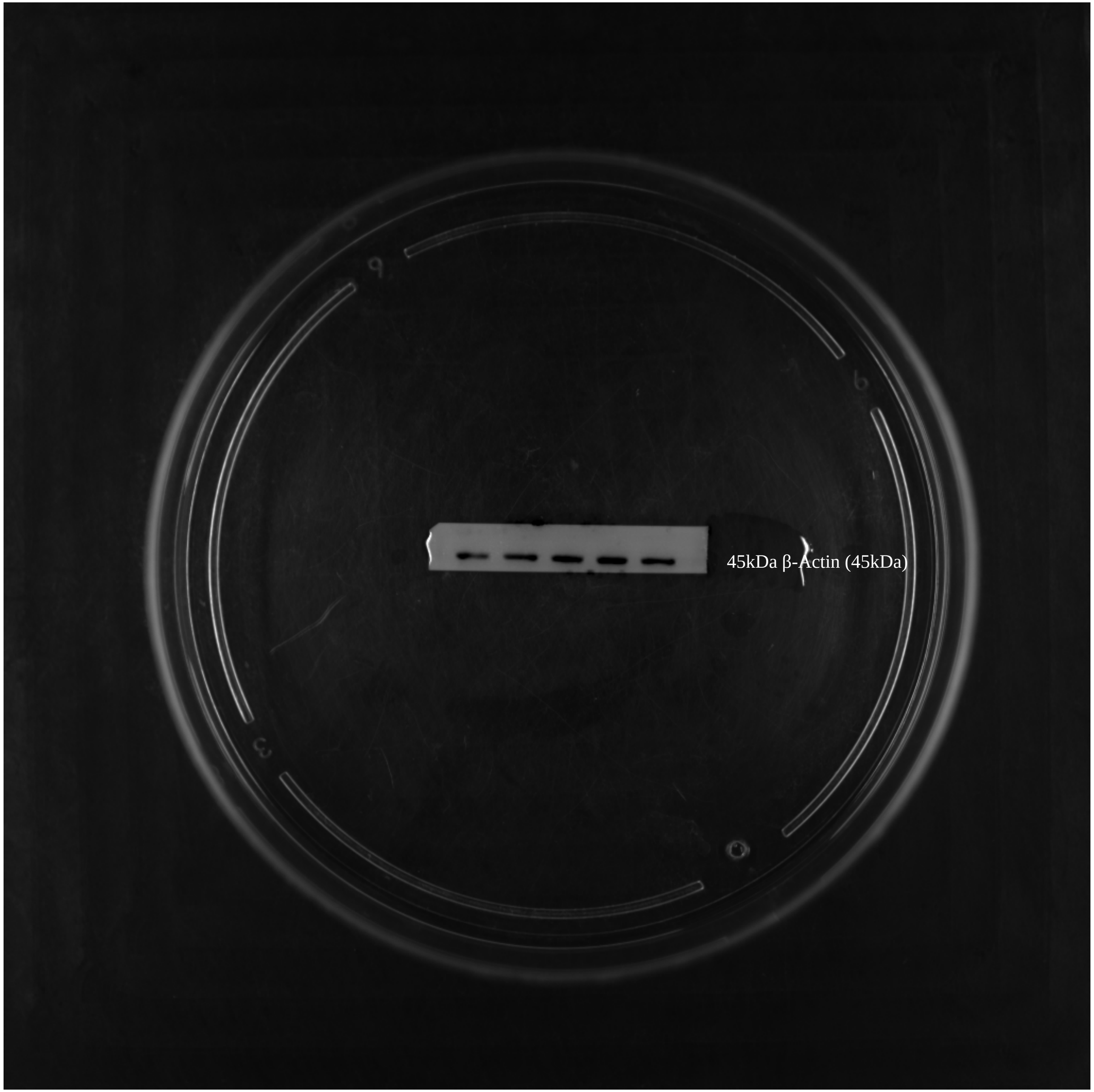

Con Mod NGR1 SSB2 NS

LC3B-1&2-full

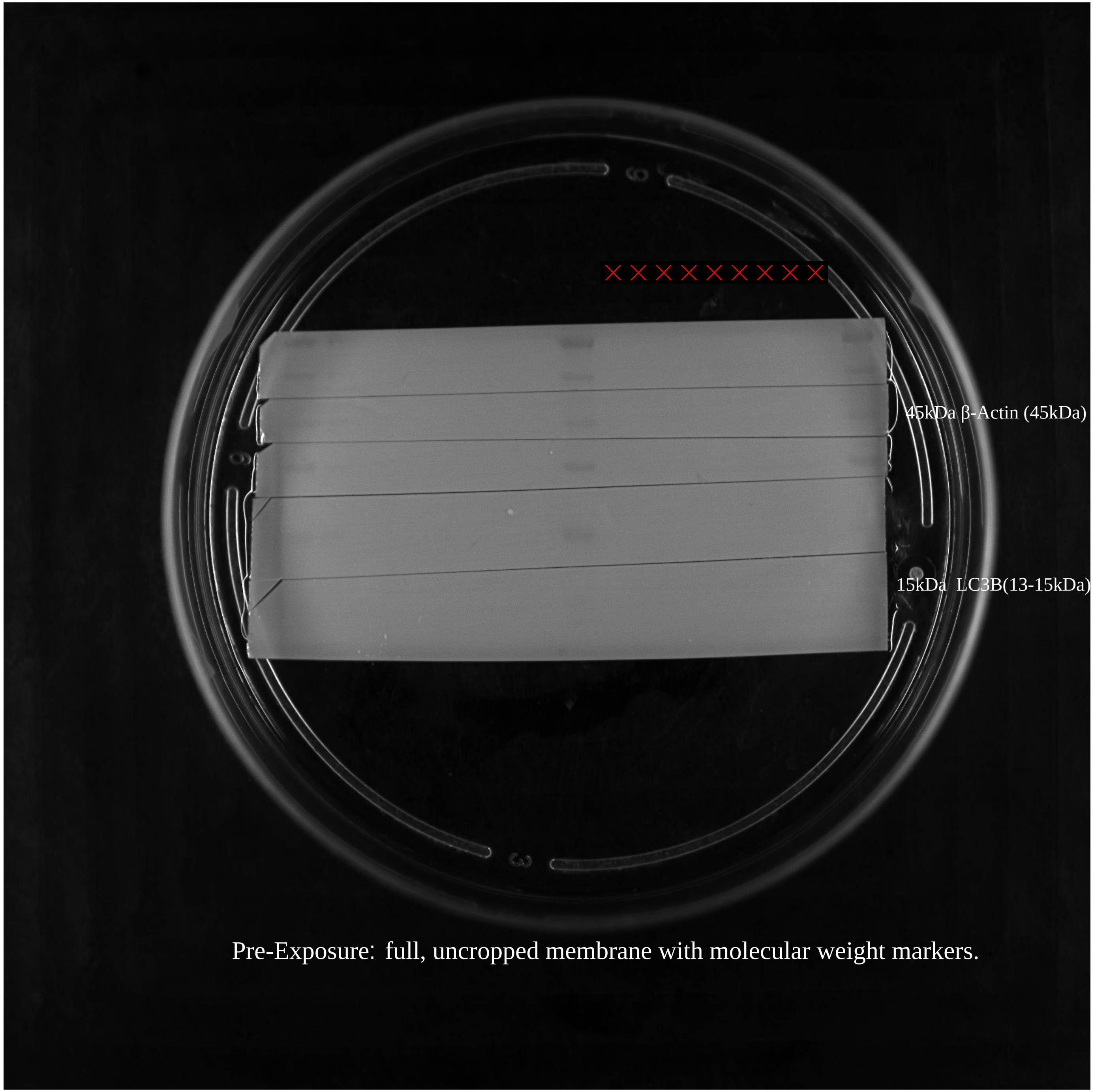

Con Mod NGR1 SSB2 NS   Con Mod NGR1 SSB2 NS

LC3B-1&2

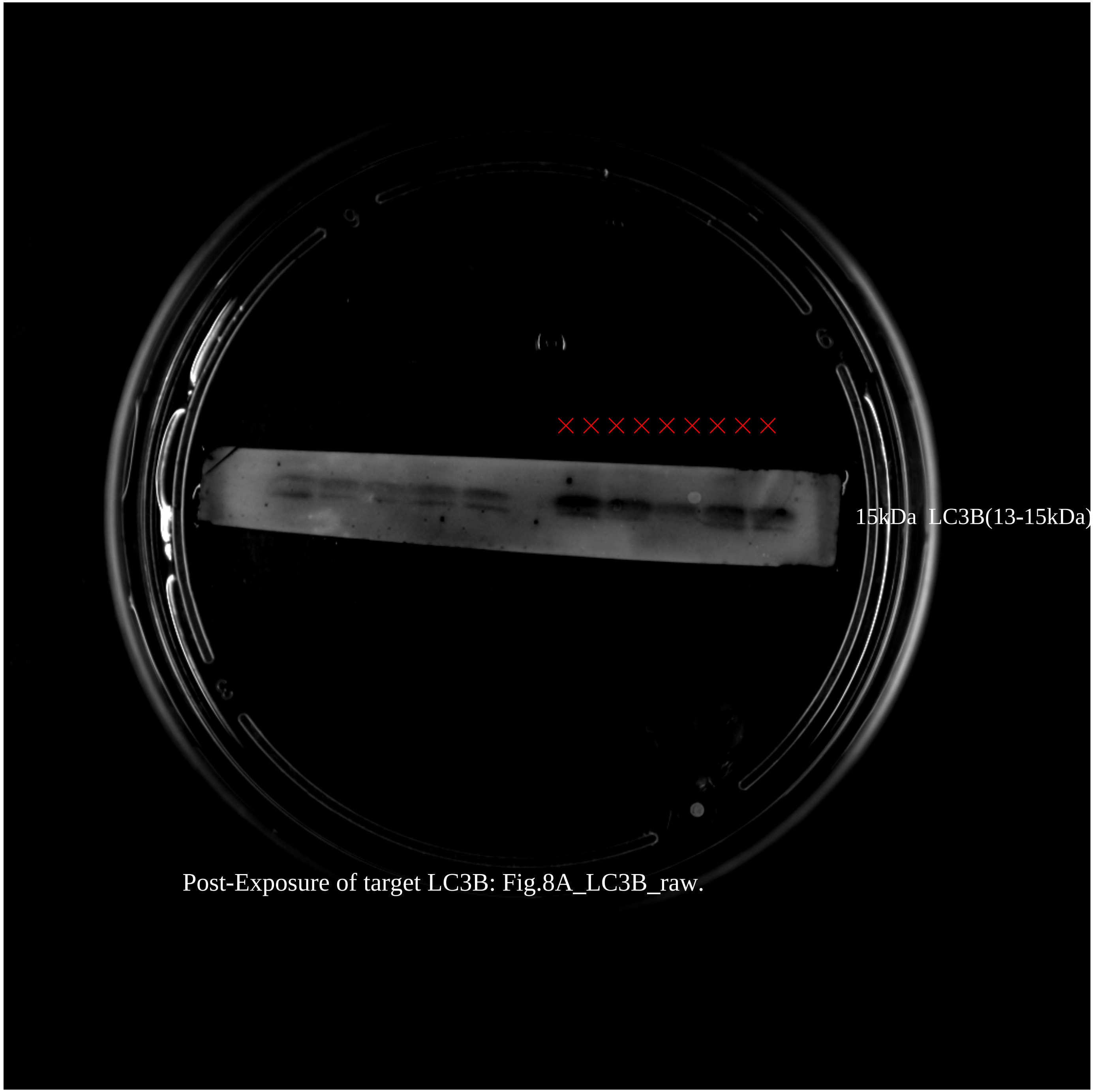

Con Mod NGR1 SSB2 NS   Con Mod NGR1 SSB2 NS

LC3B-1&2-β-Actin

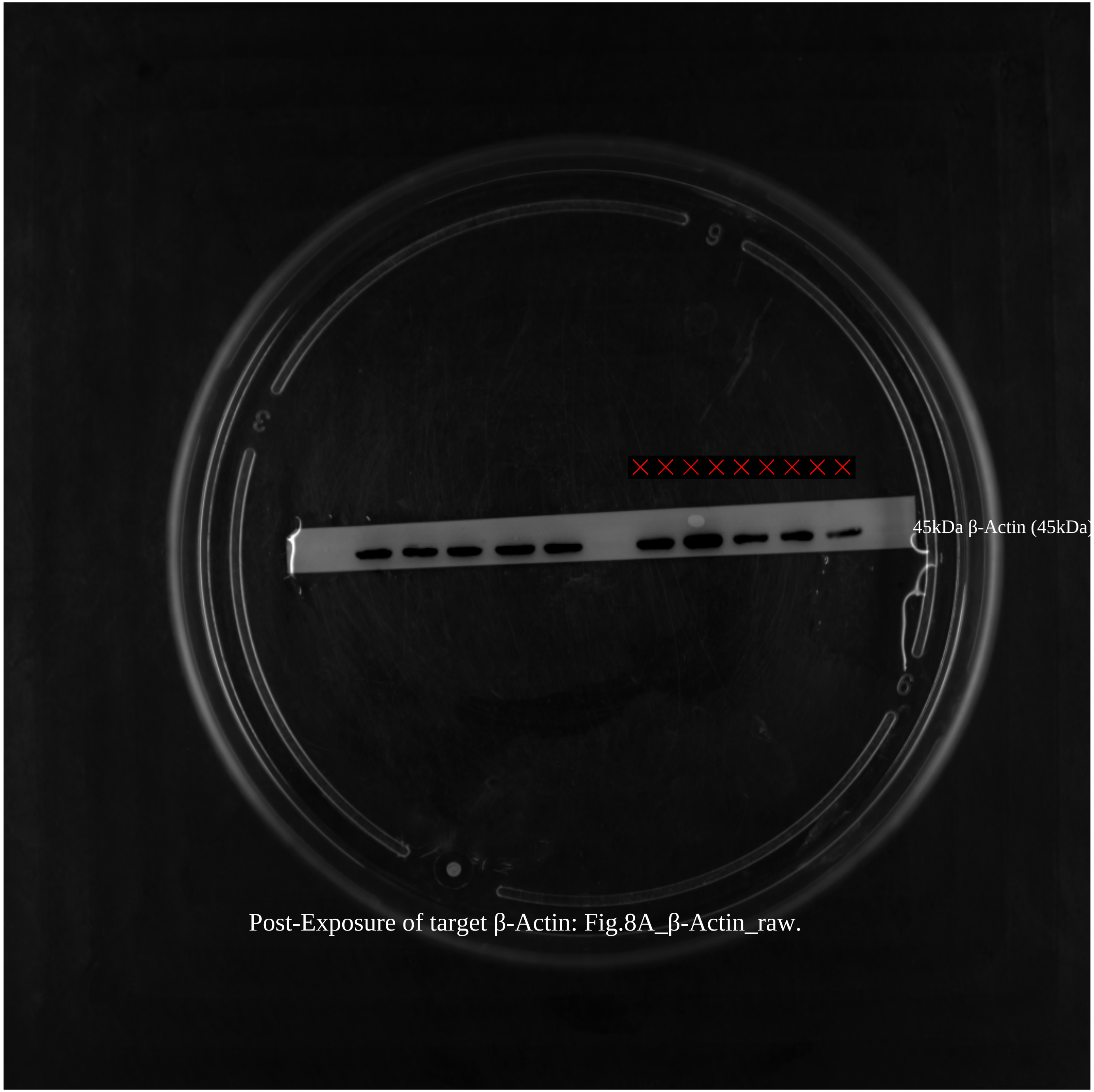

Con Mod NGR1 SSB2 NS Con Mod NGR1 SSB2 NS

P62-1-full

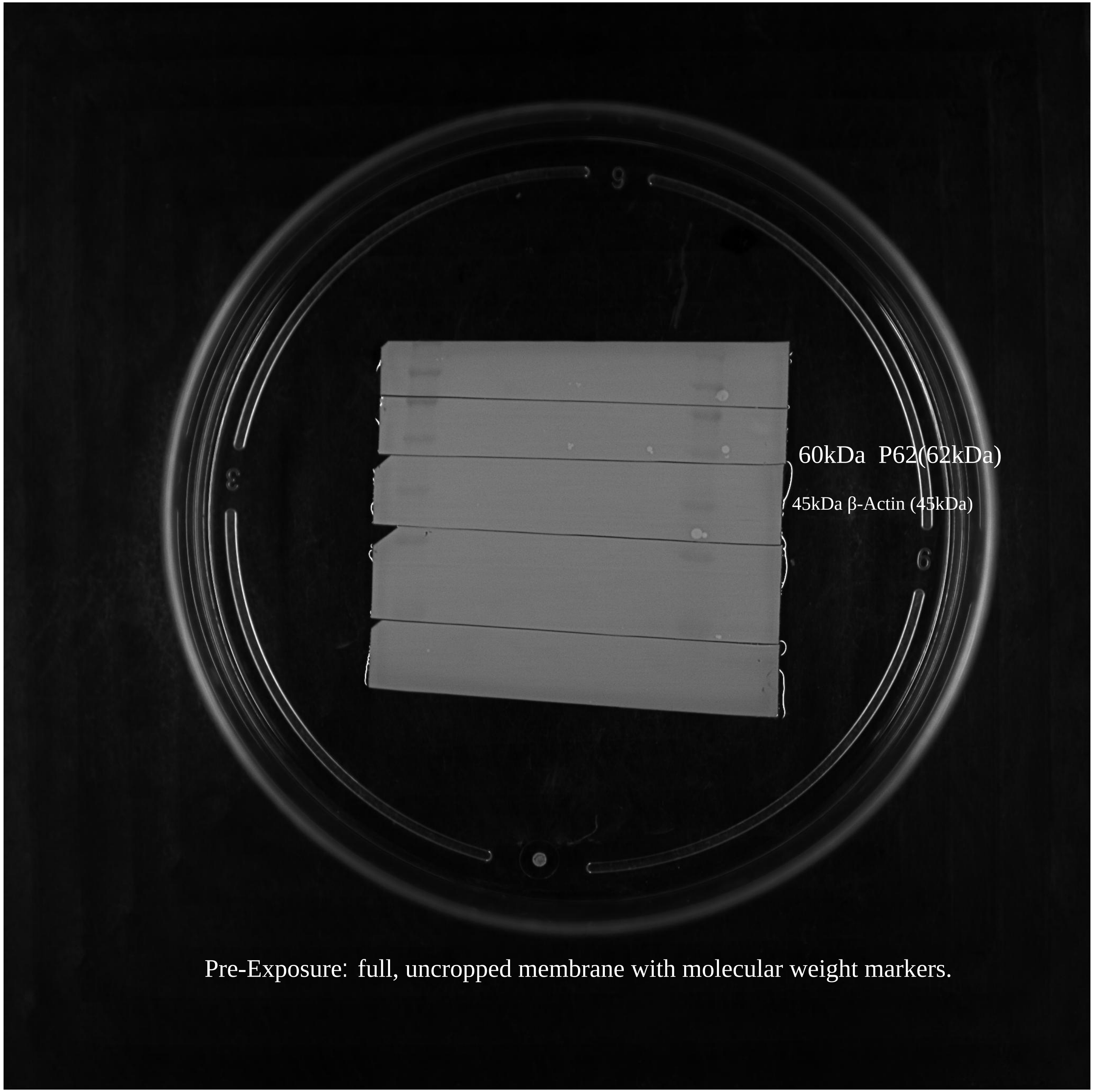

Con Mod NGR1 SSB2 NS

P62-1

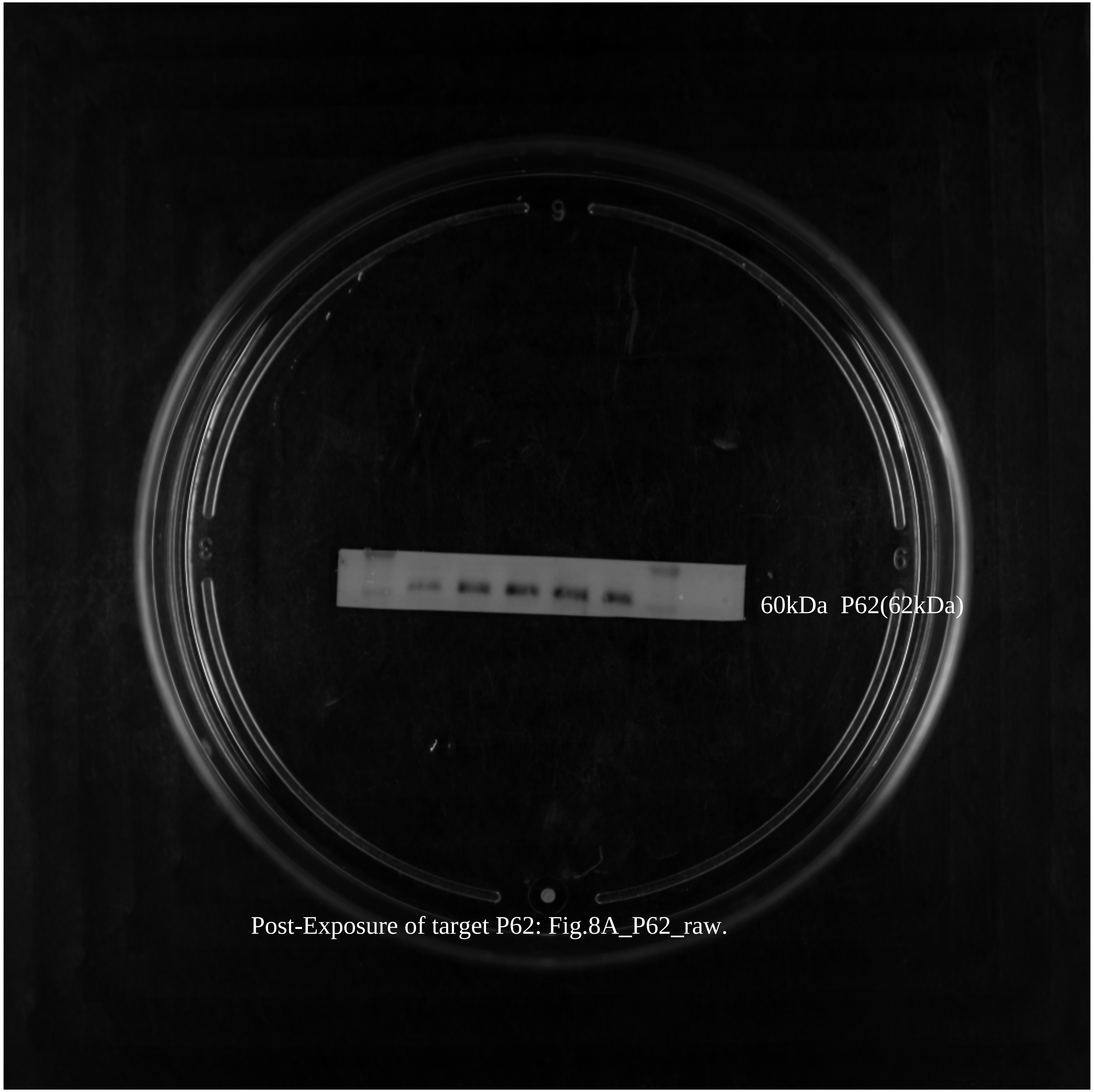

Con Mod NGR1 SSB2 NS

P62-1-β-Actin

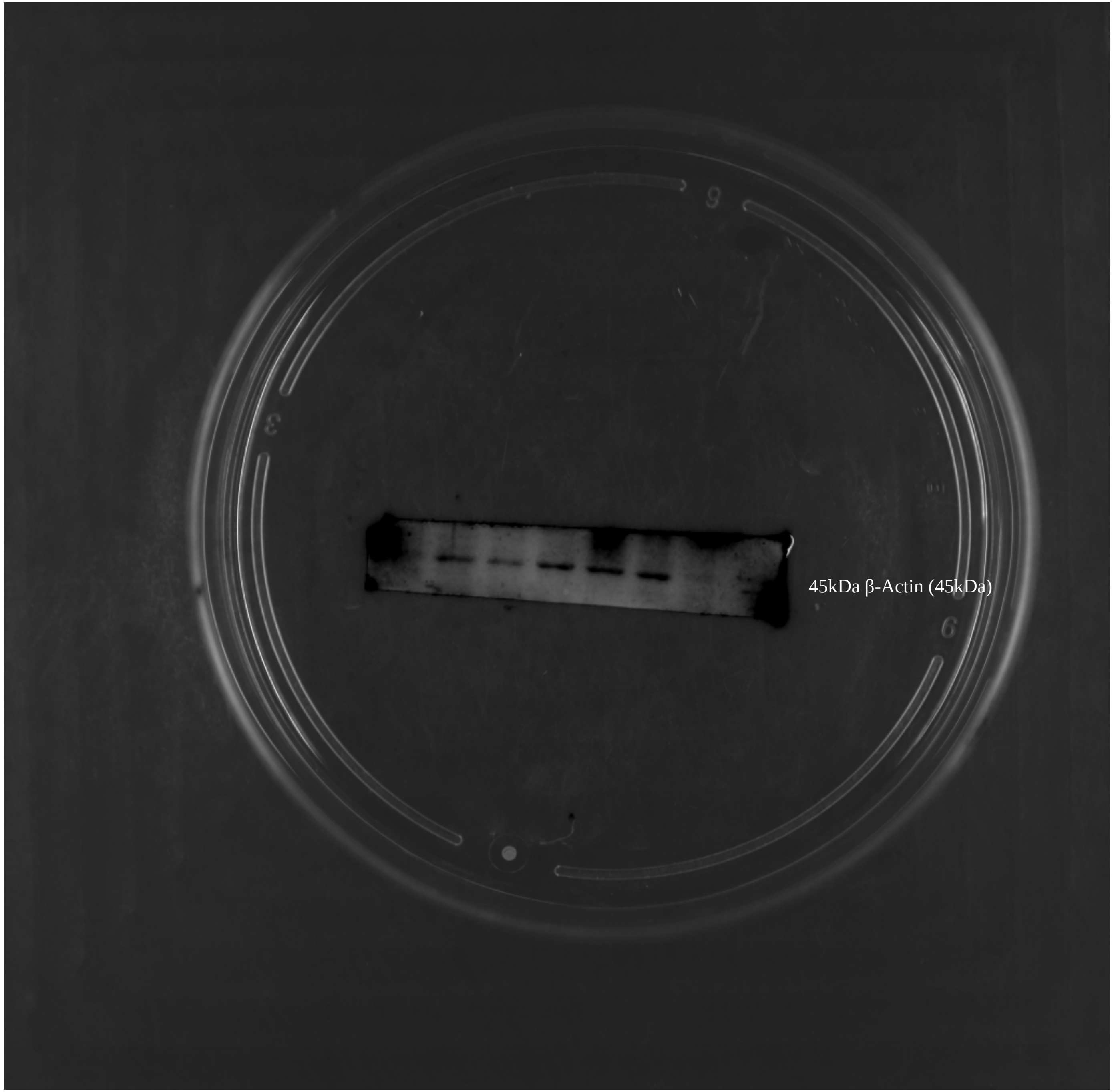

Con Mod NGR1 SSB2 NS

p-mTOR-1-full

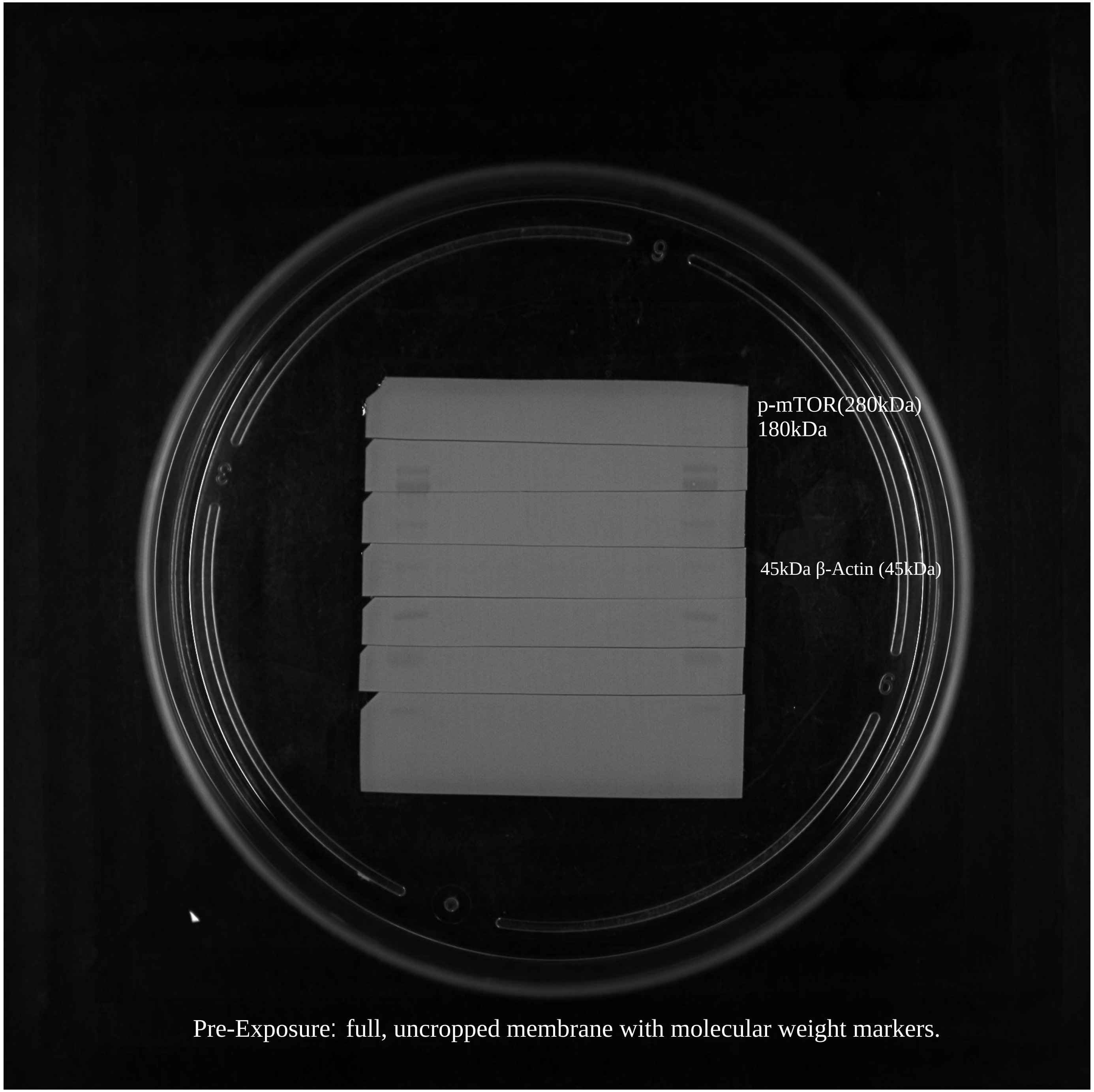

Con Mod NS NS+RA NS+3-MA

p-mTOR-1

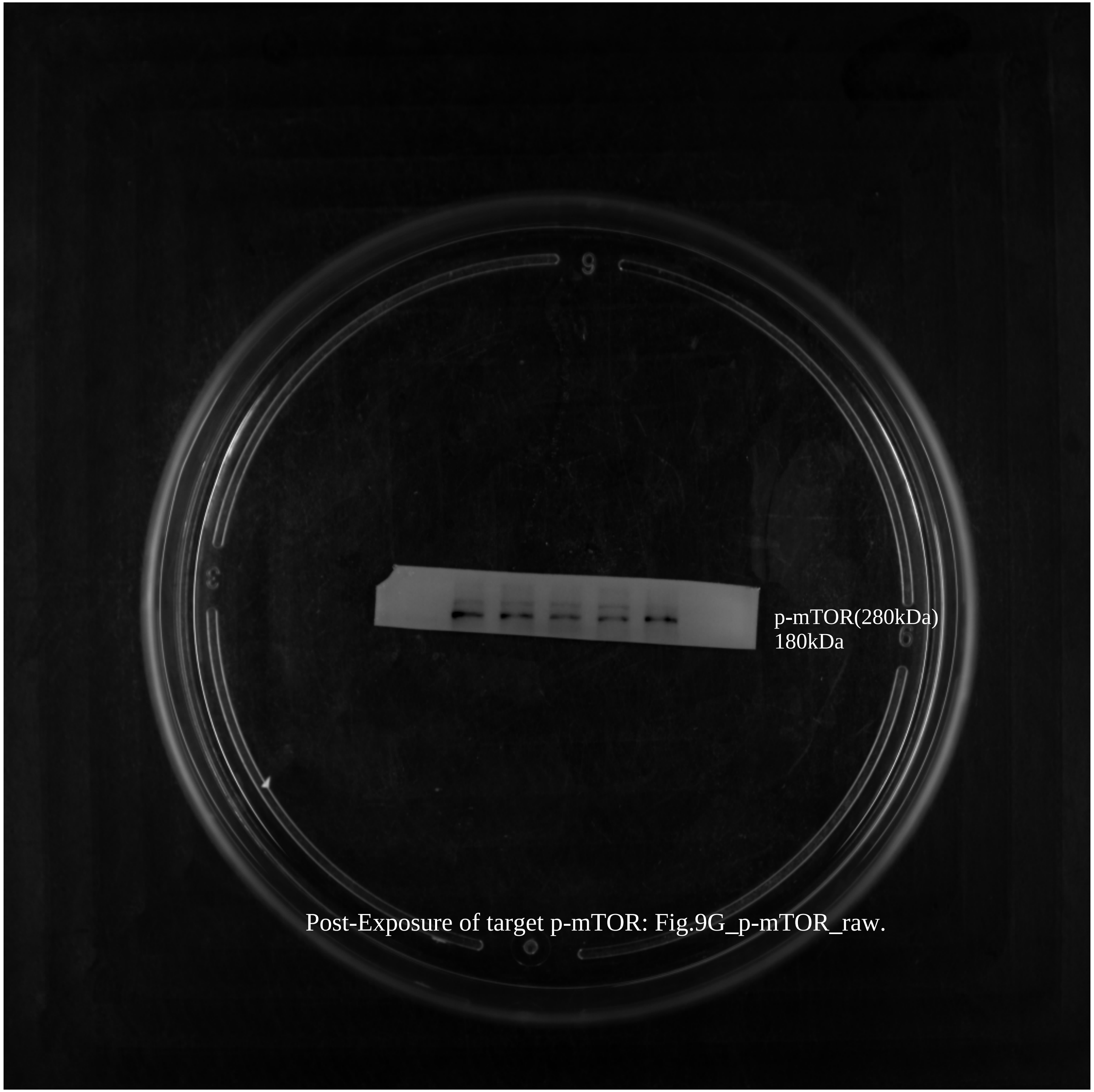

Con Mod NS NS+RA NS+3-MA

p-mTOR-1-β-Actin

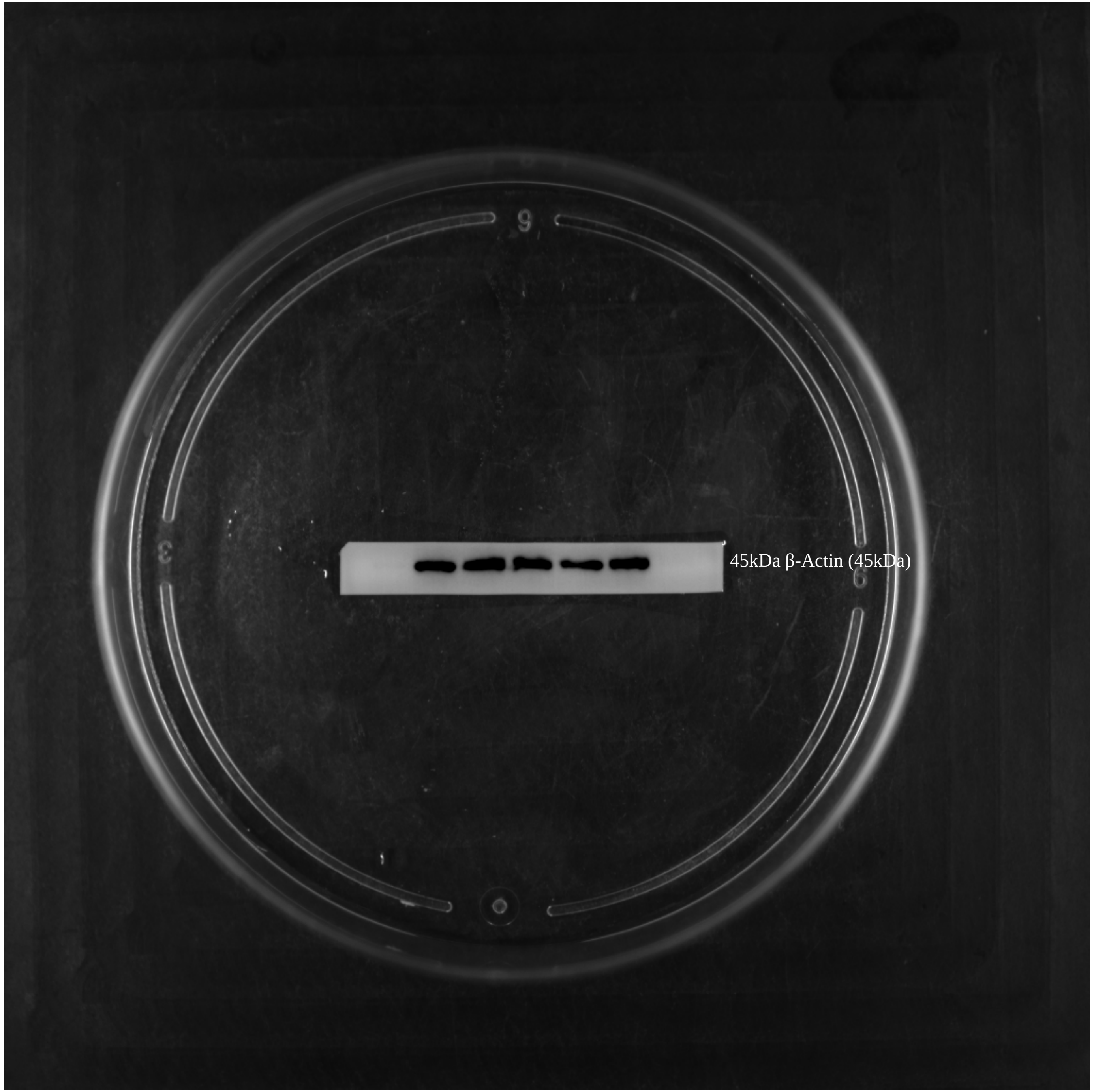

Con Mod NS NS+RA NS+3-MA

mTOR-1&2-full

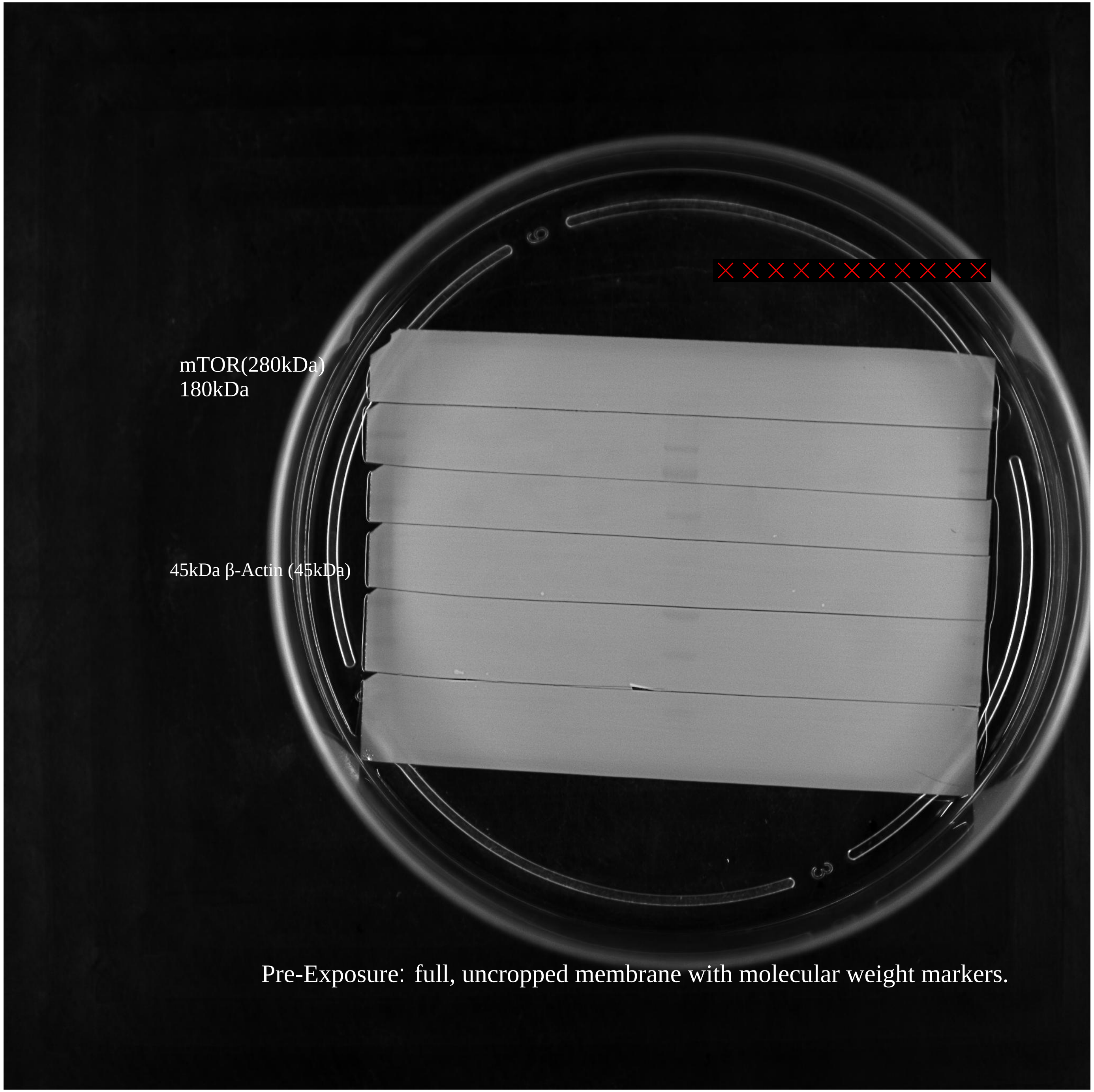

Con Mod NS NS+RA NS+3-MA Con Mod NS NS+RA NS+3-MA

mTOR-1&2

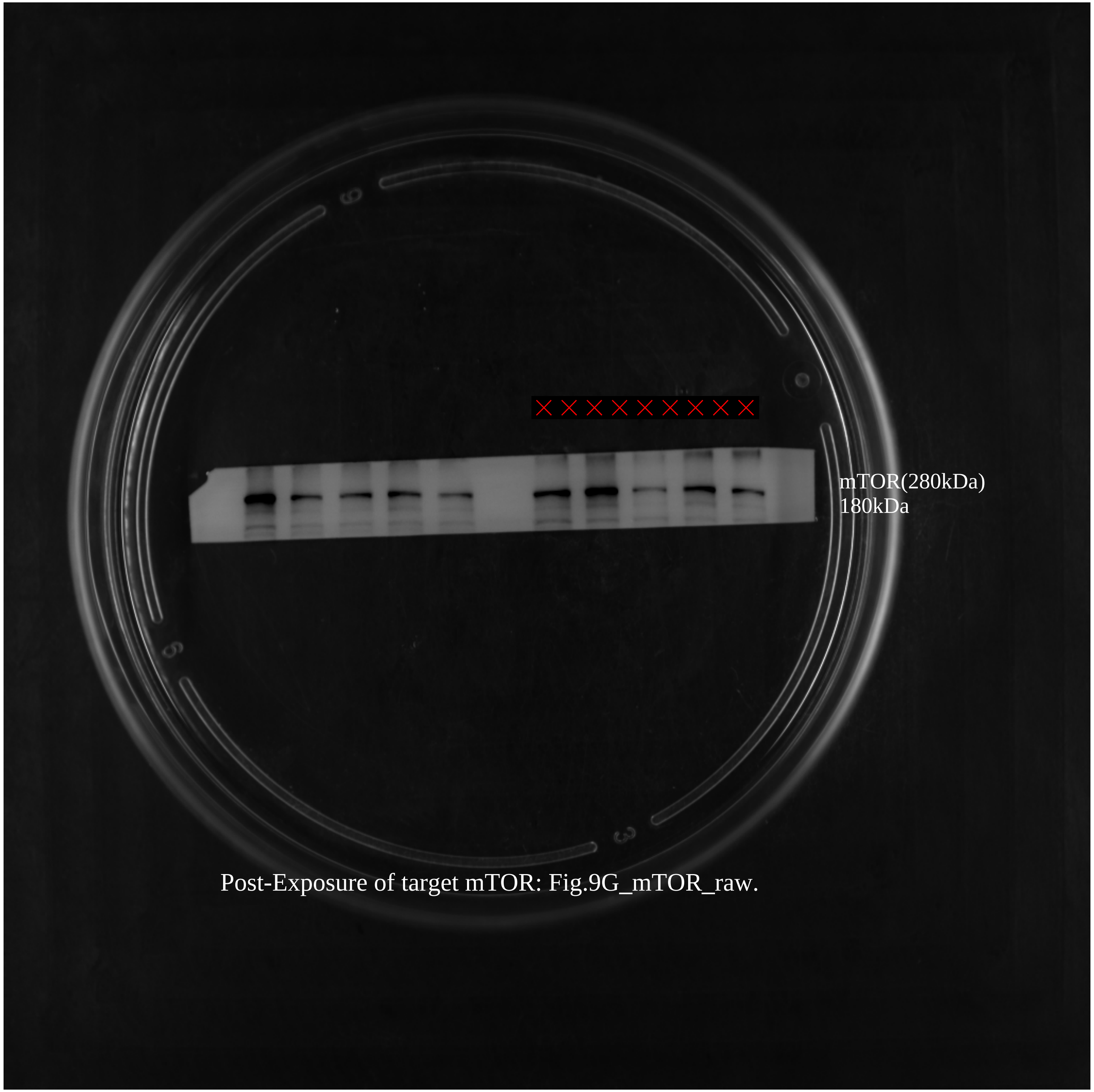

Con Mod NS NS+RA NS+3-MA Con Mod NS NS+RA NS+3-MA

mTOR-1&2-β-Actin

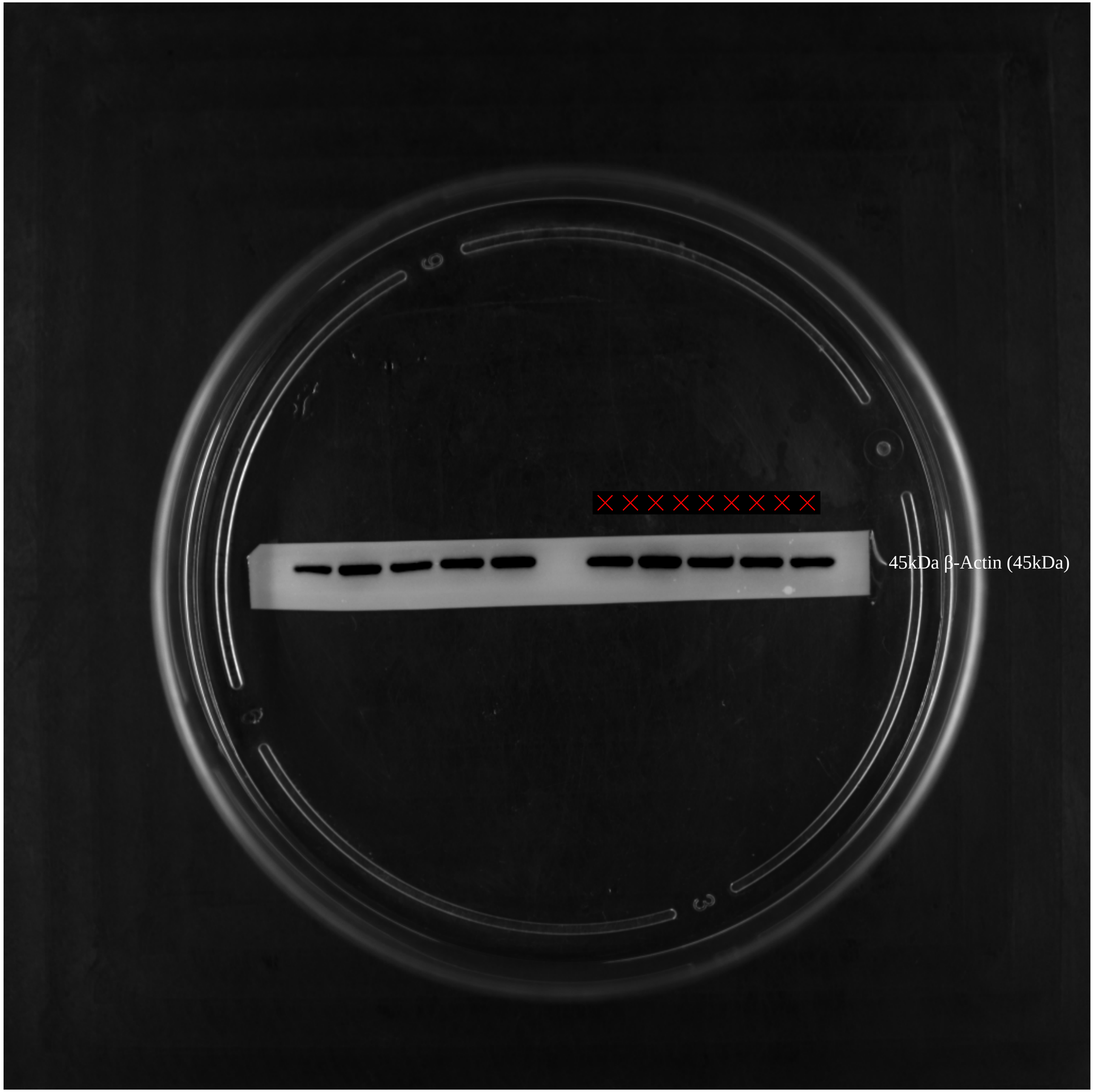

Con Mod NS NS+RA NS+3-MA Con Mod NS NS+RA NS+3-MA

NLRP3-1-full

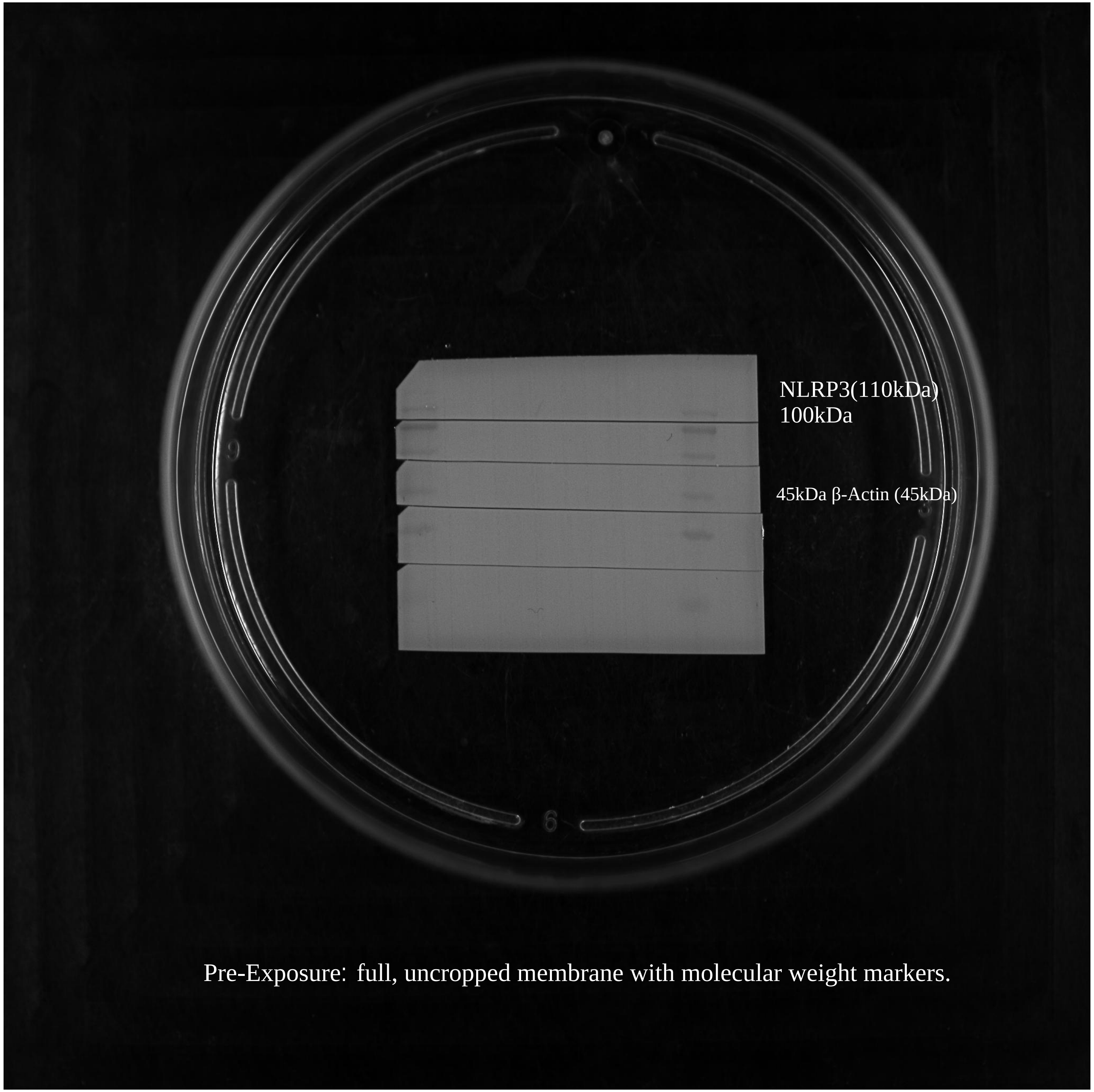

Con Mod NS NS+RA NS+3-MA

NLRP3-1

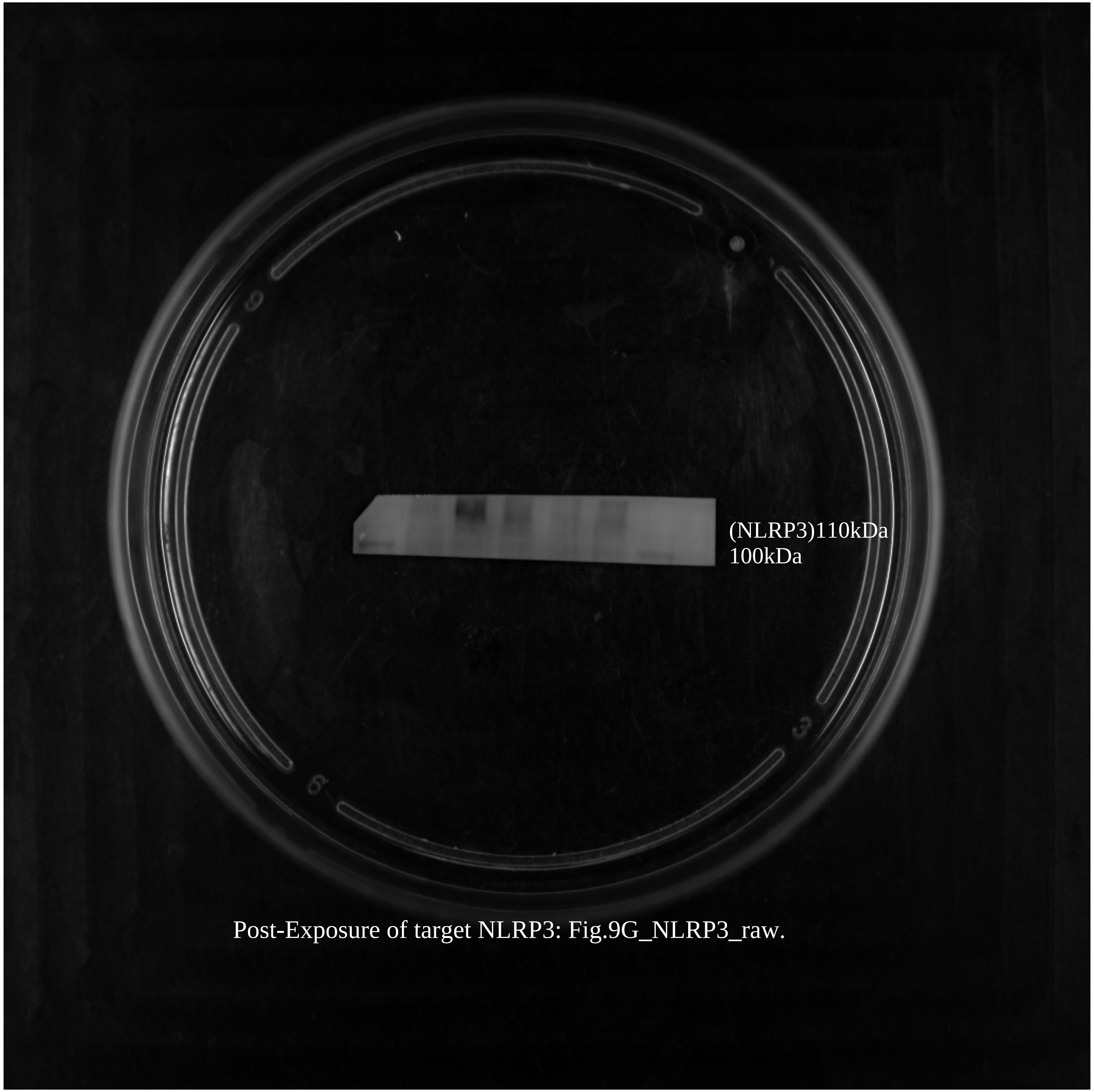

Con Mod NS NS+RA NS+3-MA

NLRP3-1-β-Actin

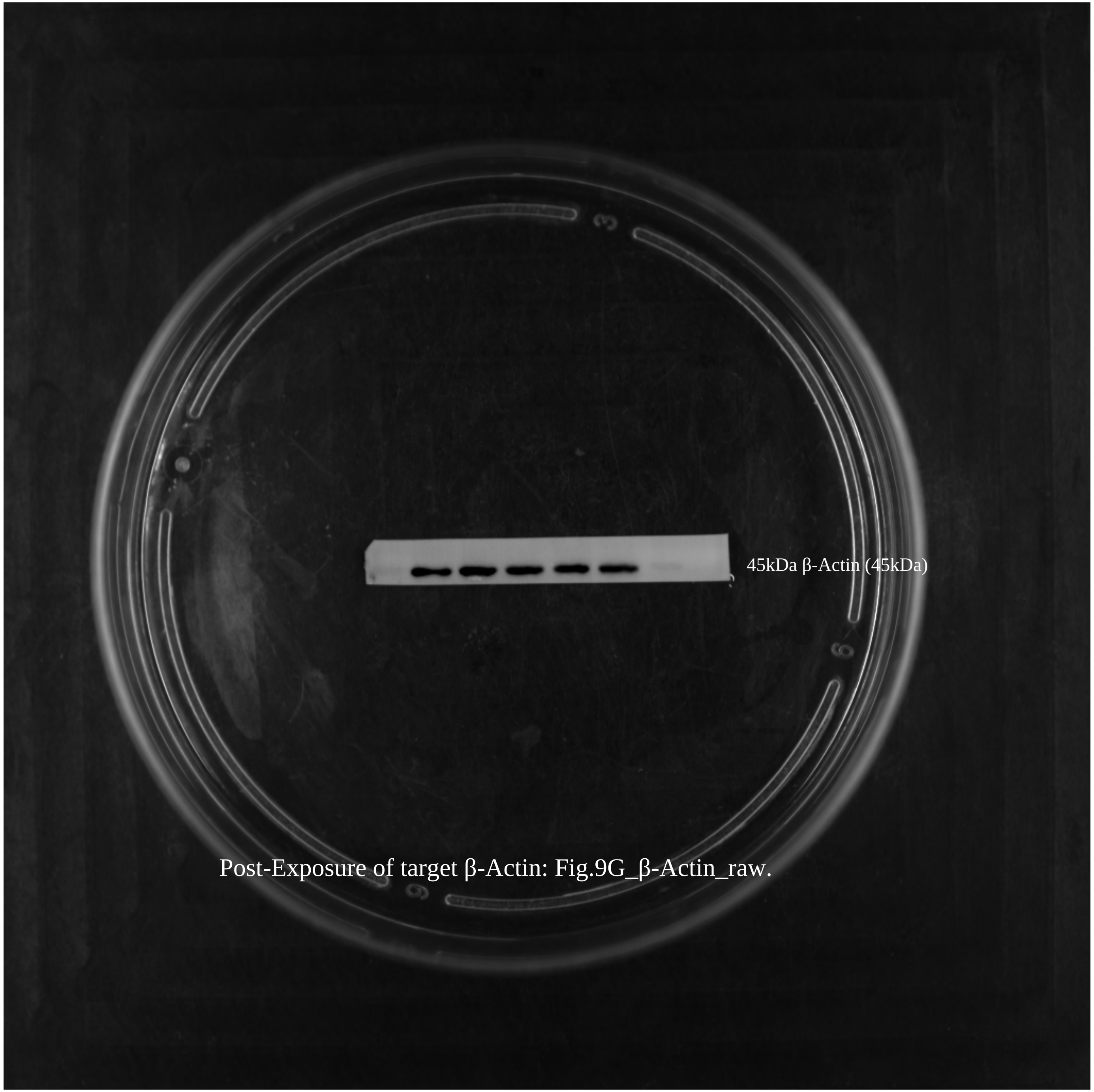

Con Mod NS NS+RA NS+3-MA

BAX-1-full

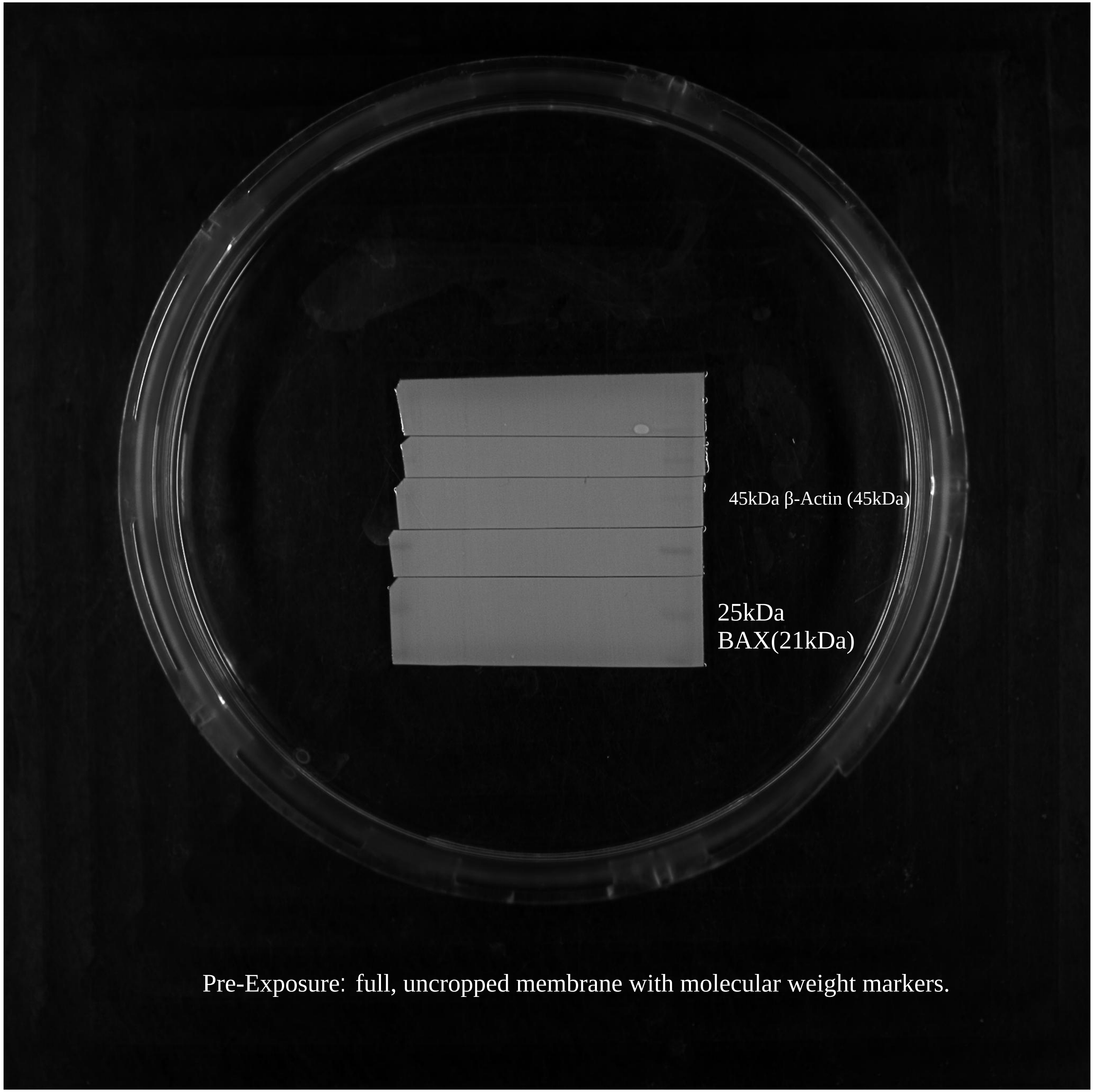

Con Mod NS NS+RA NS+3-MA

BAX-1

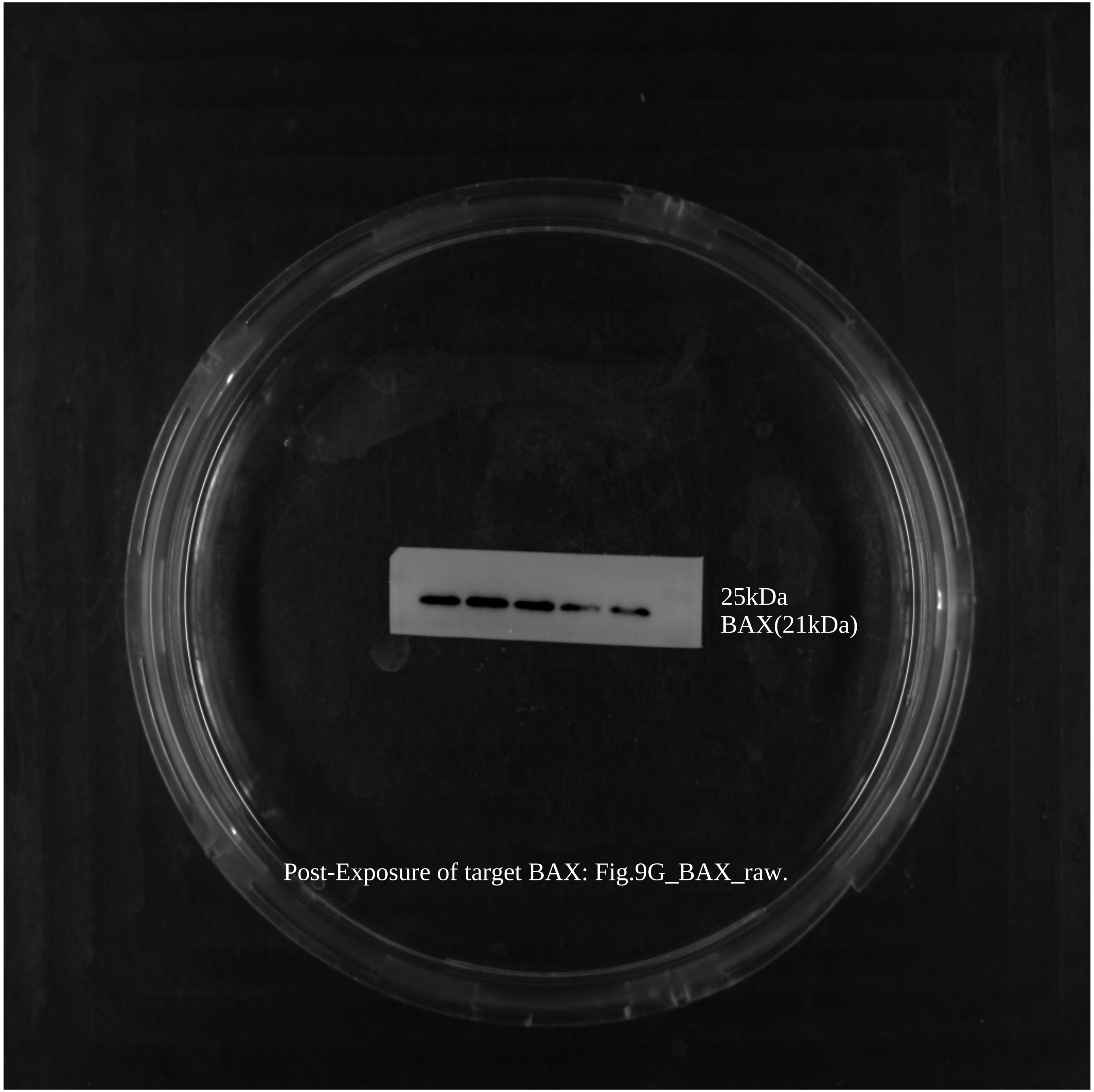

Con Mod NS NS+RA NS+3-MA

BAX-1-β-Actin

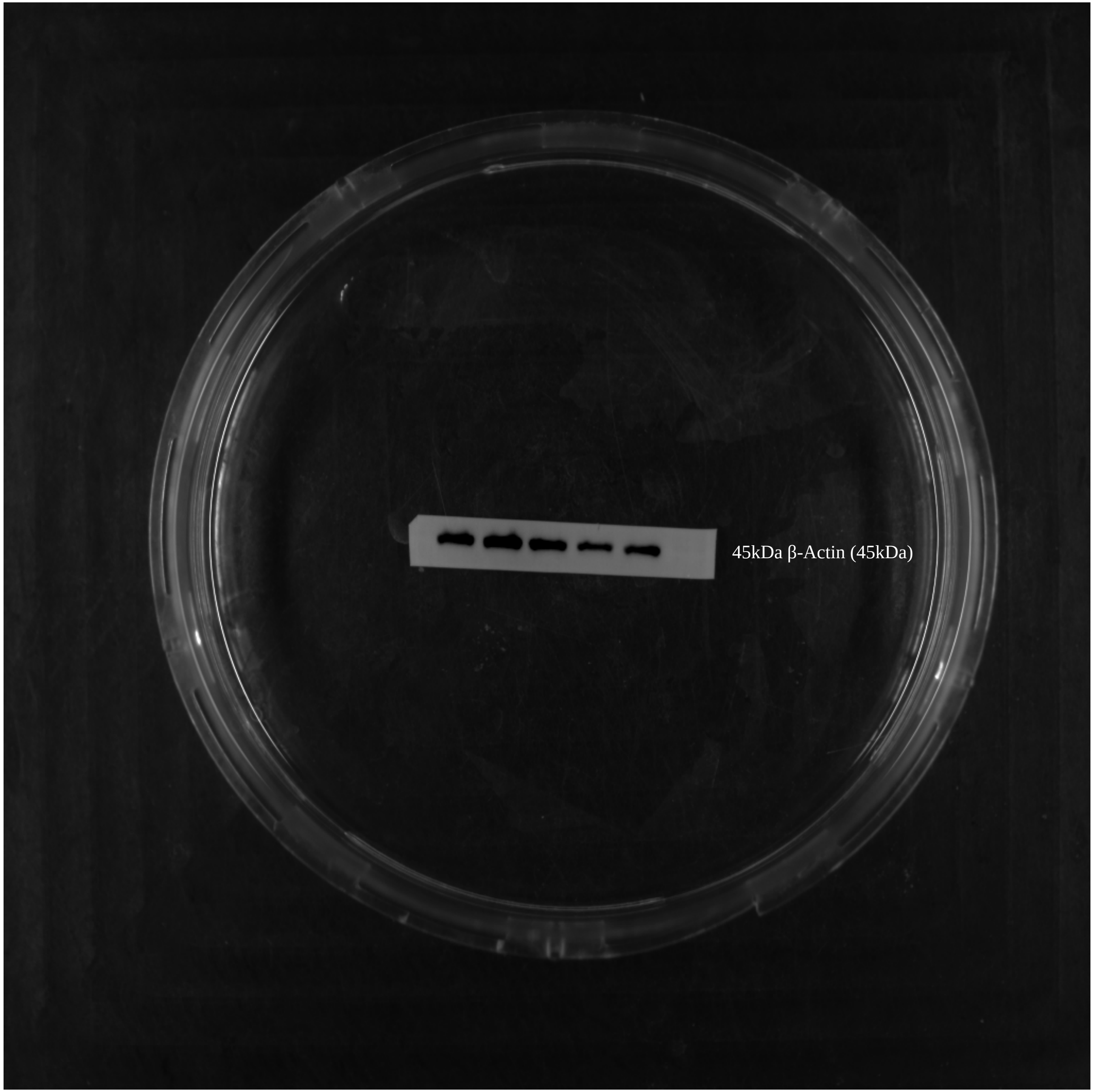

Con Mod NS NS+RA NS+3-MA

BCL2-1-full

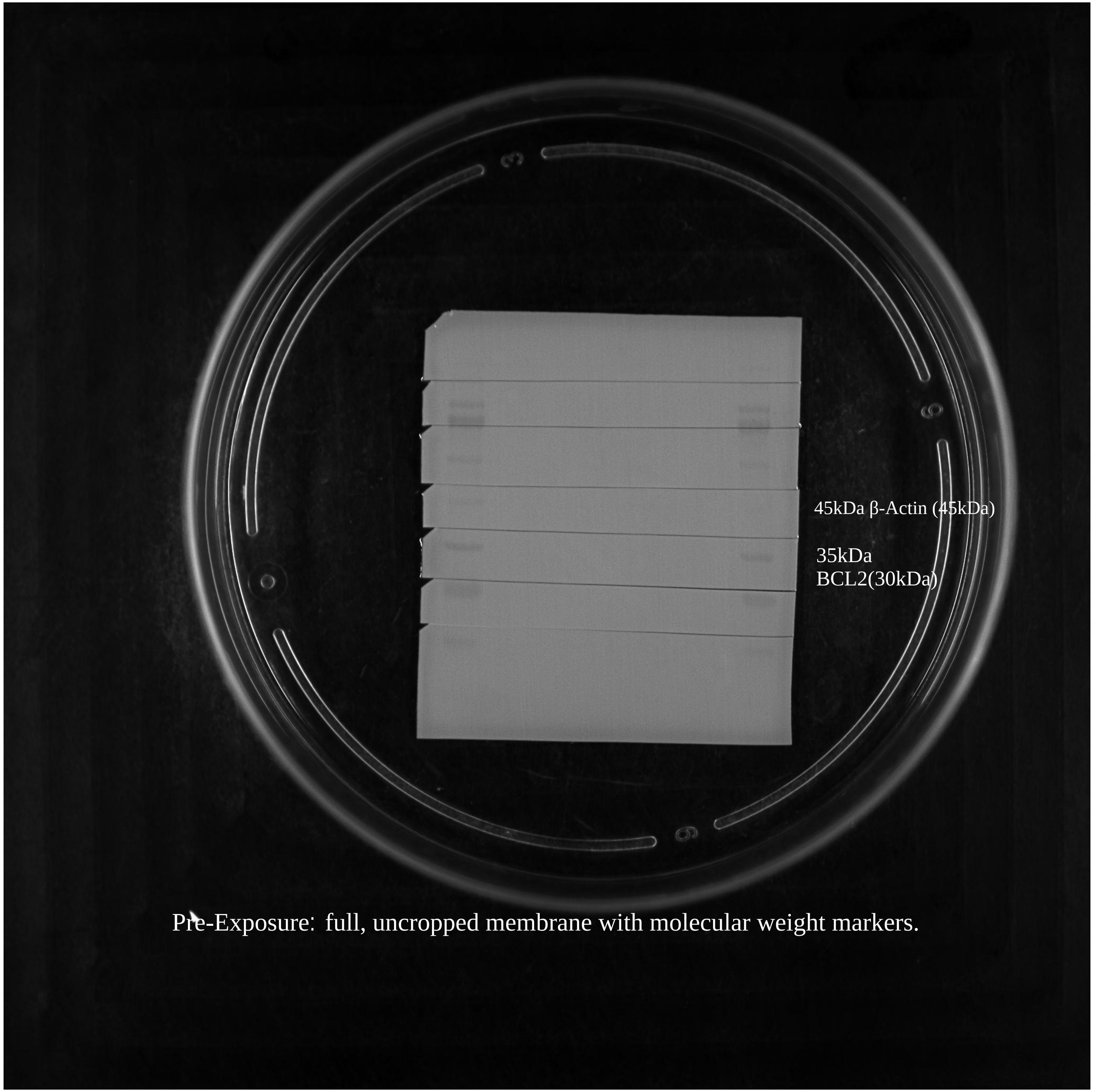

Con Mod NS NS+RA NS+3-MA

BCL2-1

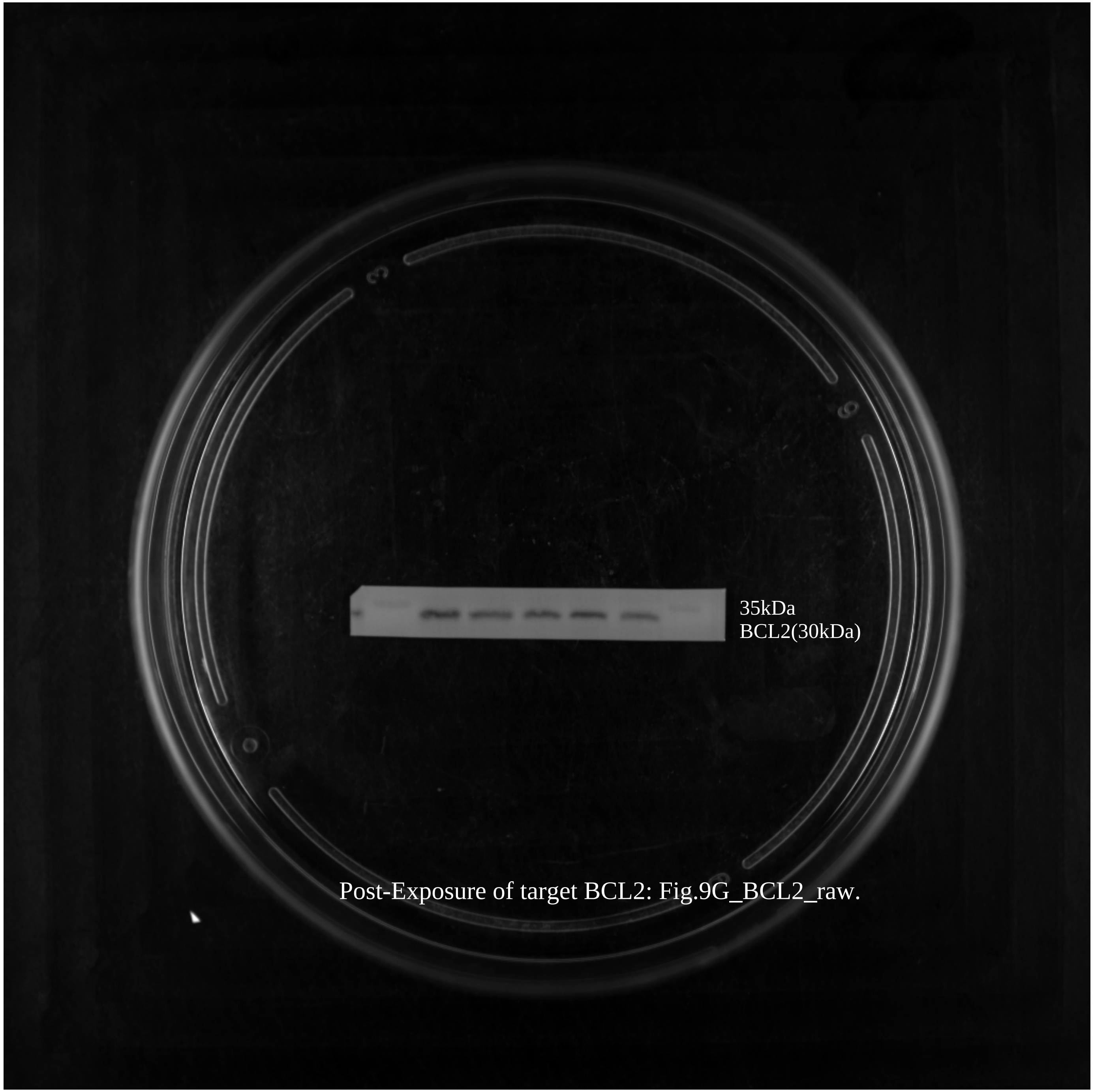

Con Mod NS NS+RA NS+3-MA

BCL2-1-β-Actin

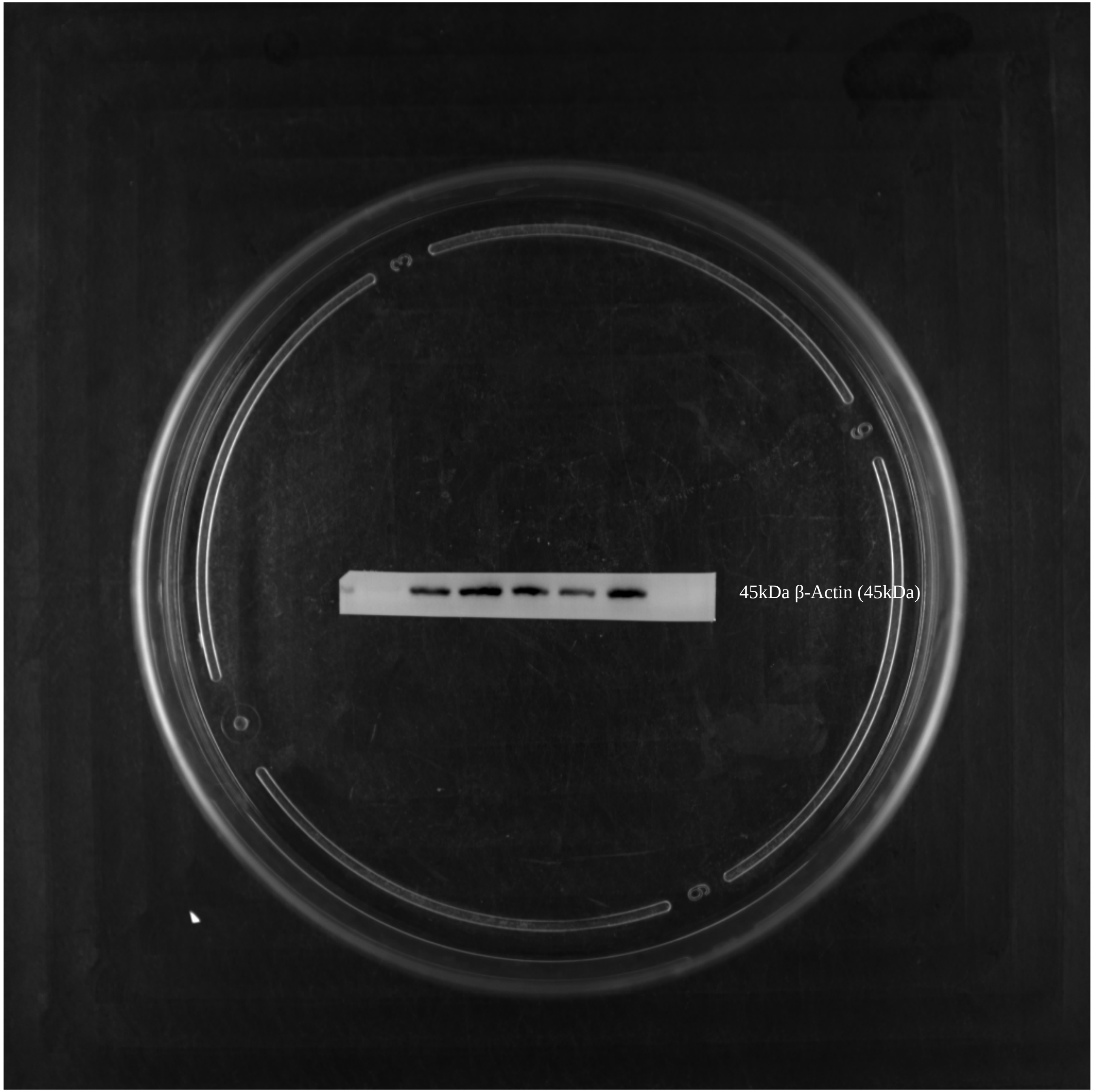

Con Mod NS NS+RA NS+3-MA

Caspase-3-1&2-full

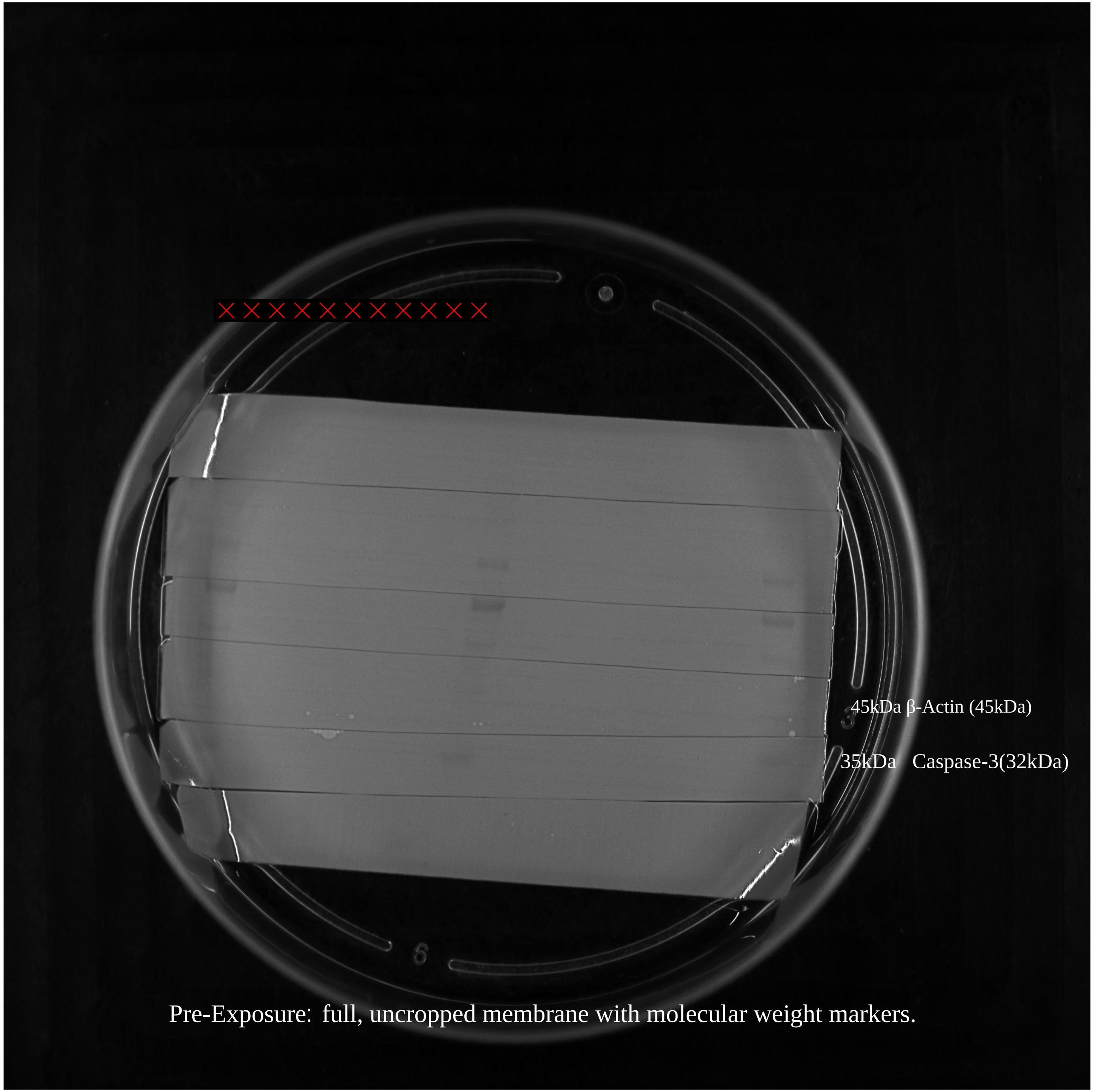

Con Mod NS NS+RA NS+3-MA Con Mod NS NS+RA NS+3-MA

Caspase-3-1&2

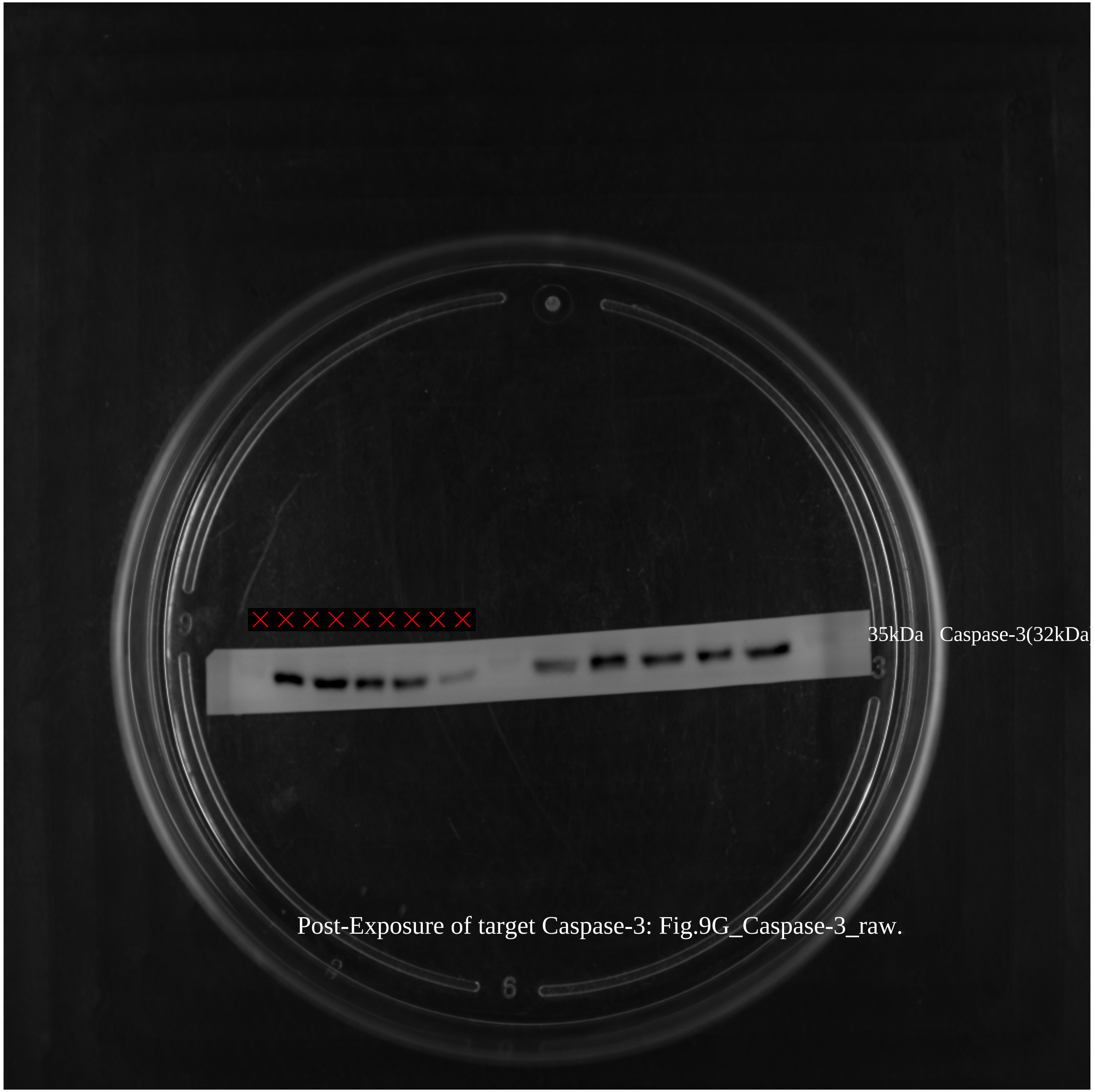

Con Mod NS NS+RA NS+3-MA   Con Mod NS NS+RA NS+3-MA

Caspase-3-1&2-β-Actin

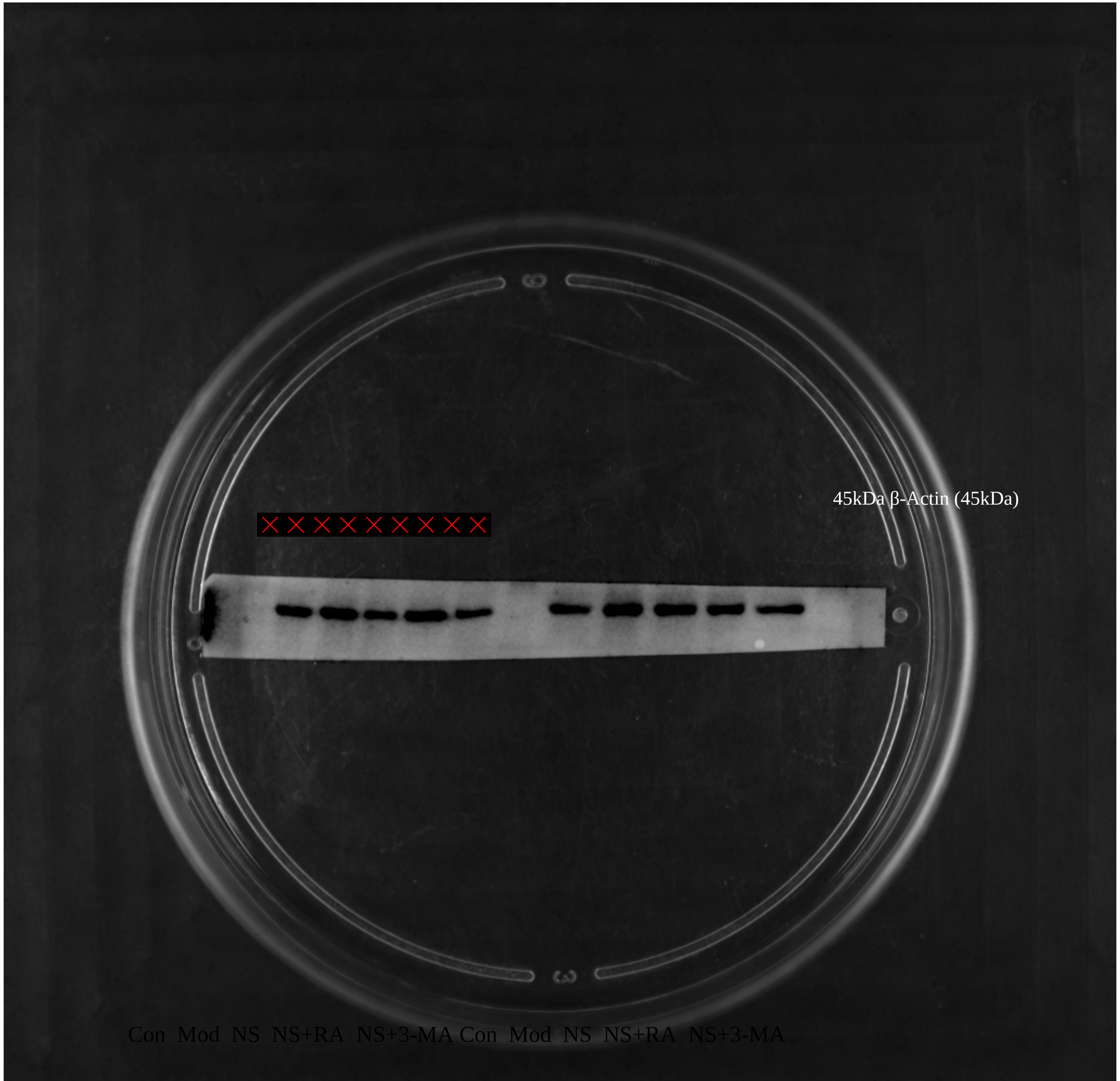

P62-1&2-full

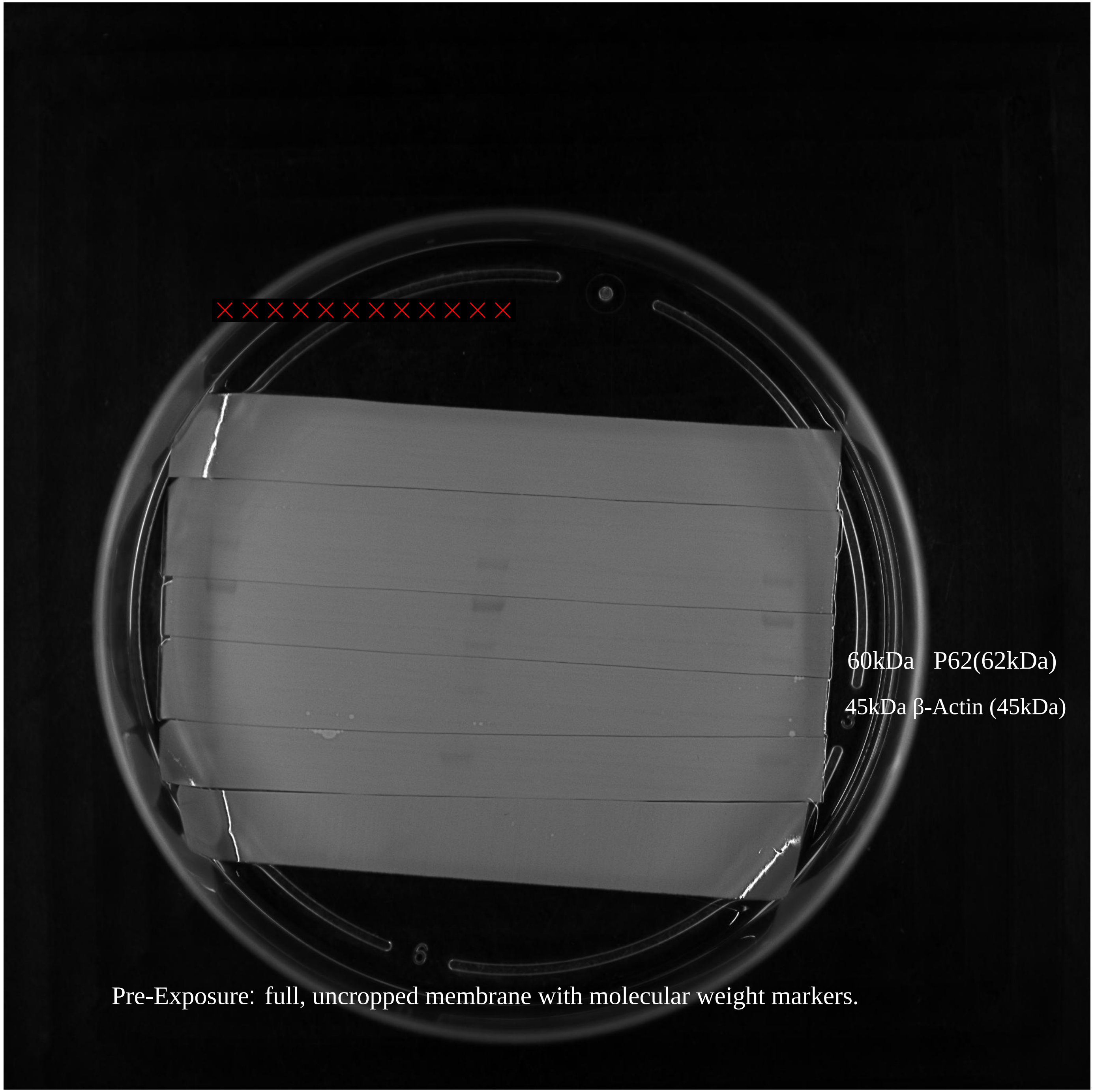

Con Mod NS NS+RA NS+3-MA Con Mod NS NS+RA NS+3-MA

P62-1&2

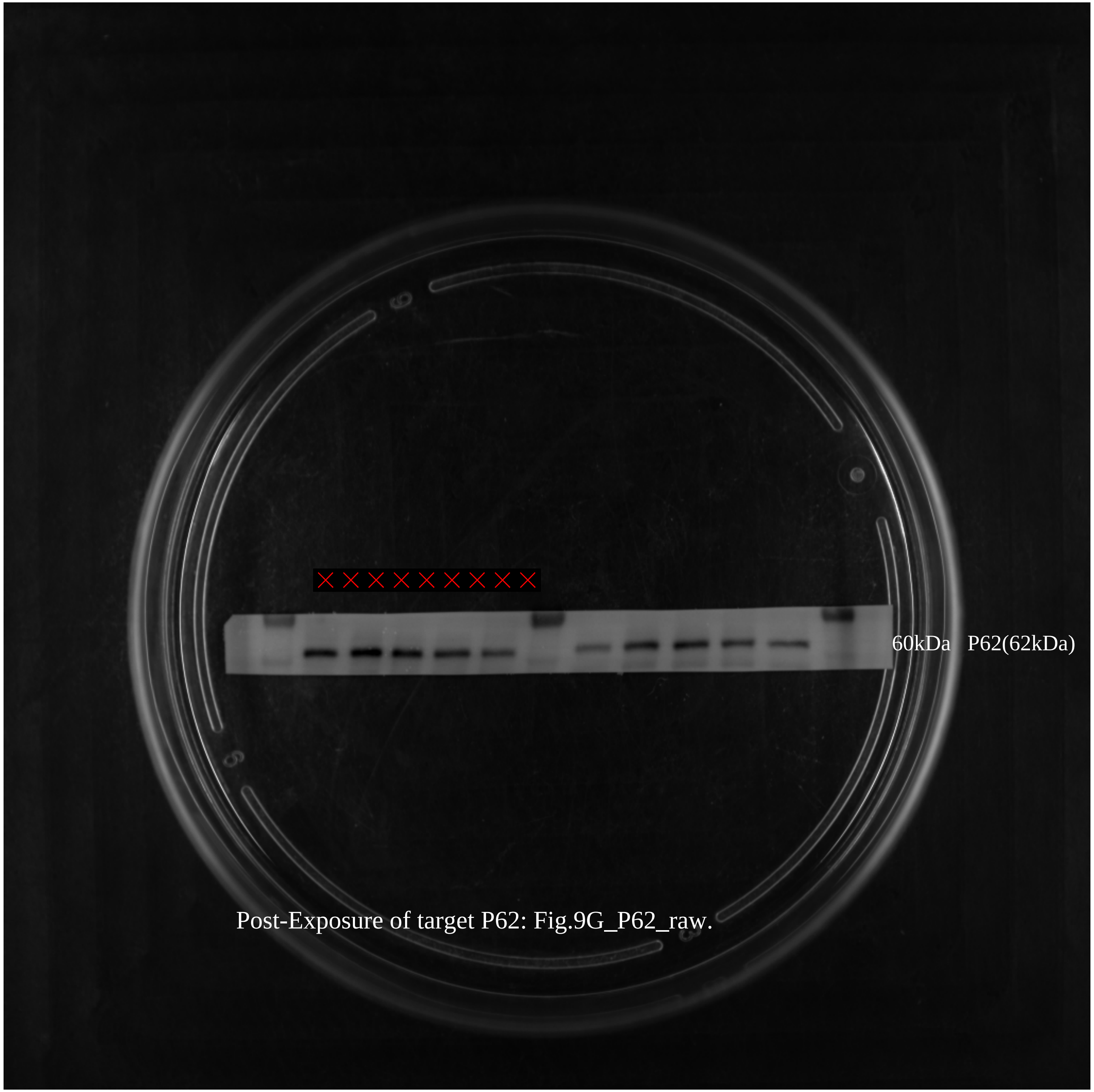

Con Mod NS NS+RA NS+3-MA   Con Mod NS NS+RA NS+3-MA

P62-1&2-β-Actin

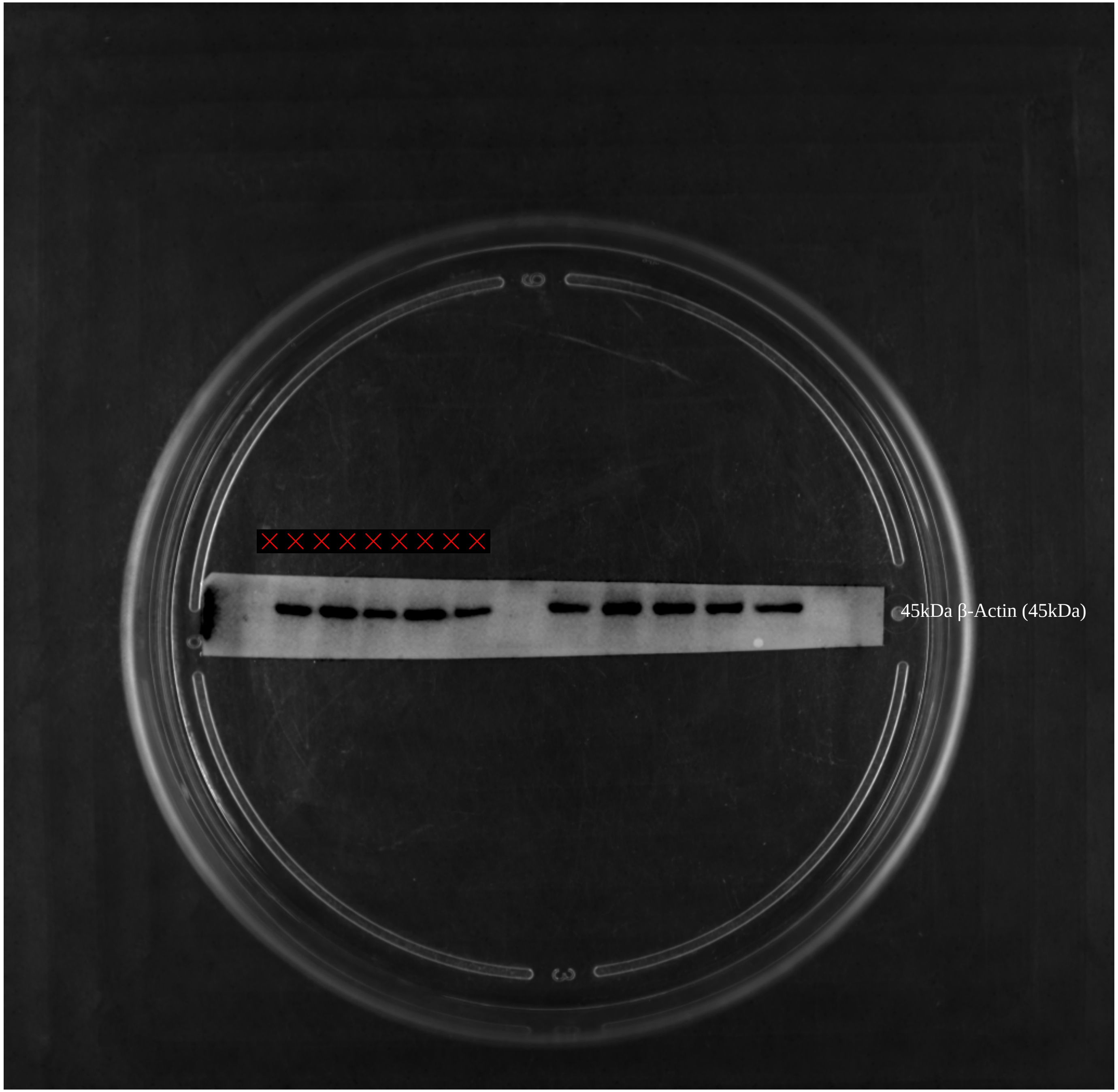

Con Mod NS NS+RA NS+3-MA   Con Mod NS NS+RA NS+3-MA

Beclin1-1-full

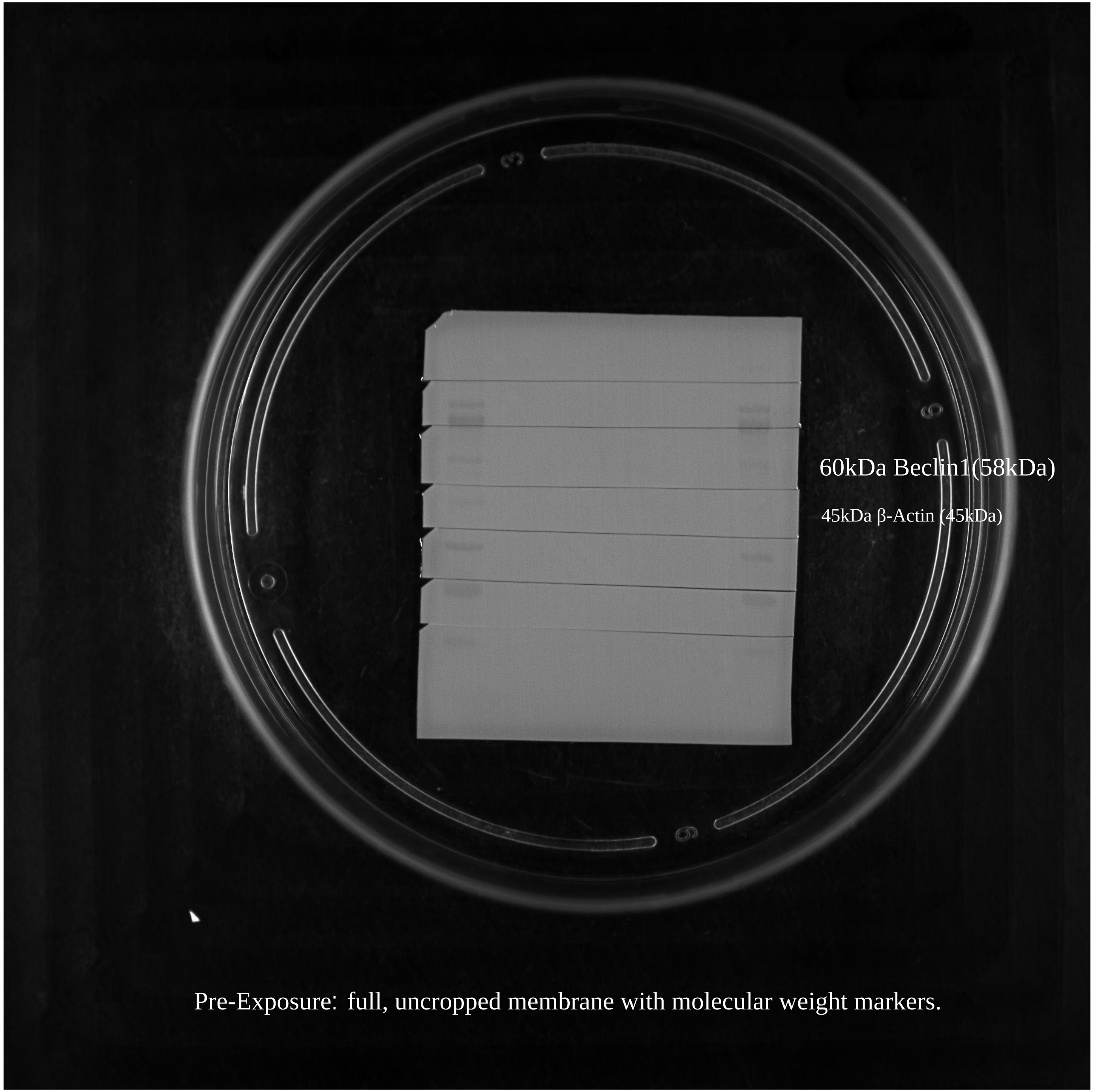

Con Mod NS NS+RA NS+3-MA

Beclin1-1

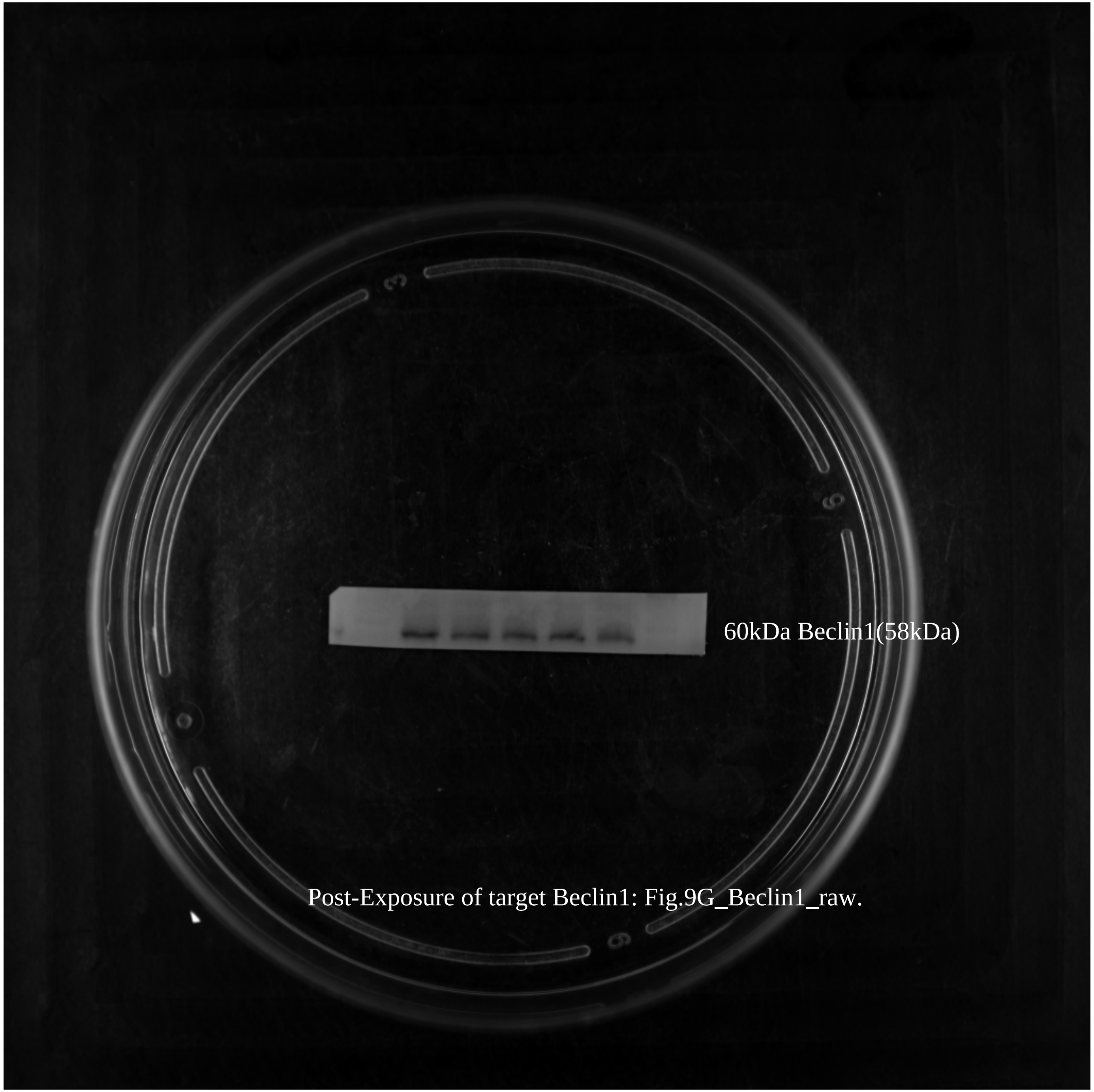

Con Mod NS NS+RA NS+3-MA

Beclin1-1-β-Actin

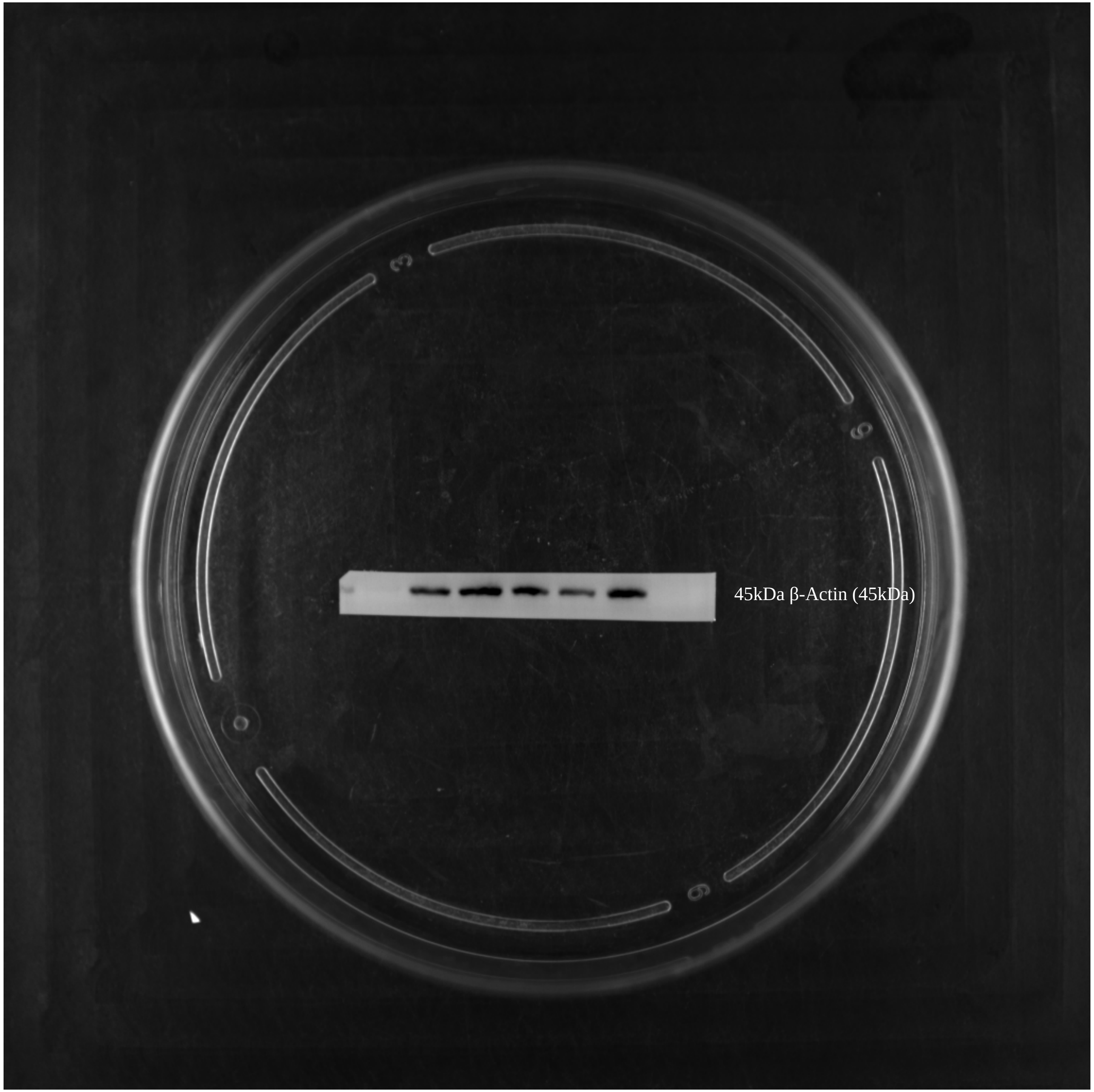

Con Mod NS NS+RA NS+3-MA

LC3-1-β-Actin

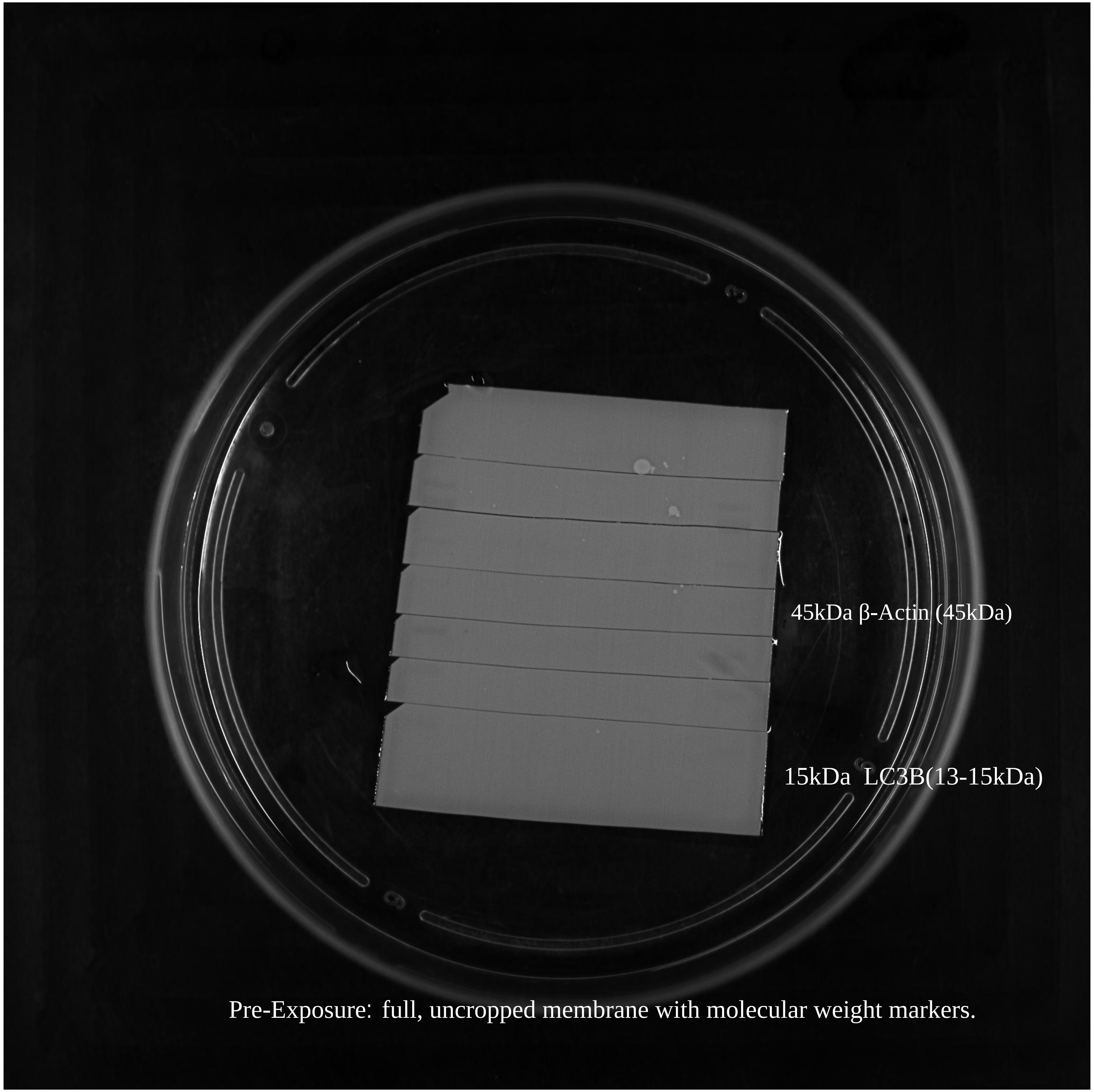

Con Mod NS NS+RA NS+3-MA

LC3-1

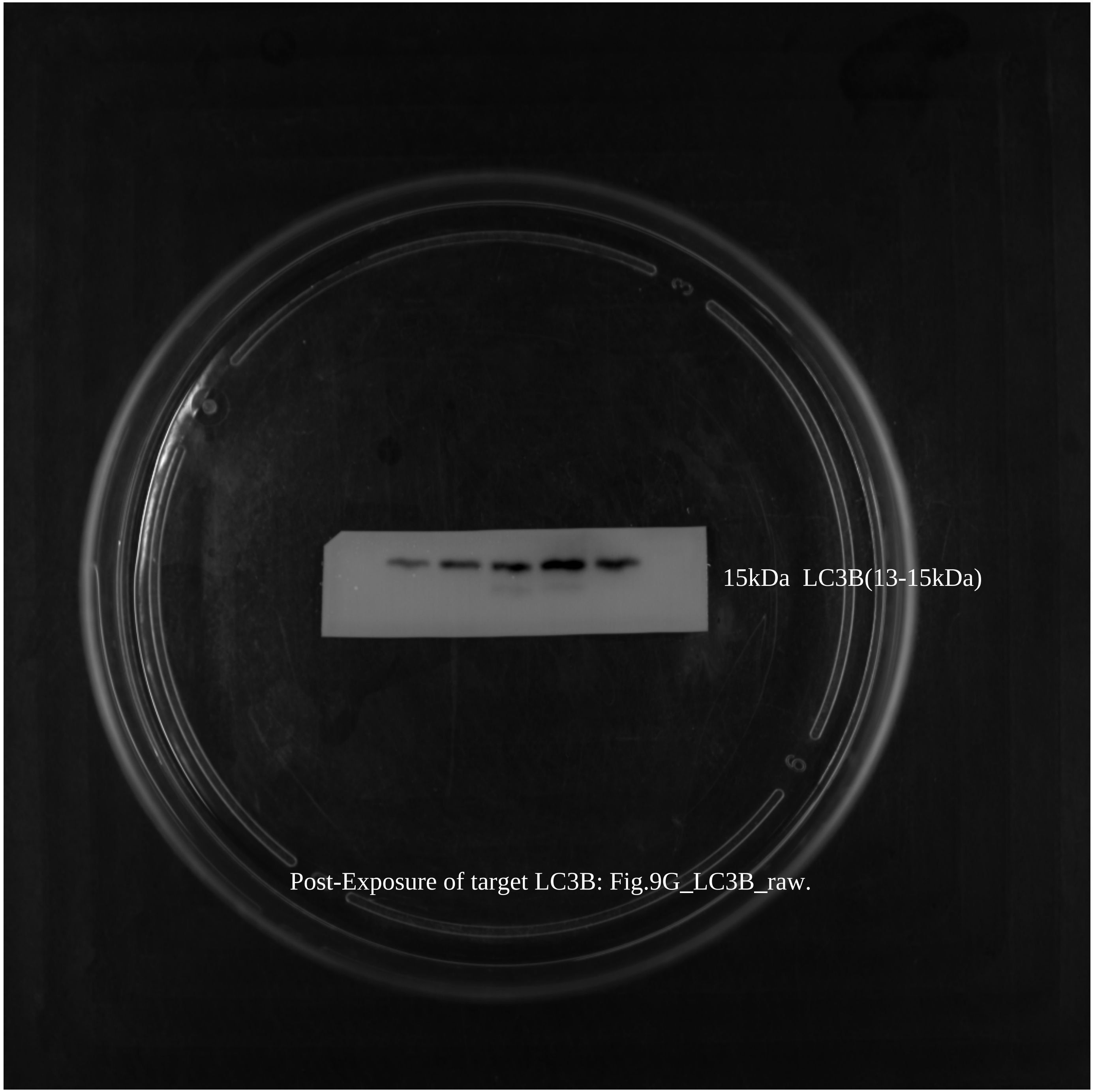

Con Mod NS NS+RA NS+3-MA

LC3-1-β-Actin

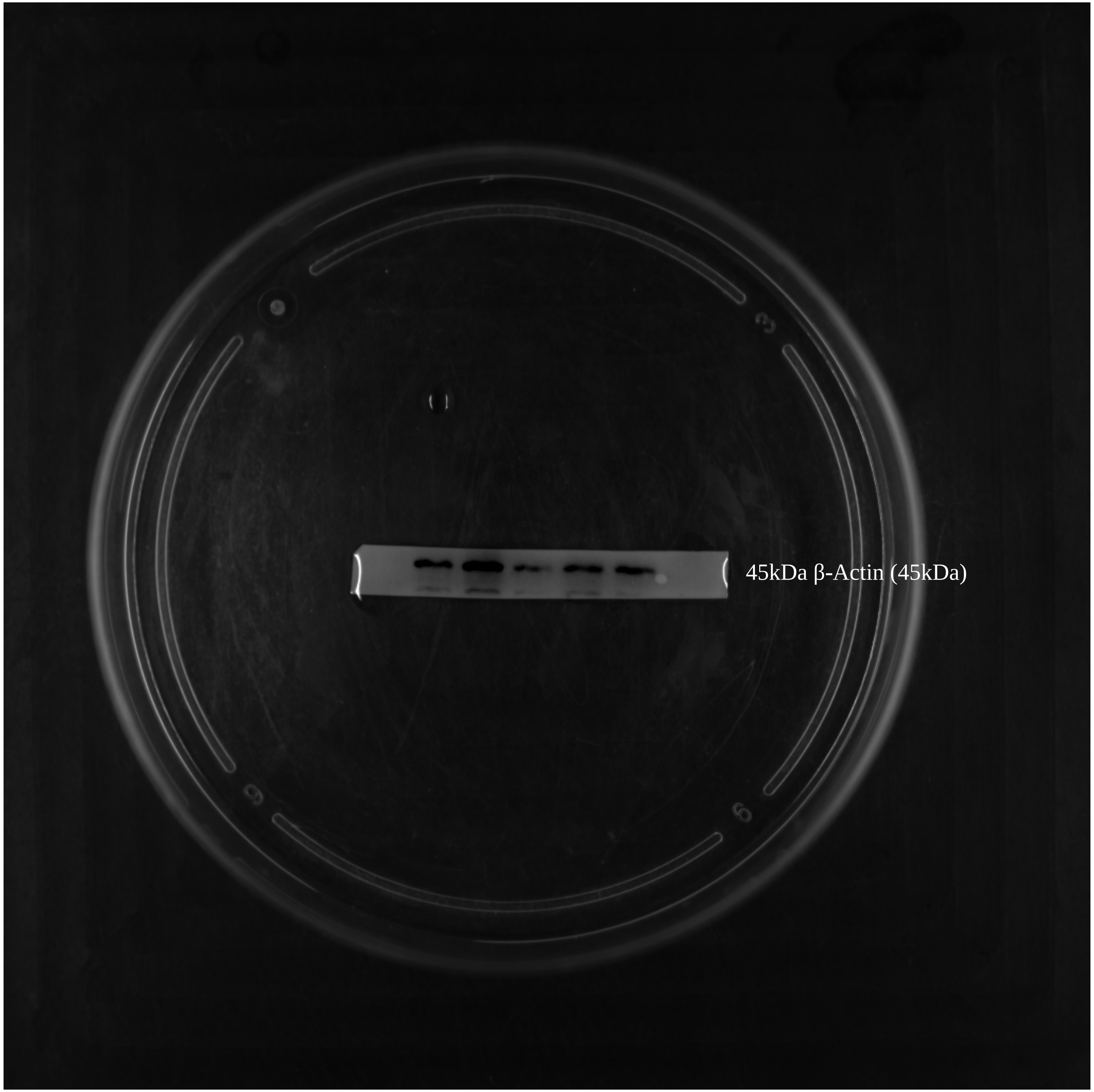

Con Mod NS NS+RA NS+3-MA

NLRP3-2-full

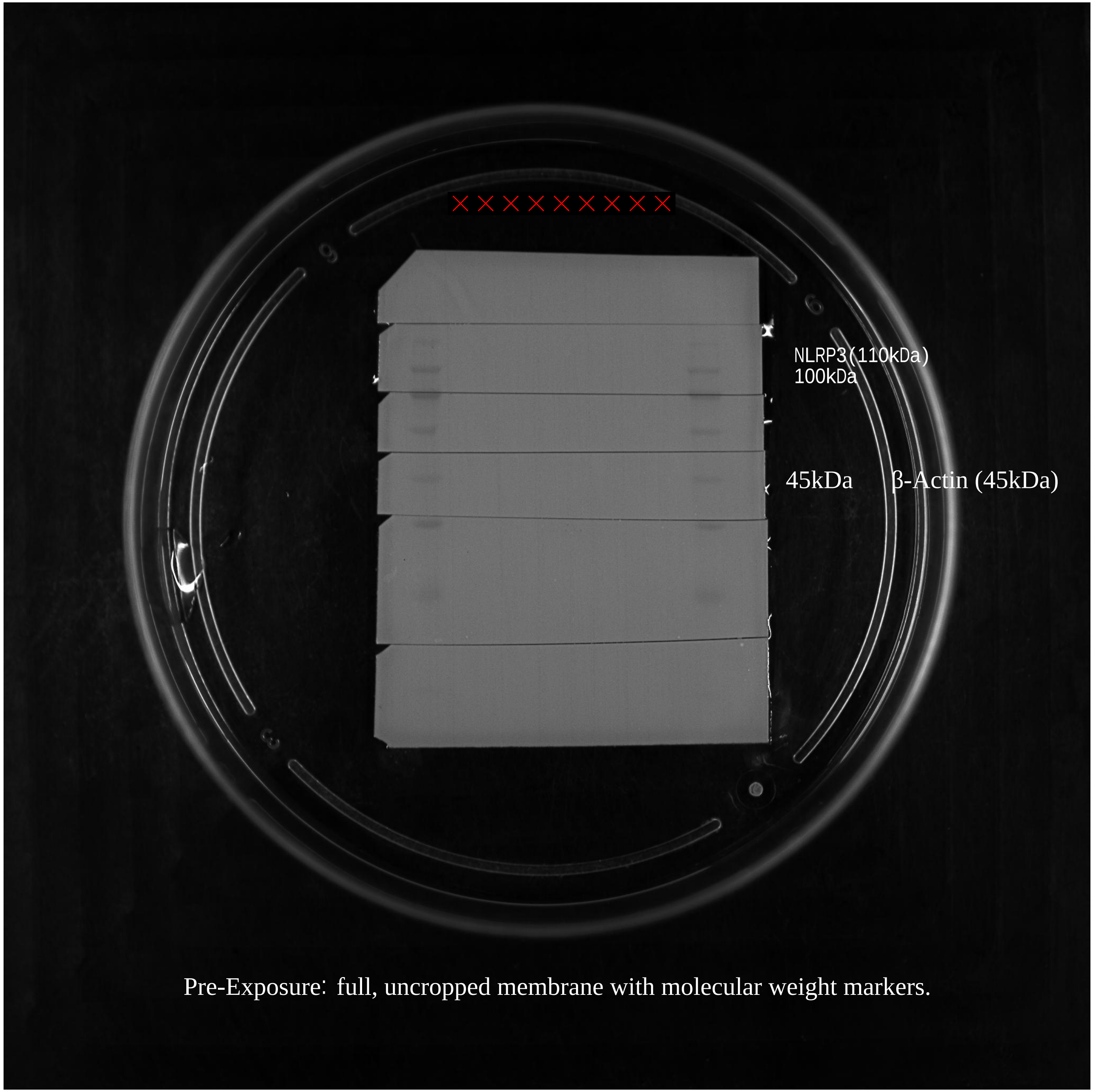

Con Mod NGR1 SSB2 NS

NLRP3-2

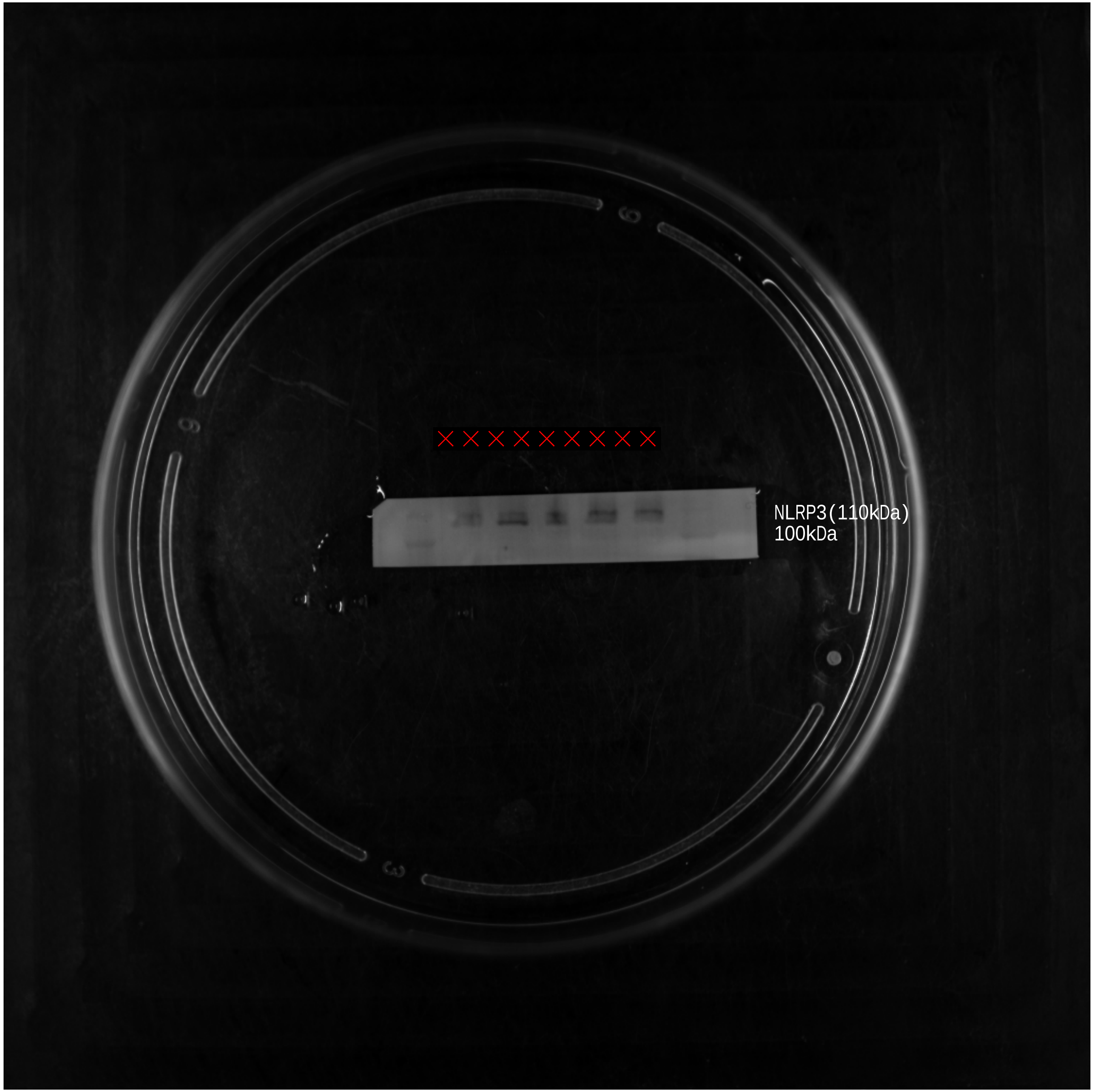

Con Mod NGR1 SSB2 NS

NLRP3-2-β-Actin

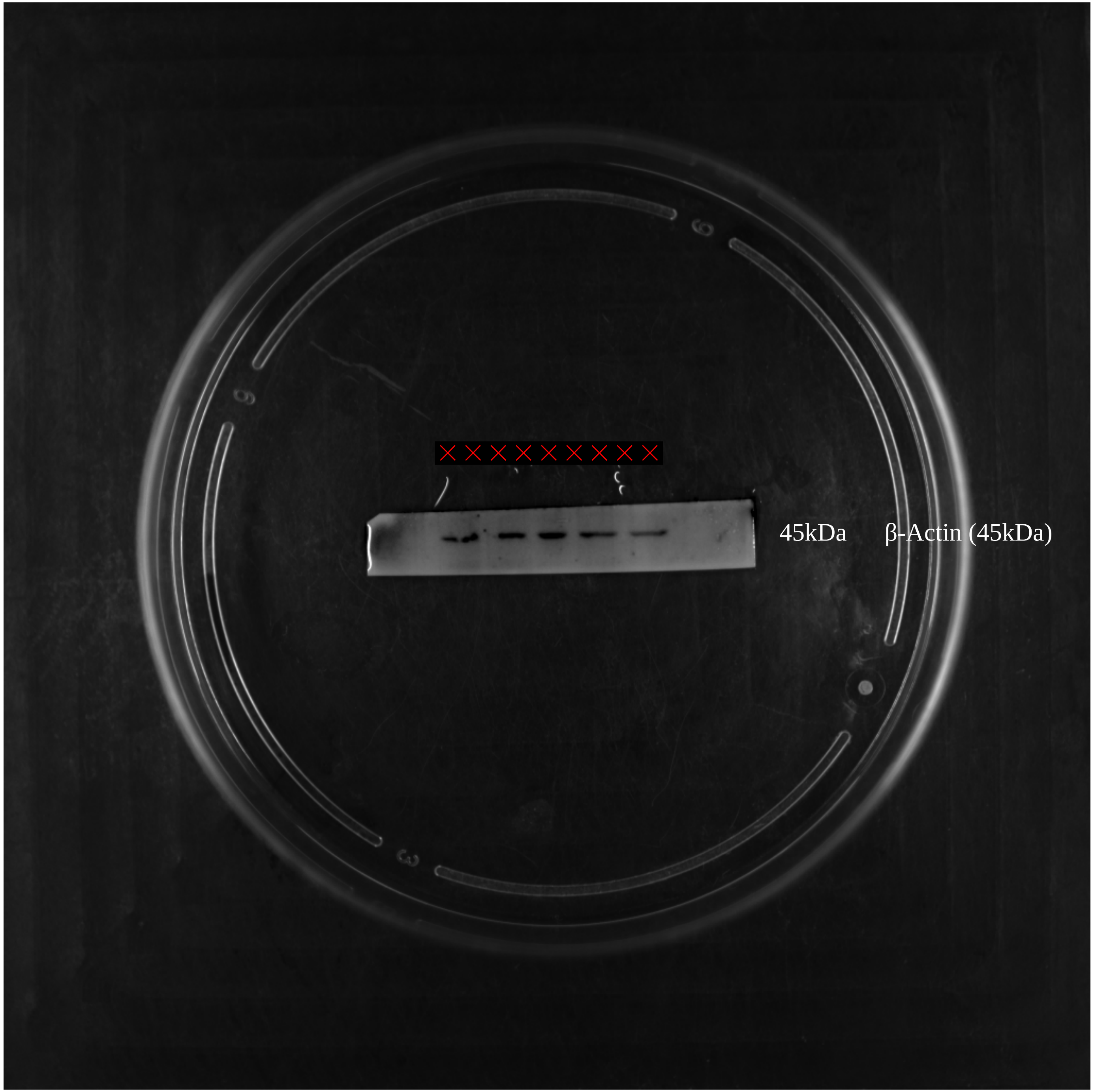

Con Mod NGR1 SSB2 NS

NLRP3-3-full

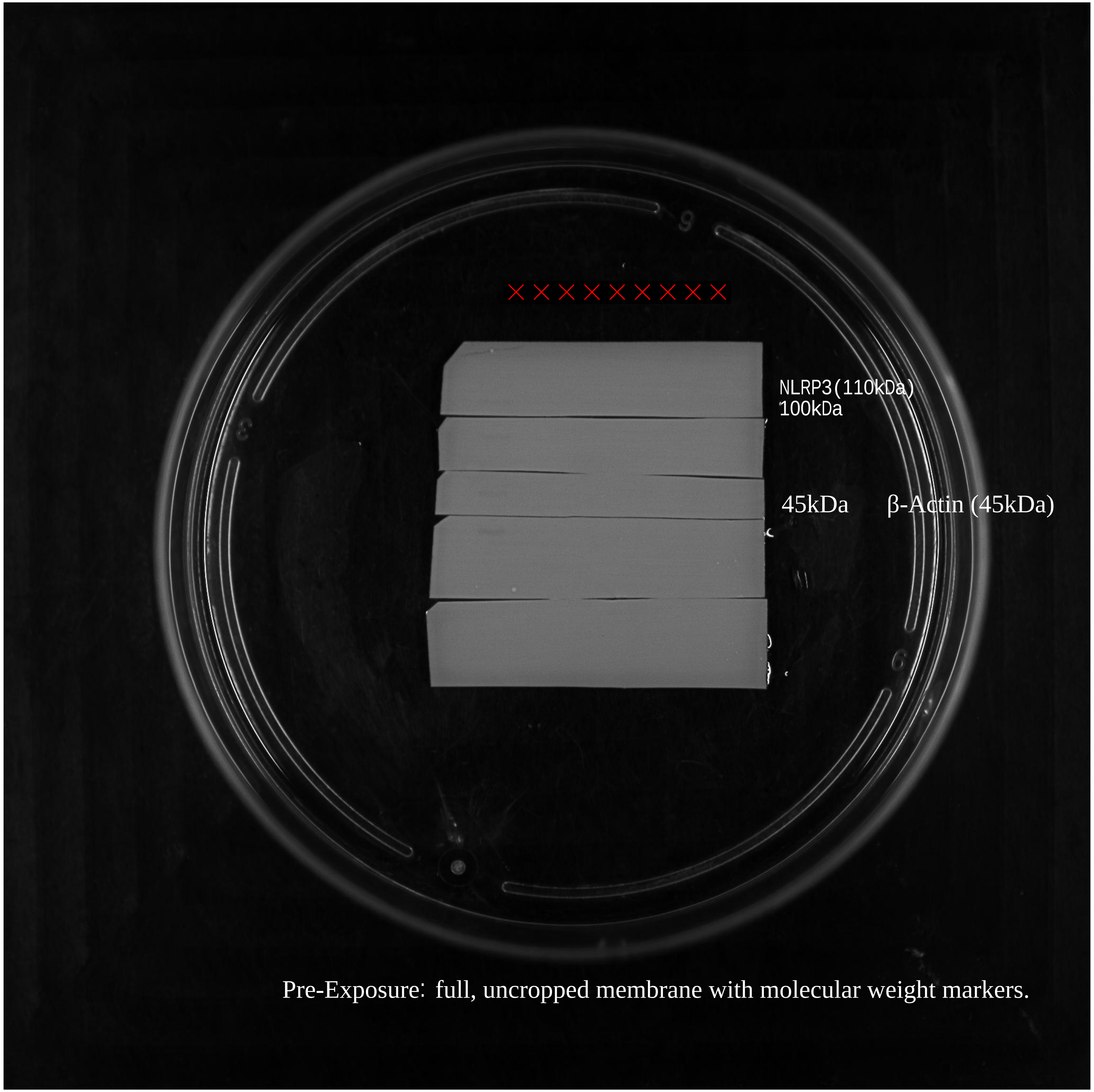

Con Mod NGR1 SSB2 NS

NLRP3-2

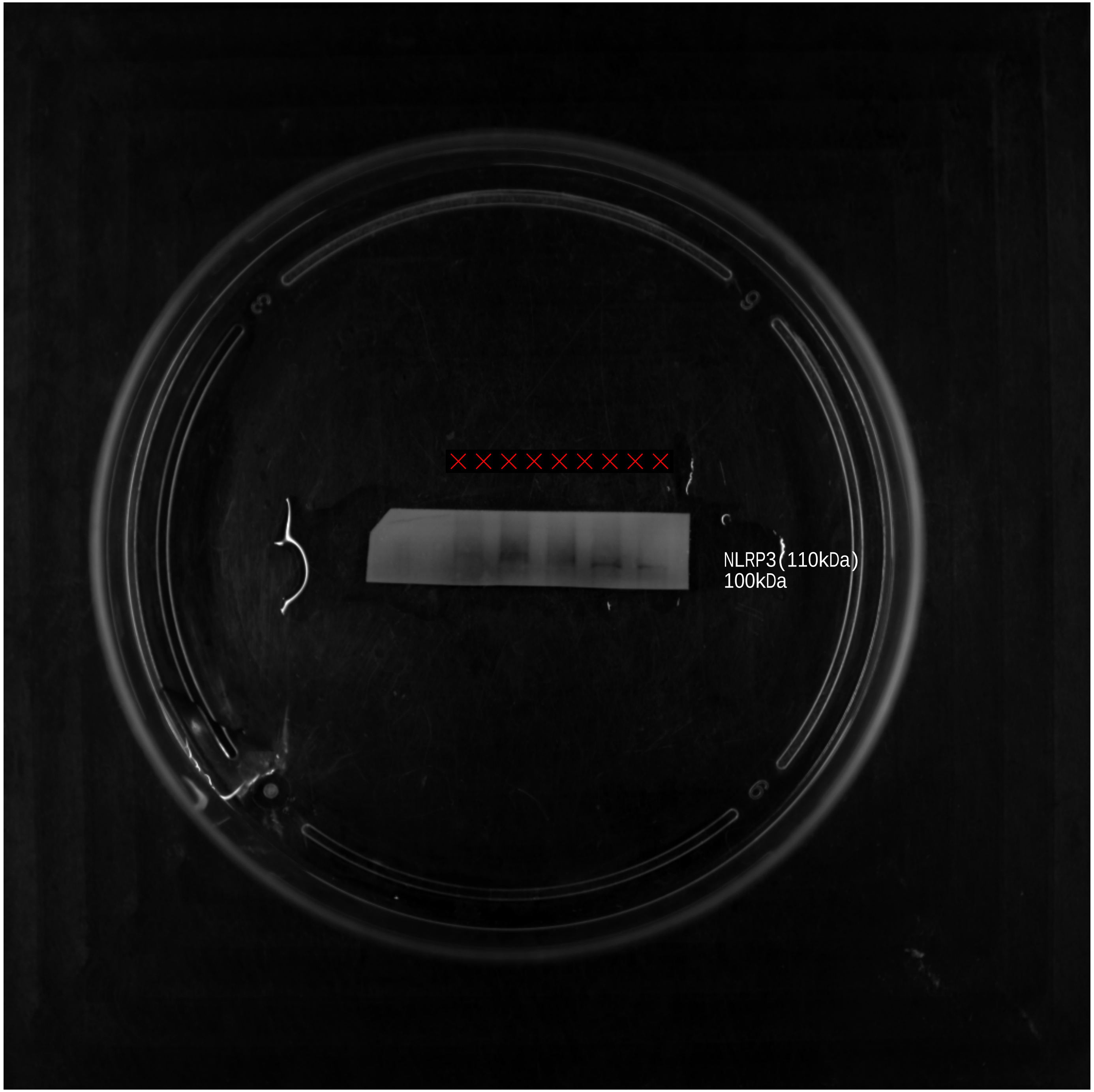

Con Mod NGR1 SSB2 NS

NLRP3-3-β-Actin

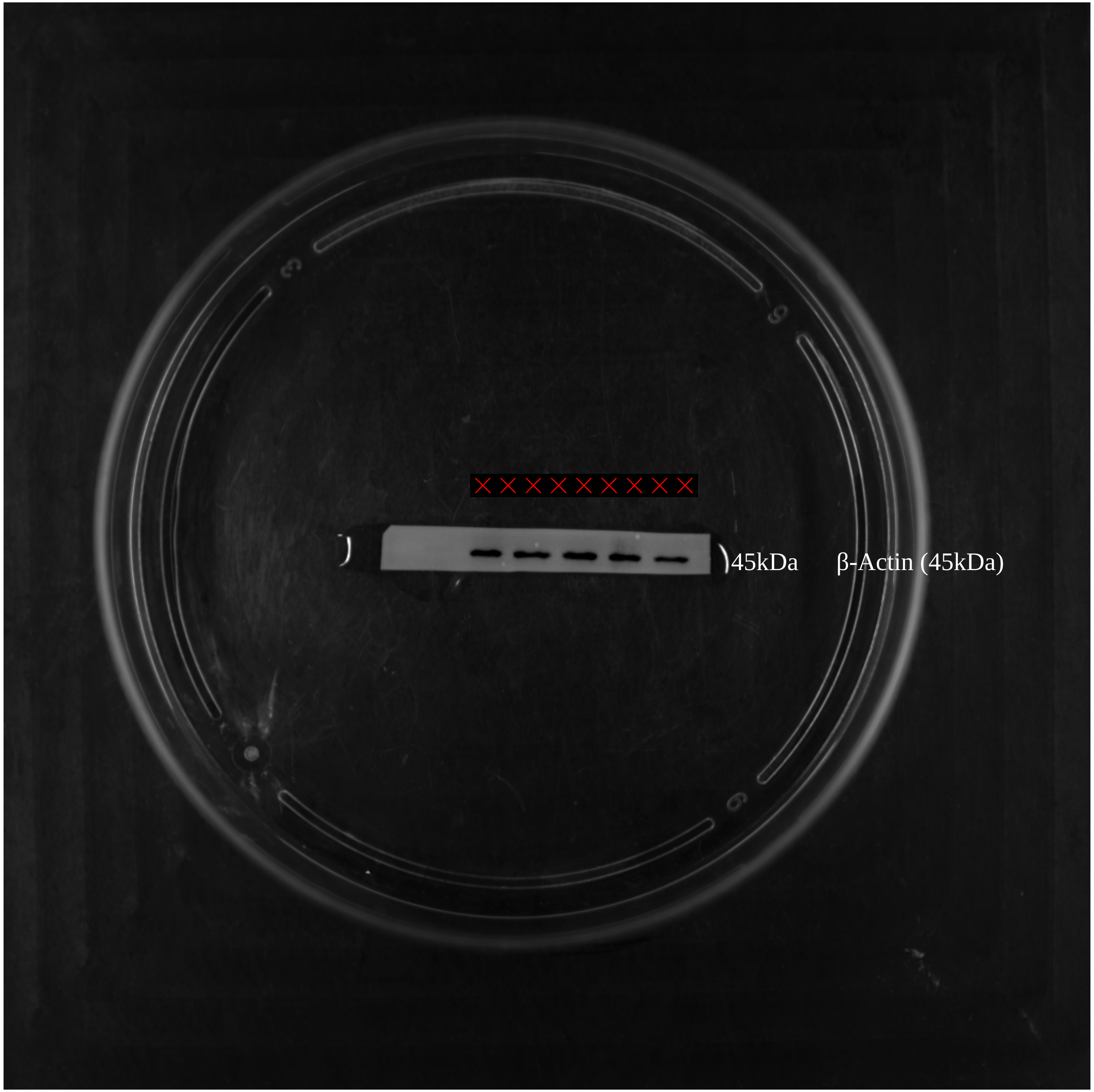

Con Mod NGR1 SSB2 NS

IL1β-3&4-full

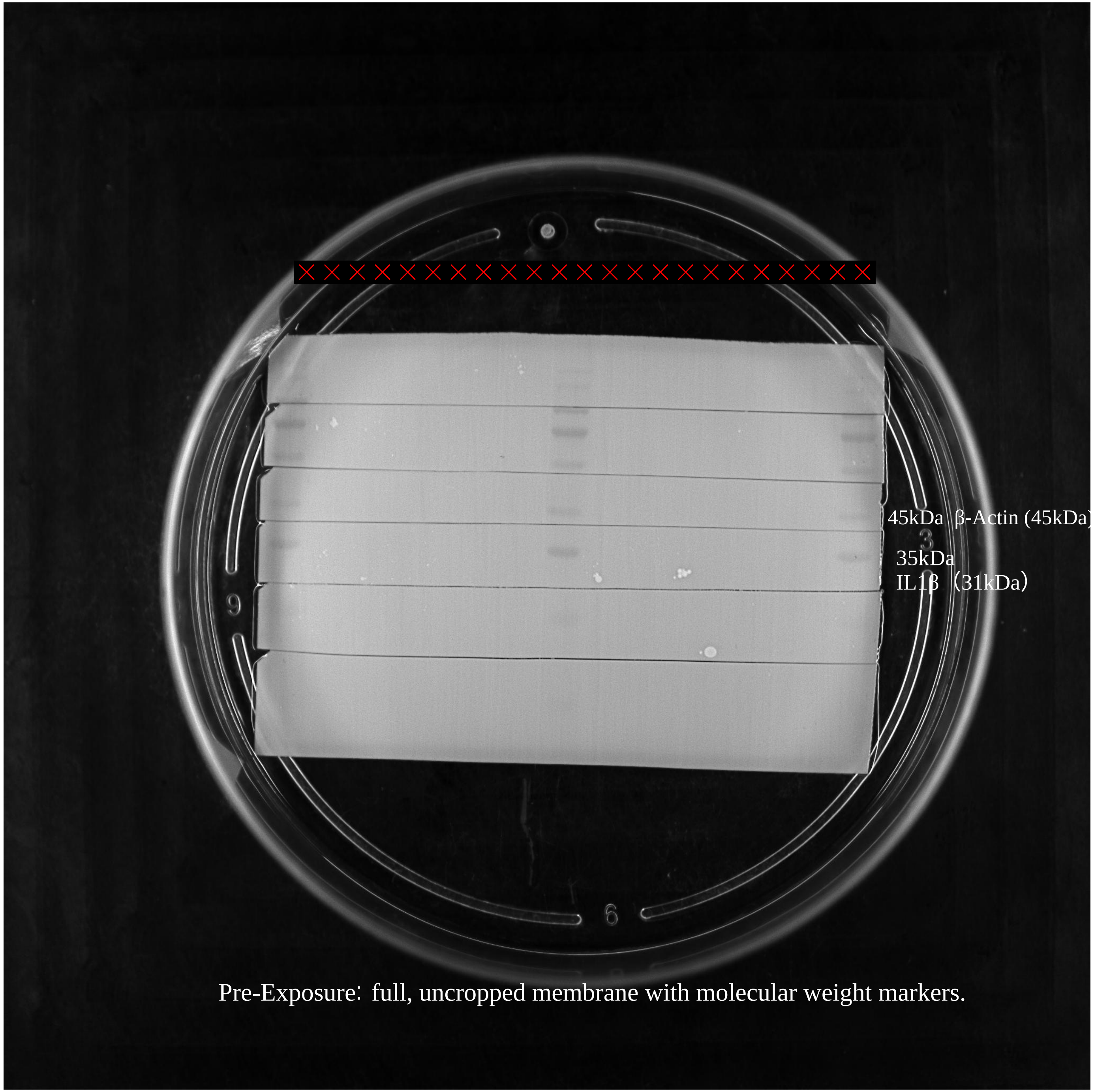

Con Mod NGR1 SSB2 NS Con Mod NGR1 SSB2 NS

IL1β-3&4

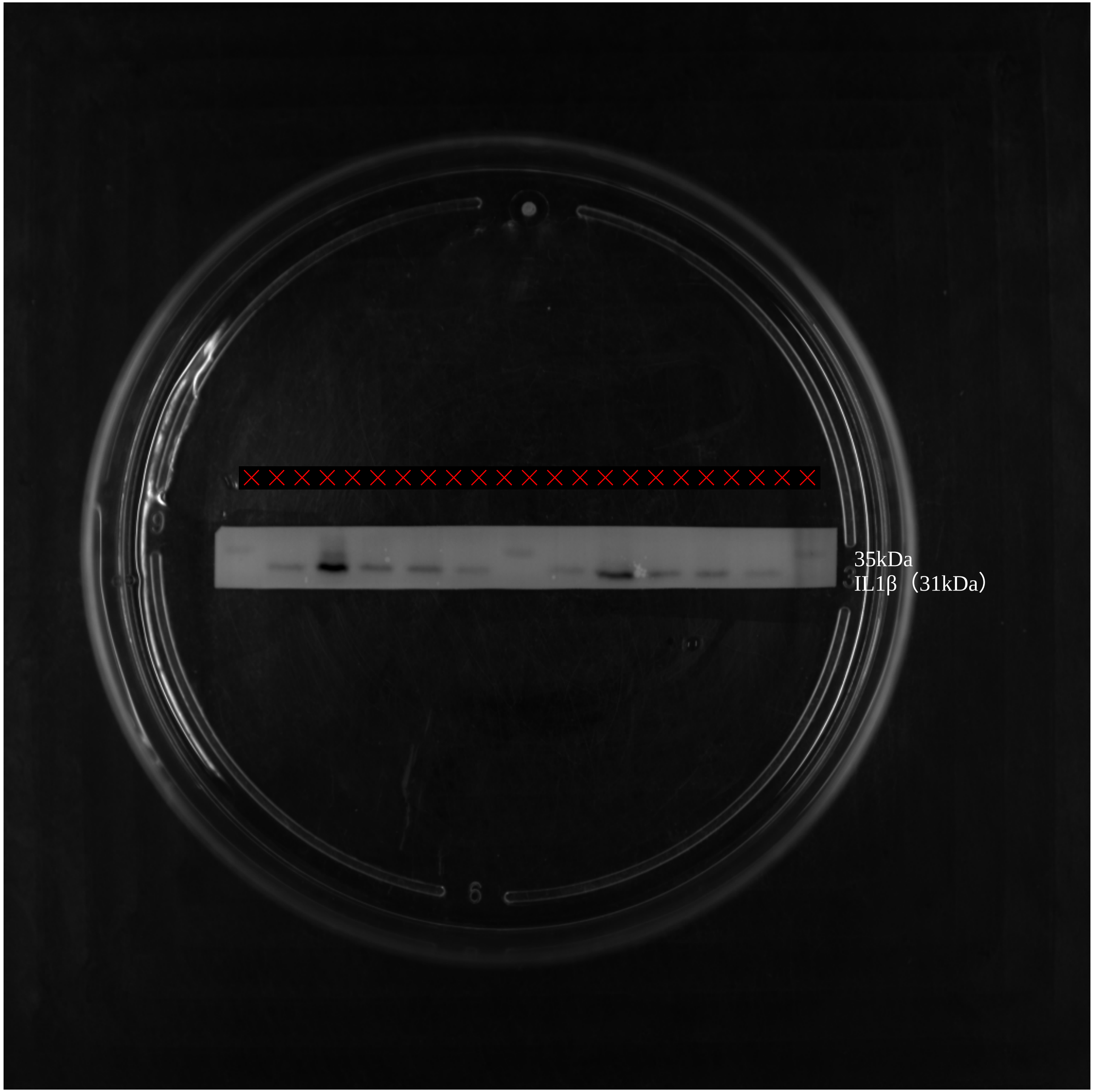

Con Mod NGR1 SSB2 NS   Con Mod NGR1 SSB2 NS

IL1β-3&4-β-Actin

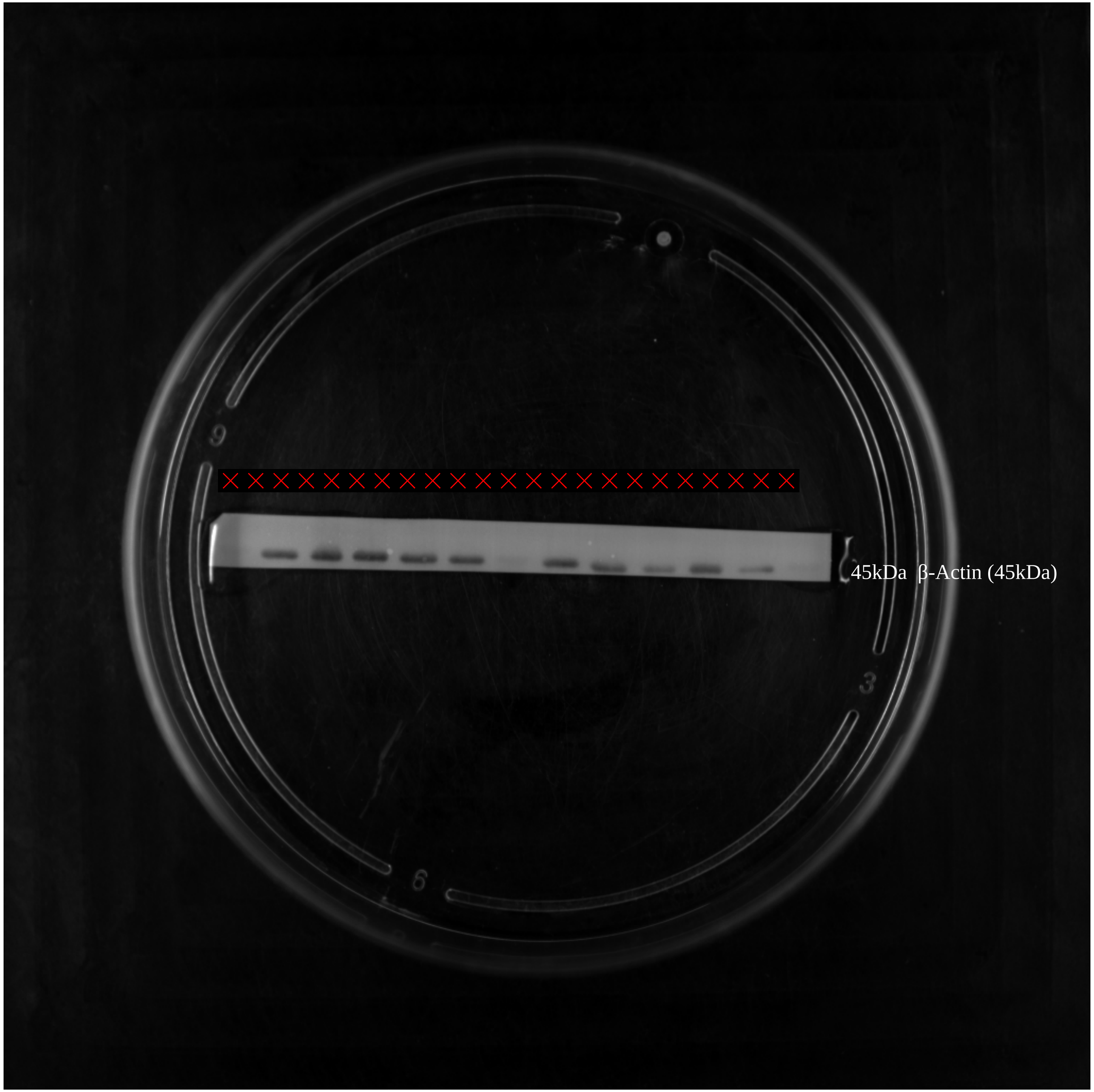

Con Mod NGR1 SSB2 NS Con Mod NGR1 SSB2 NS

IL18-3-full

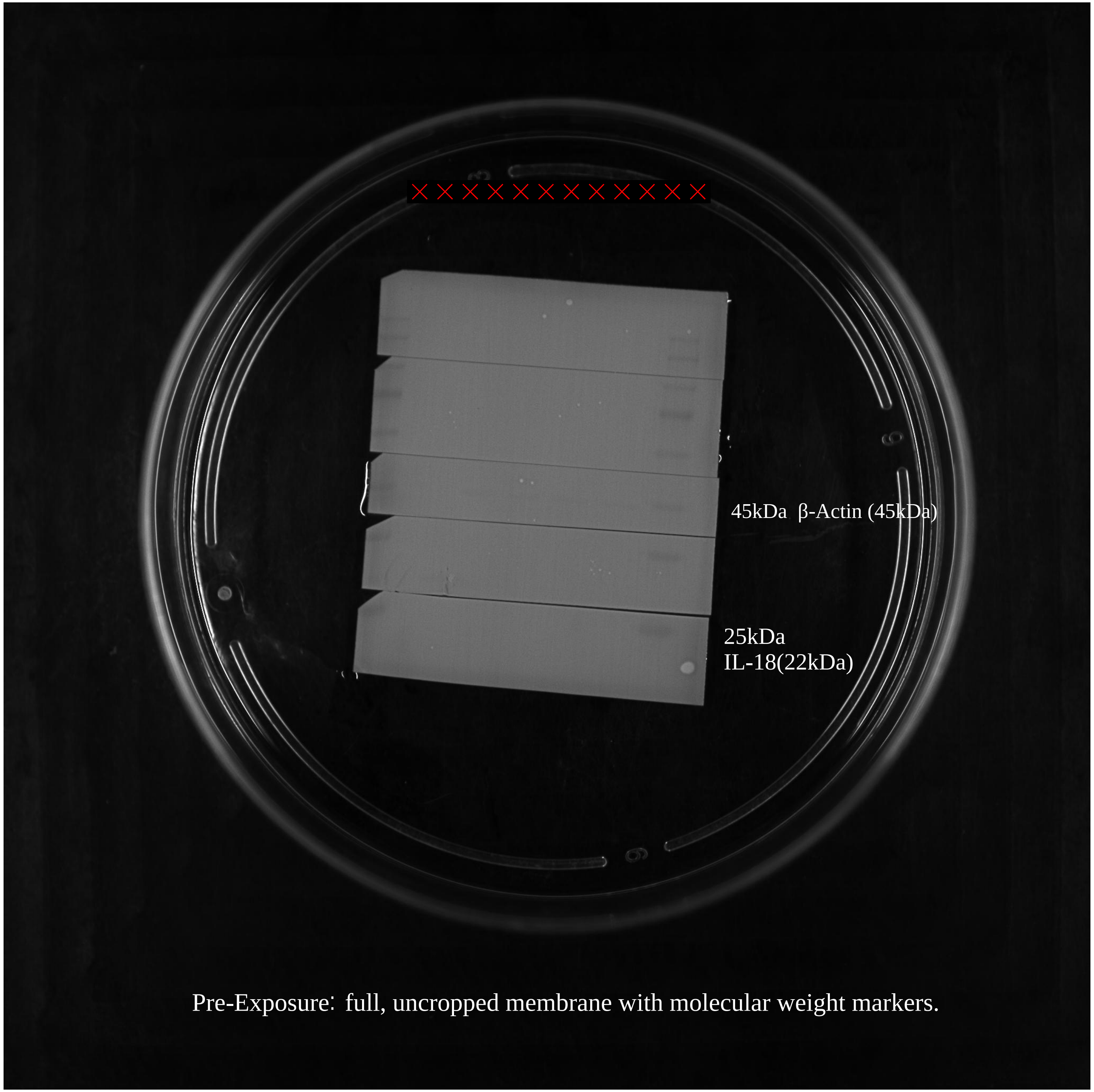

Con Mod NGR1 SSB2 NS

IL18-3

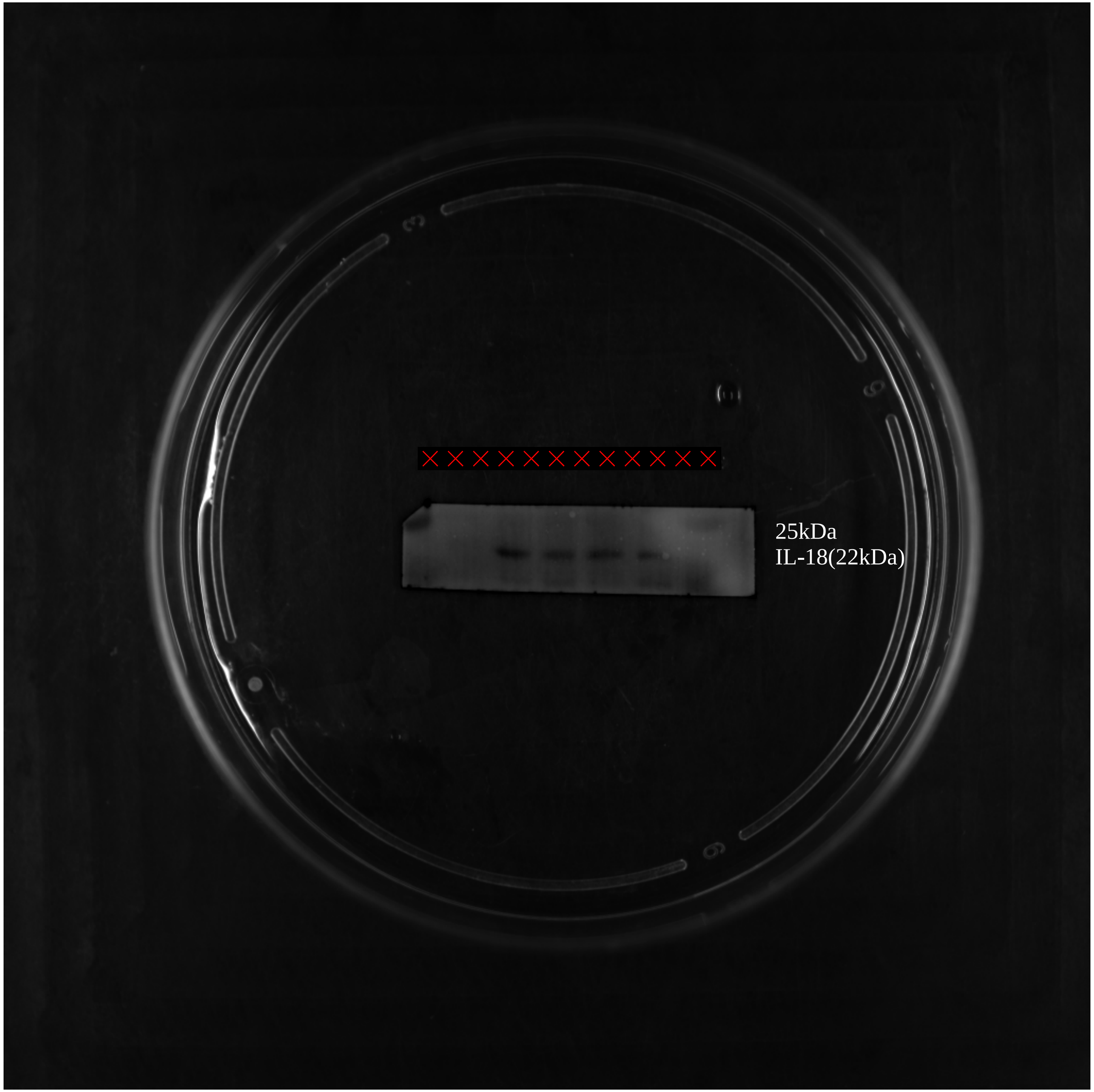

Con Mod NGR1 SSB2 NS

IL18-3-β-Actin

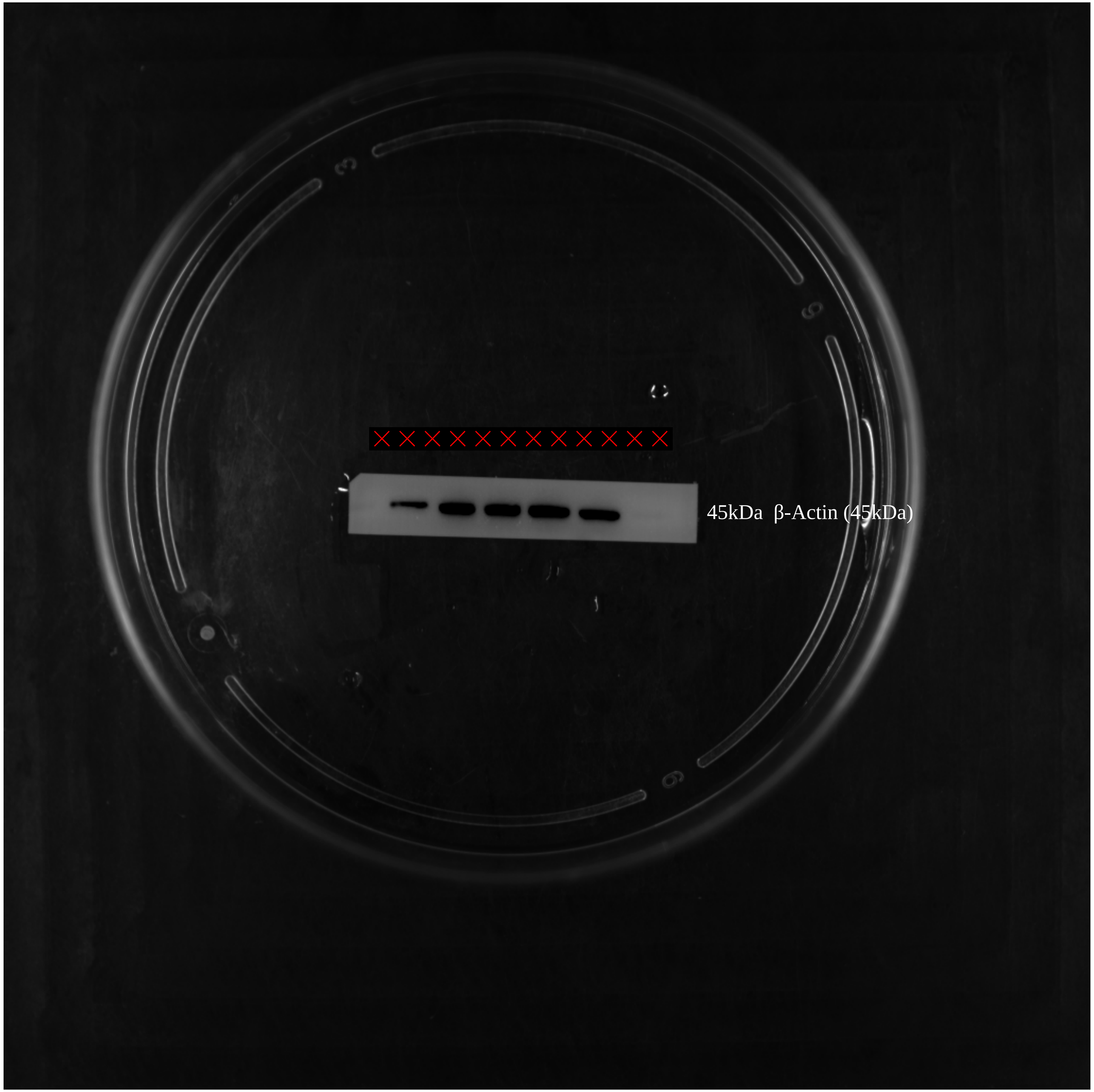

Con Mod NGR1 SSB2 NS

BAX-3&4-full

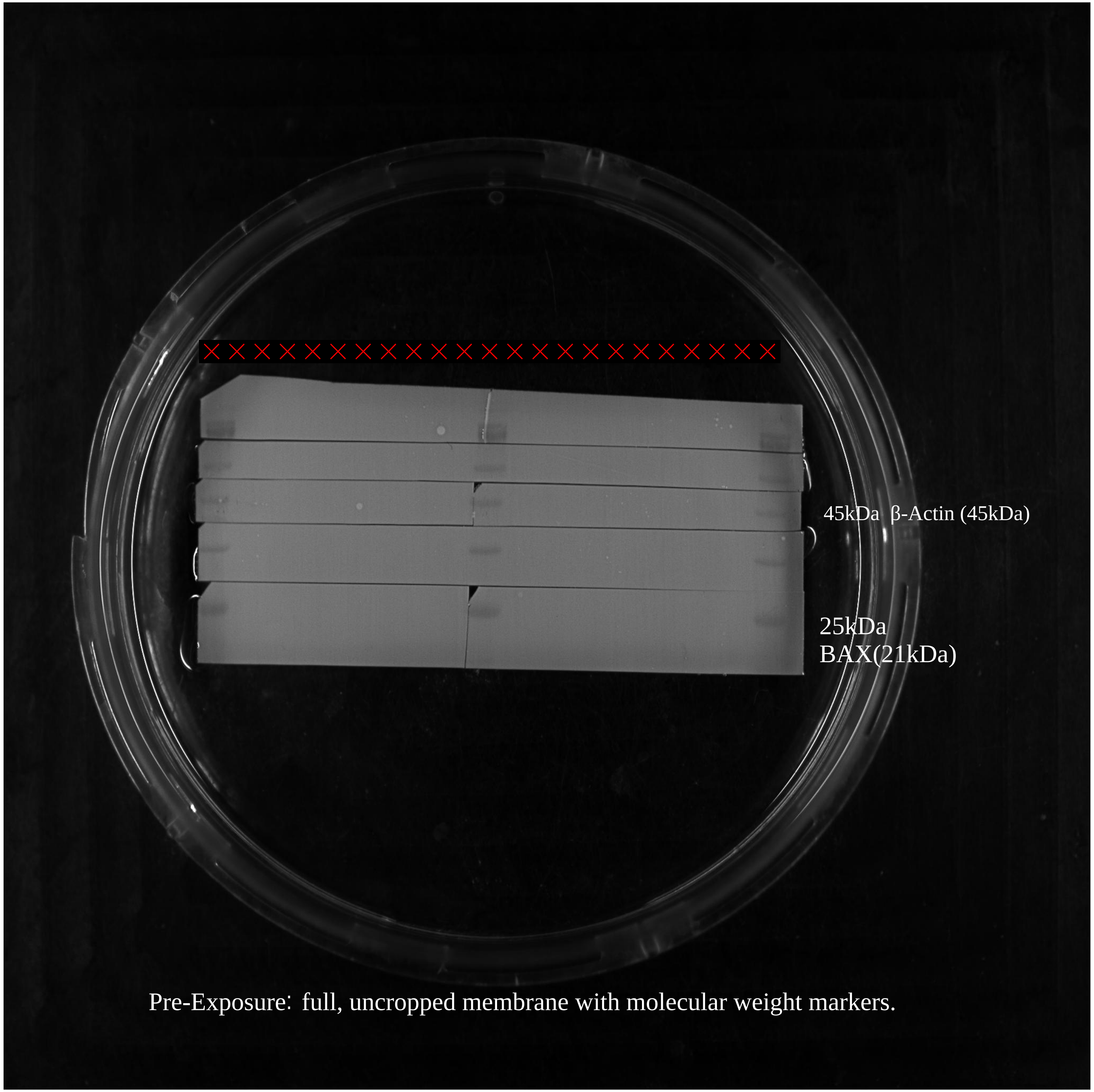

Con Mod NGR1 SSB2 NS Con Mod NGR1 SSB2 NS

BAX-3&4

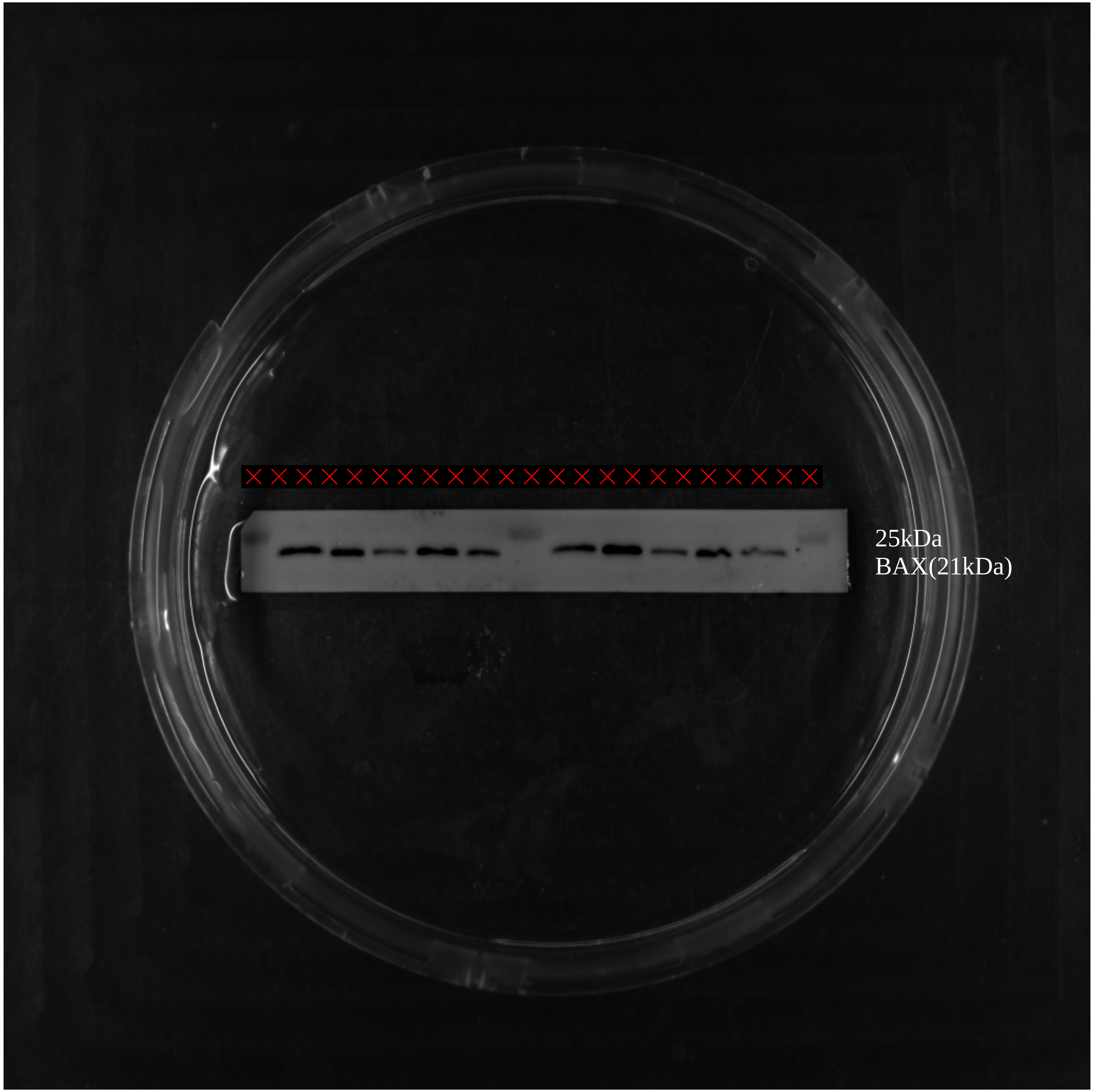

Con Mod NGR1 SSB2 NS Con Mod NGR1 SSB2 NS

BAX-3&4-β-Actin

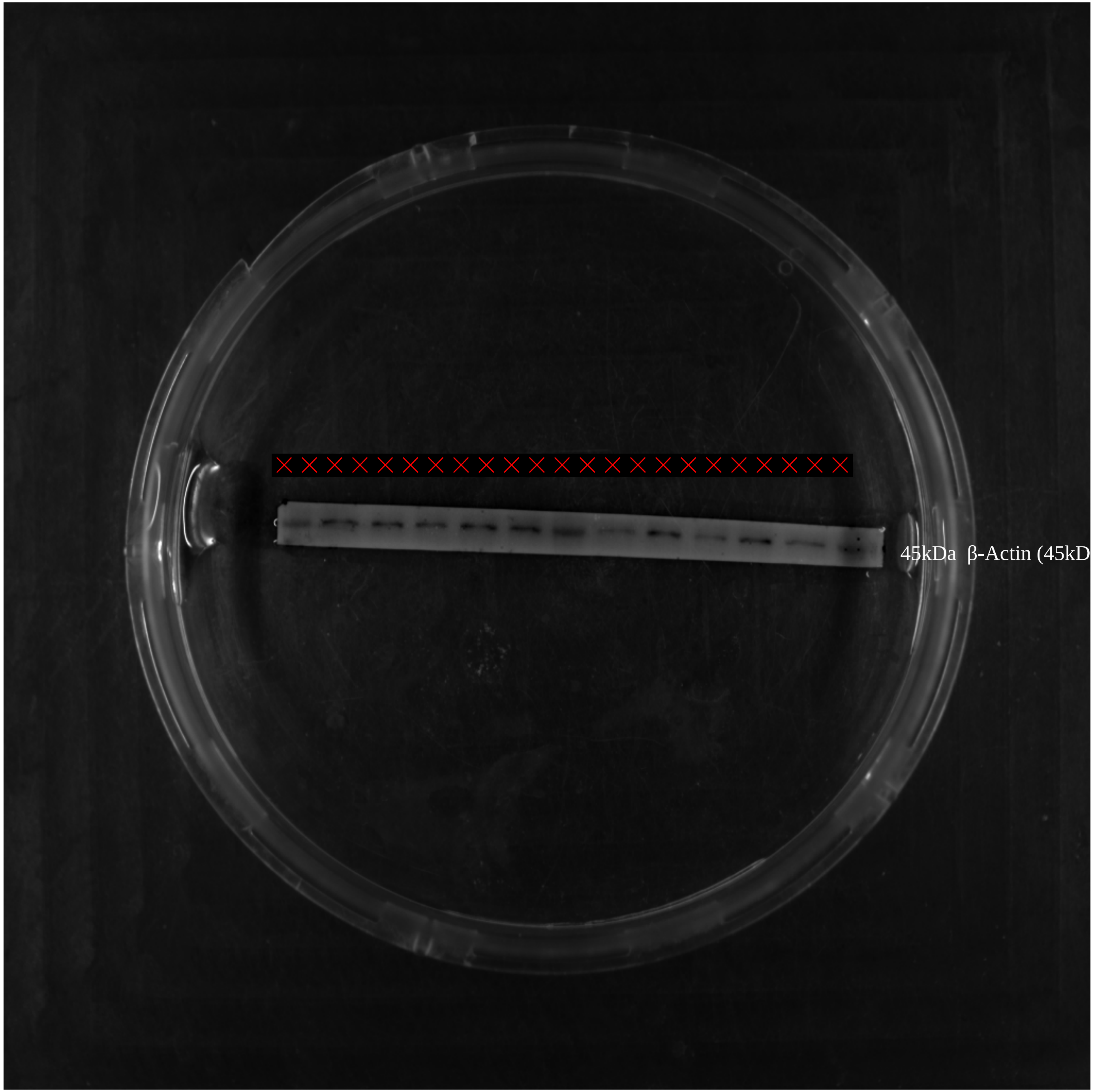

Con Mod NGR1 SSB2 NS Con Mod NGR1 SSB2 NS

BCL2-2&3-full

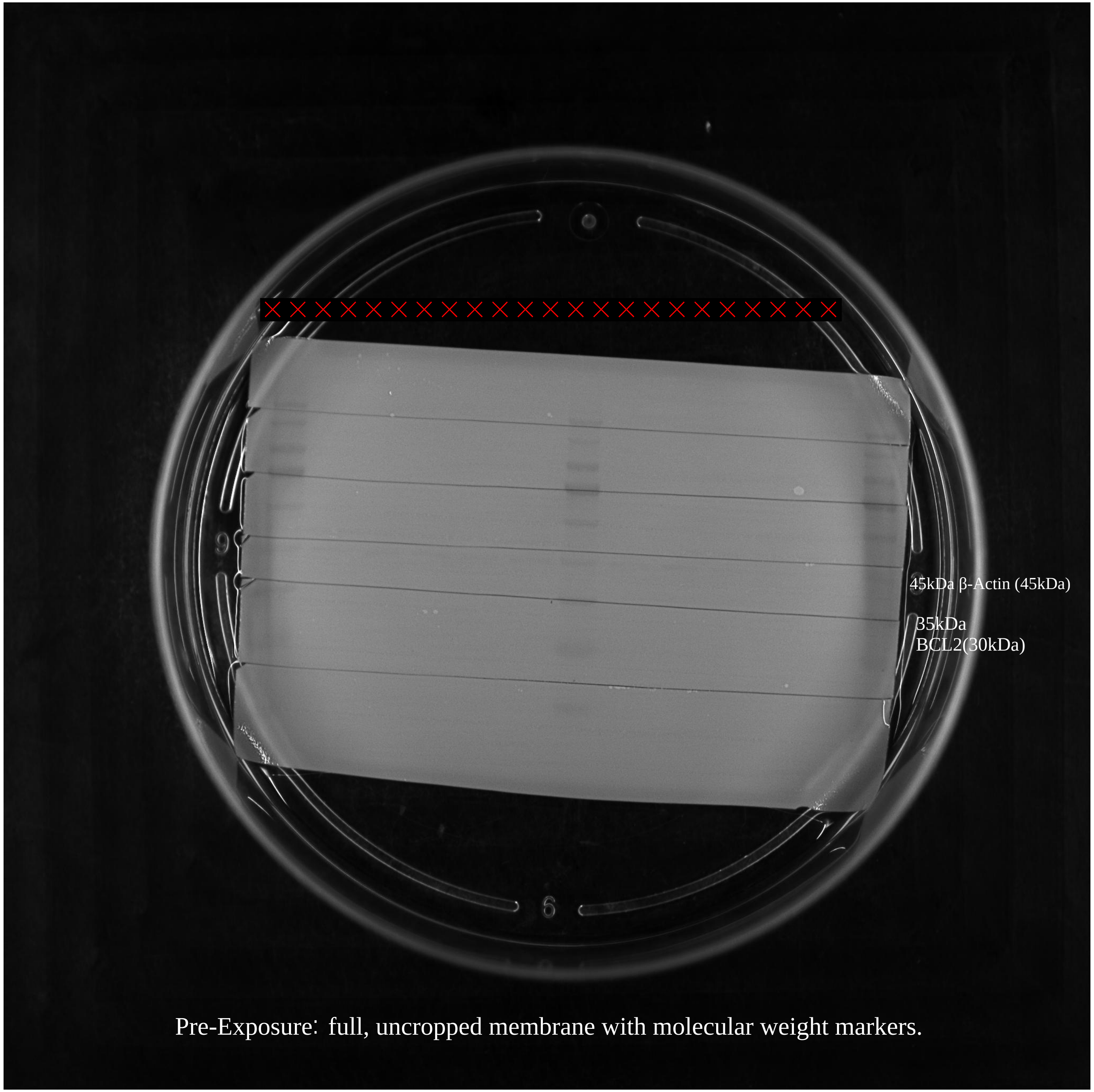

Pre-Exposure: full, uncropped membrane with molecular weight markers.

Con Mod NGR1 SSB2 NS   Con Mod NGR1 SSB2 NS

BCL2-2&3

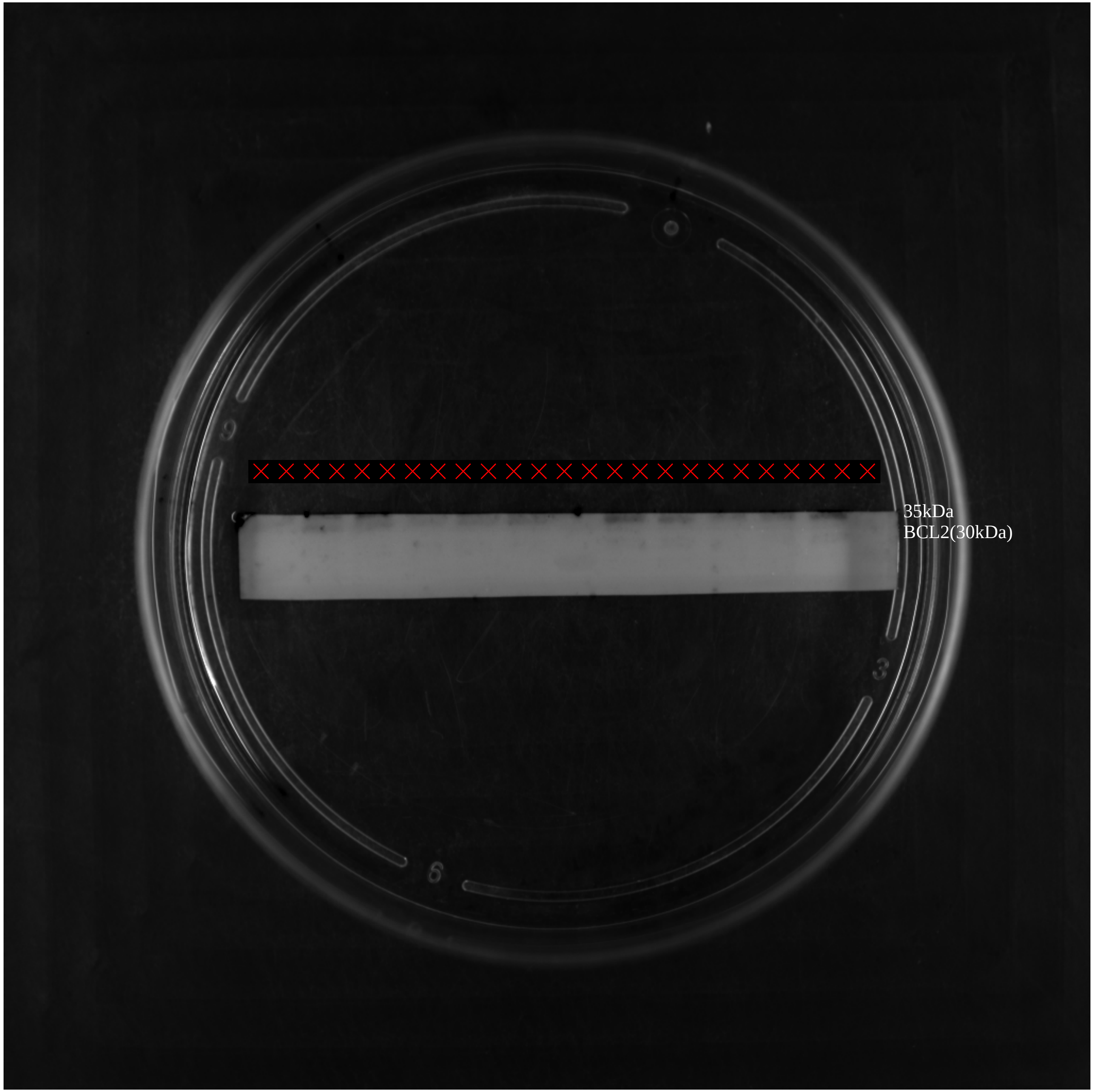

Con Mod NGR1 SSB2 NS Con Mod NGR1 SSB2 NS

BCL2-2&3-β-Actin

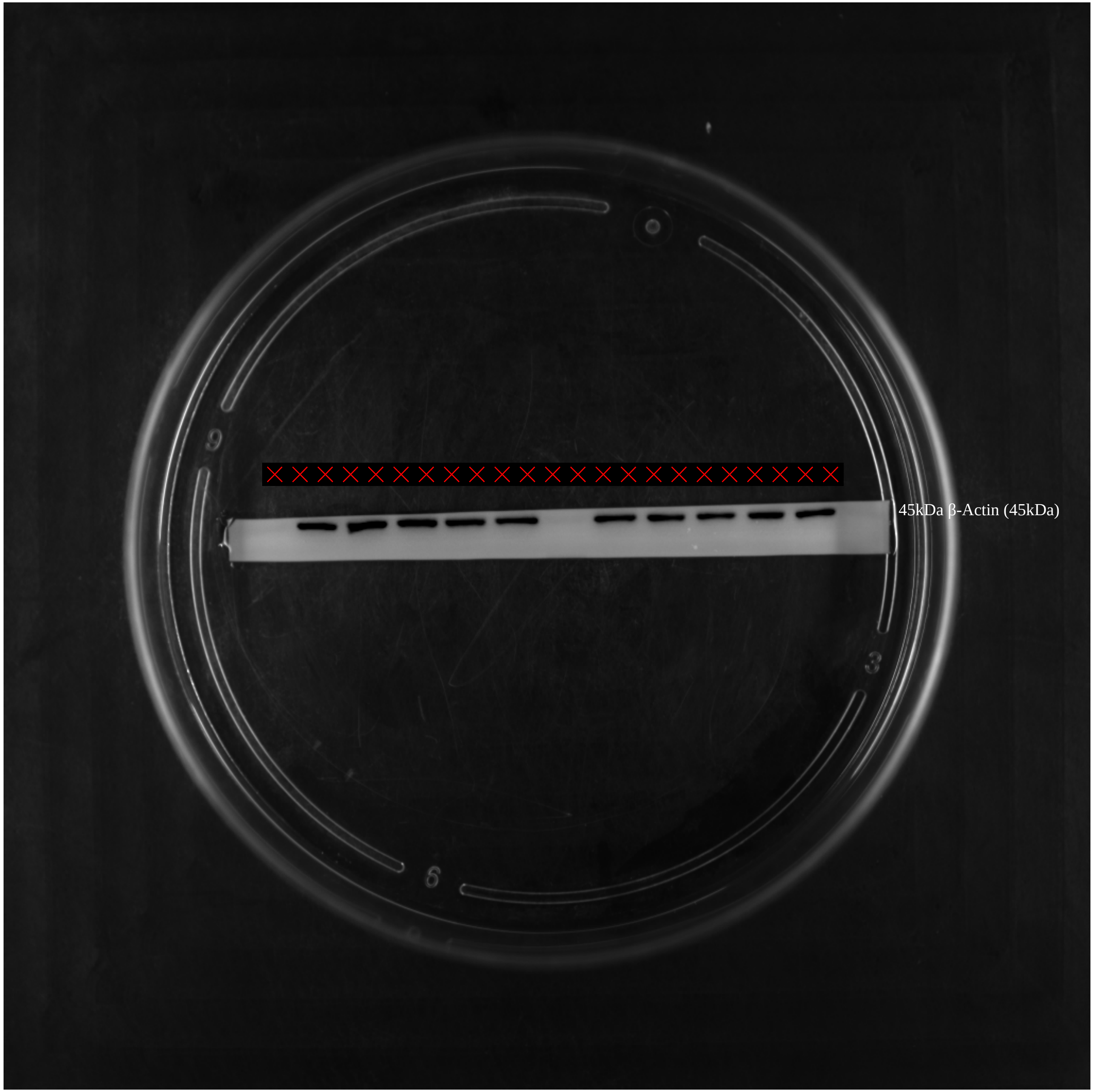

Con Mod NGR1 SSB2 NS Con Mod NGR1 SSB2 NS

Caspase-3-2-full

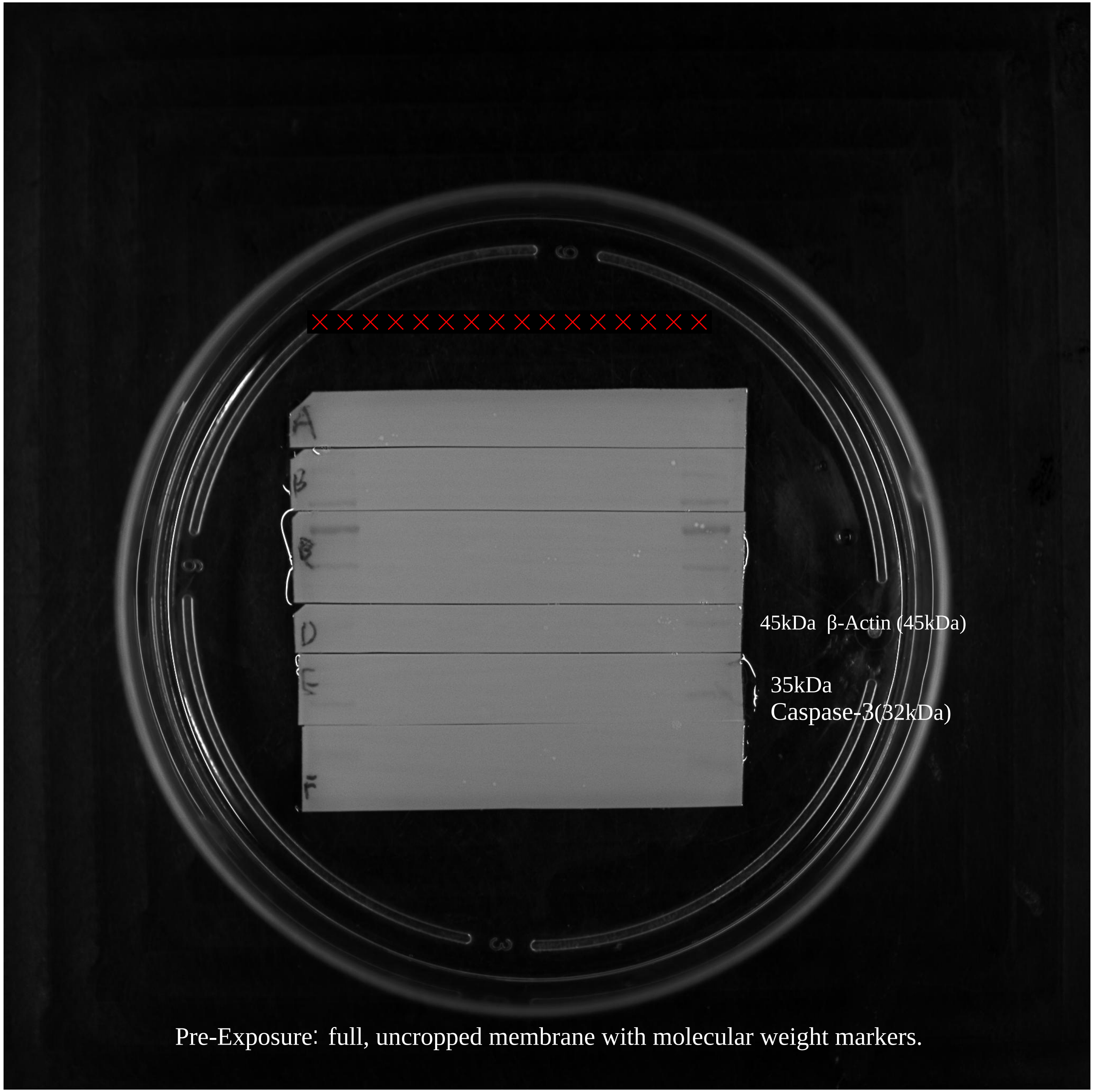

Pre-Exposure: full, uncropped membrane with molecular weight markers.

Con Mod NGR1 SSB2 NS

Caspase-3-2

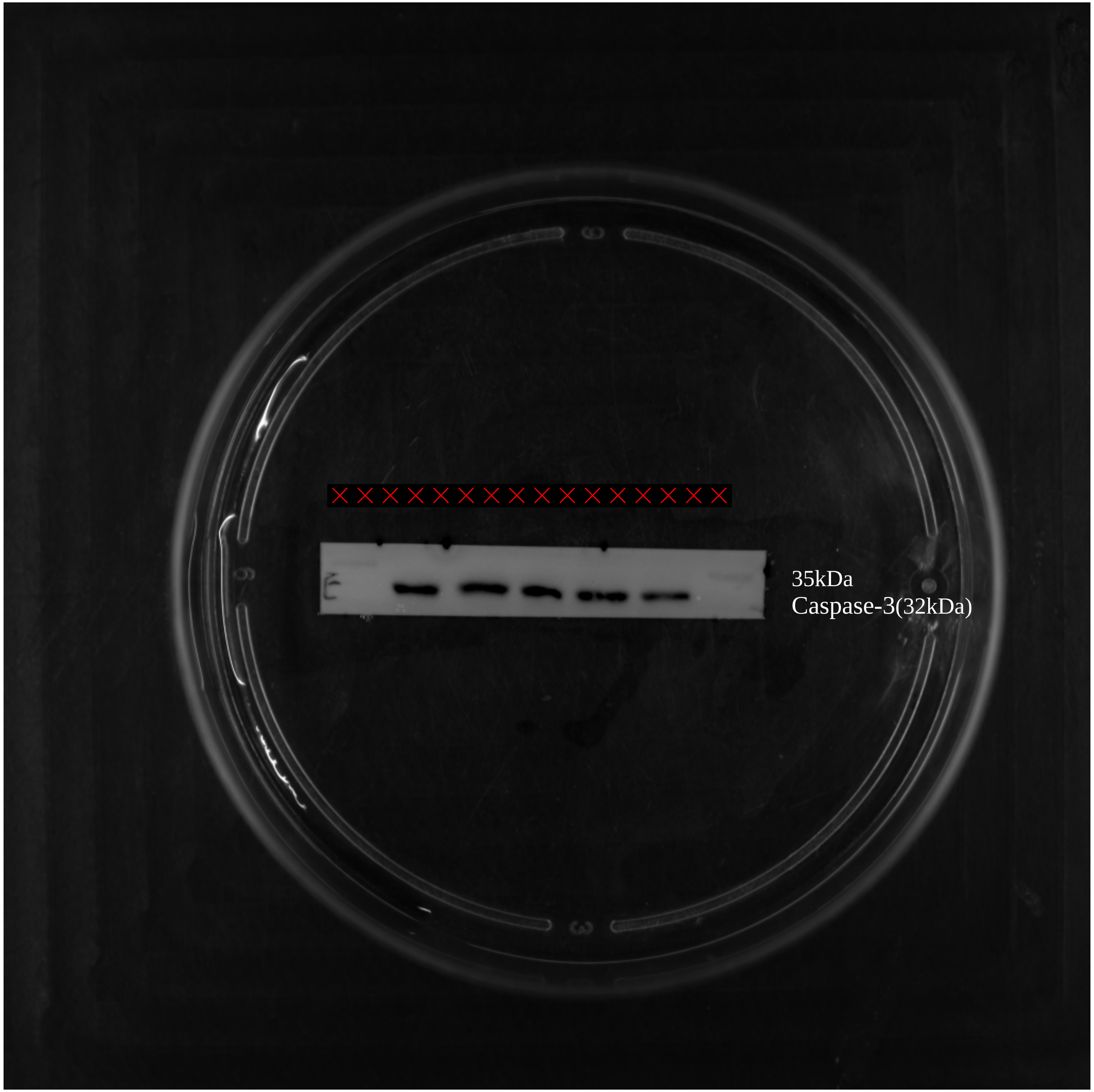

Con Mod NGR1 SSB2 NS

Caspase-3-2-β-Actin

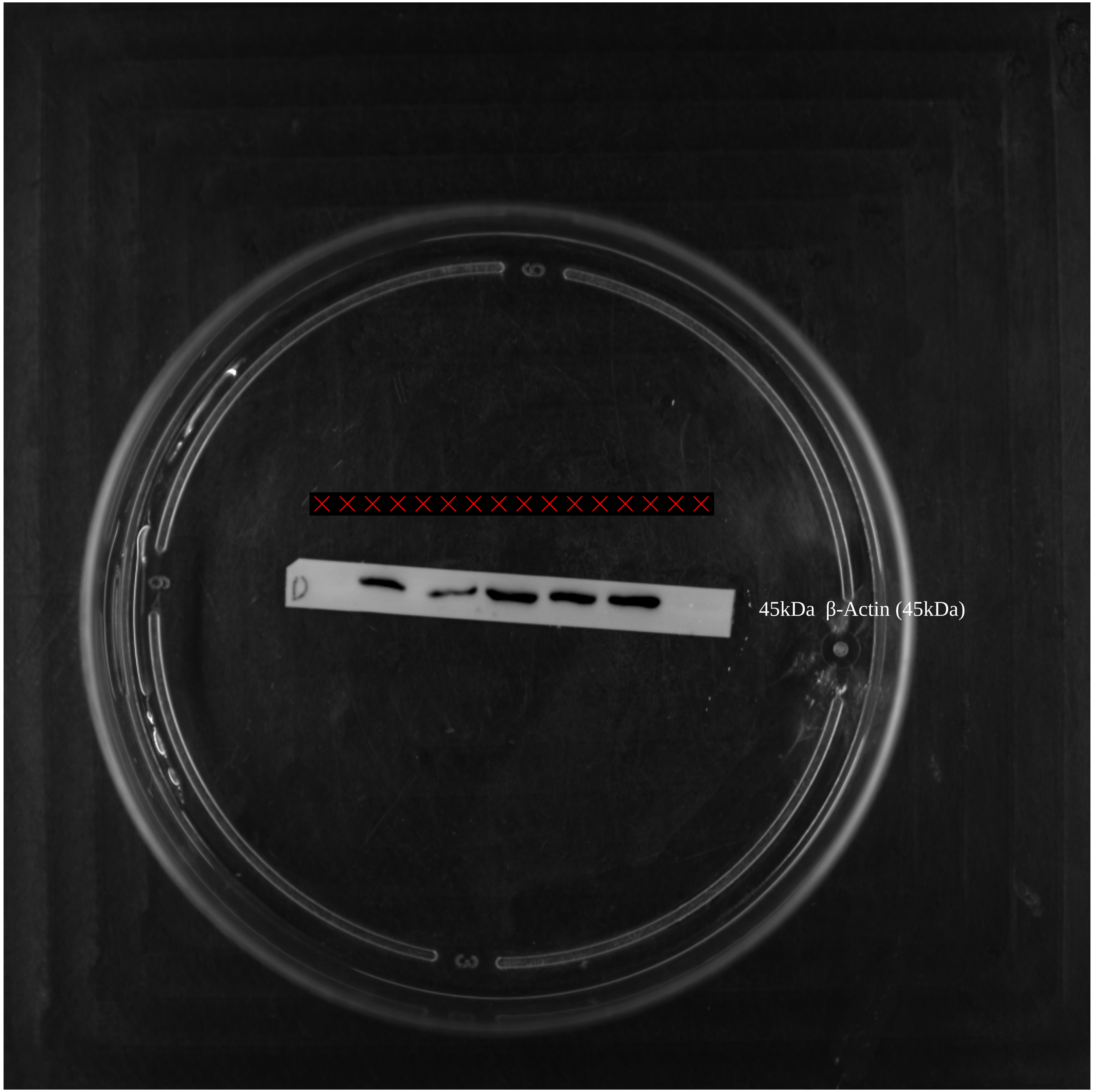

Con Mod NGR1 SSB2 NS

Caspase-3-3-full

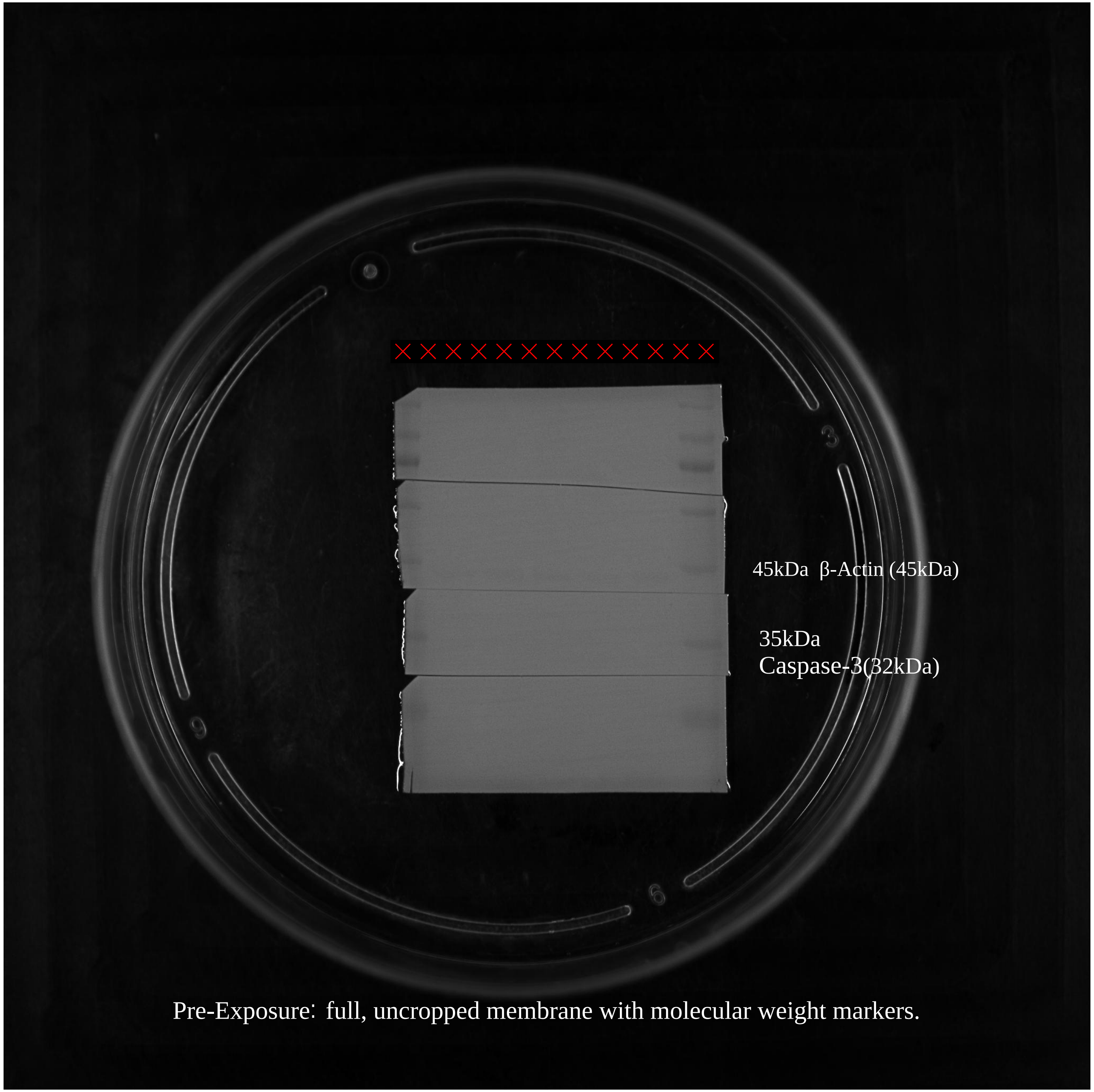

Con Mod NGR1 SSB2 NS

Caspase-3-3

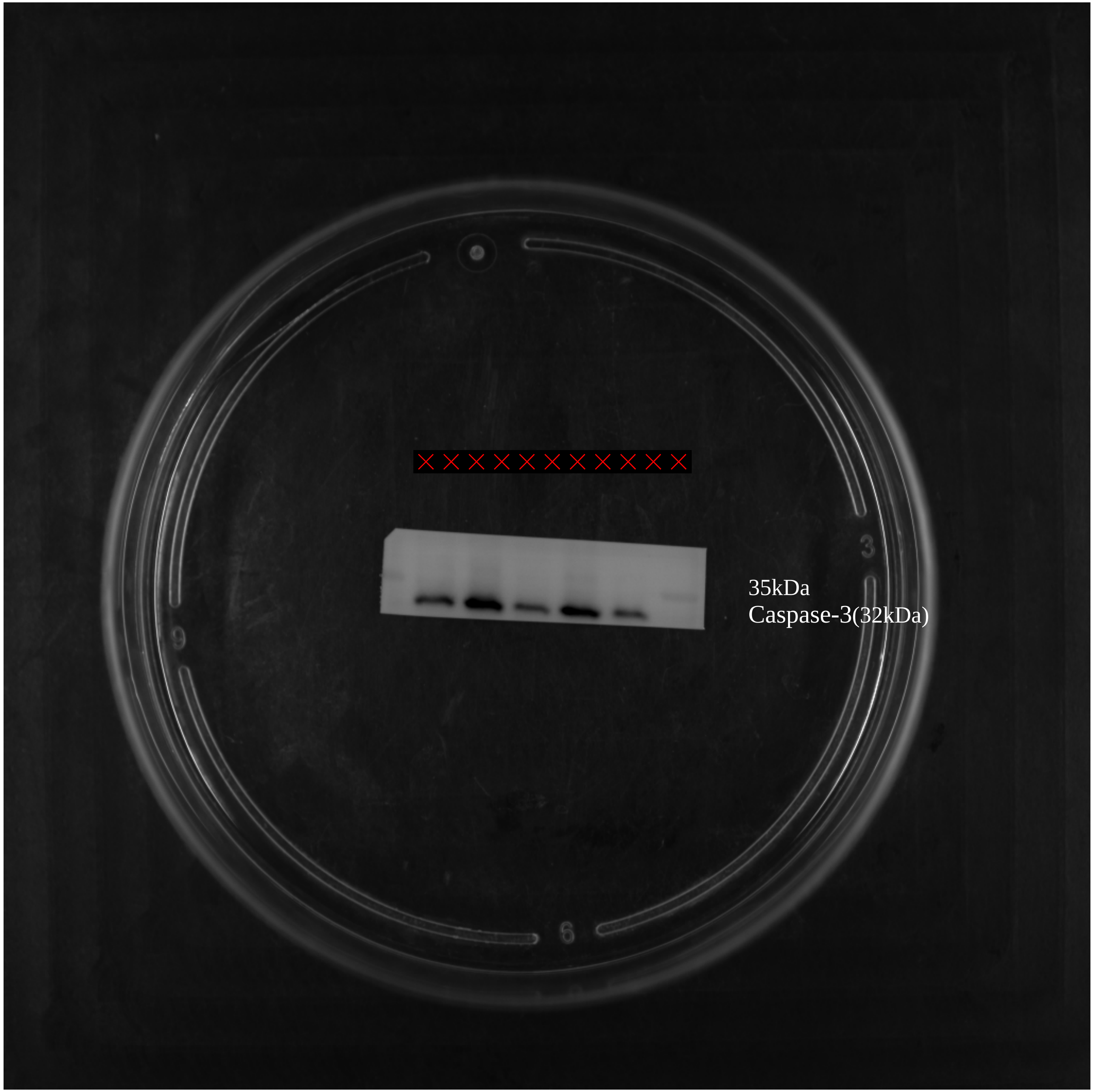

Con Mod NGR1 SSB2 NS

Caspase-3-3-β-Actin

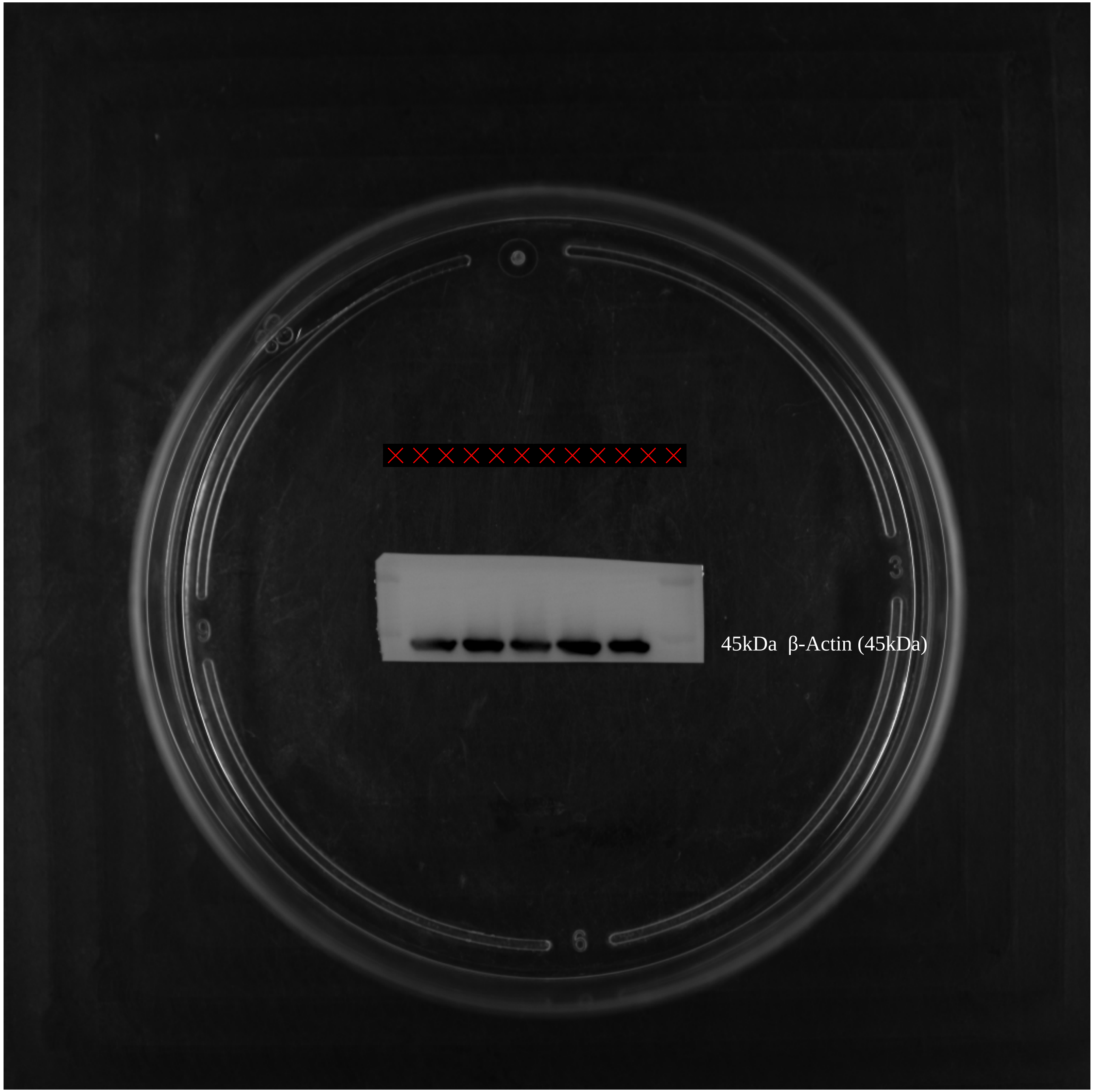

Con Mod NGR1 SSB2 NS

p-PI3K-2-full

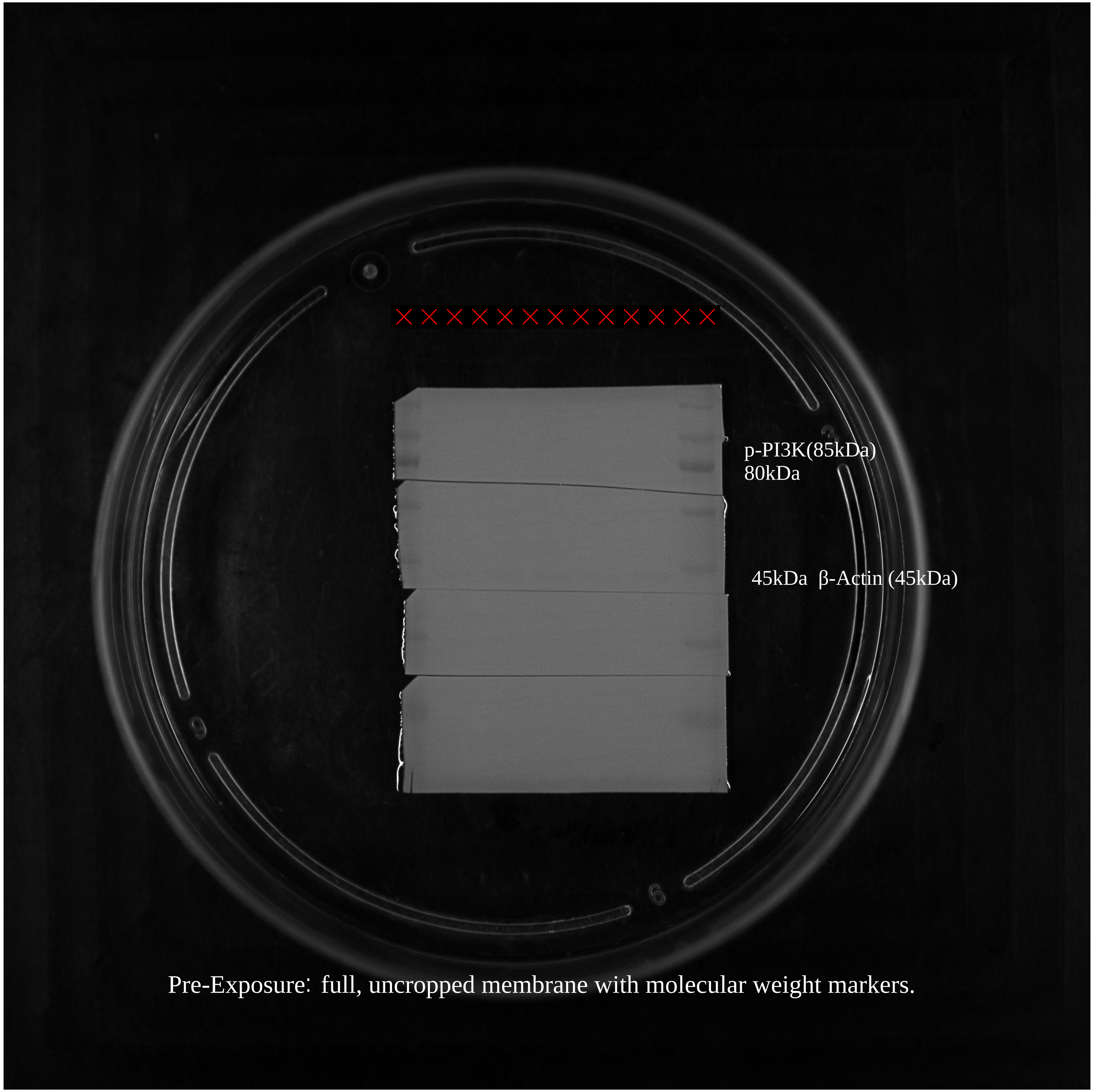

Con Mod NGR1 SSB2 NS

p-PI3K-2

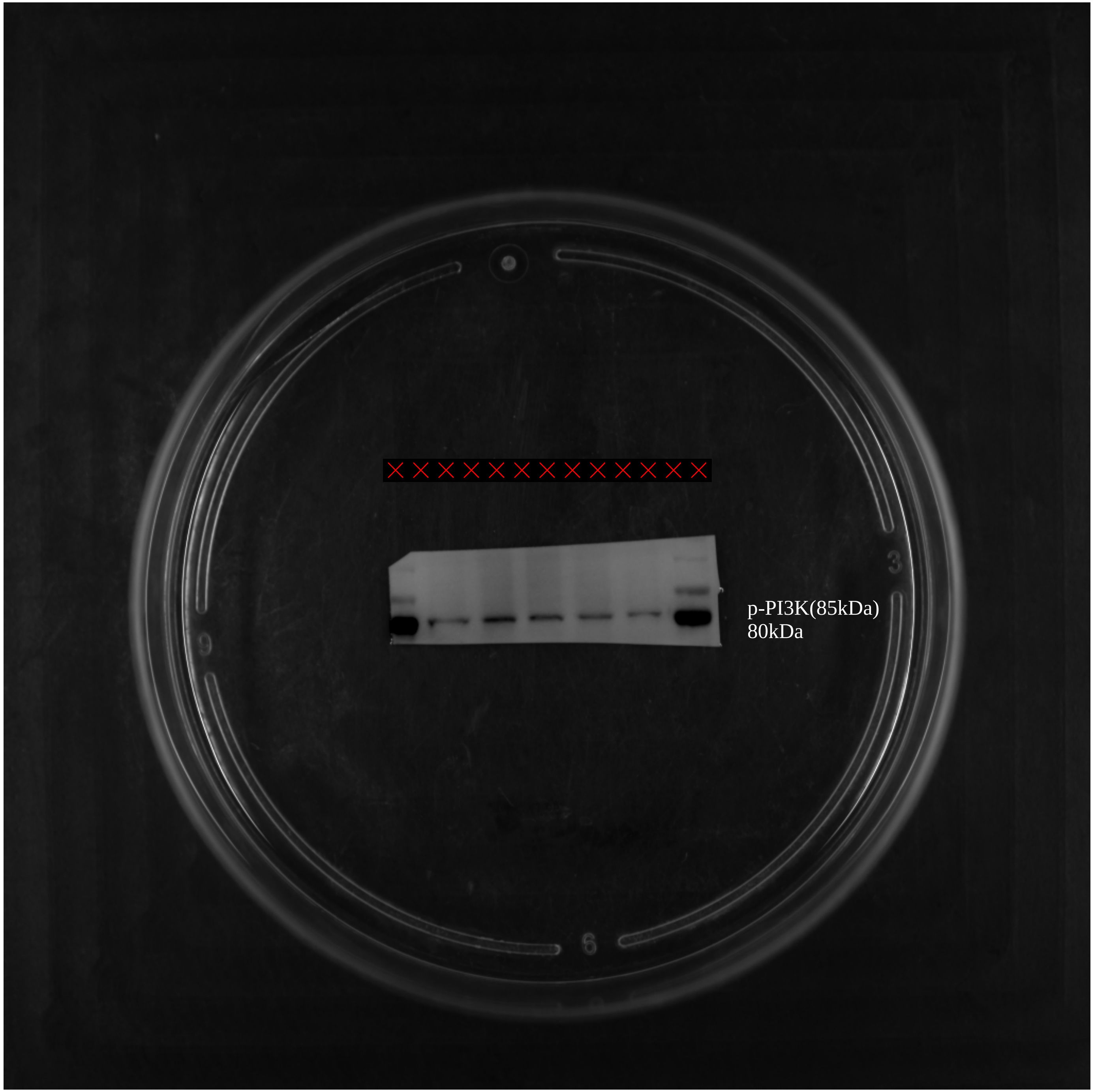

Con Mod NGR1 SSB2 NS

p-PI3K-2-β-Actin

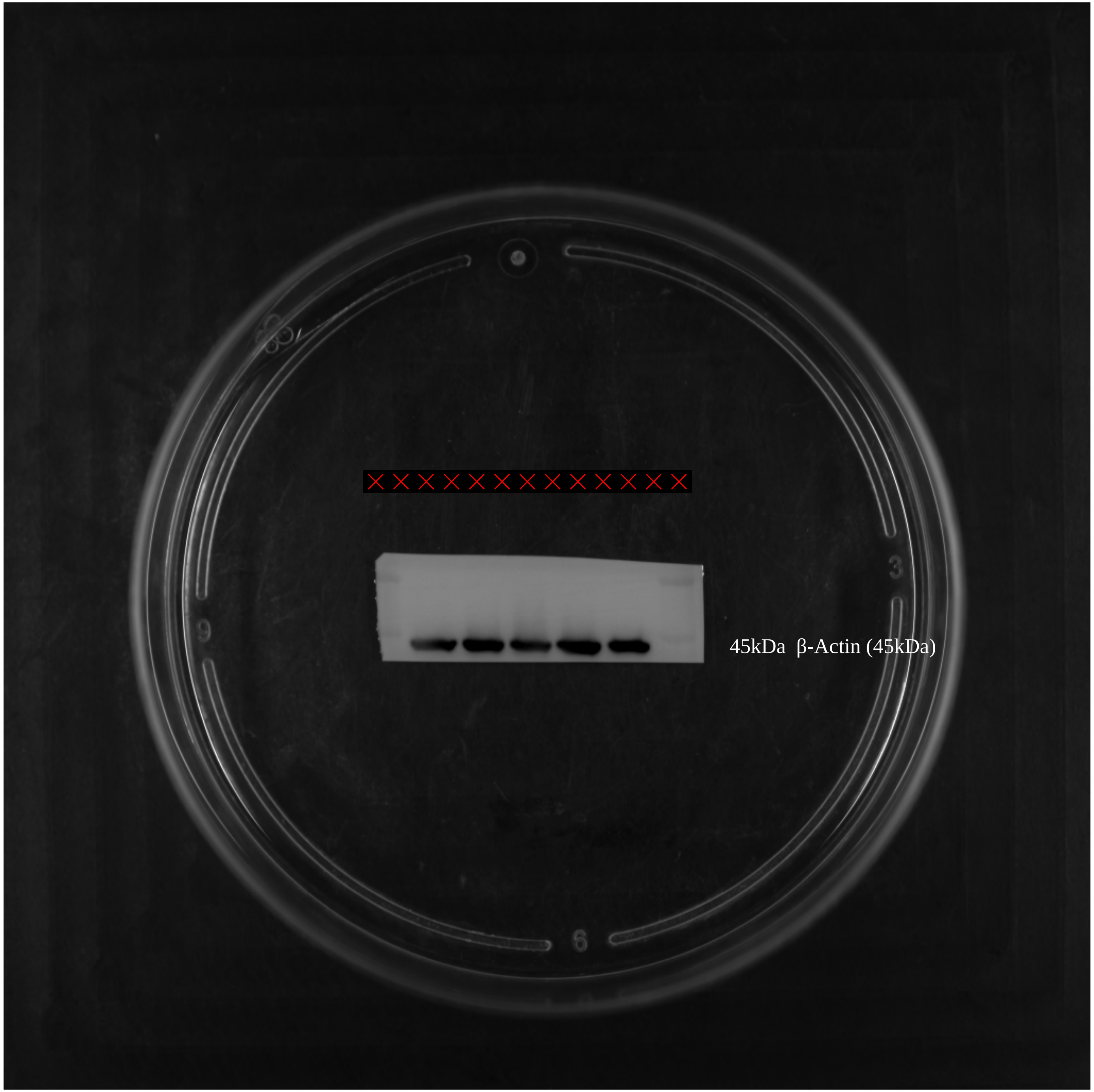

Con Mod NGR1 SSB2 NS

p-PI3K-3&4-full

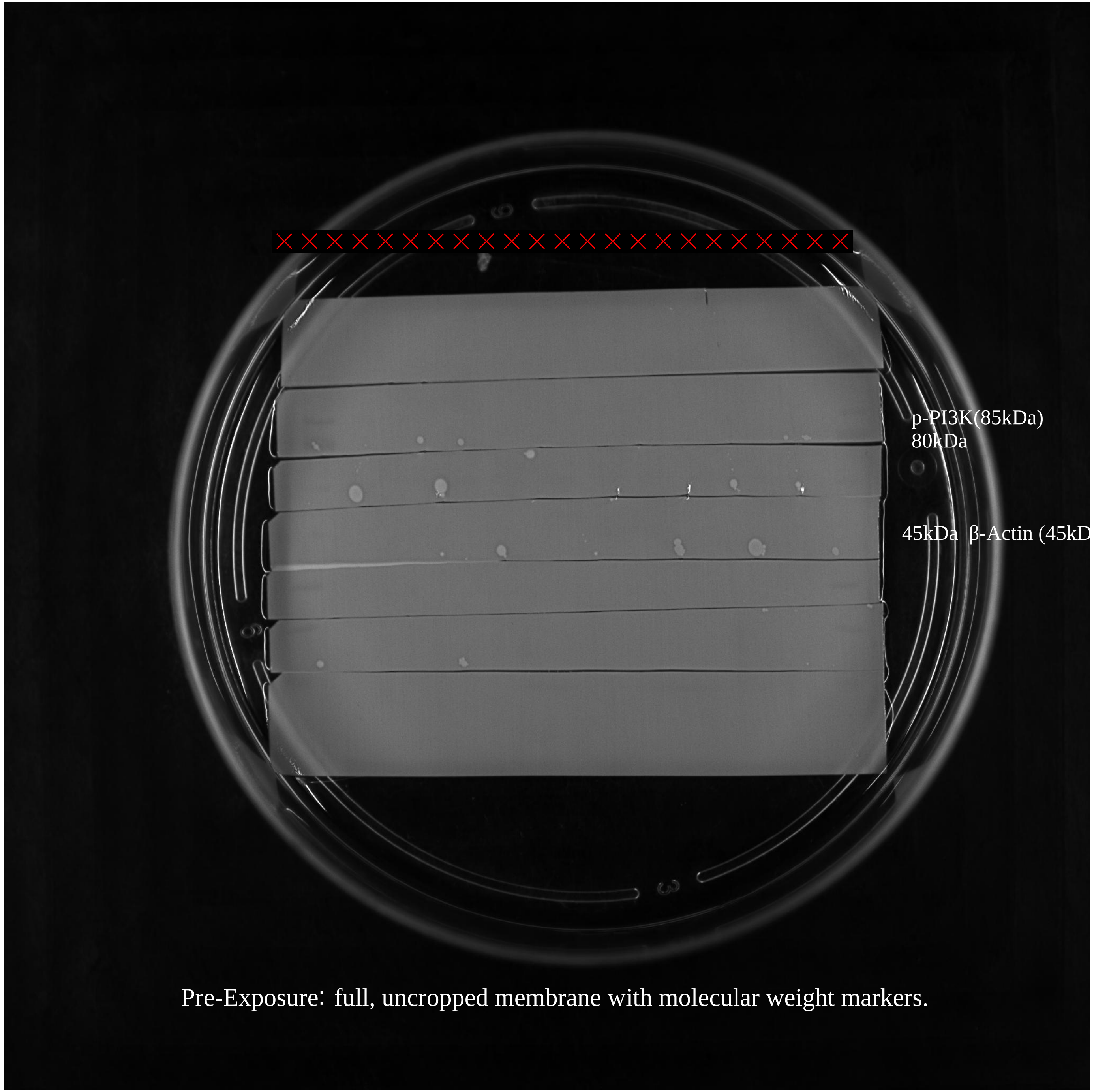

Pre-Exposure: full, uncropped membrane with molecular weight markers.

Con Mod NGR1 SSB2 NS Con Mod NGR1 SSB2 NS

p-PI3K-3&4

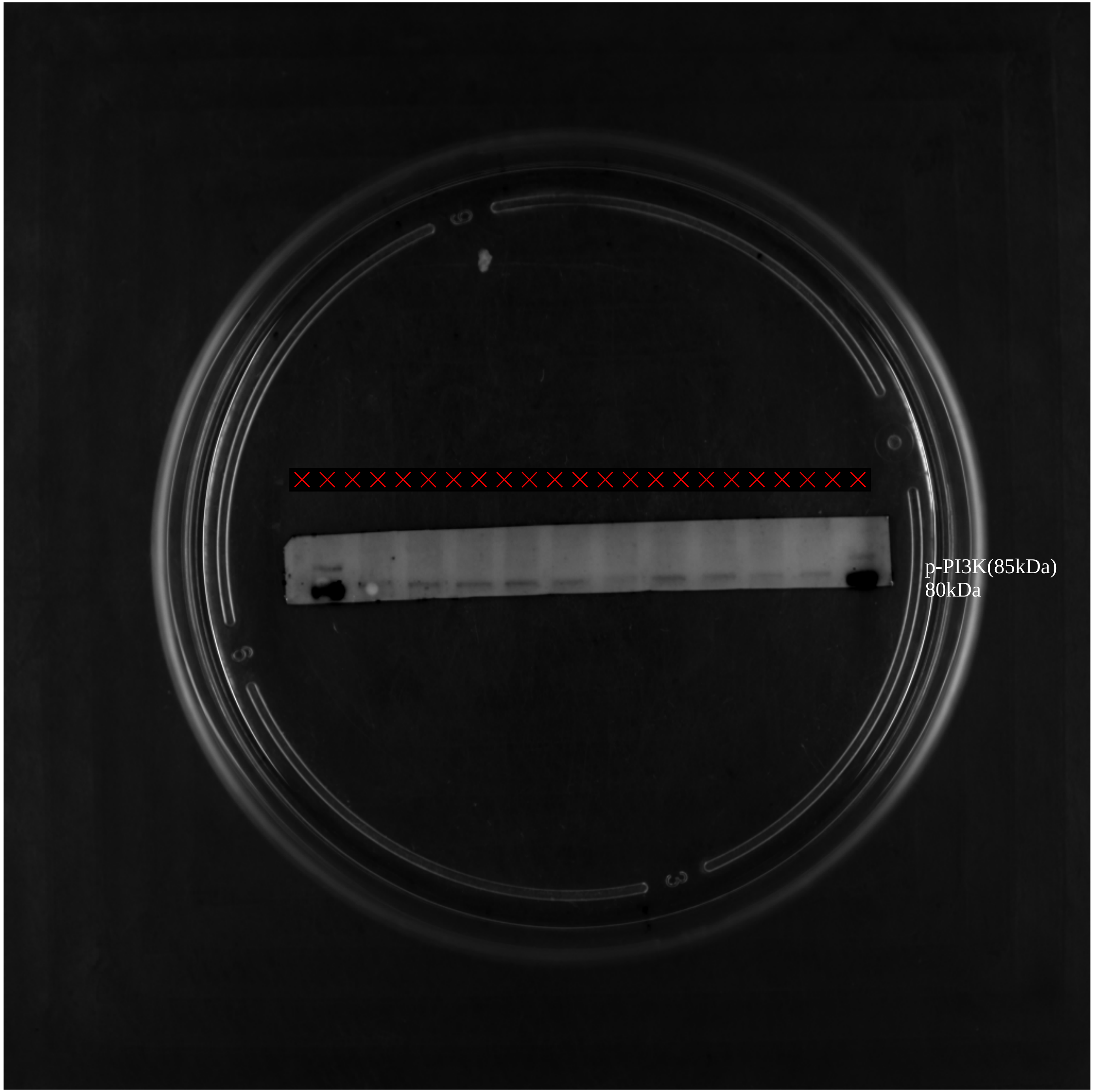

Con Mod NGR1 SSB2 NS Con Mod NGR1 SSB2 NS

p-PI3K-3&4-β-Actin

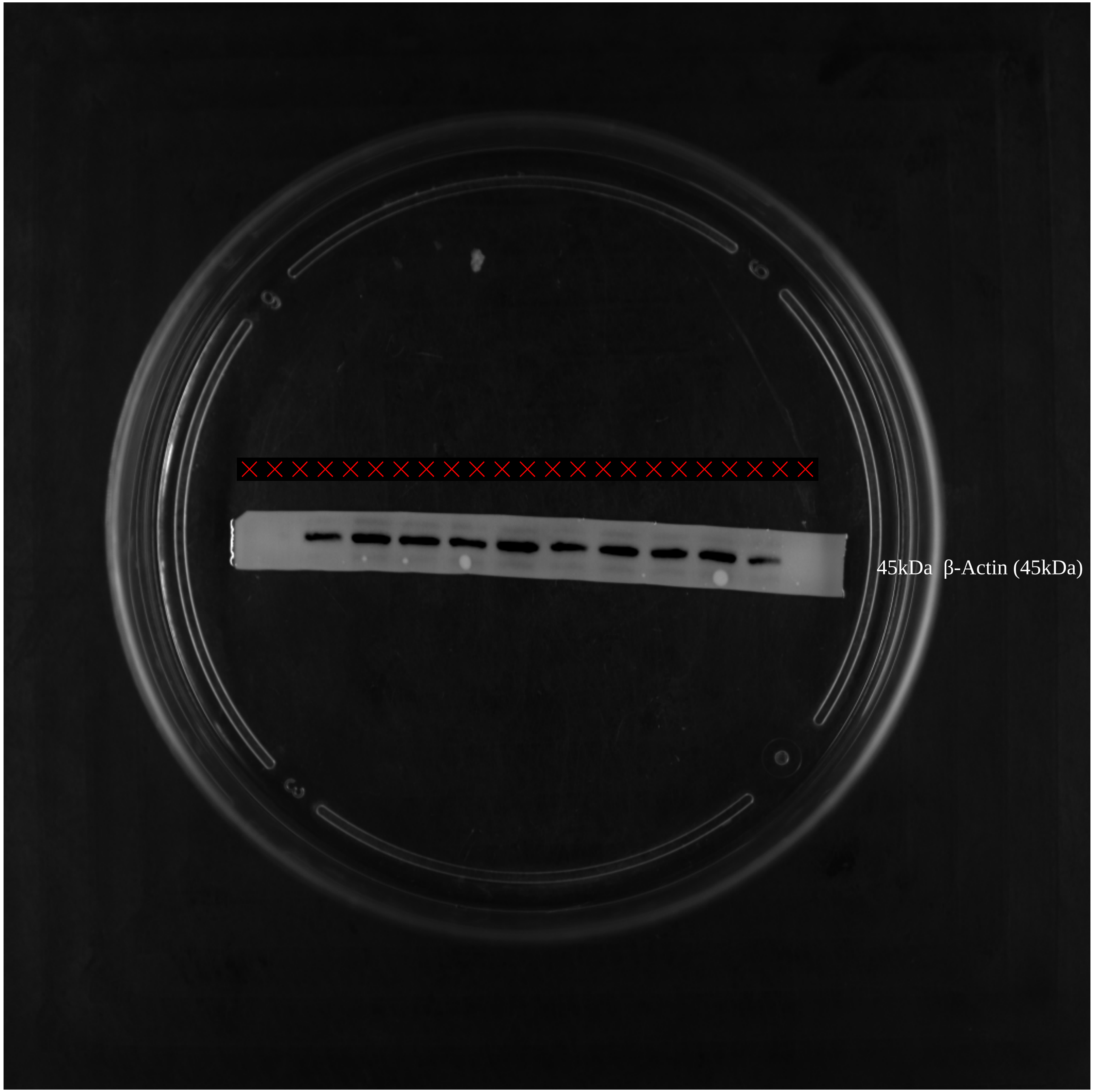

Con Mod NGR1 SSB2 NS Con Mod NGR1 SSB2 NS

PI3K-3&4-full

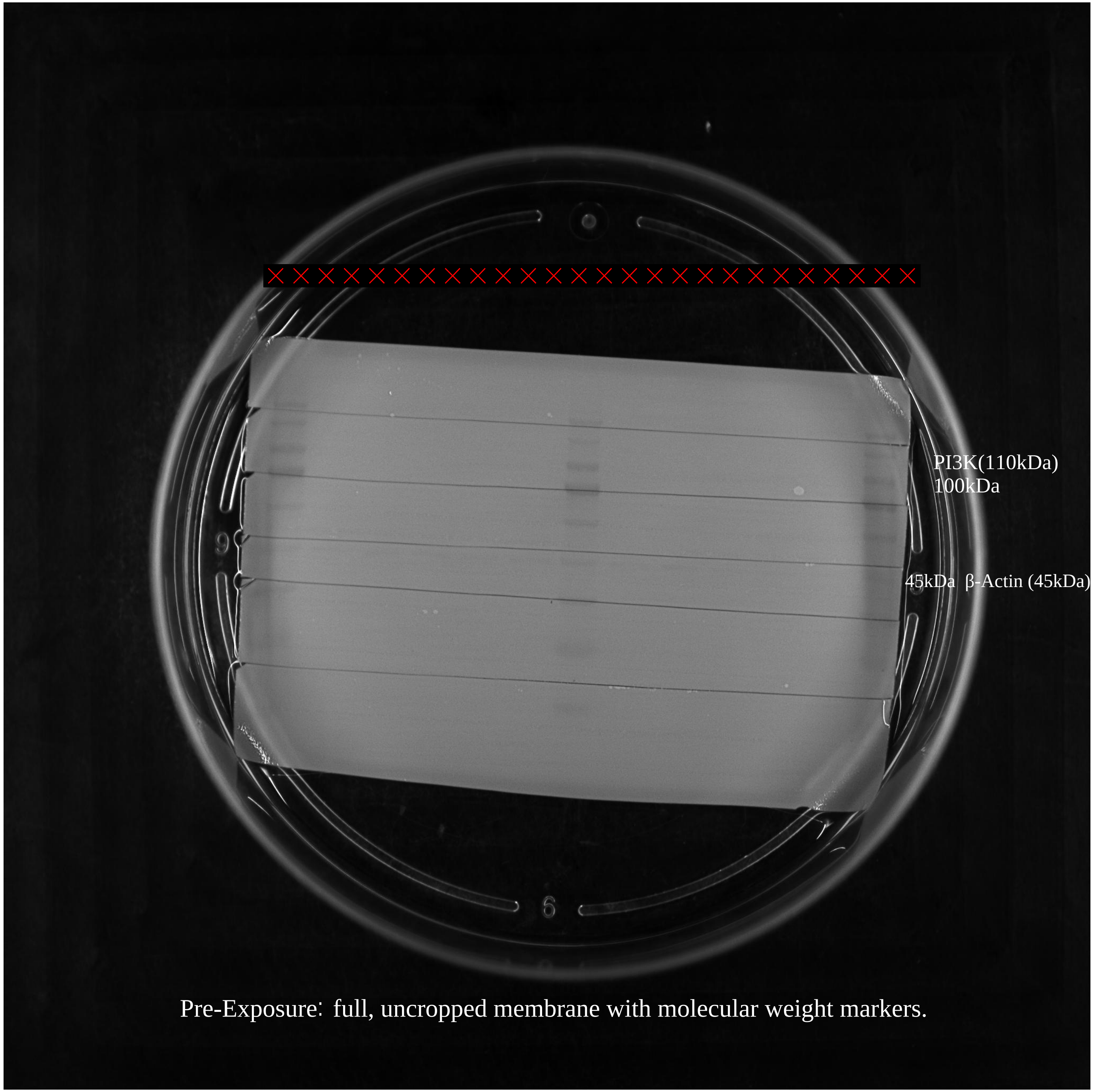

Con Mod NGR1 SSB2 NS Con Mod NGR1 SSB2 NS

PI3K-3&4

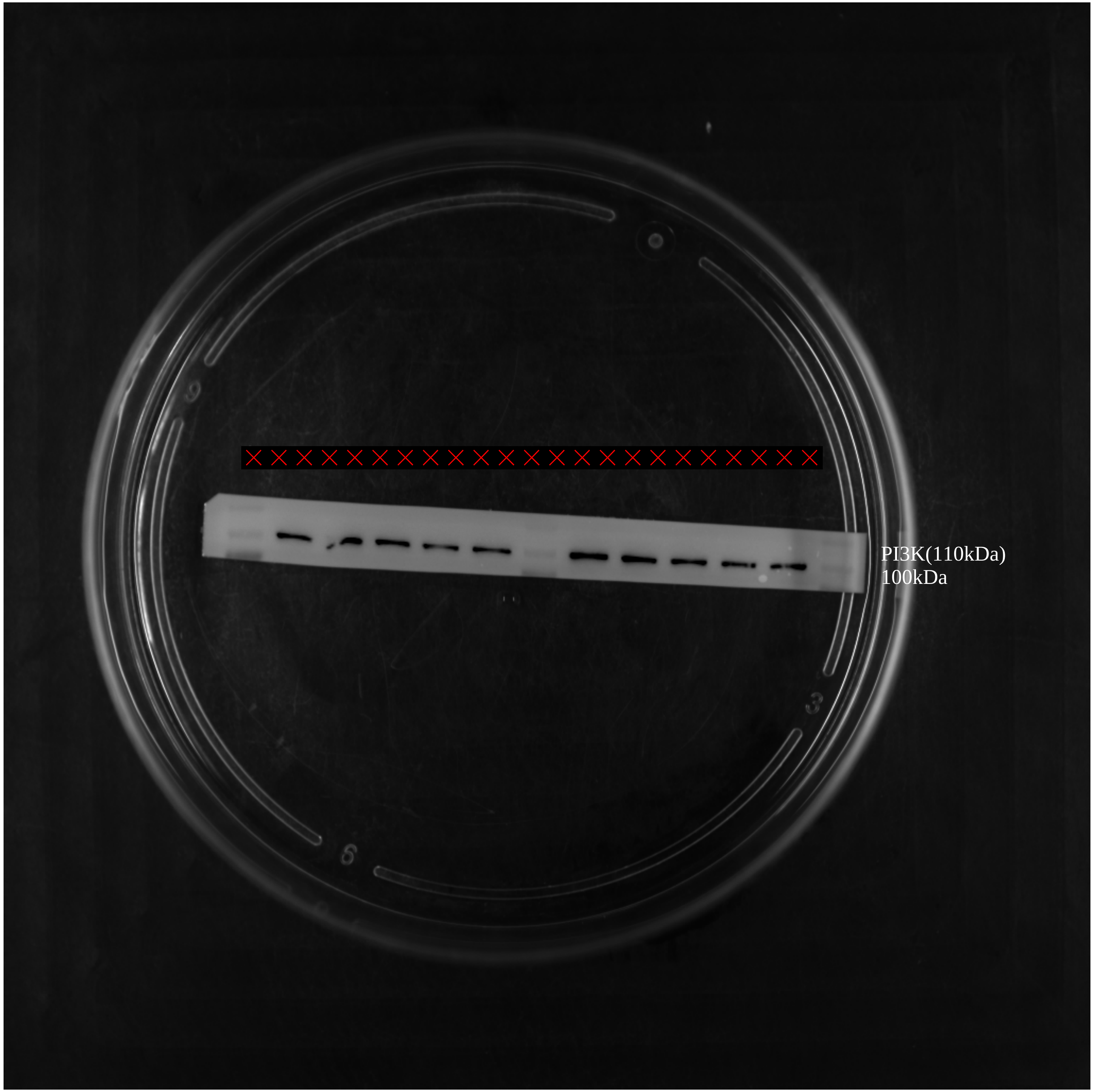

Con Mod NGR1 SSB2 NS Con Mod NGR1 SSB2 NS

PI3K-3&4-β-Actin

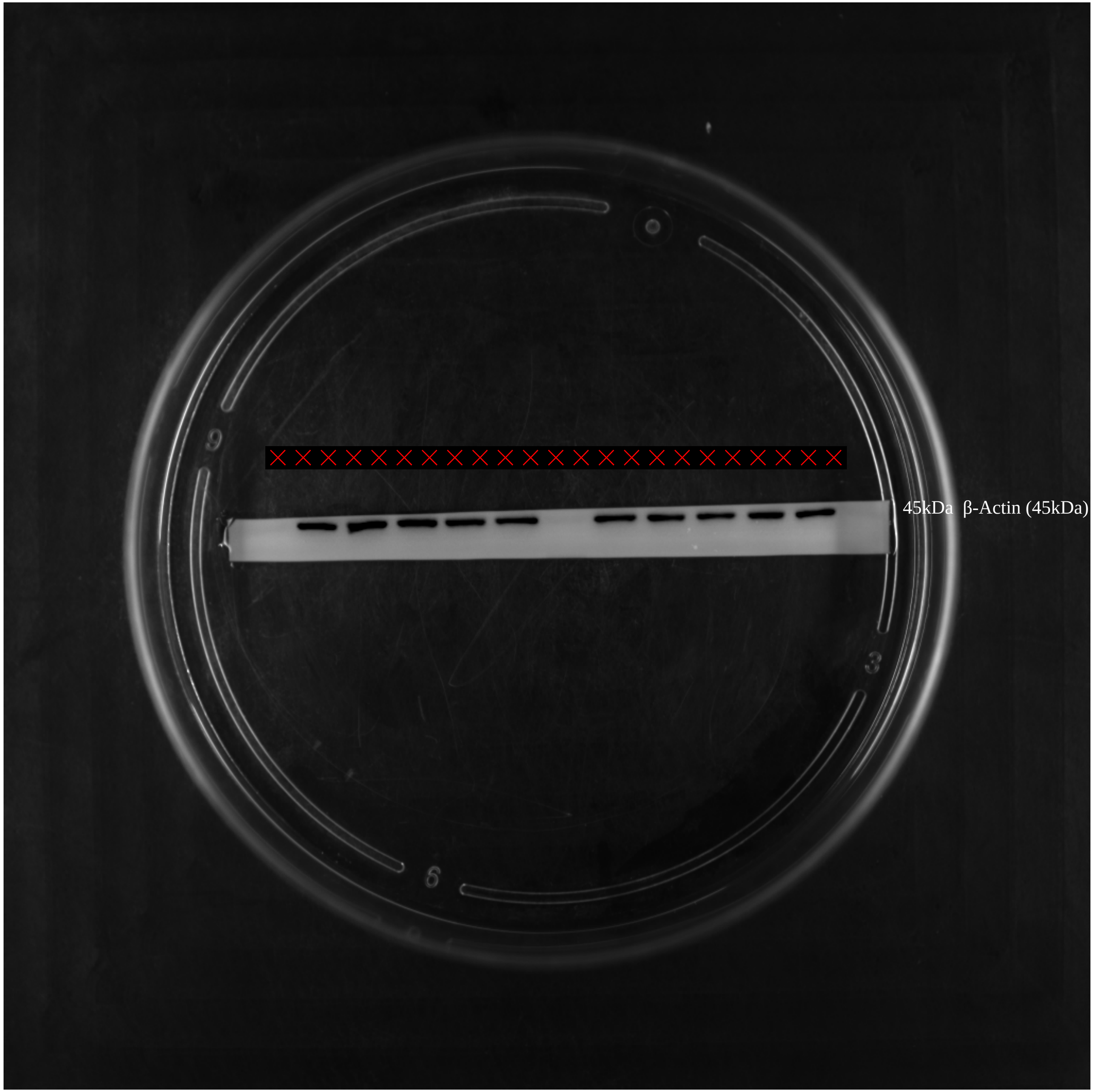

Con Mod NGR1 SSB2 NS Con Mod NGR1 SSB2 NS

p-AKT-2-full

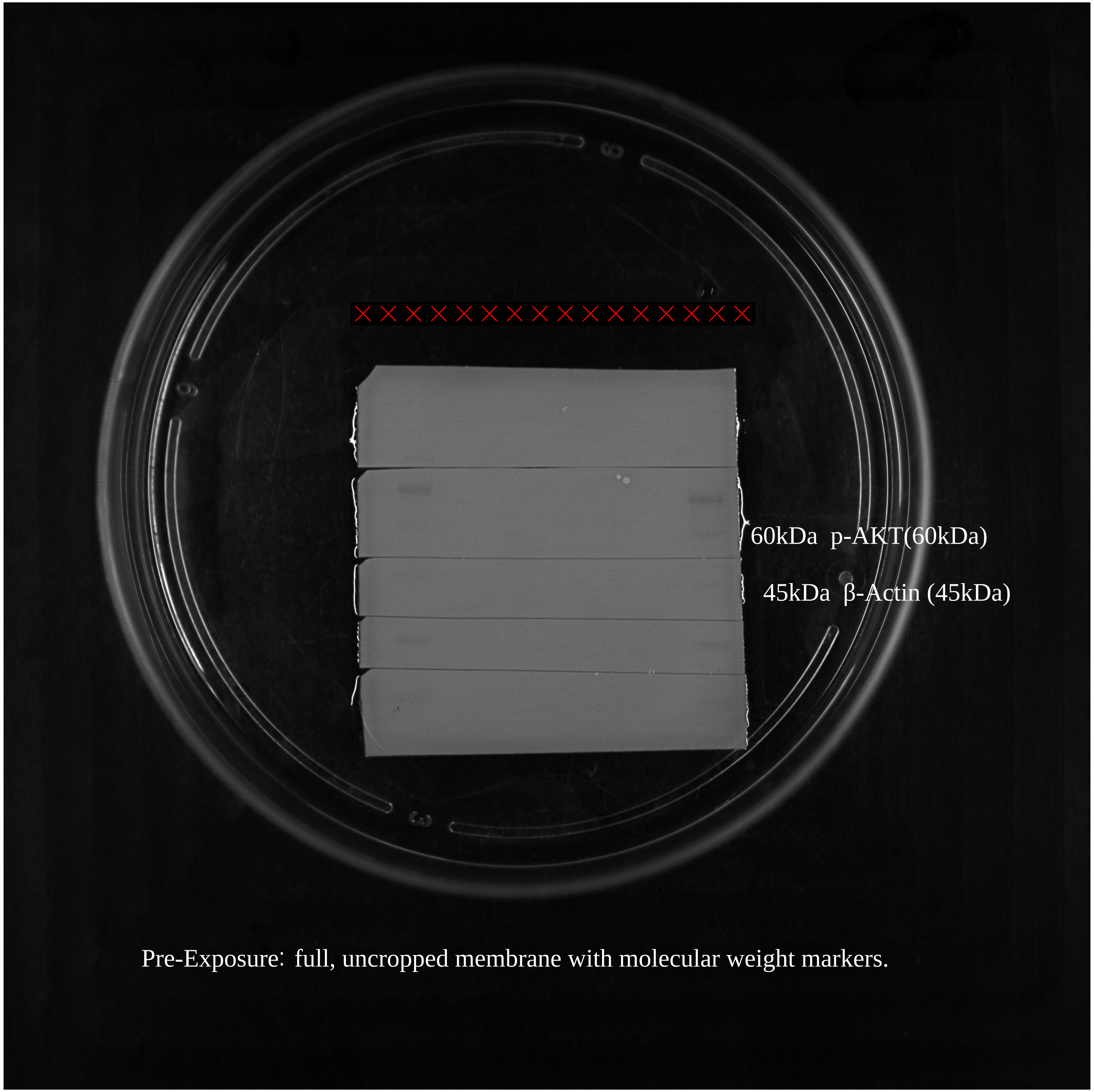

Con Mod NGR1 SSB2 NS

p-AKT-2

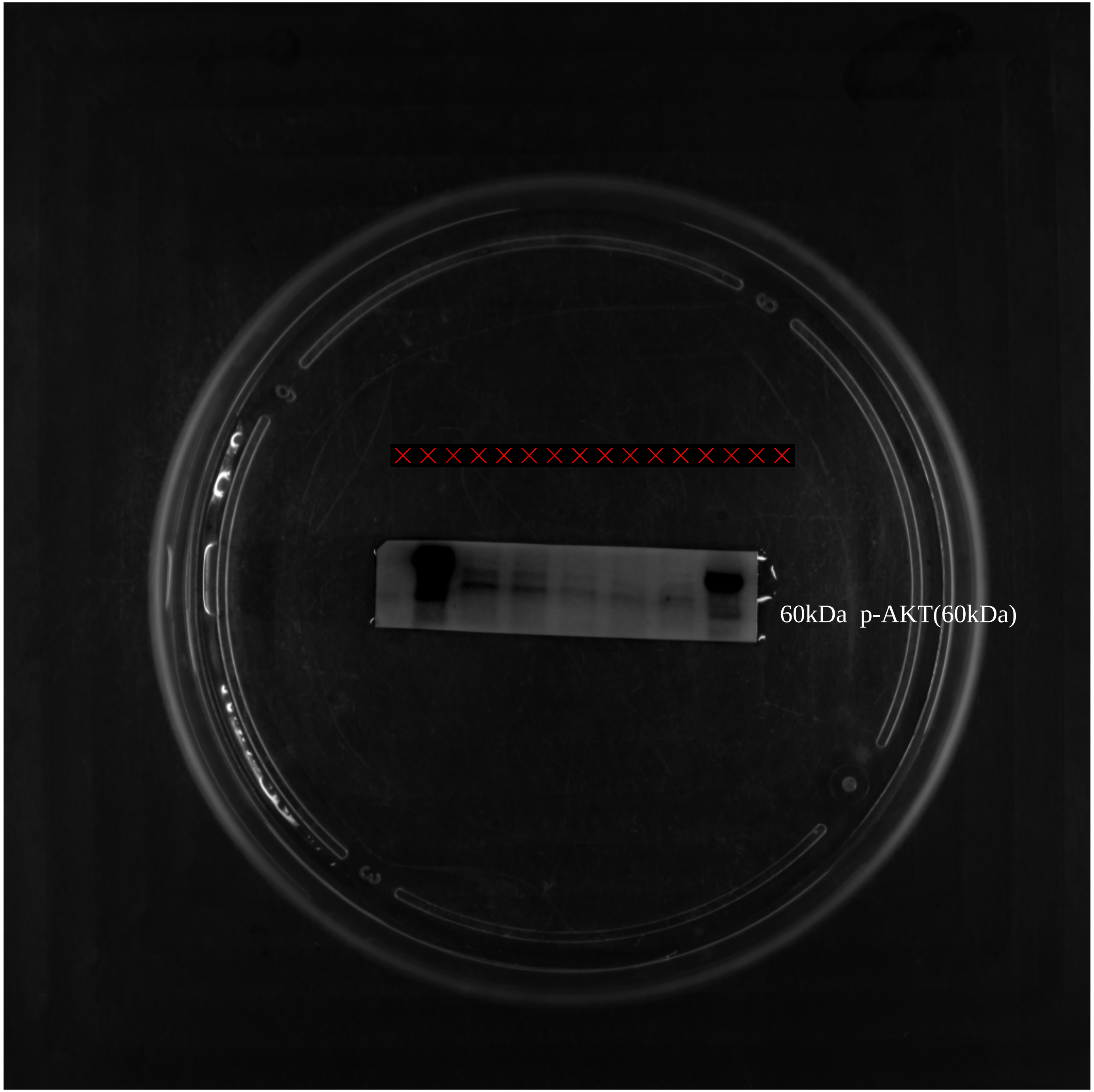

Con Mod NGR1 SSB2 NS

p-AKT-2-β-Actin

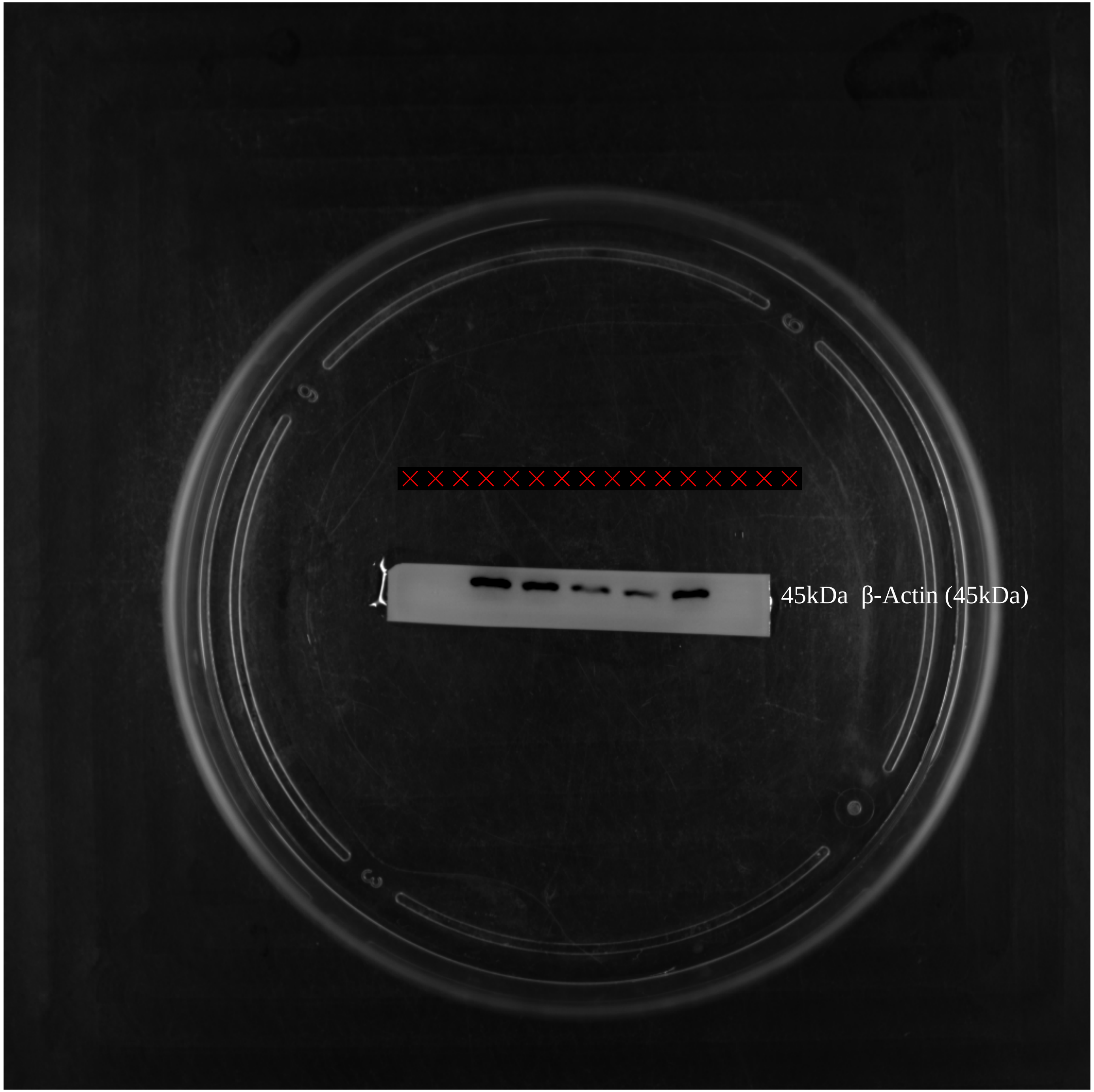

Con Mod NGR1 SSB2 NS

p-AKT-3&4-full

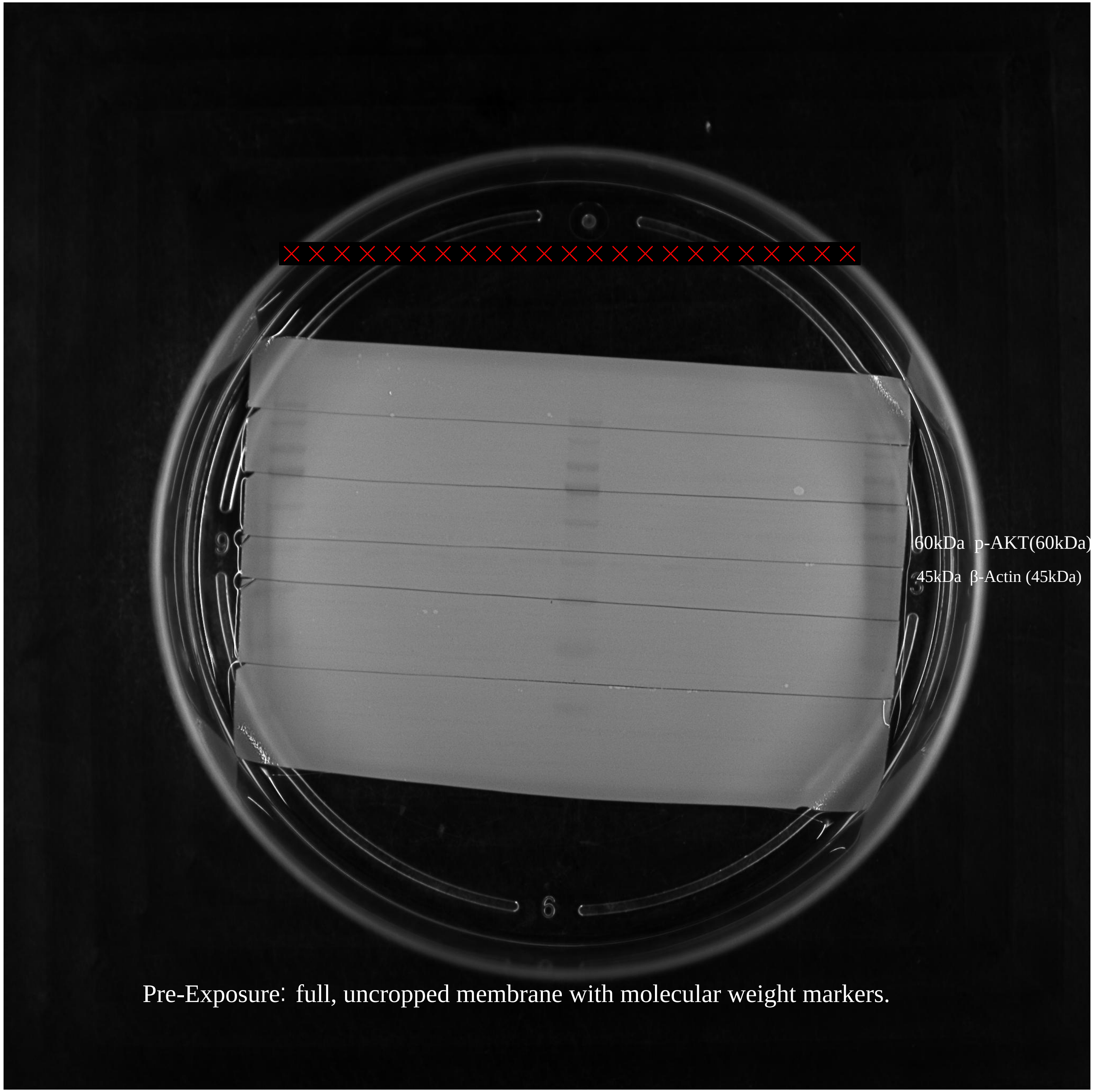

Con Mod NGR1 SSB2 NS Con Mod NGR1 SSB2 NS

p-AKT-3&4

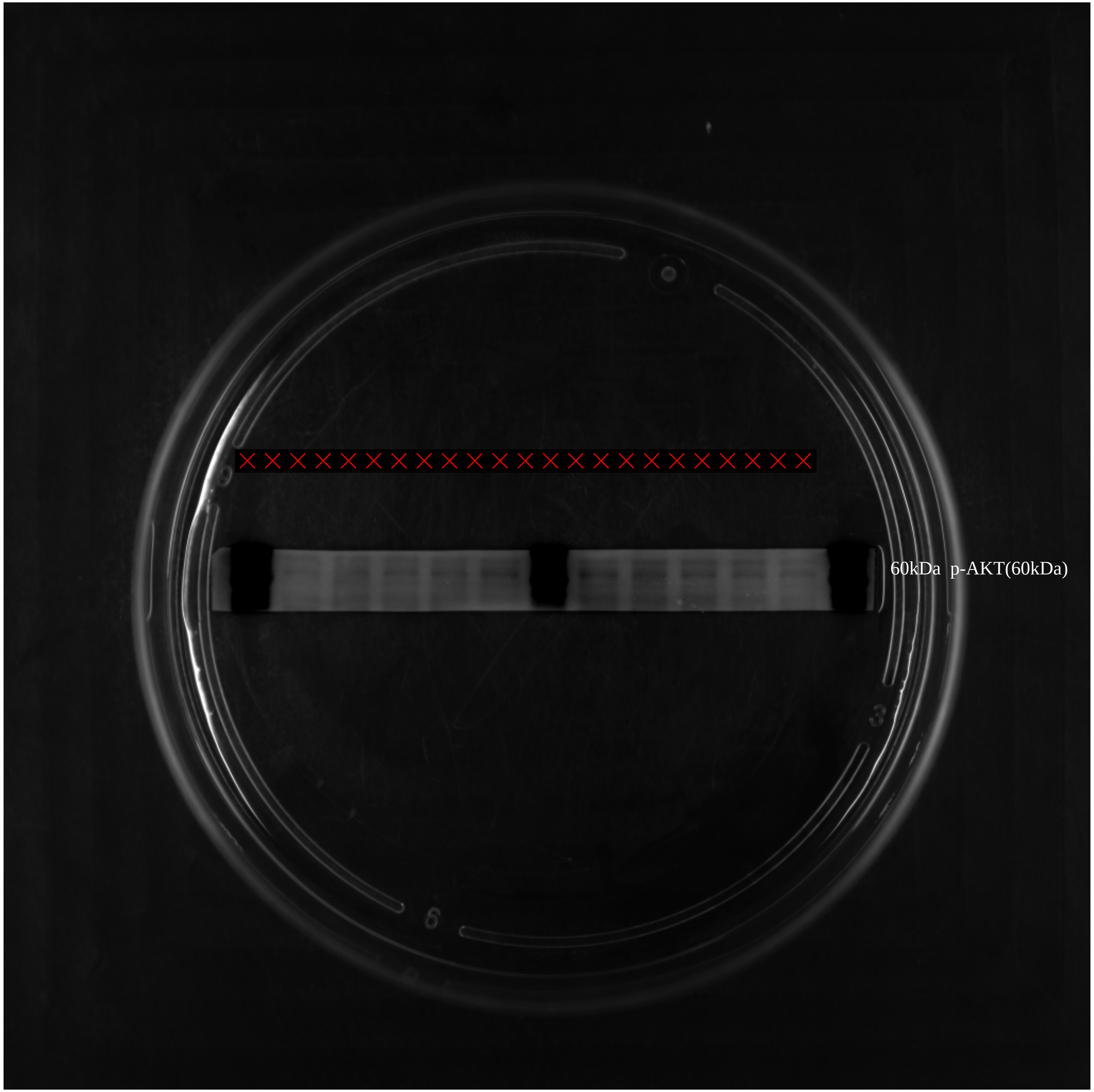

Con Mod NGR1 SSB2 NS Con Mod NGR1 SSB2 NS

p-AKT-3&4-β-Actin

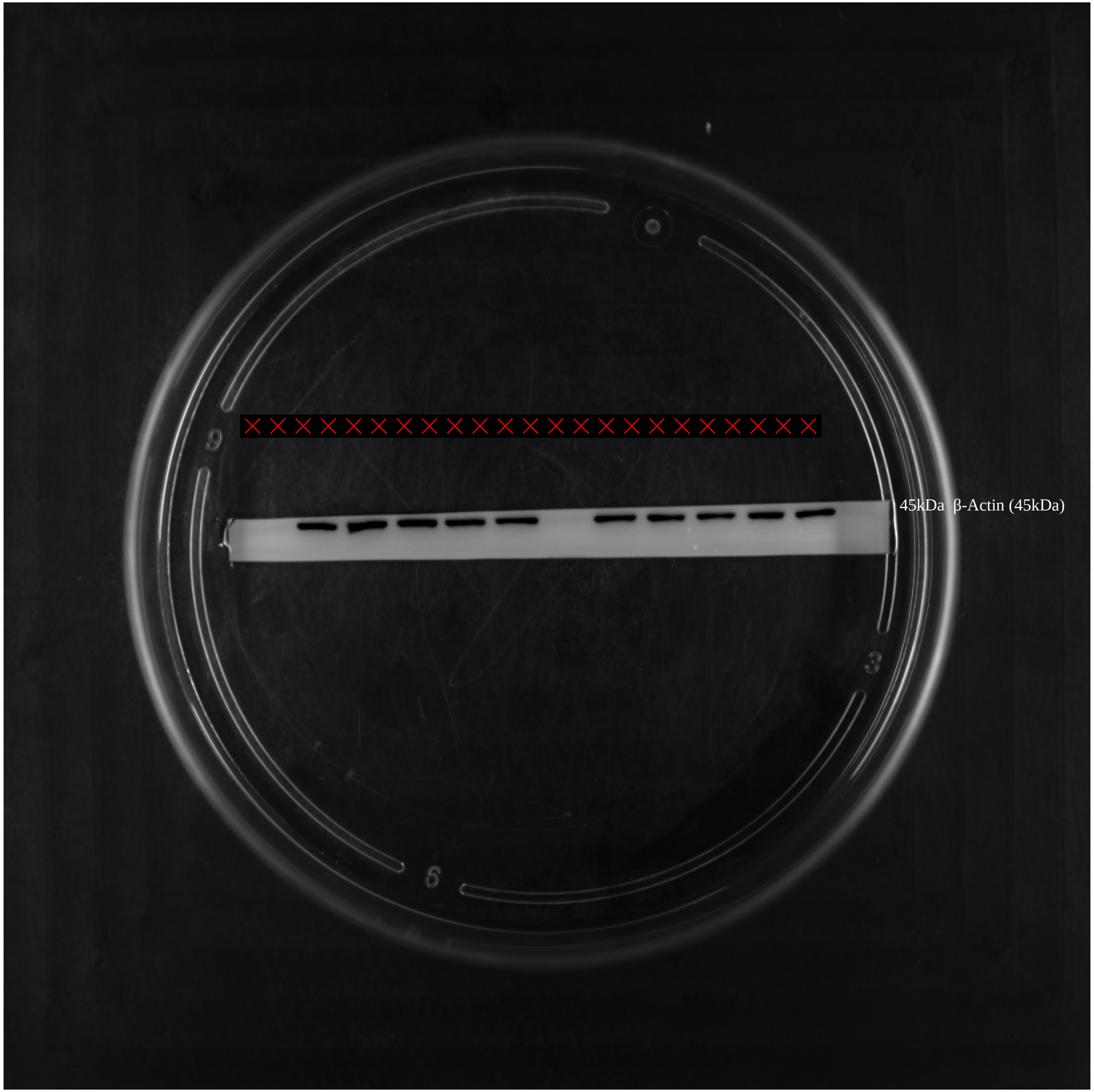

Con Mod NGR1 SSB2 NS Con Mod NGR1 SSB2 NS

AKT-3&4-full

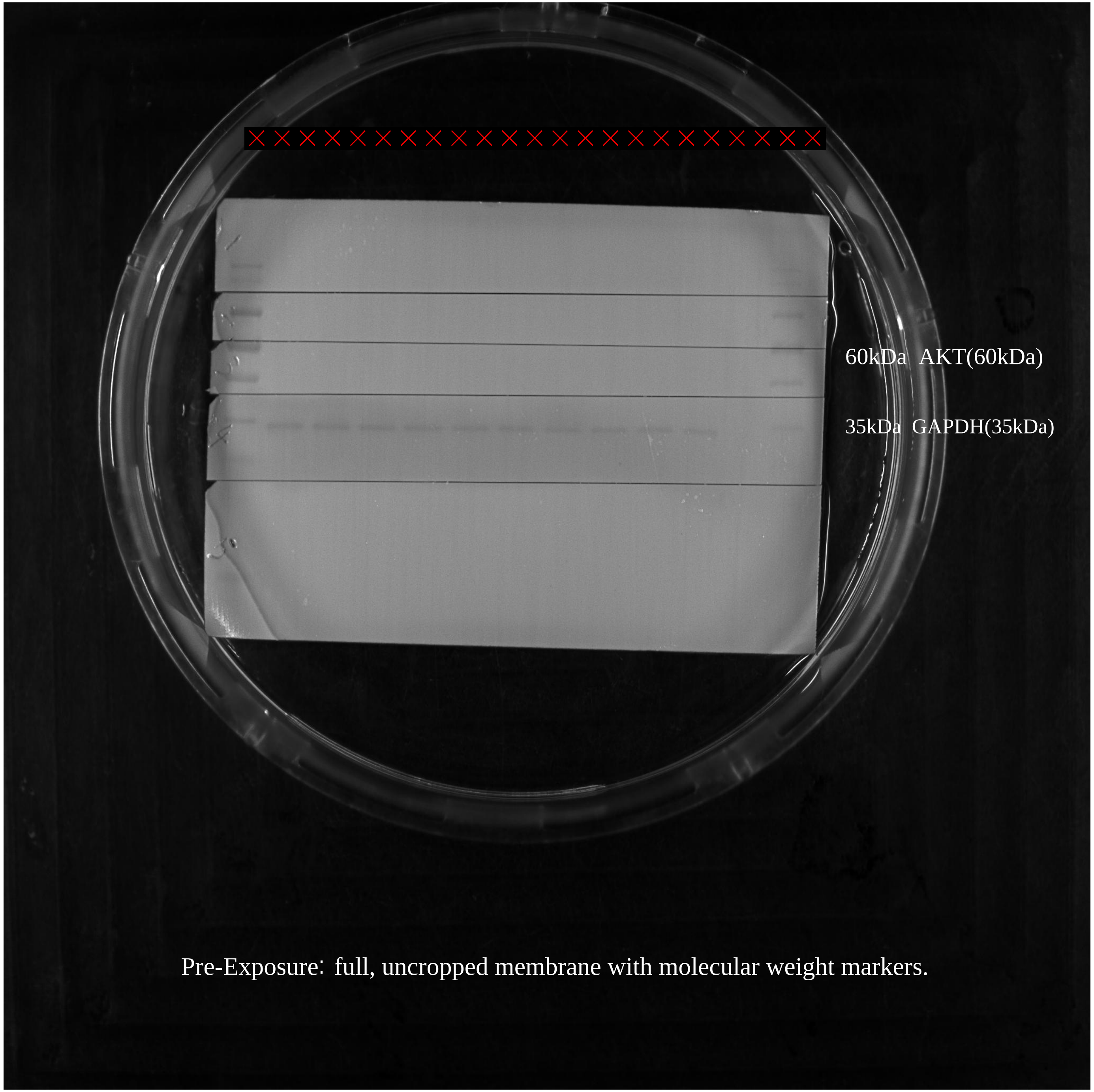

Con Mod NGR1 SSB2 NS Con Mod NGR1 SSB2 NS

AKT-3&4-GAPDH

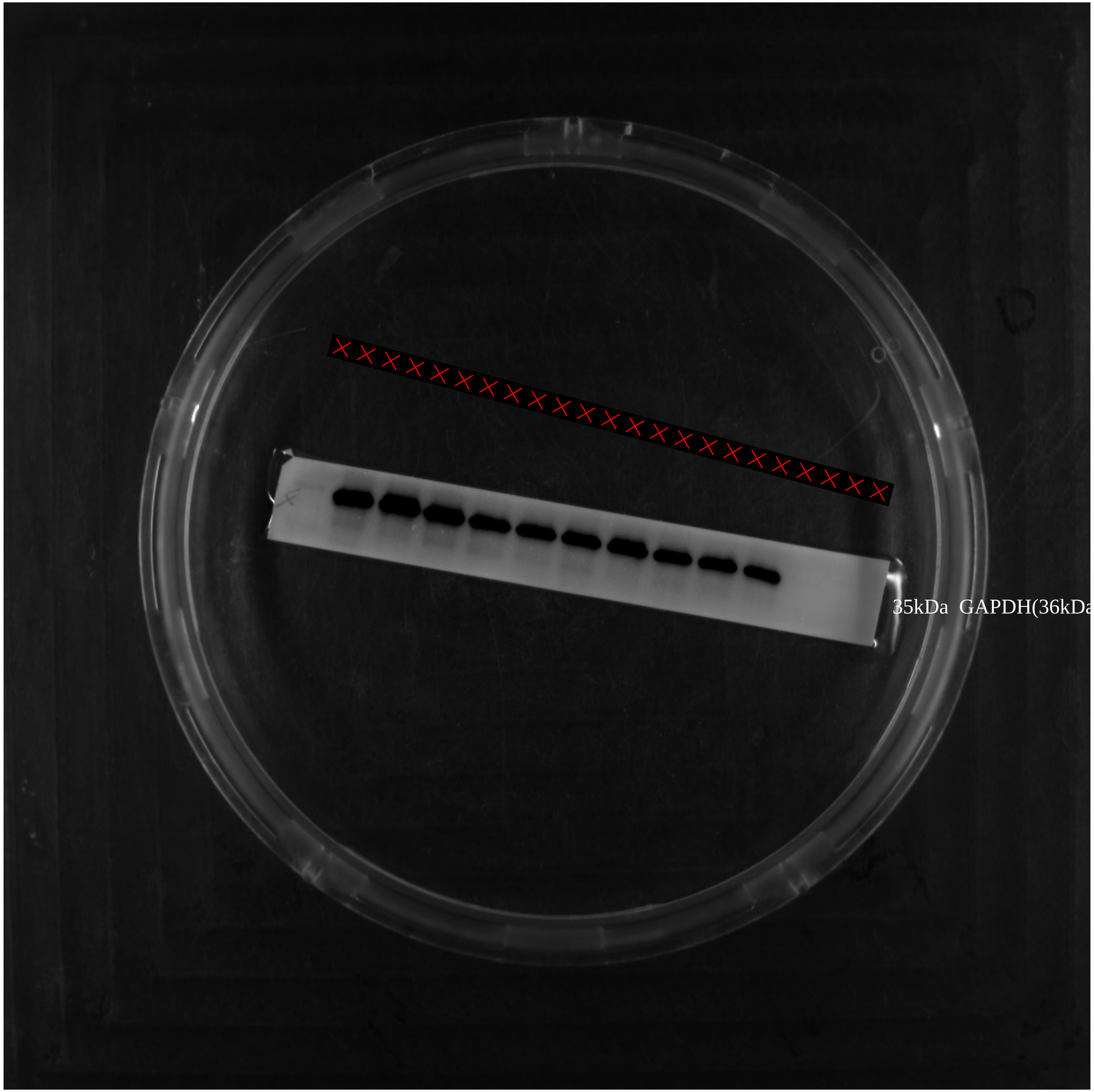

Con Mod NGR1 SSB2 NS Con Mod NGR1 SSB2 NS

AKT-3&4

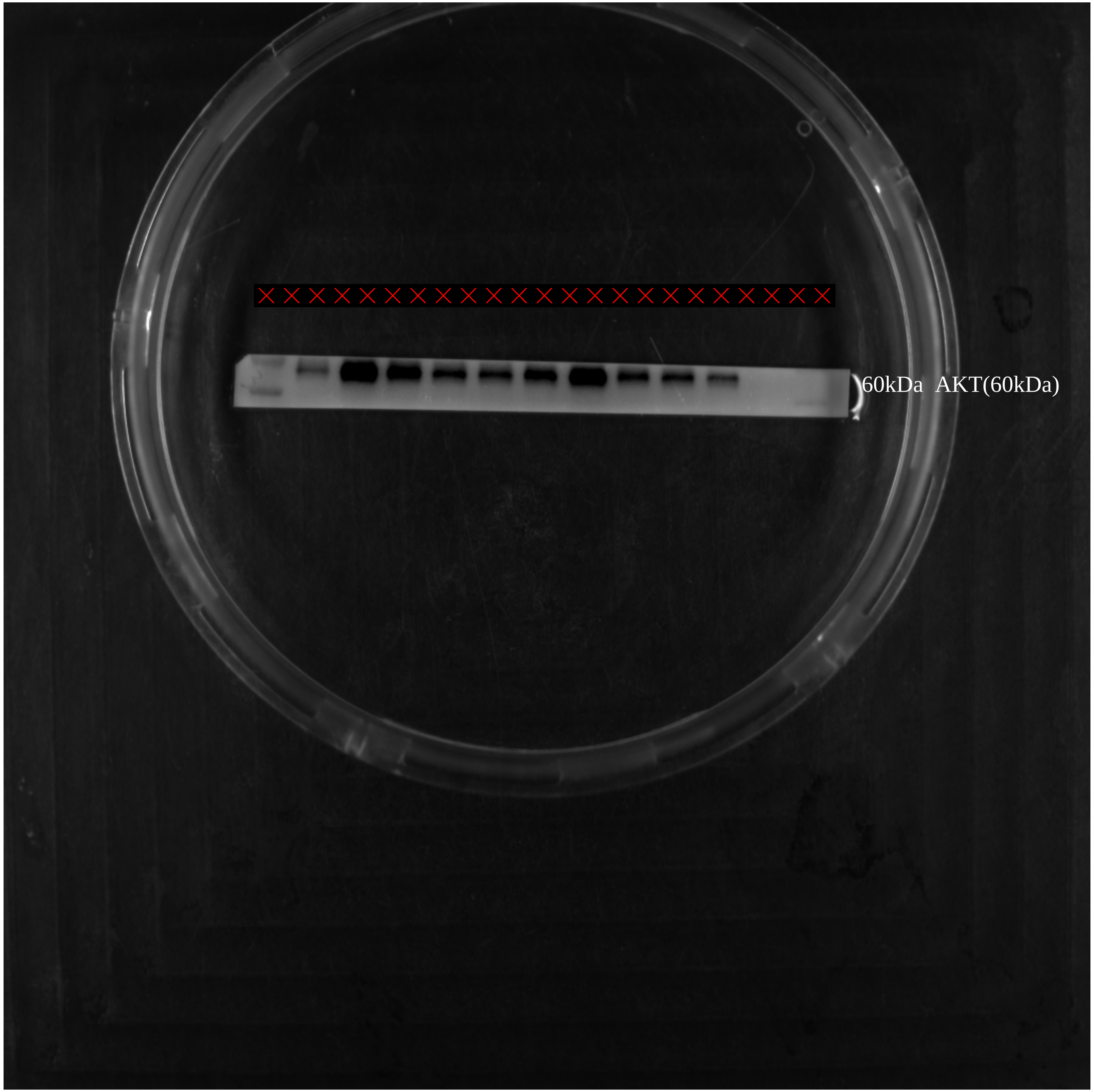

Con Mod NGR1 SSB2 NS Con Mod NGR1 SSB2 NS

p-mTOR-3-full

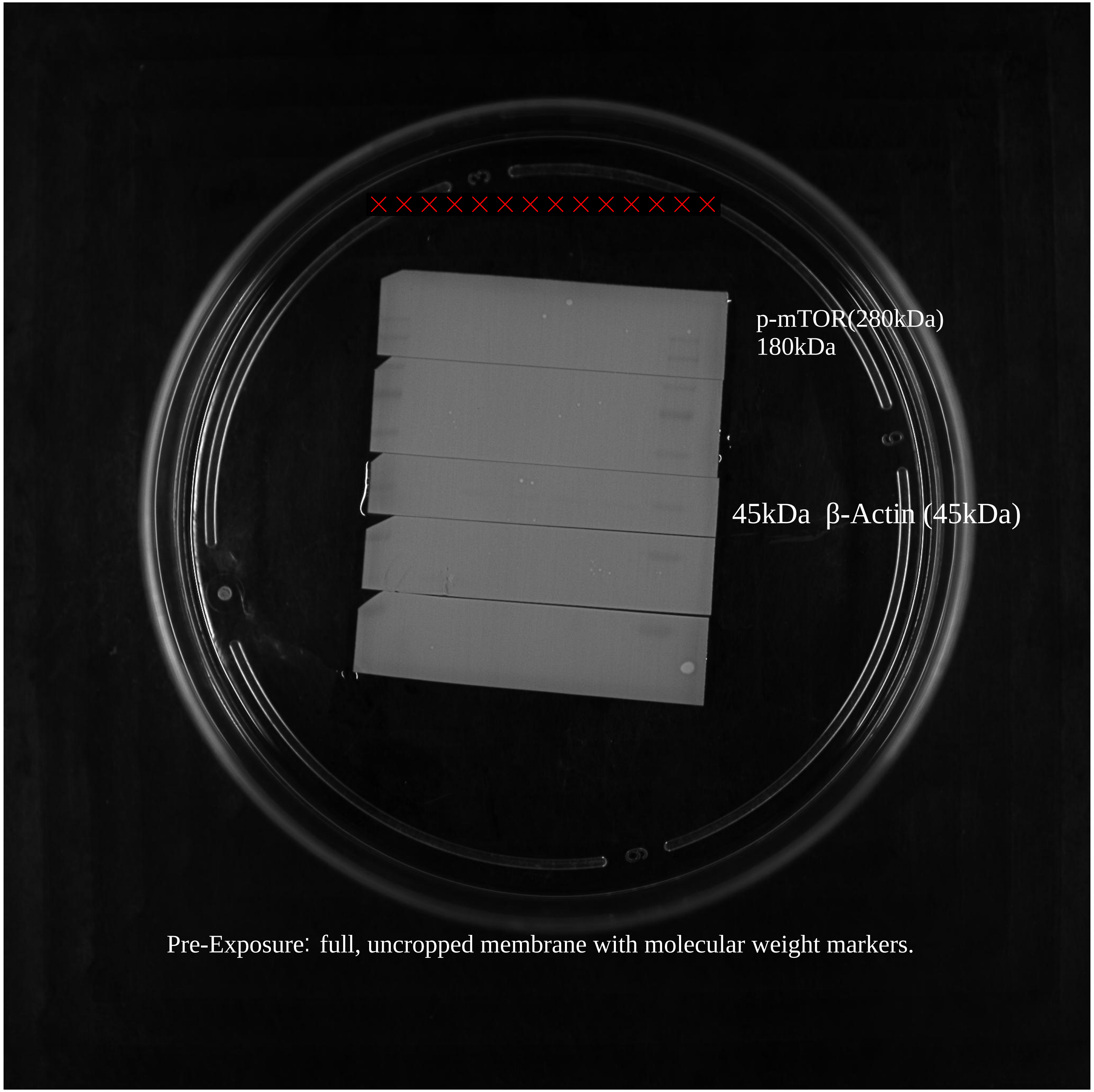

Con Mod NGR1 SSB2 NS

p-mTOR-3

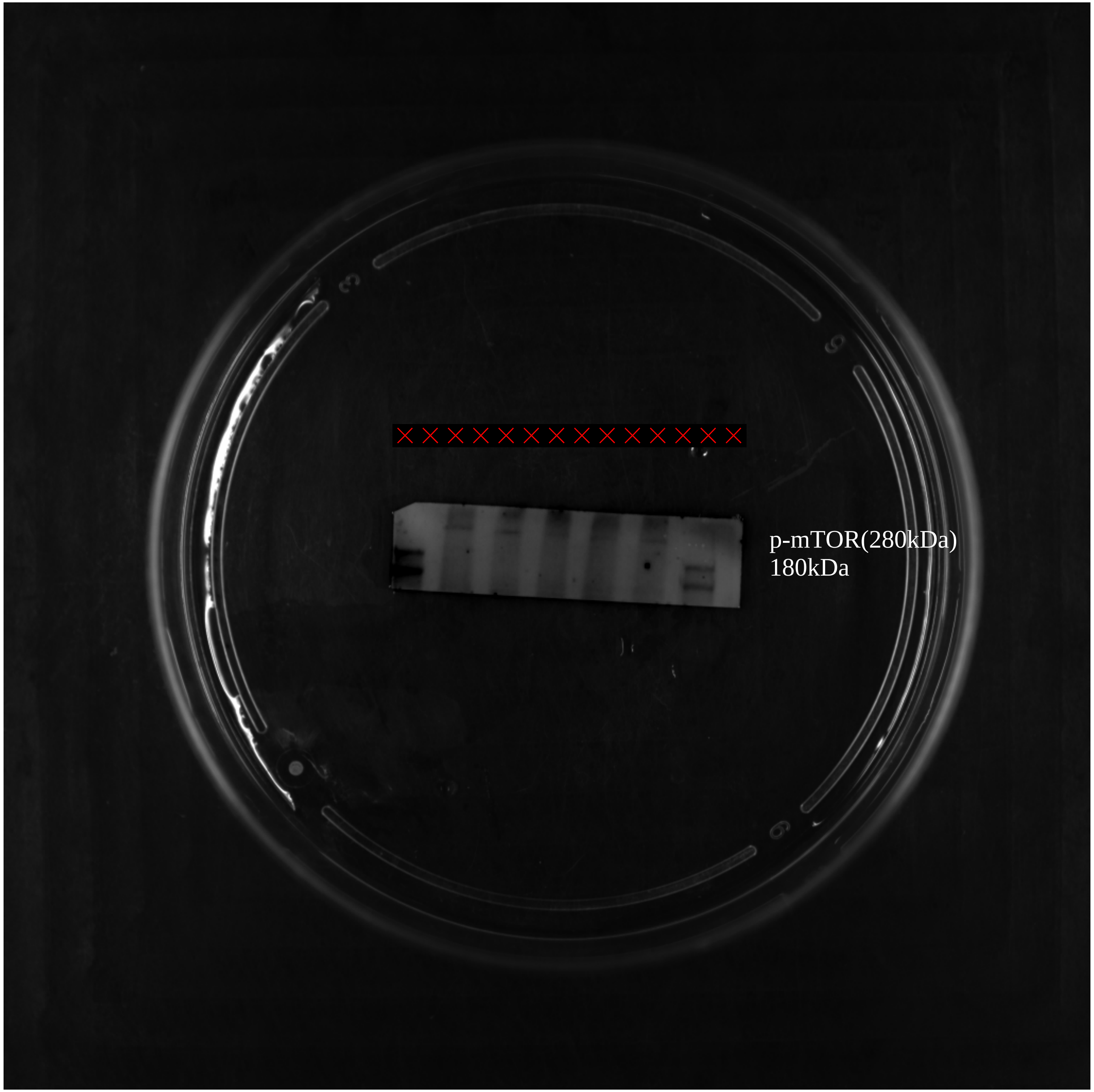

Con Mod NGR1 SSB2 NS

p-mTOR-3-β-Actin

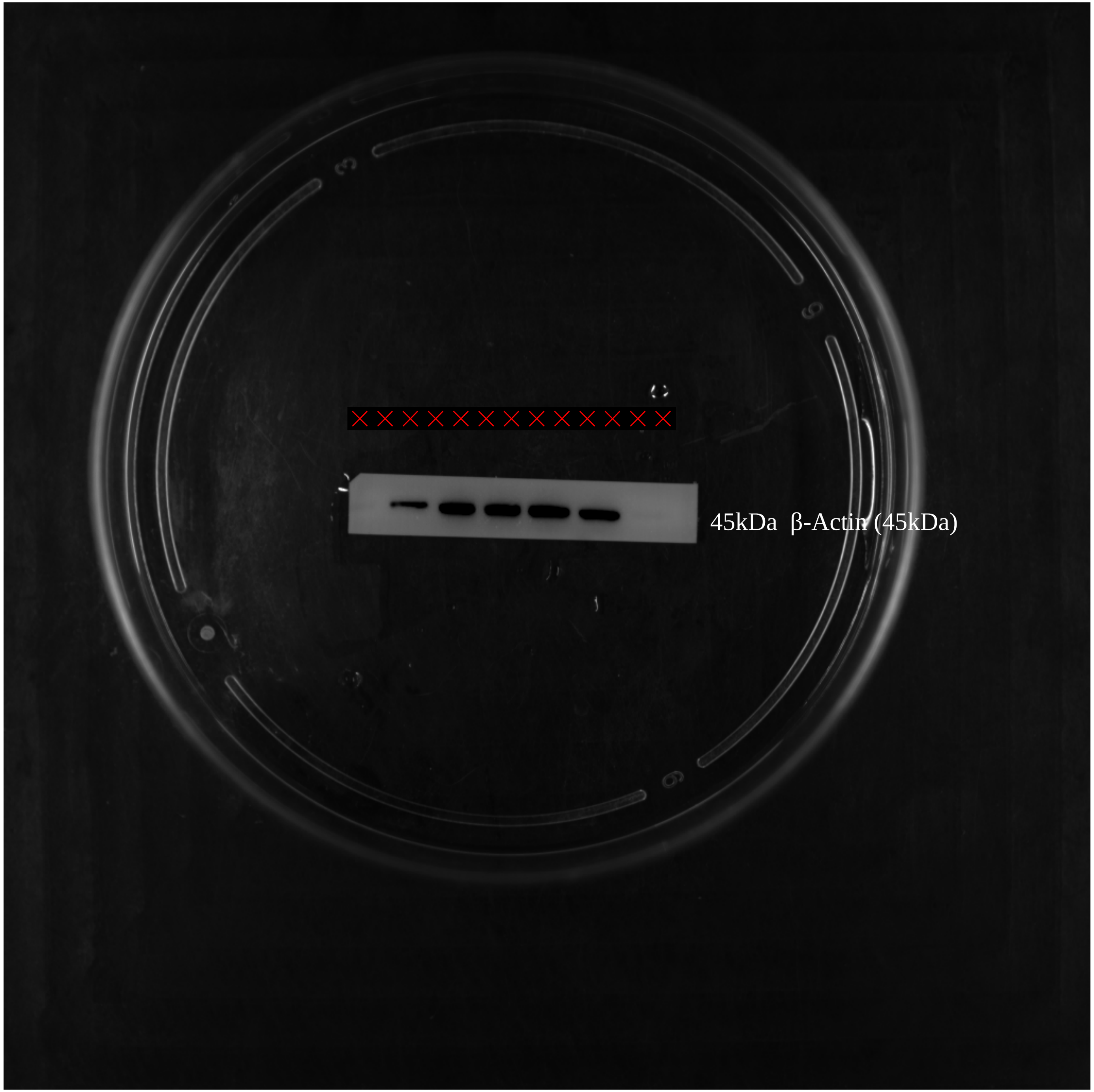

Con Mod NGR1 SSB2 NS

mTOR-3&4-full

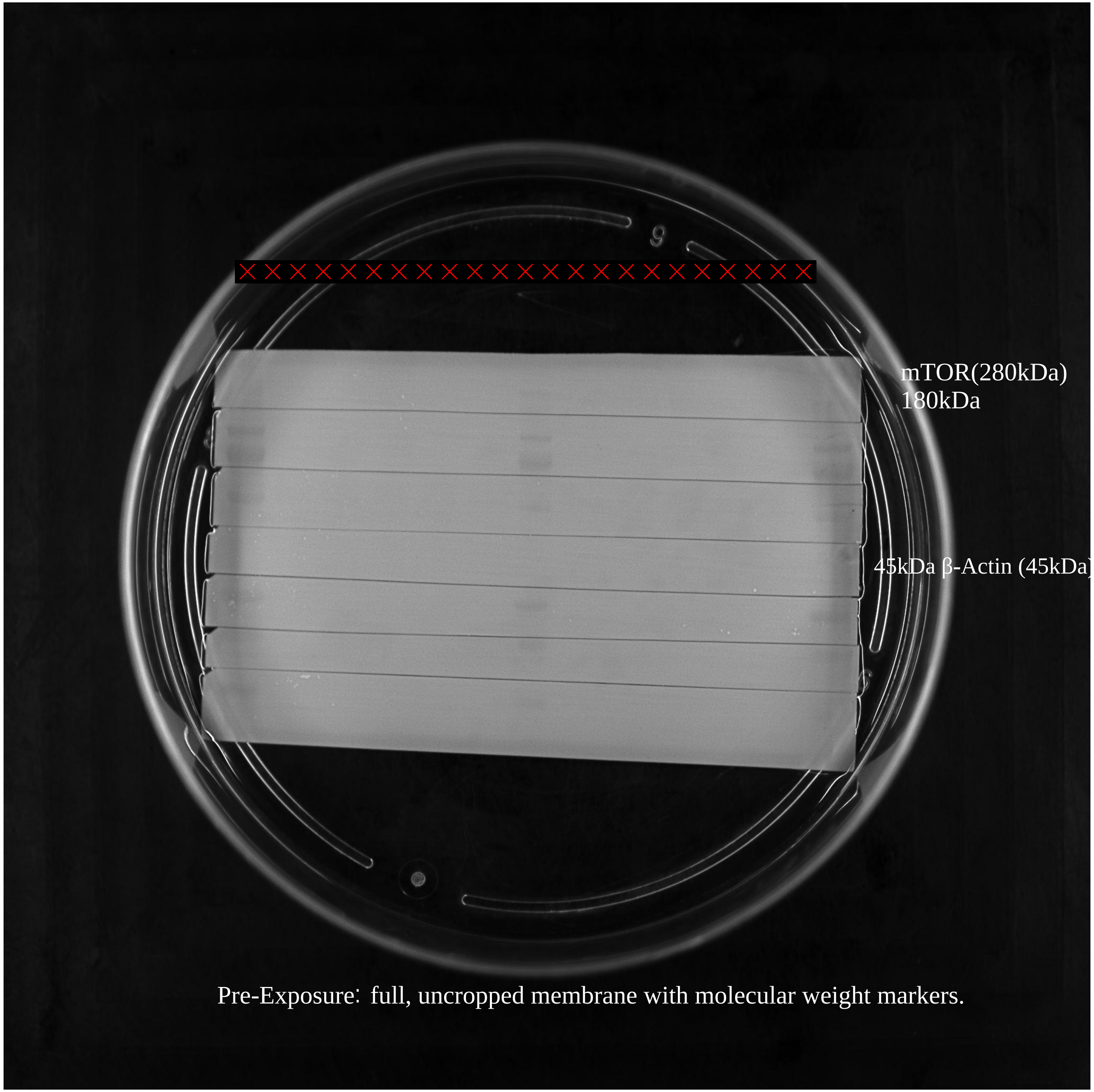

Con Mod NGR1 SSB2 NS Con Mod NGR1 SSB2 NS

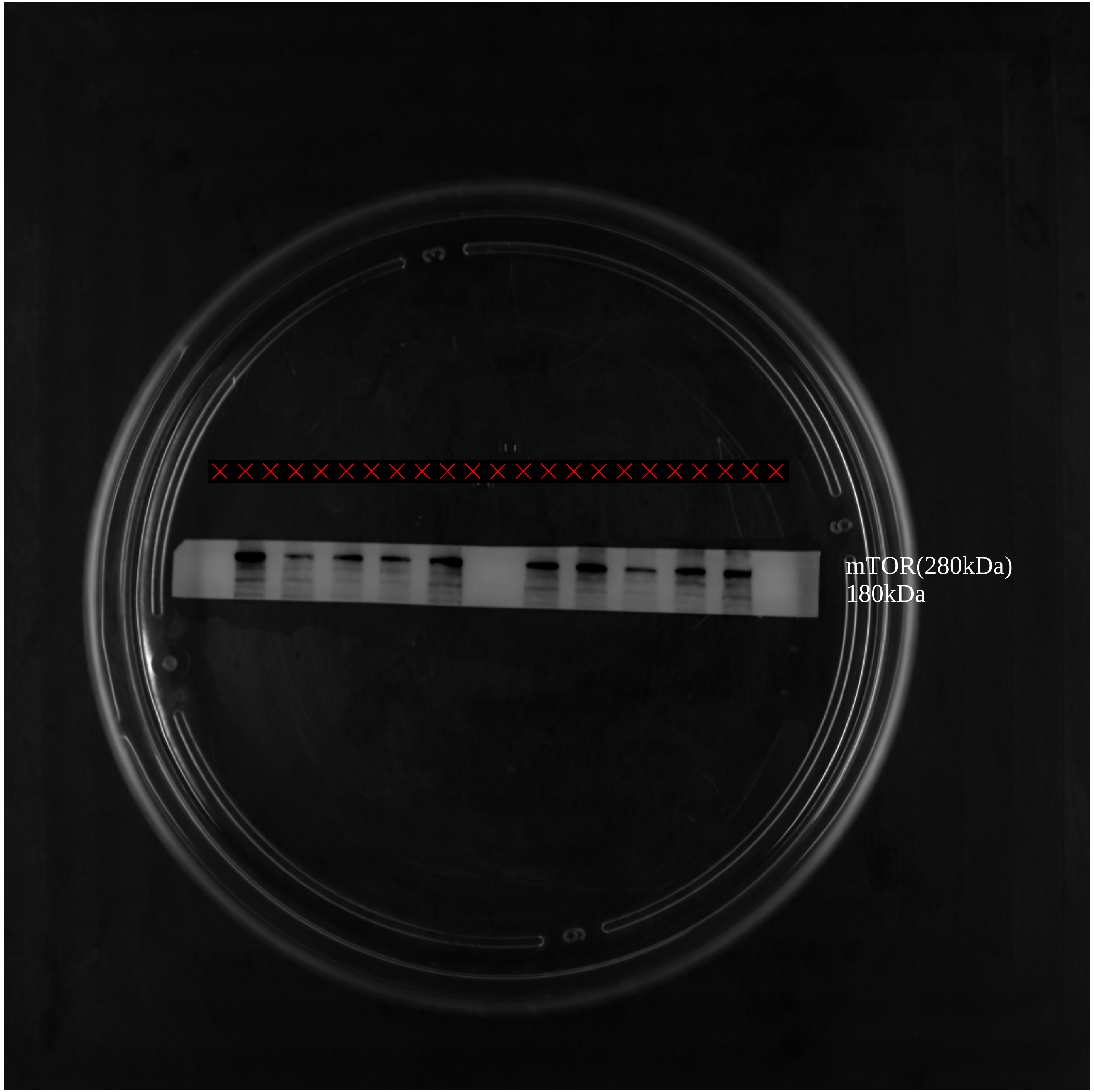

Con Mod NGR1 SSB2 NS Con Mod NGR1 SSB2 NS

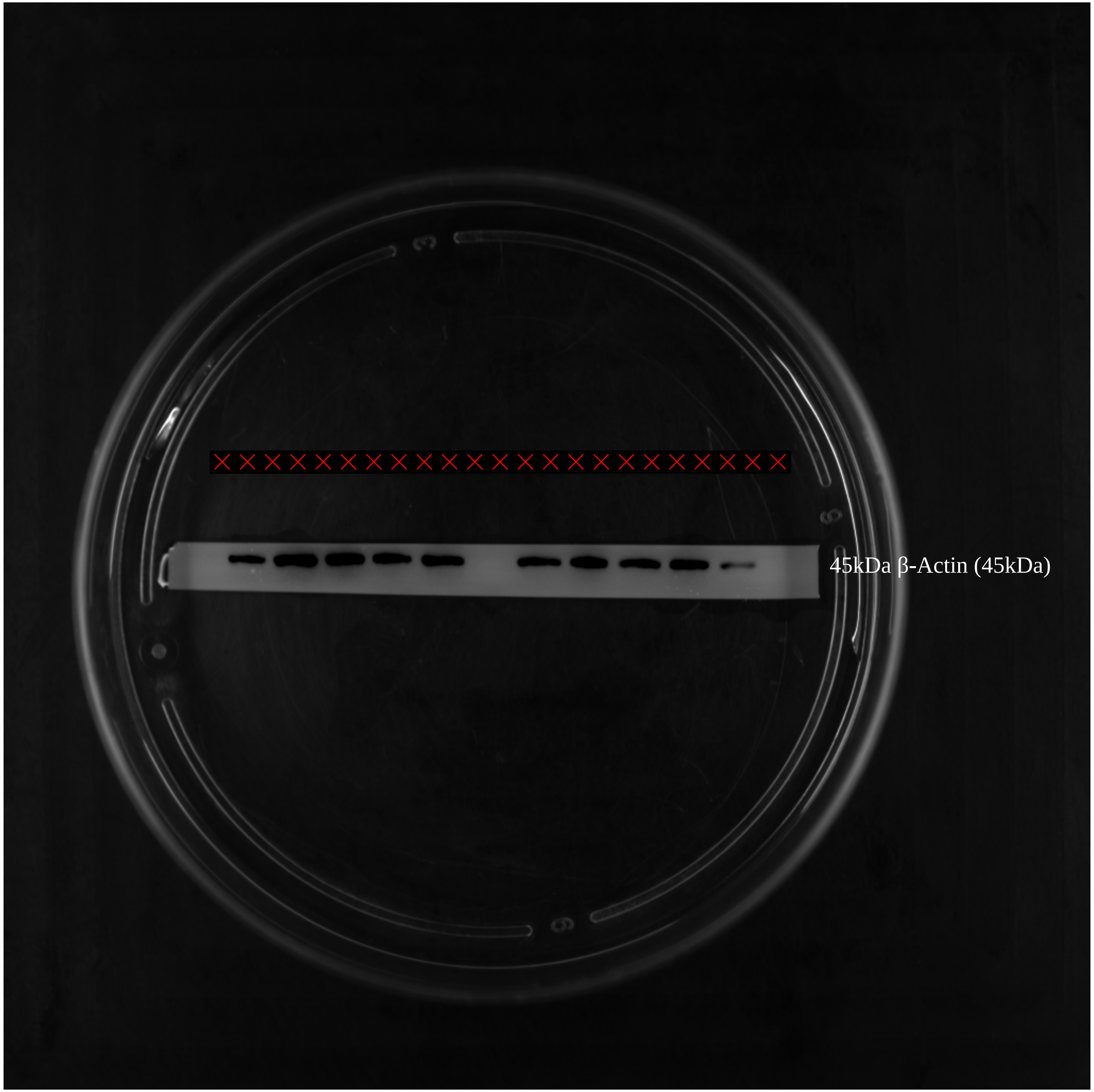

Con Mod NGR1 SSB2 NS Con Mod NGR1 SSB2 NS

Beclin1-2-full

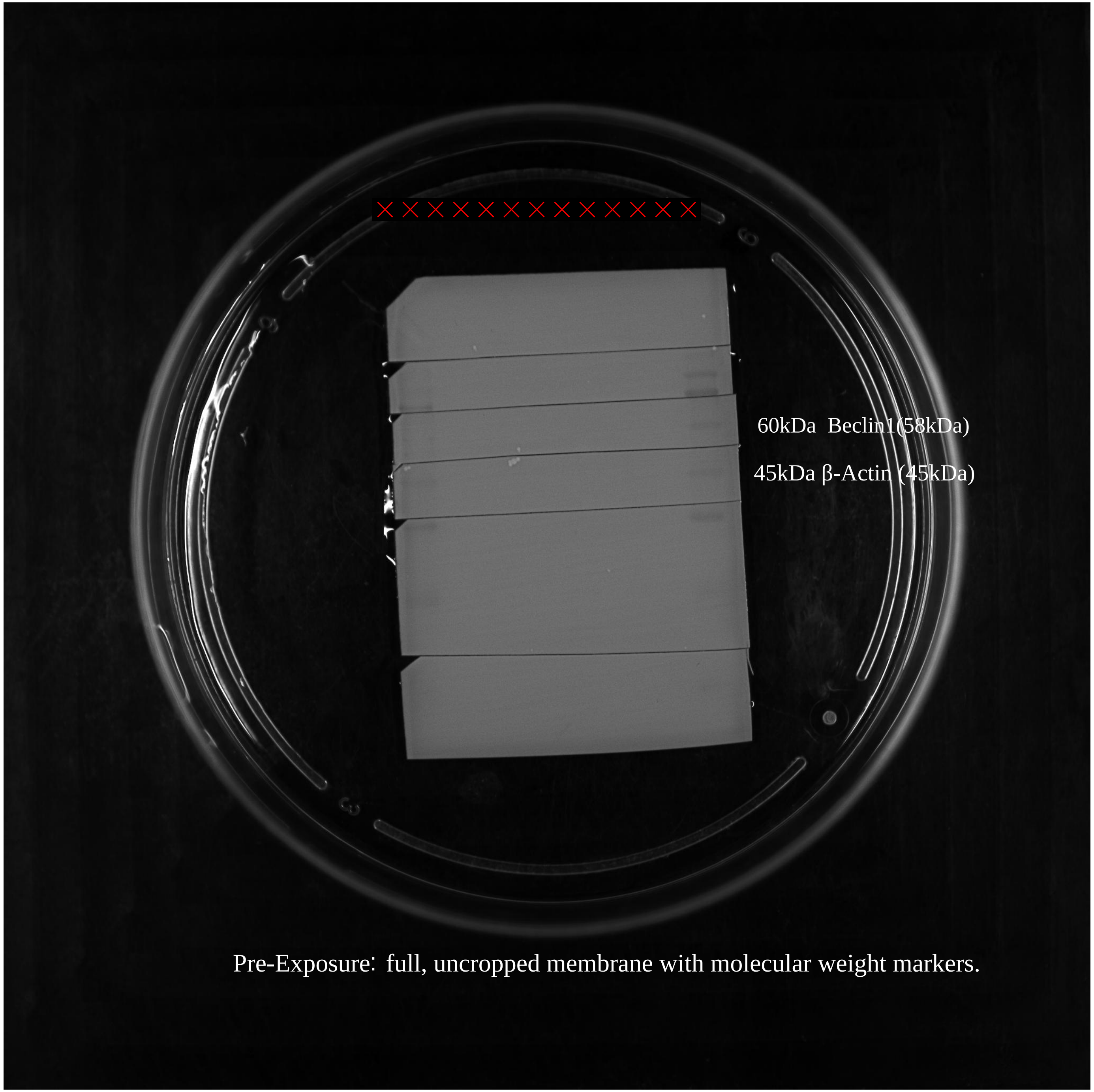

Con Mod NGR1 SSB2 NS

Beclin1-2

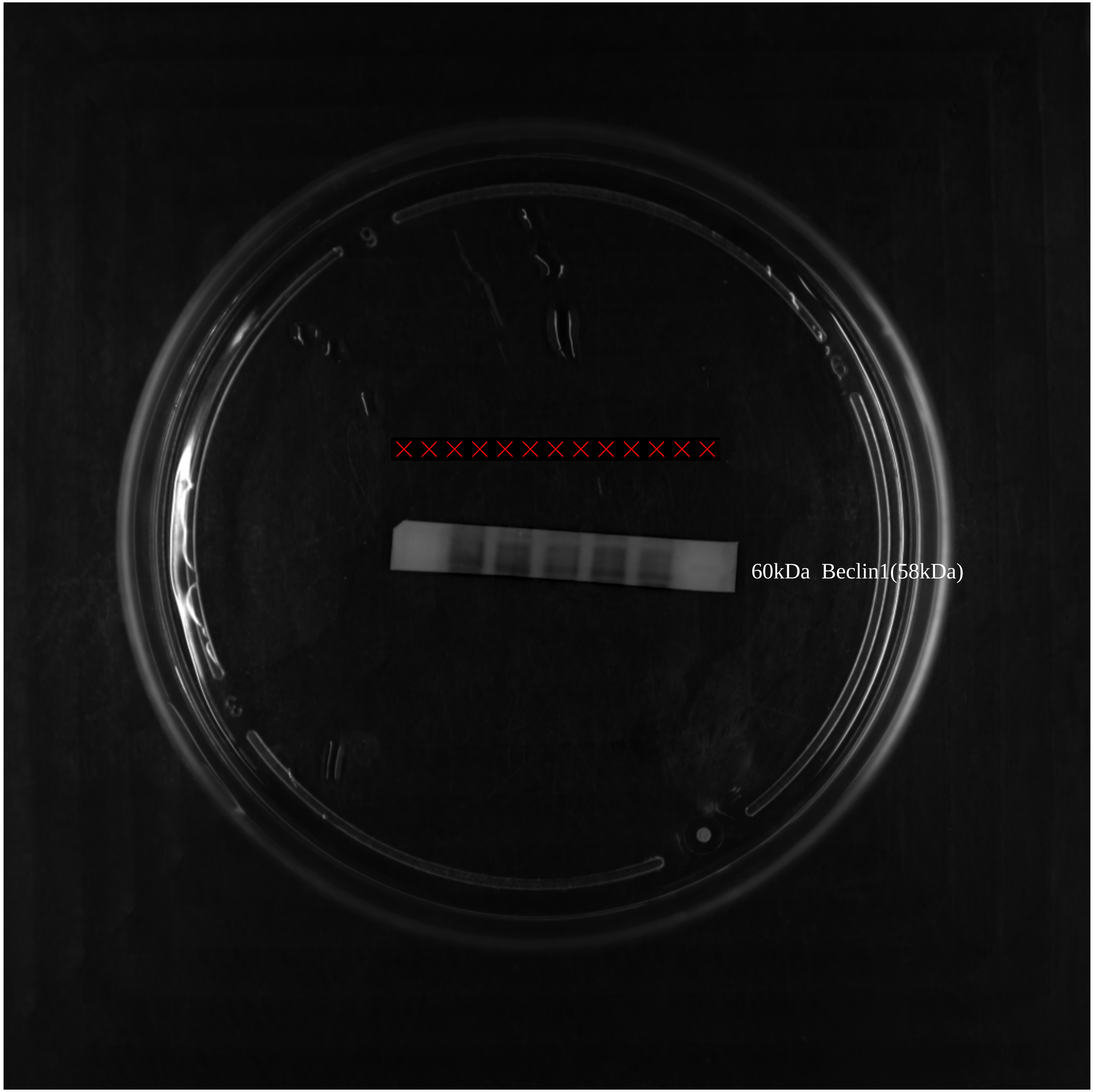

Con Mod NGR1 SSB2 NS

Beclin1-2-β-Actin

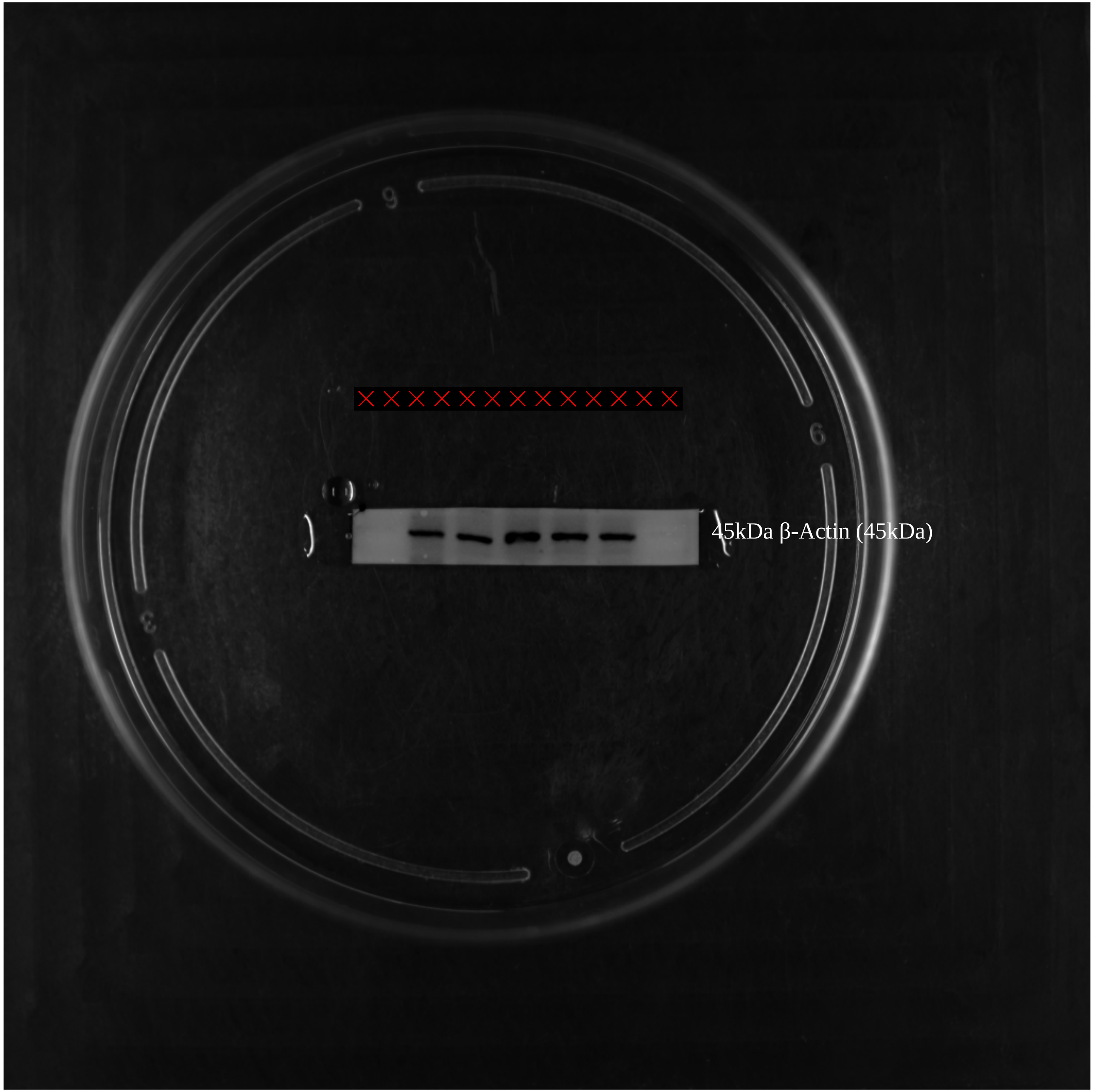

Con Mod NGR1 SSB2 NS

Beclin1-3-full

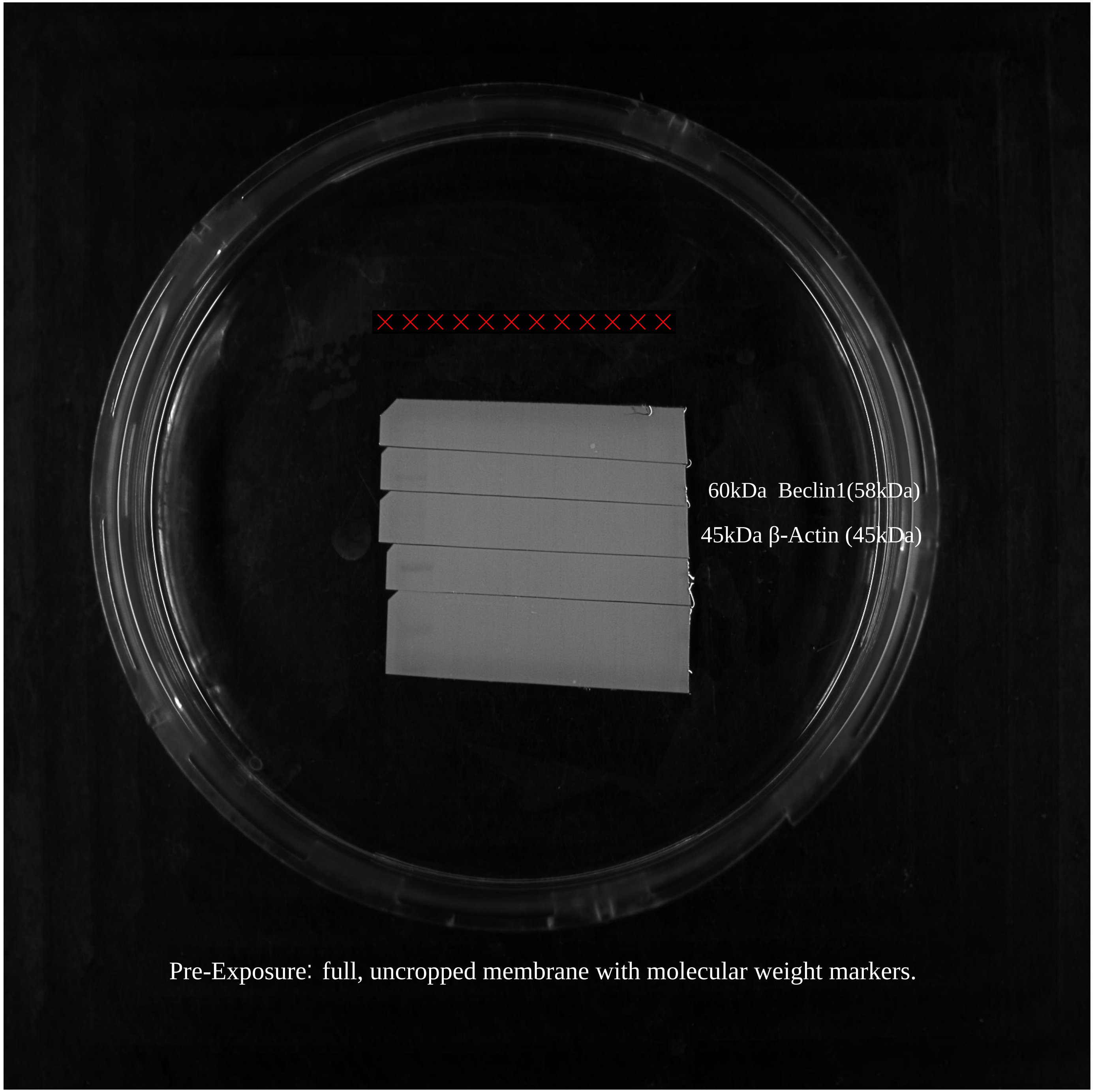

Con Mod NGR1 SSB2 NS

Beclin1-3-

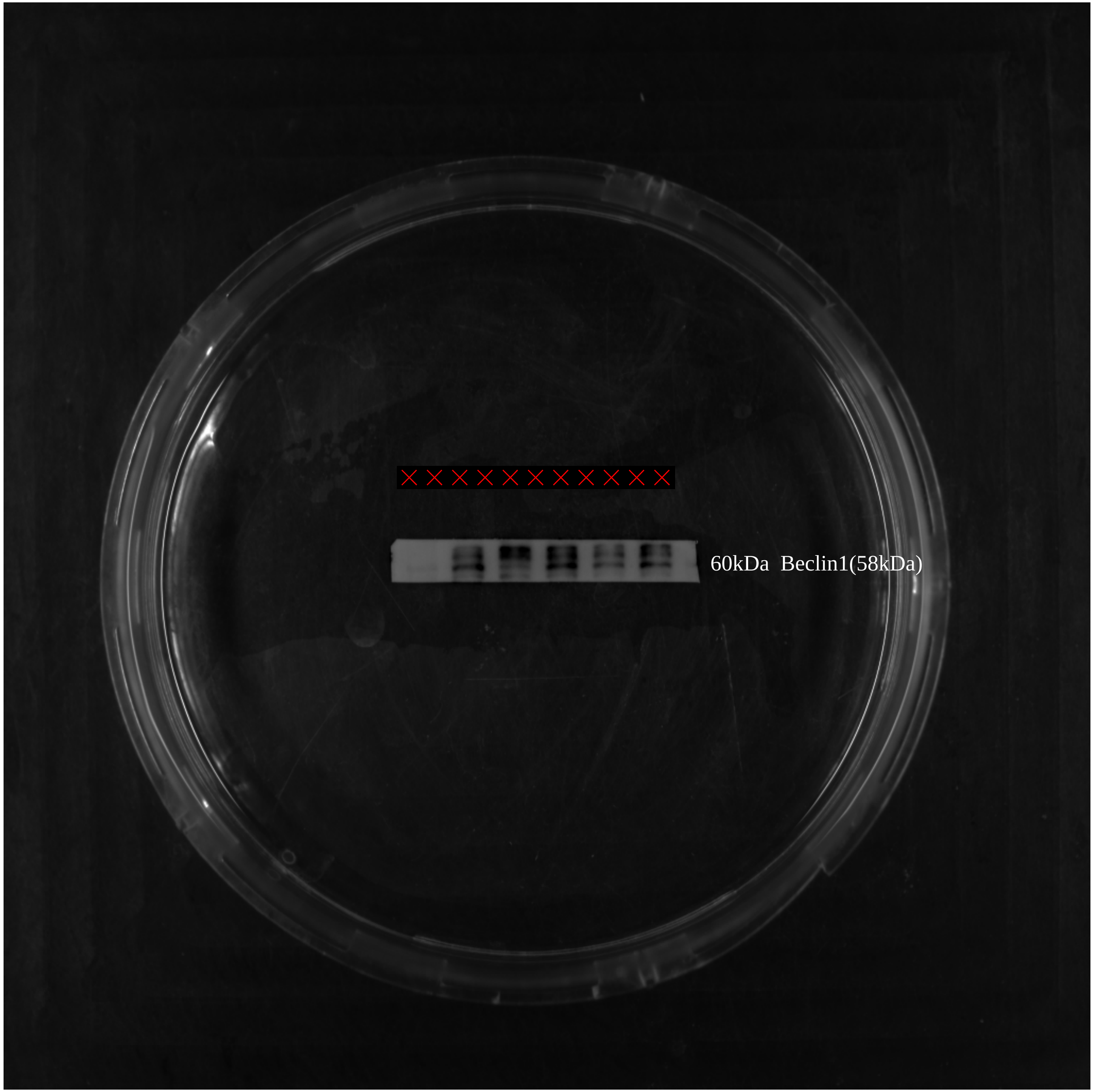

Con Mod NGR1 SSB2 NS

Beclin1-3-β-Actin

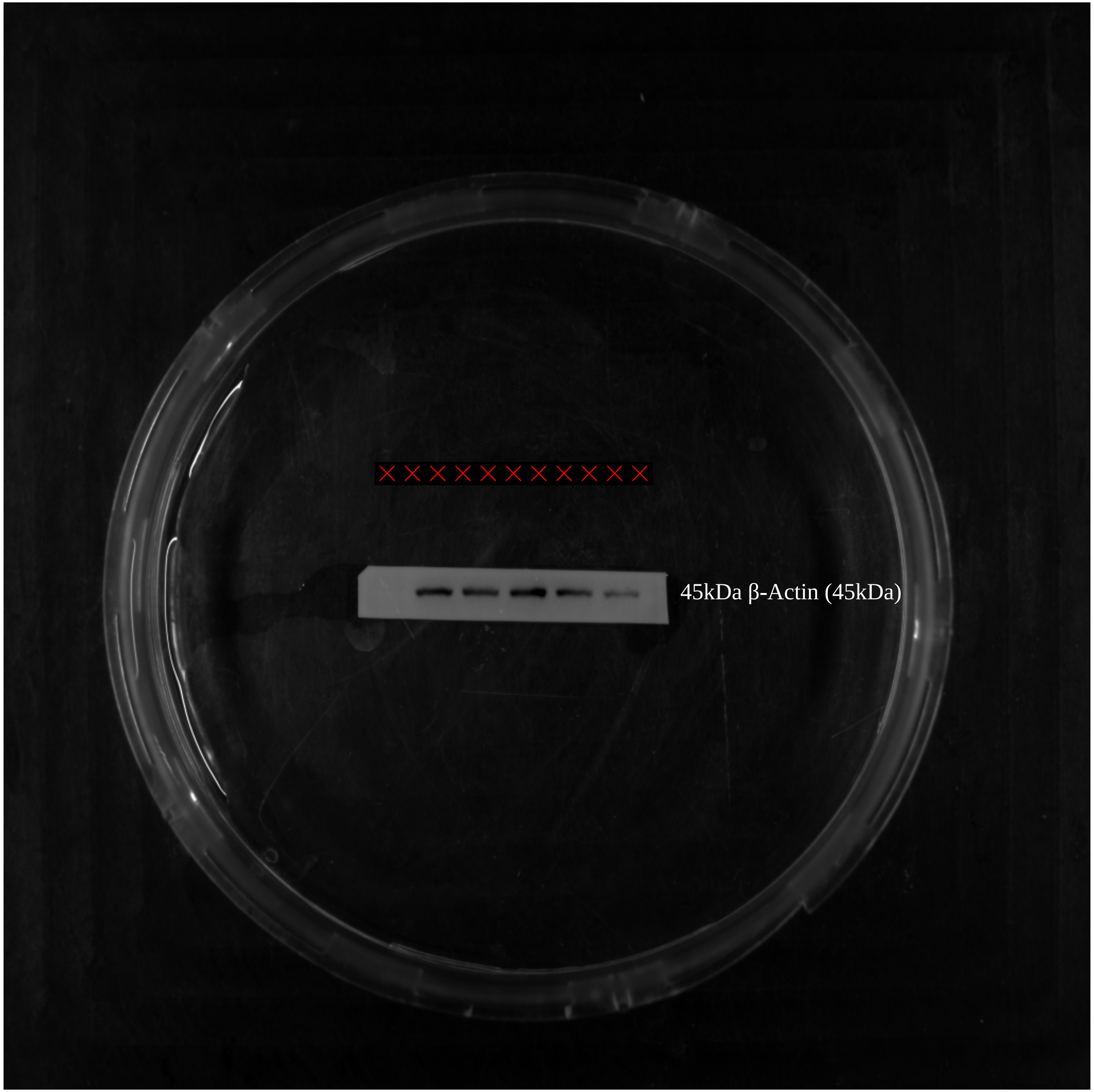

Con Mod NGR1 SSB2 NS

LC3B-3&4-full

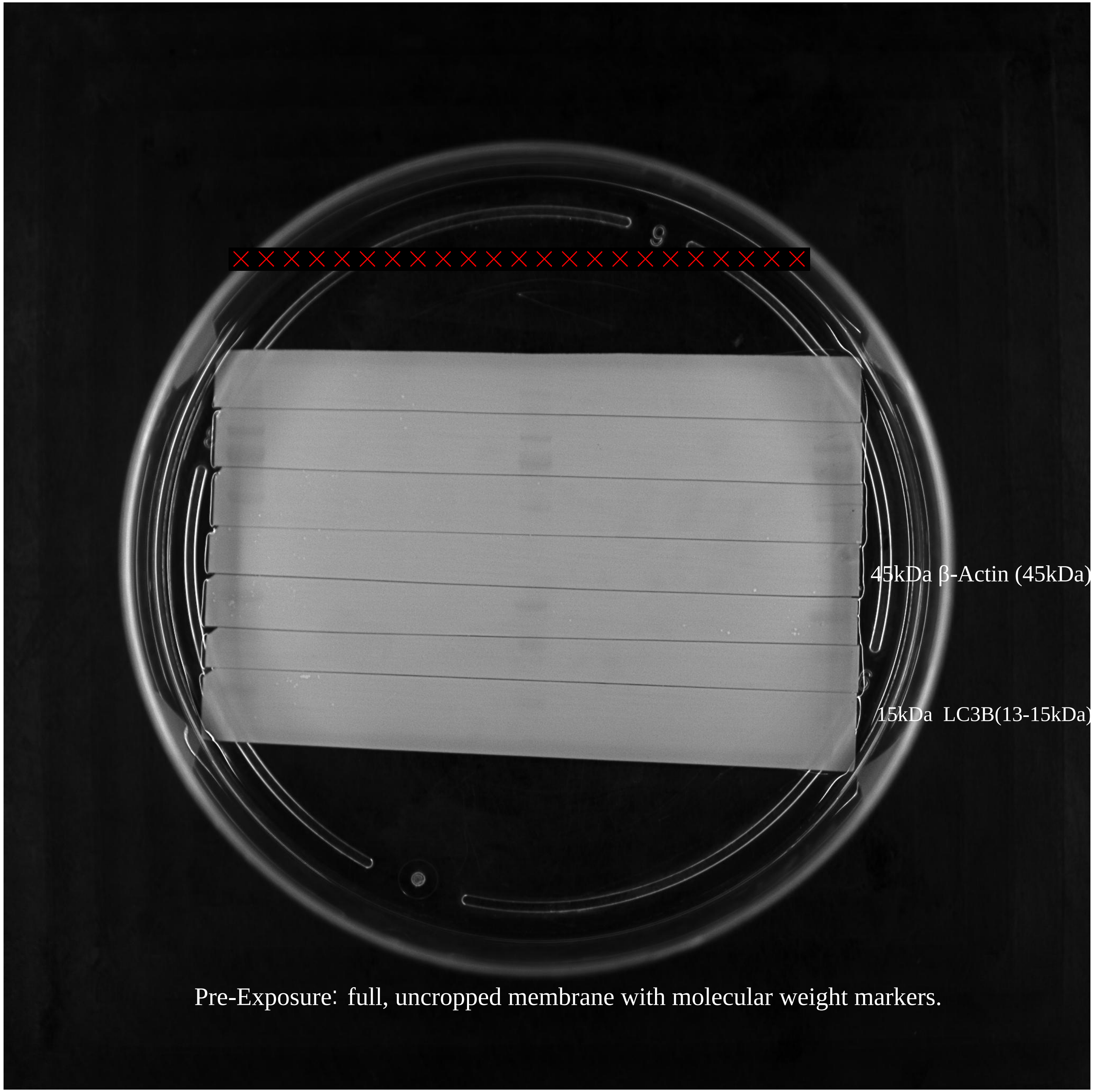

Con Mod NGR1 SSB2 NS Con Mod NGR1 SSB2 NS

LC3B-3&4

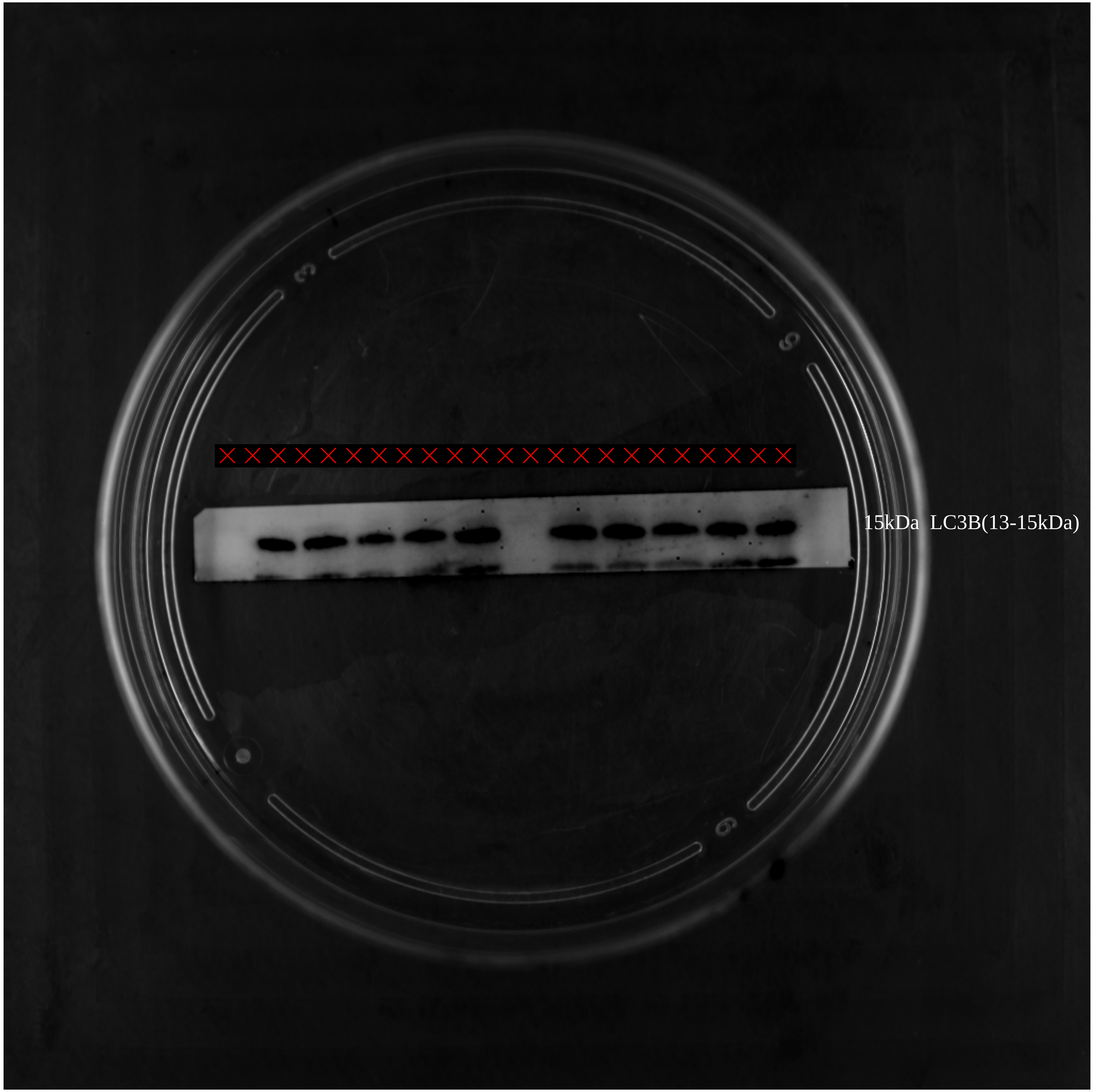

Con Mod NGR1 SSB2 NS Con Mod NGR1 SSB2 NS

LC3B-3&4-β-Actin

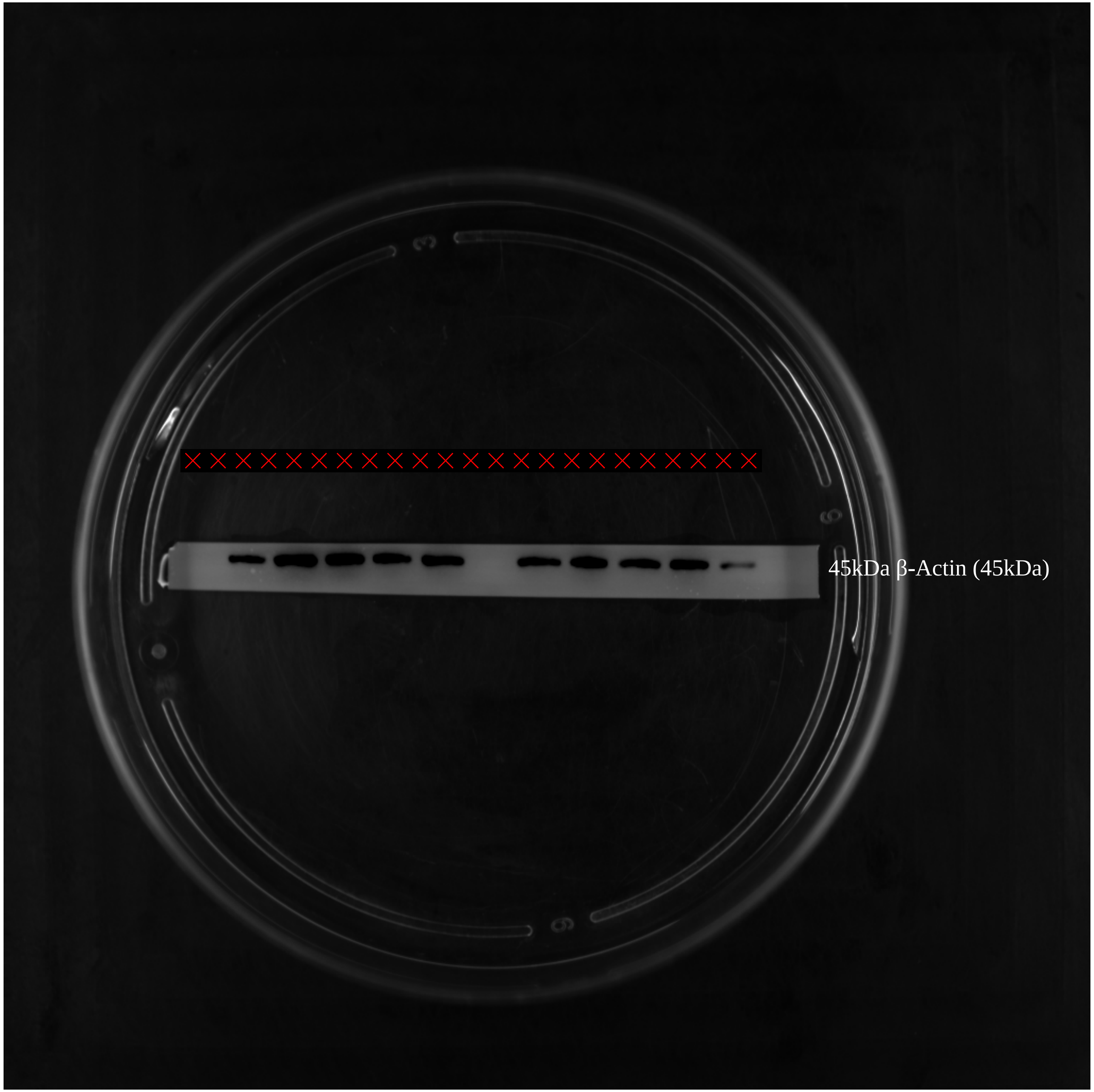

Con Mod NGR1 SSB2 NS Con Mod NGR1 SSB2 NS

P62-2&3-full

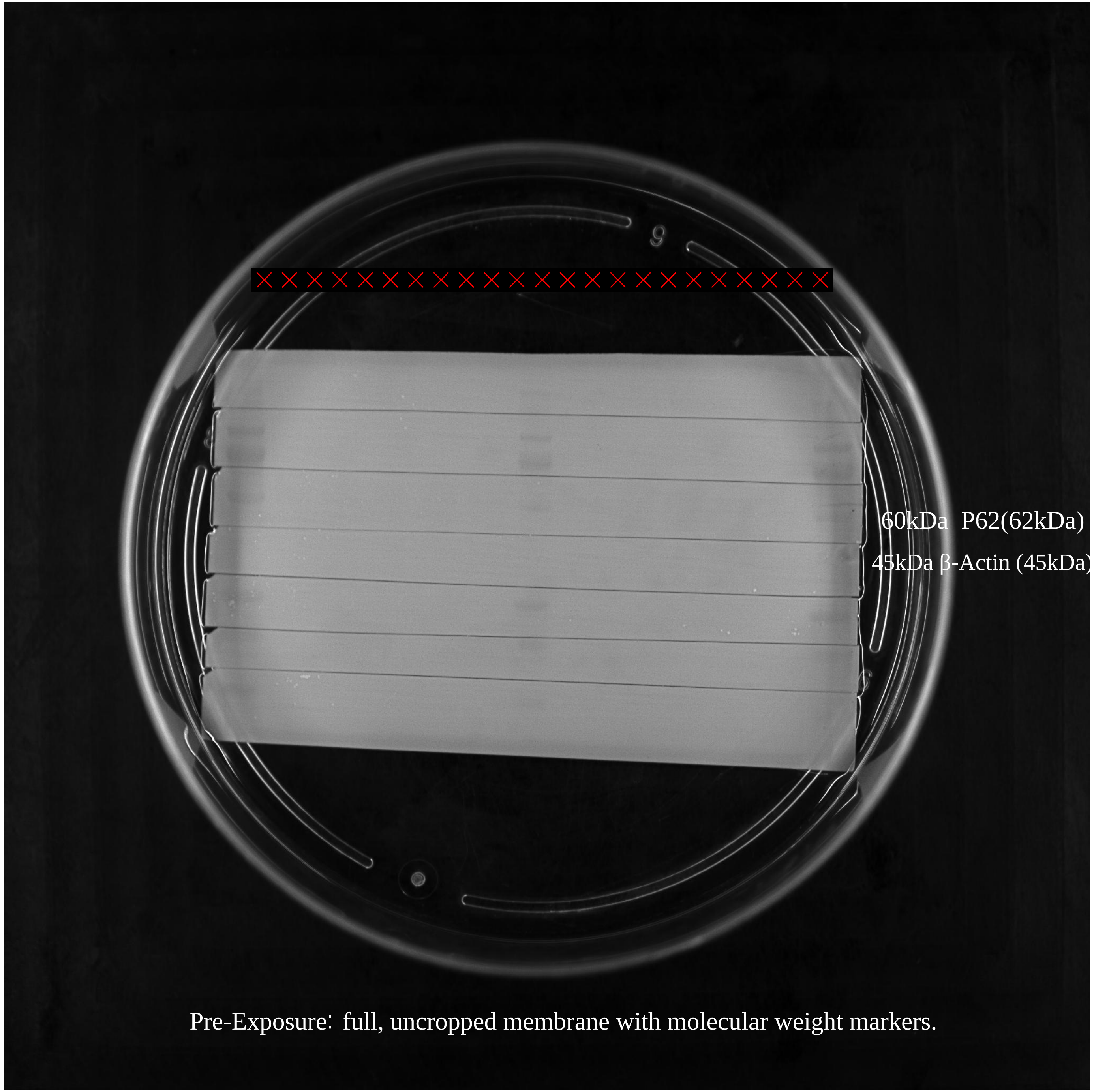

Con Mod NGR1 SSB2 NS Con Mod NGR1 SSB2 NS

P62-2&3

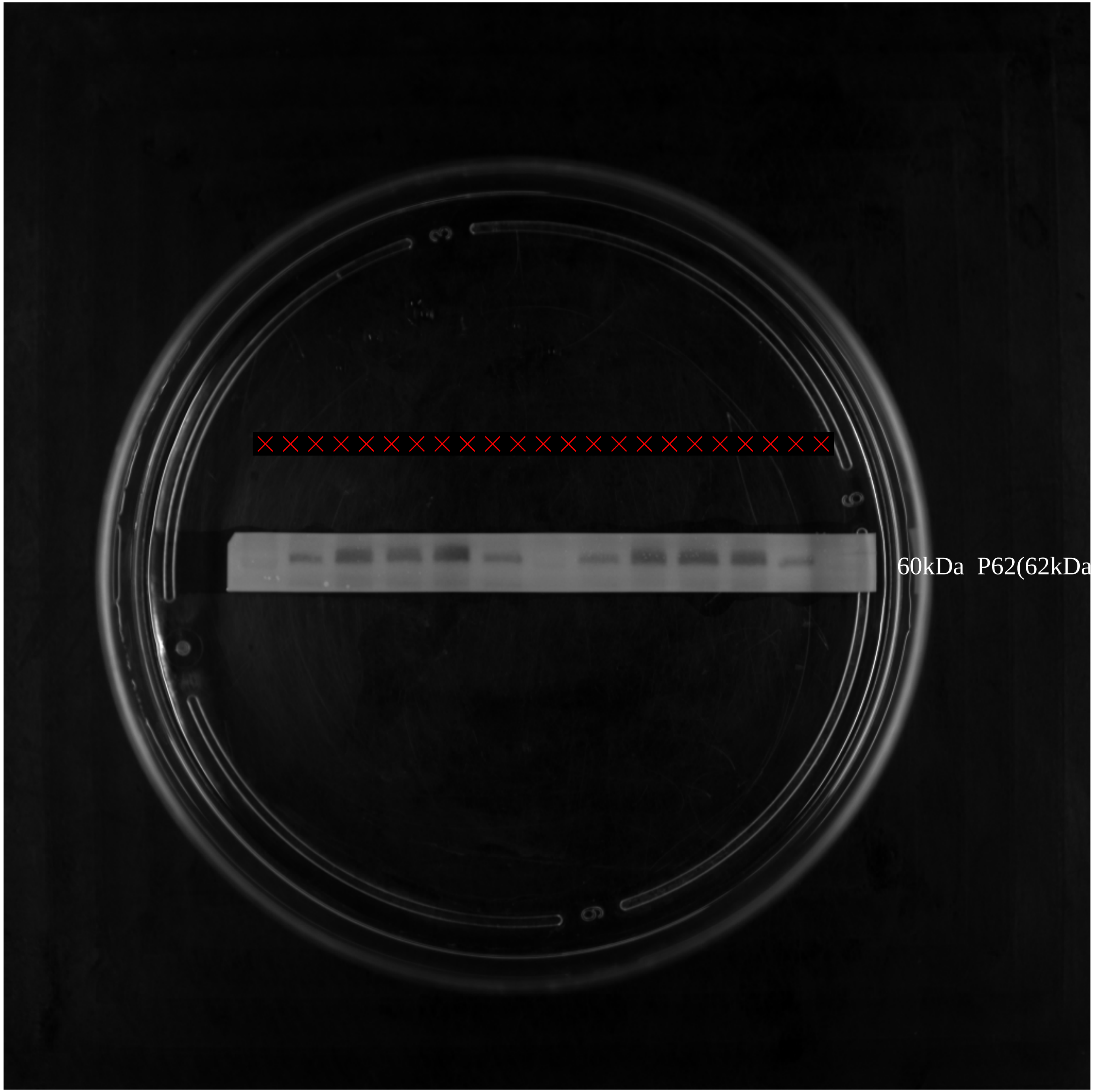

Con Mod NGR1 SSB2 NS Con Mod NGR1 SSB2 NS

P62-2&3-β-Actin

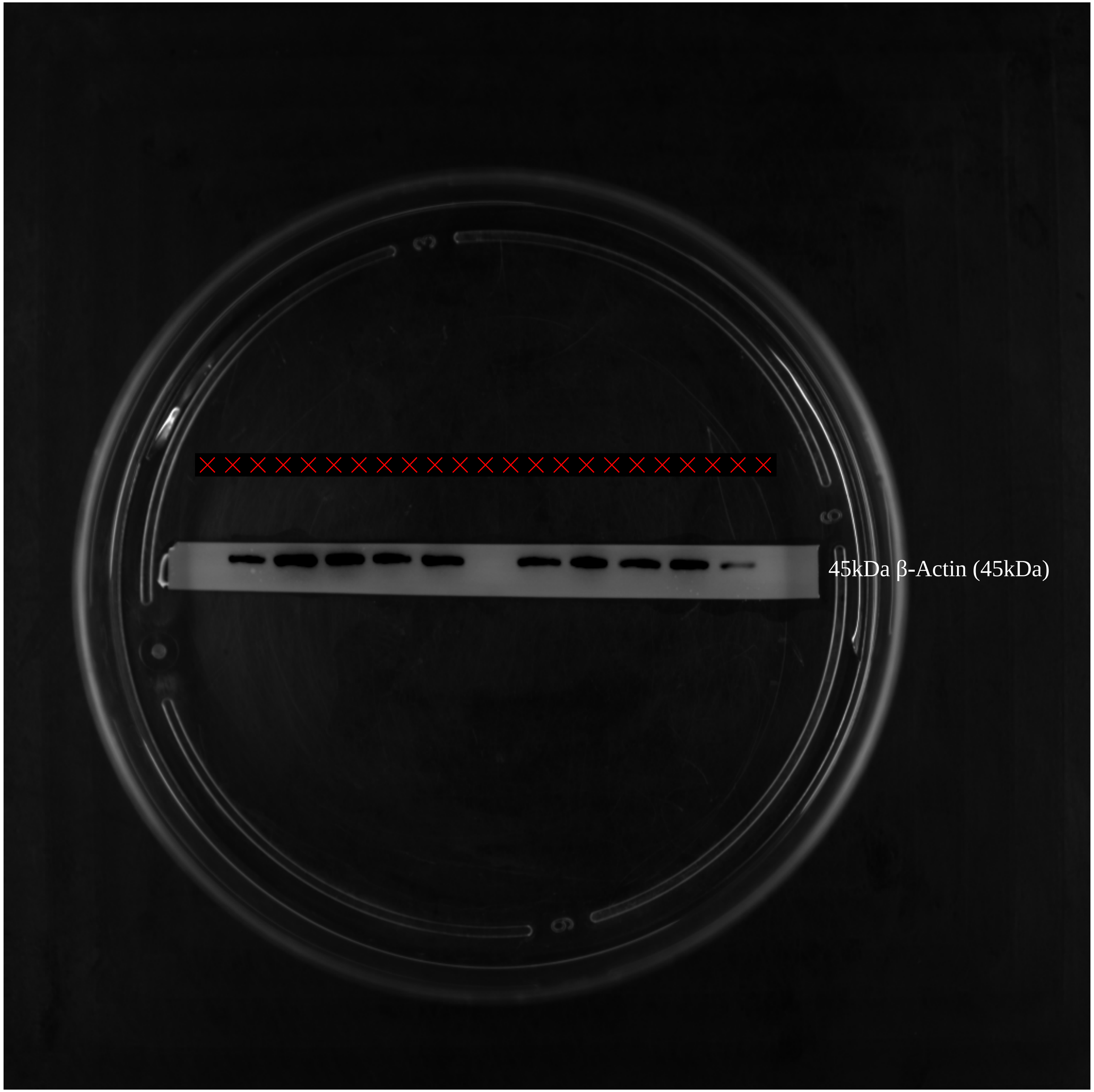

Con Mod NGR1 SSB2 NS Con Mod NGR1 SSB2 NS

mTOR-3&4-full

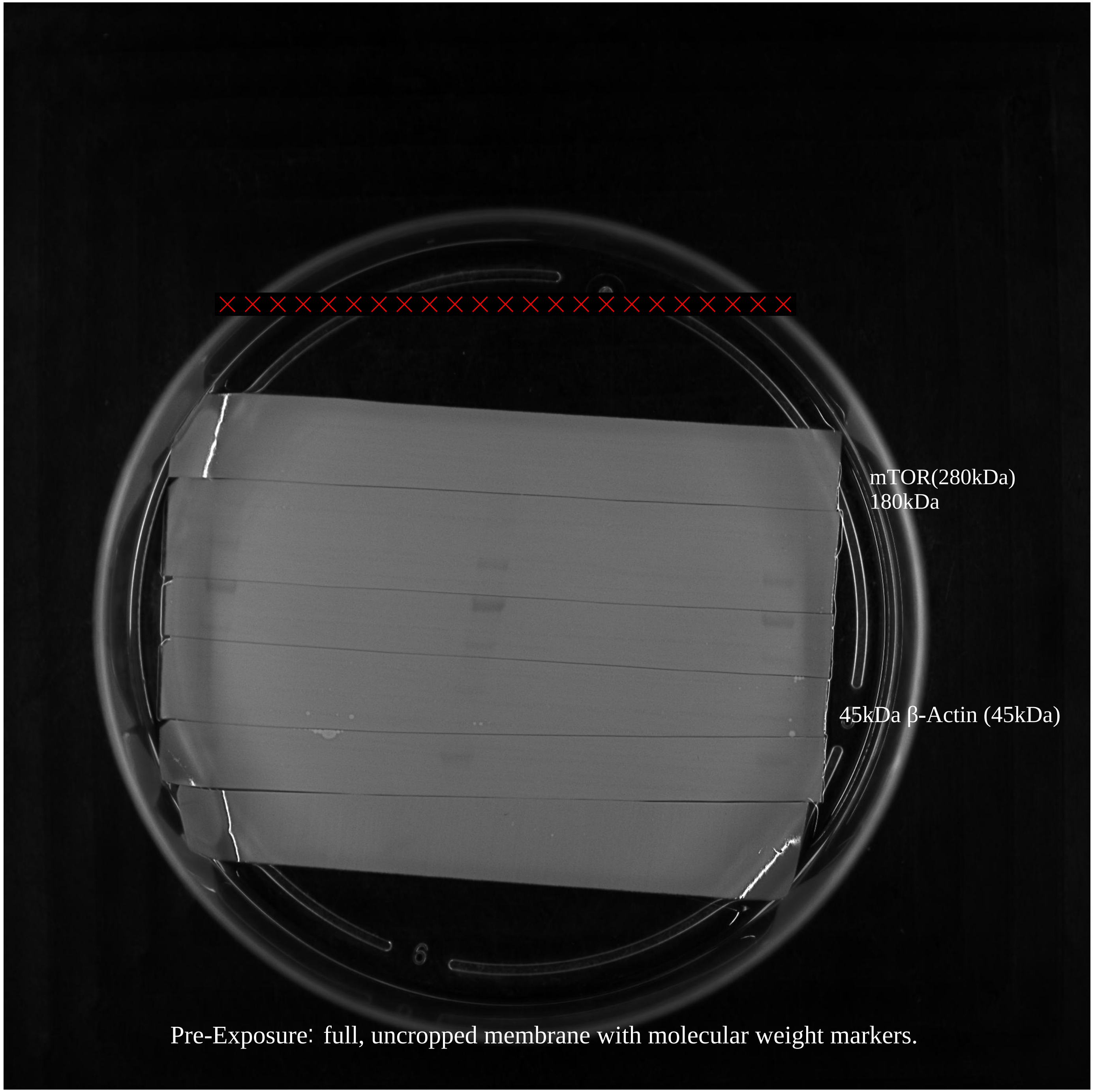

Pre-Exposure: full, uncropped membrane with molecular weight markers.

Con Mod NS NS+Ra NS+3-MA Con Mod NS NS+Ra NS+3-MA

mTOR-3&4

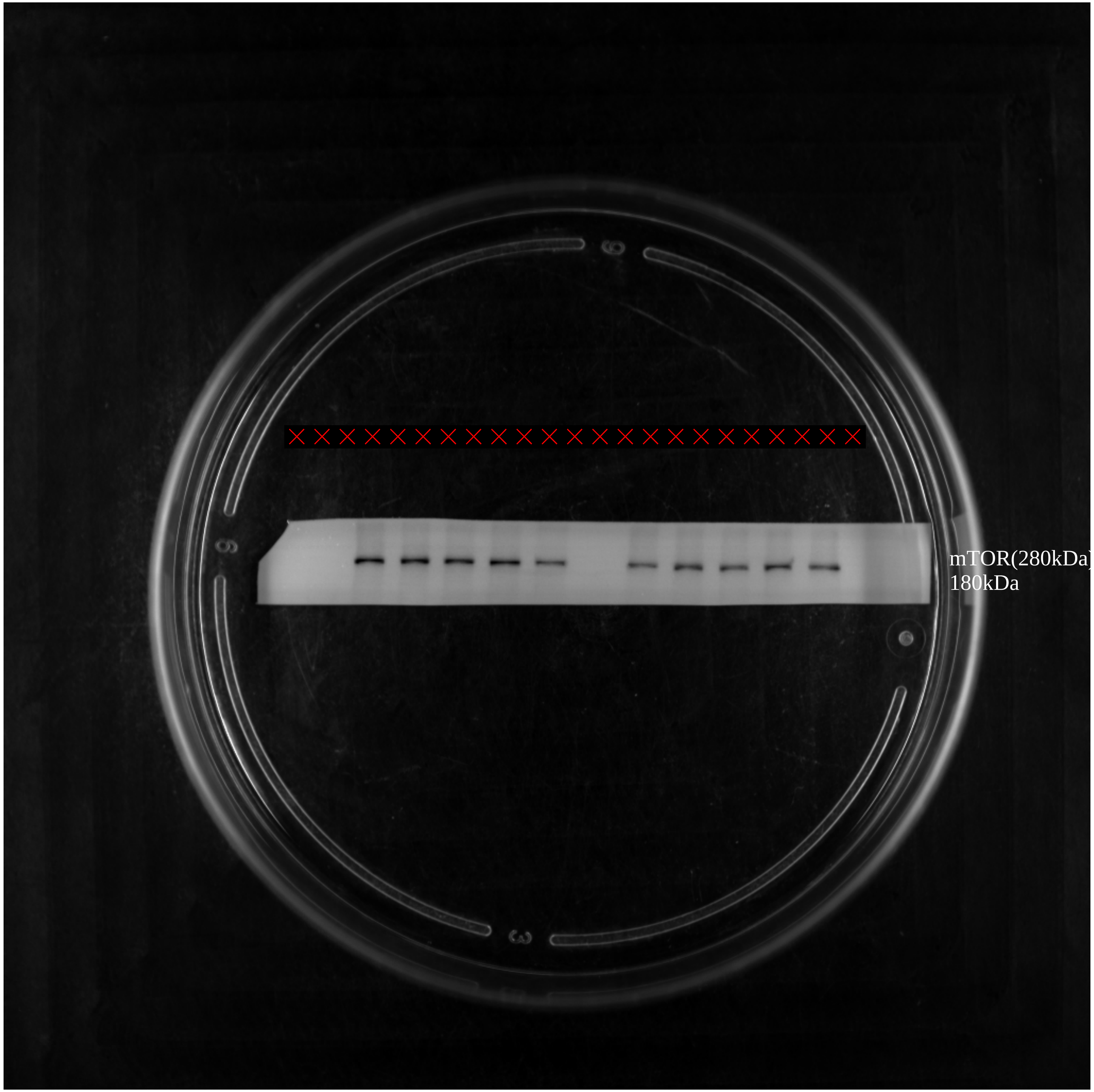

Con Mod NS NS+Ra NS+3-MA Con Mod NS NS+Ra NS+3-MA

mTOR-3&4-β-Actin

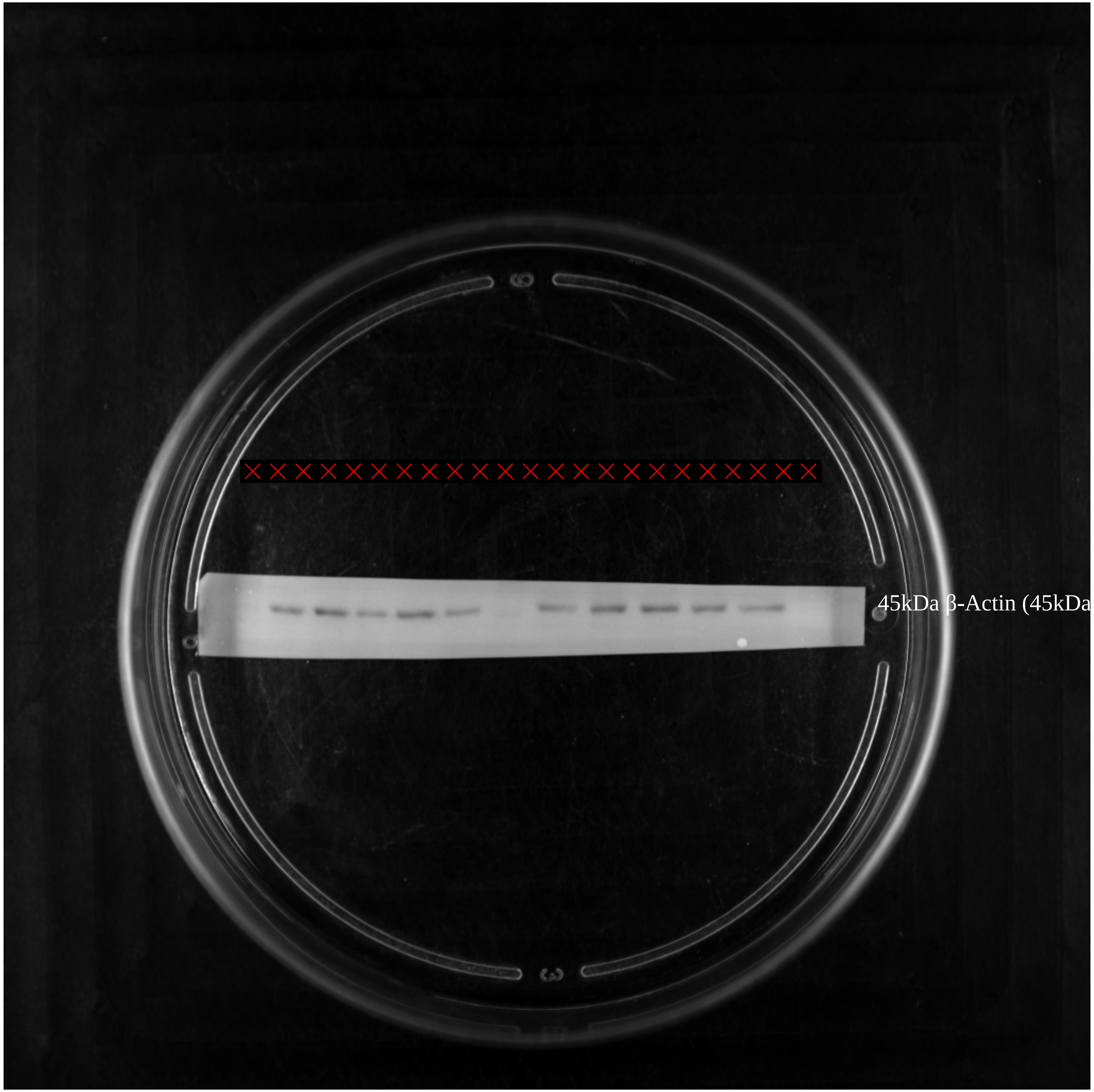

Con Mod NS NS+Ra NS+3-MA Con Mod NS NS+Ra NS+3-MA

p-mTOR-2&3-full

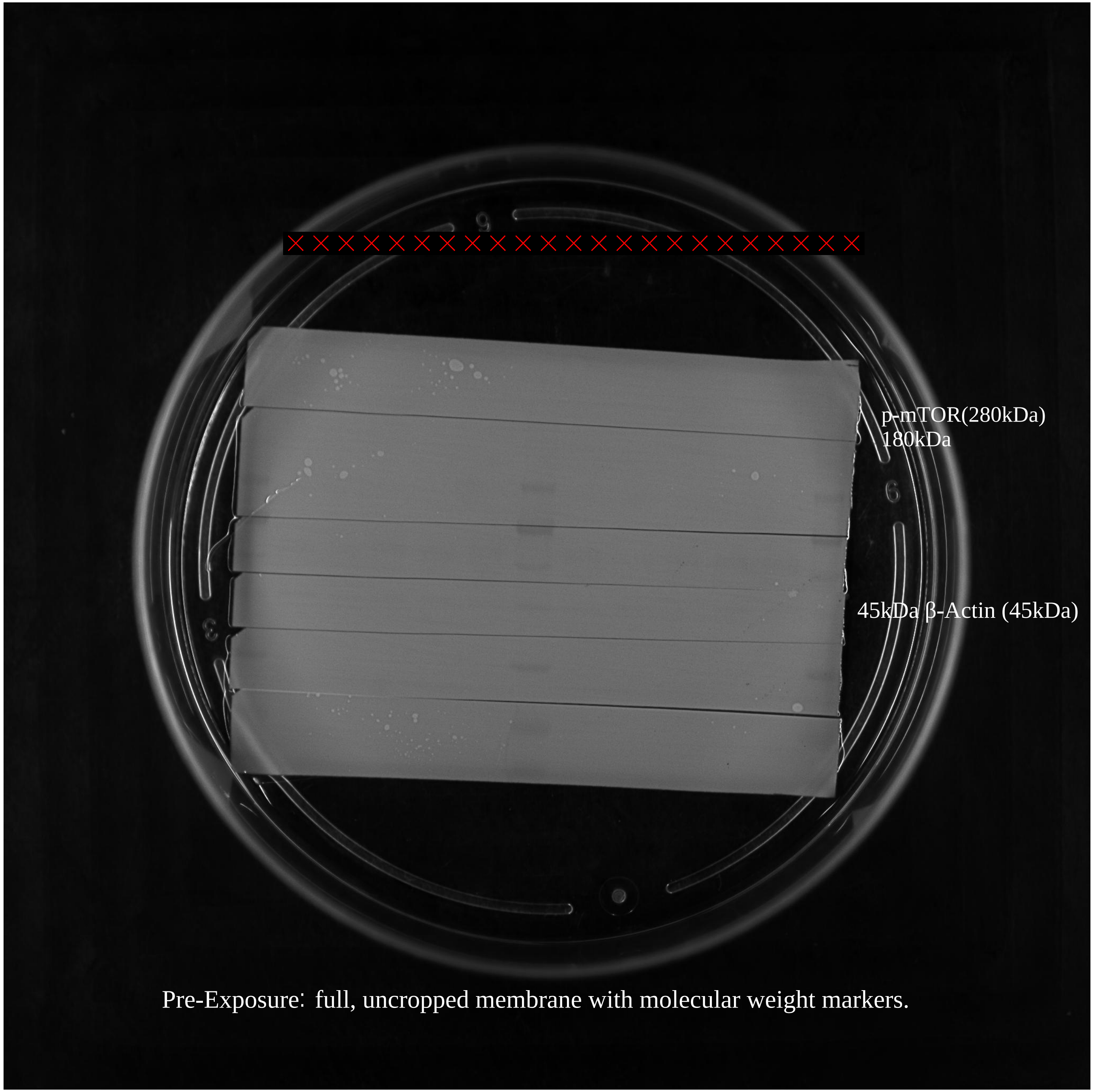

Con Mod NS NS+Ra NS+3-MA Con Mod NS NS+Ra NS+3-MA

p-mTOR-2&3

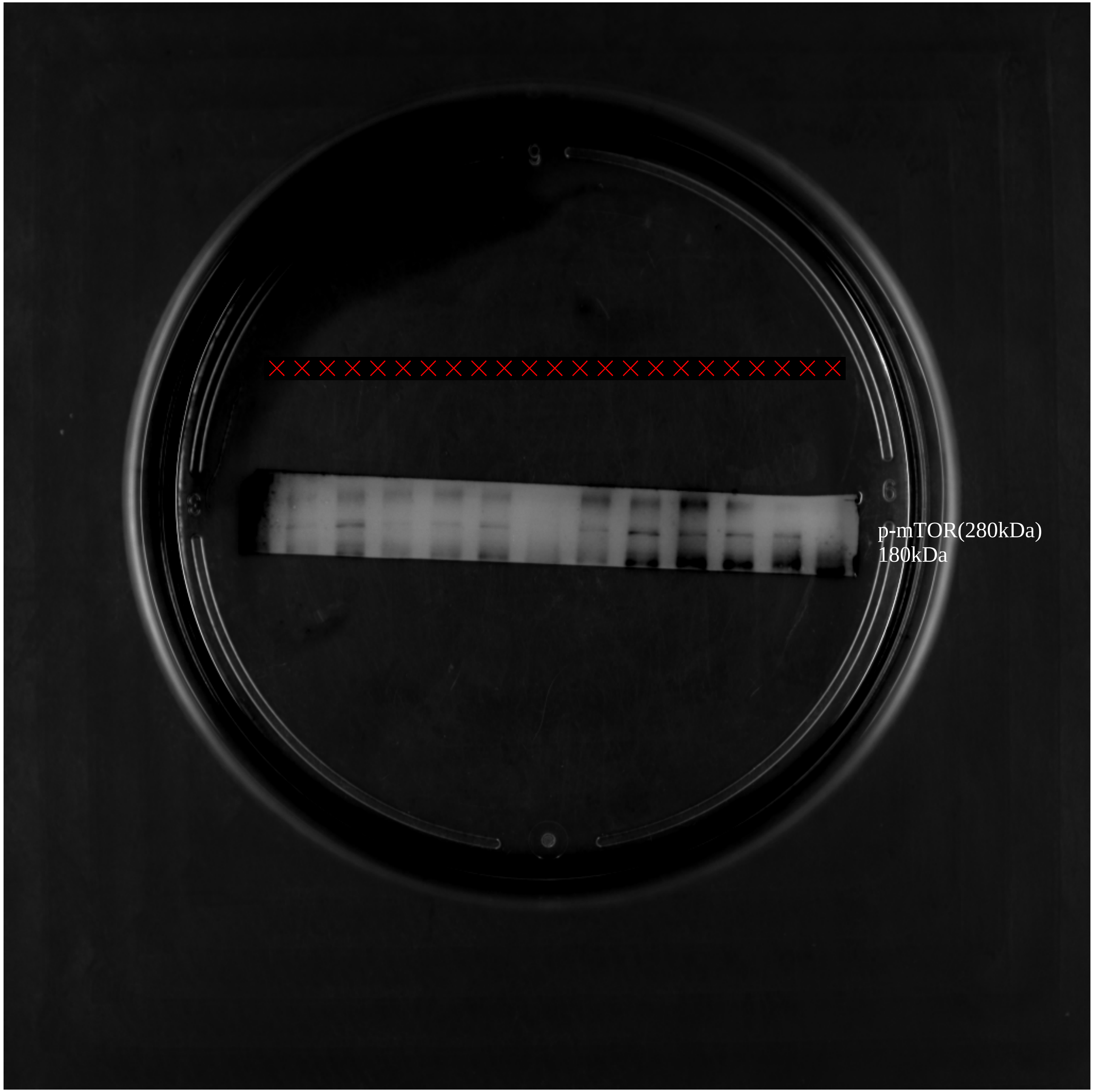

Con Mod NS NS+Ra NS+3-MA Con Mod NS NS+Ra NS+3-MA

p-mTOR-2&3-β-Actin

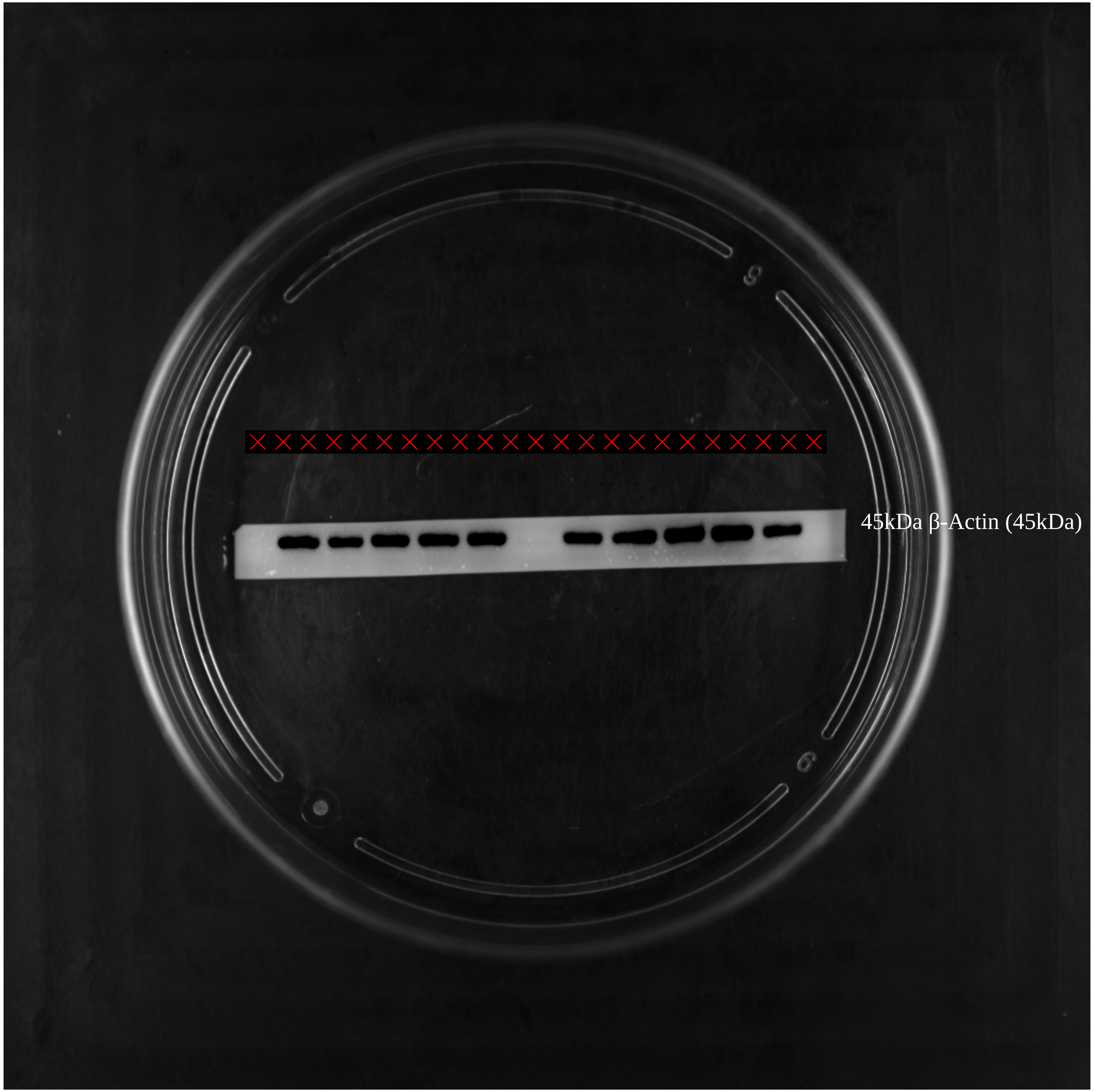

Con Mod NS NS+Ra NS+3-MA Con Mod NS NS+Ra NS+3-MA

NLRP3-2-full

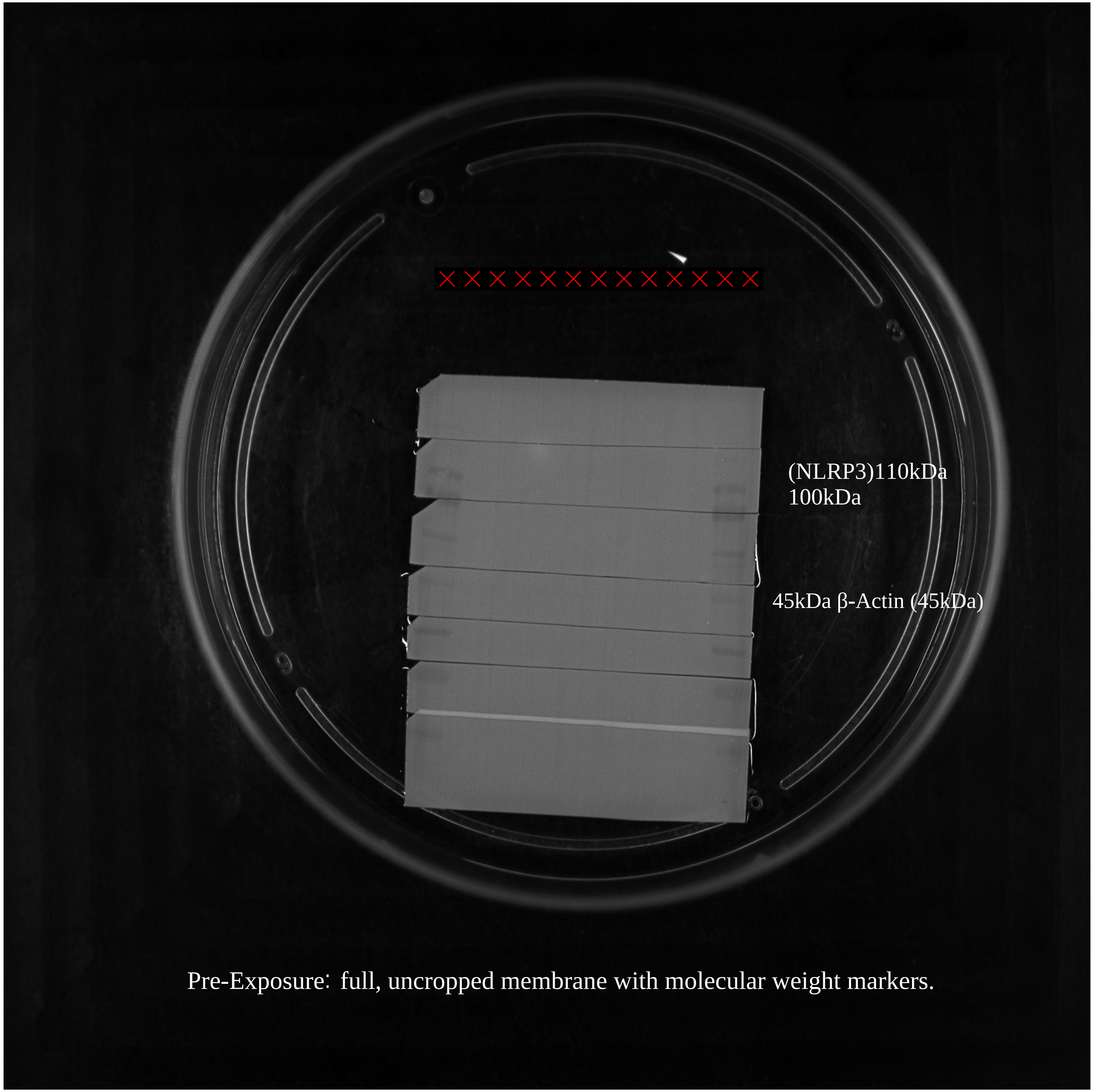

Con Mod NS NS+Ra NS+3-MA

NLRP3-2

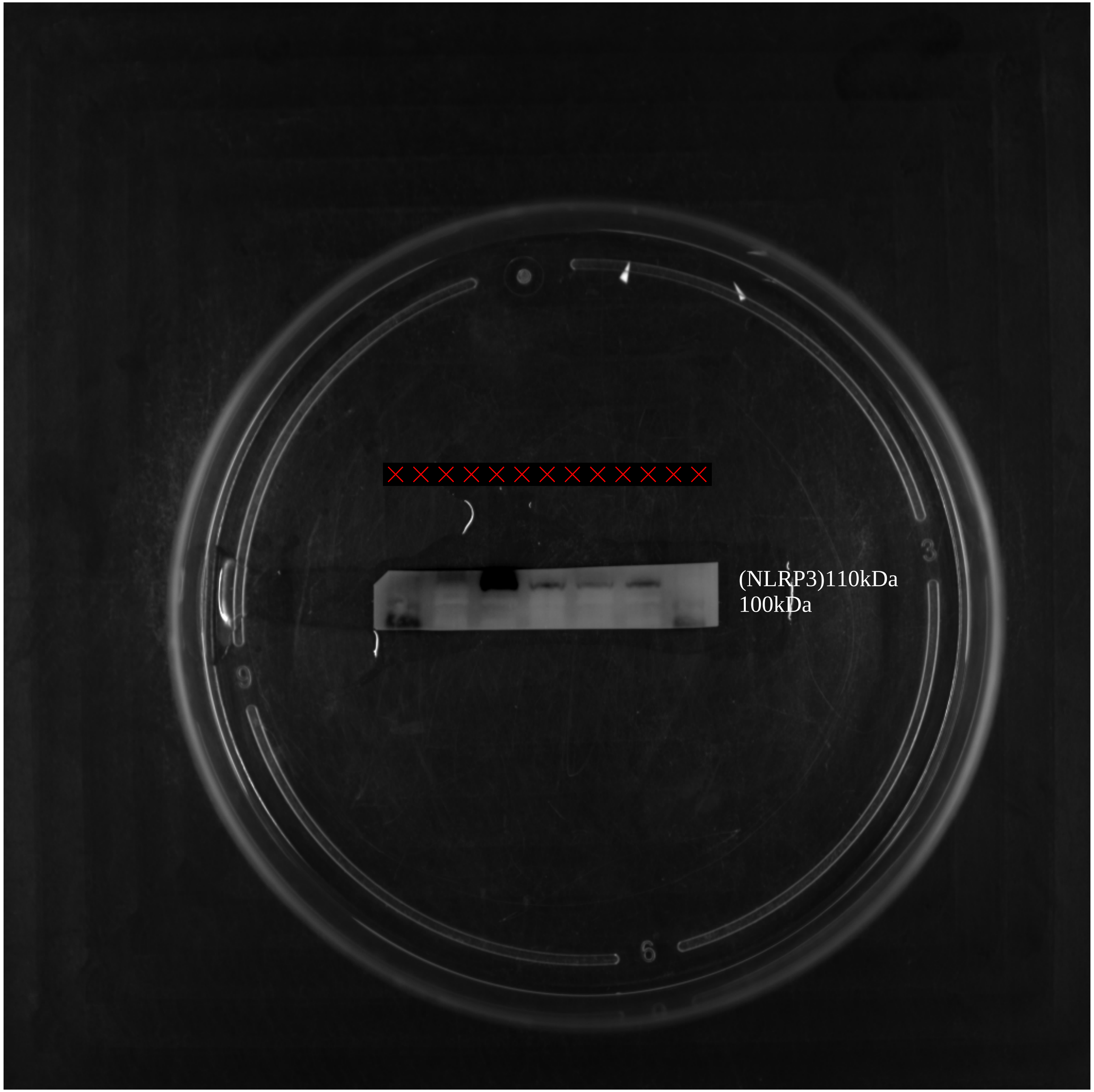

Con Mod NS NS+Ra NS+3-MA

NLRP3-2-β-Actin

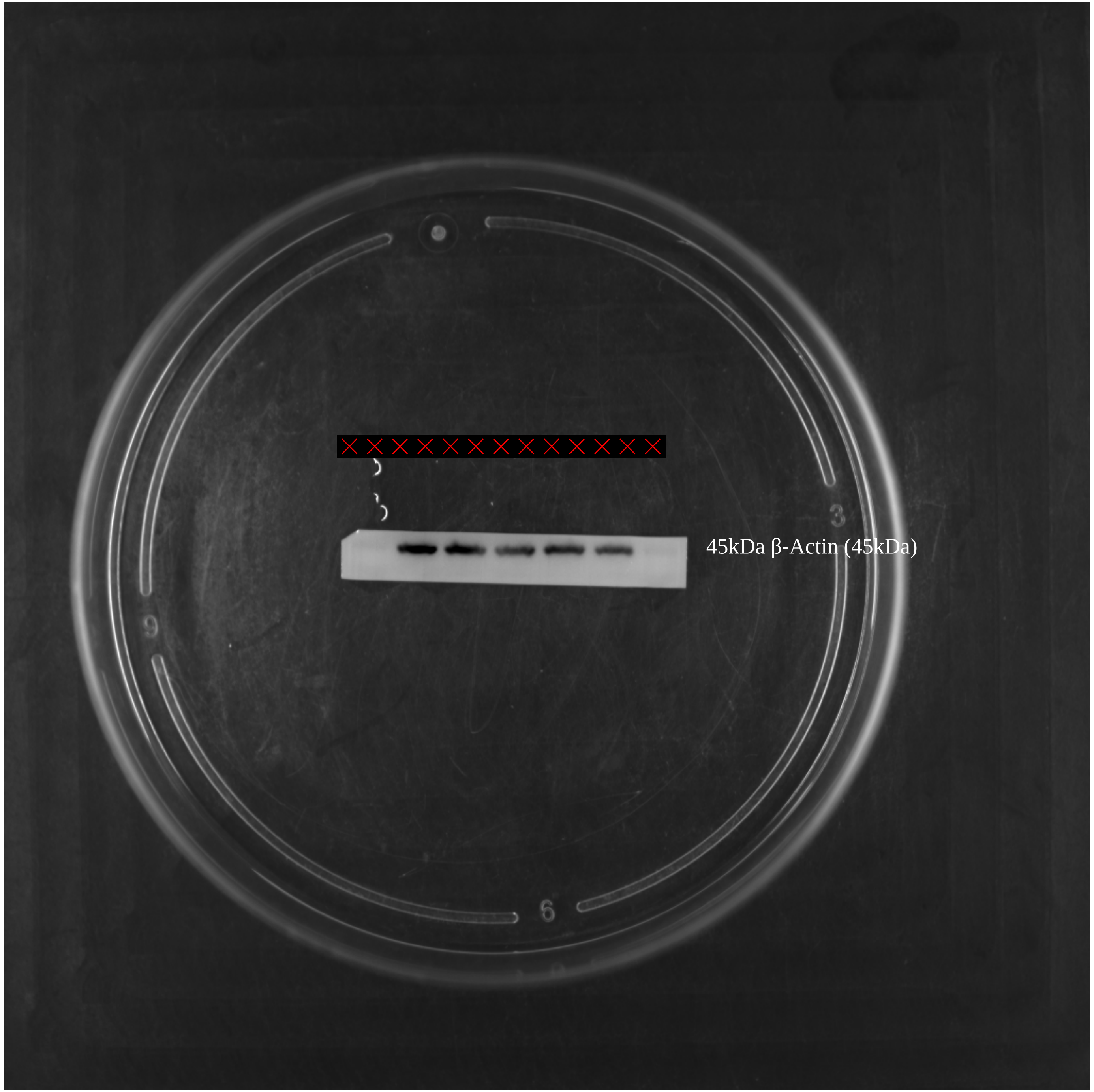

Con Mod NS NS+Ra NS+3-MA

BAX-2-full

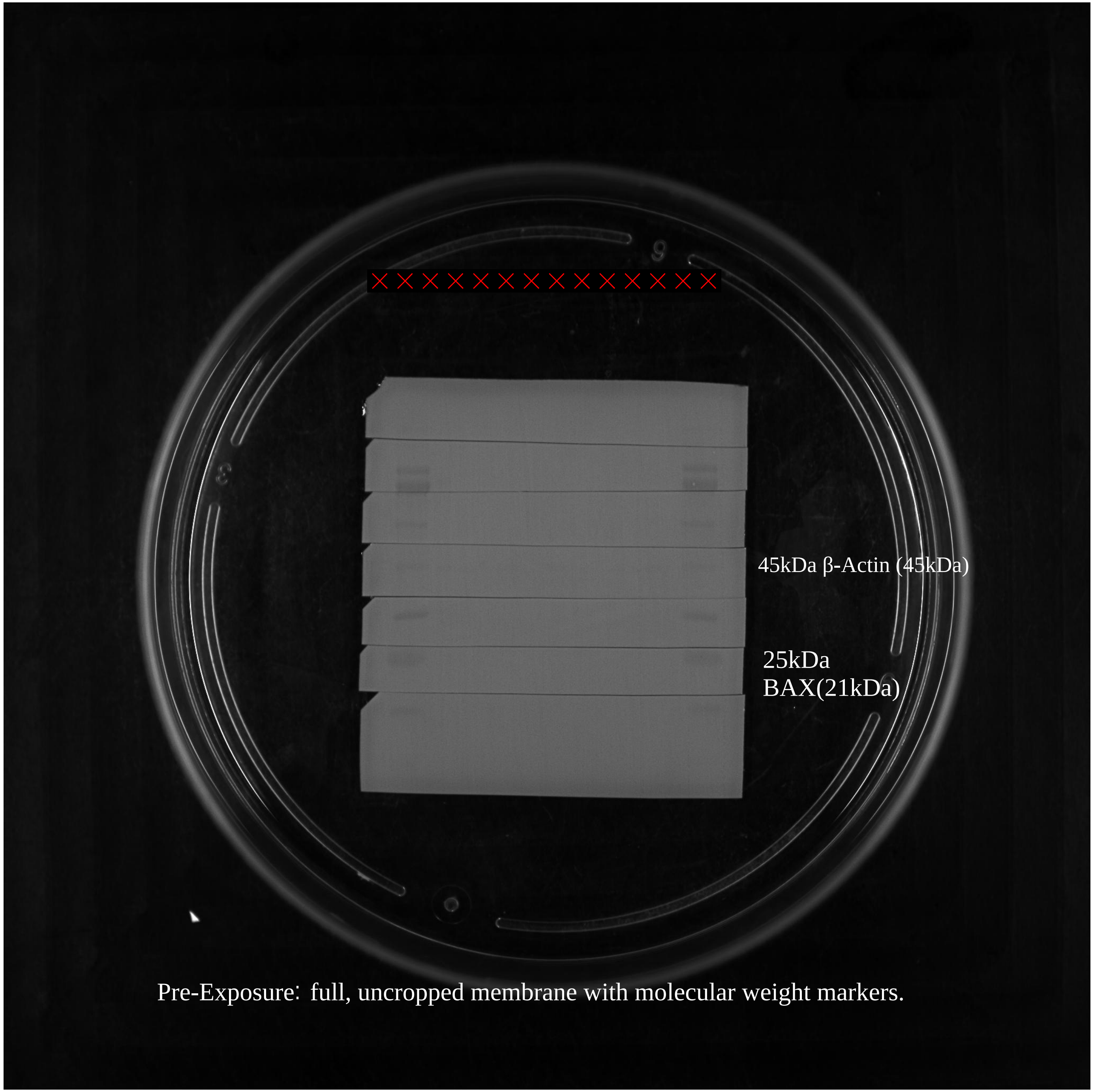

Con Mod NS NS+Ra NS+3-MA

BAX-2

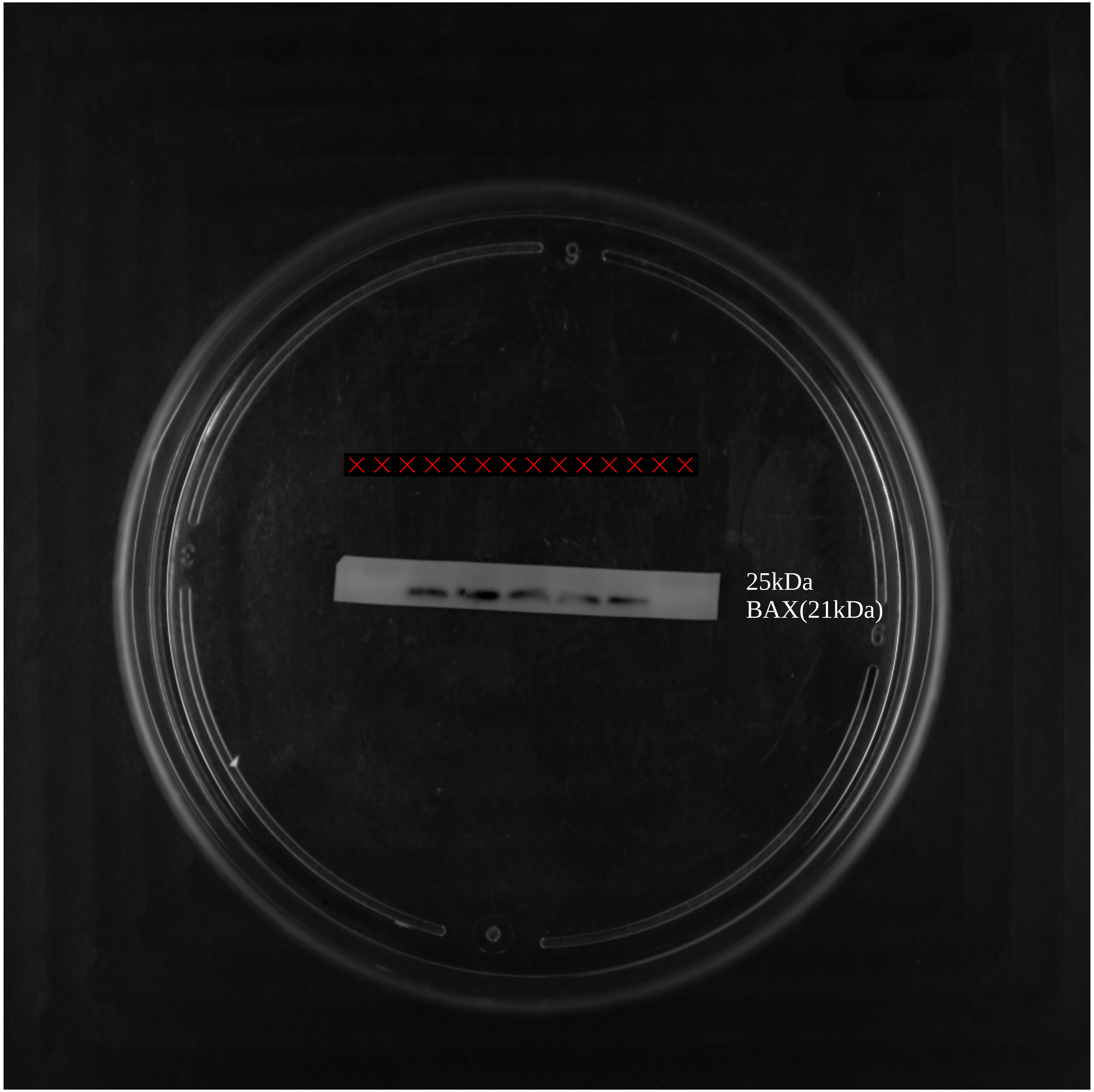

Con Mod NS NS+Ra NS+3-MA

BAX-2-β-Actin

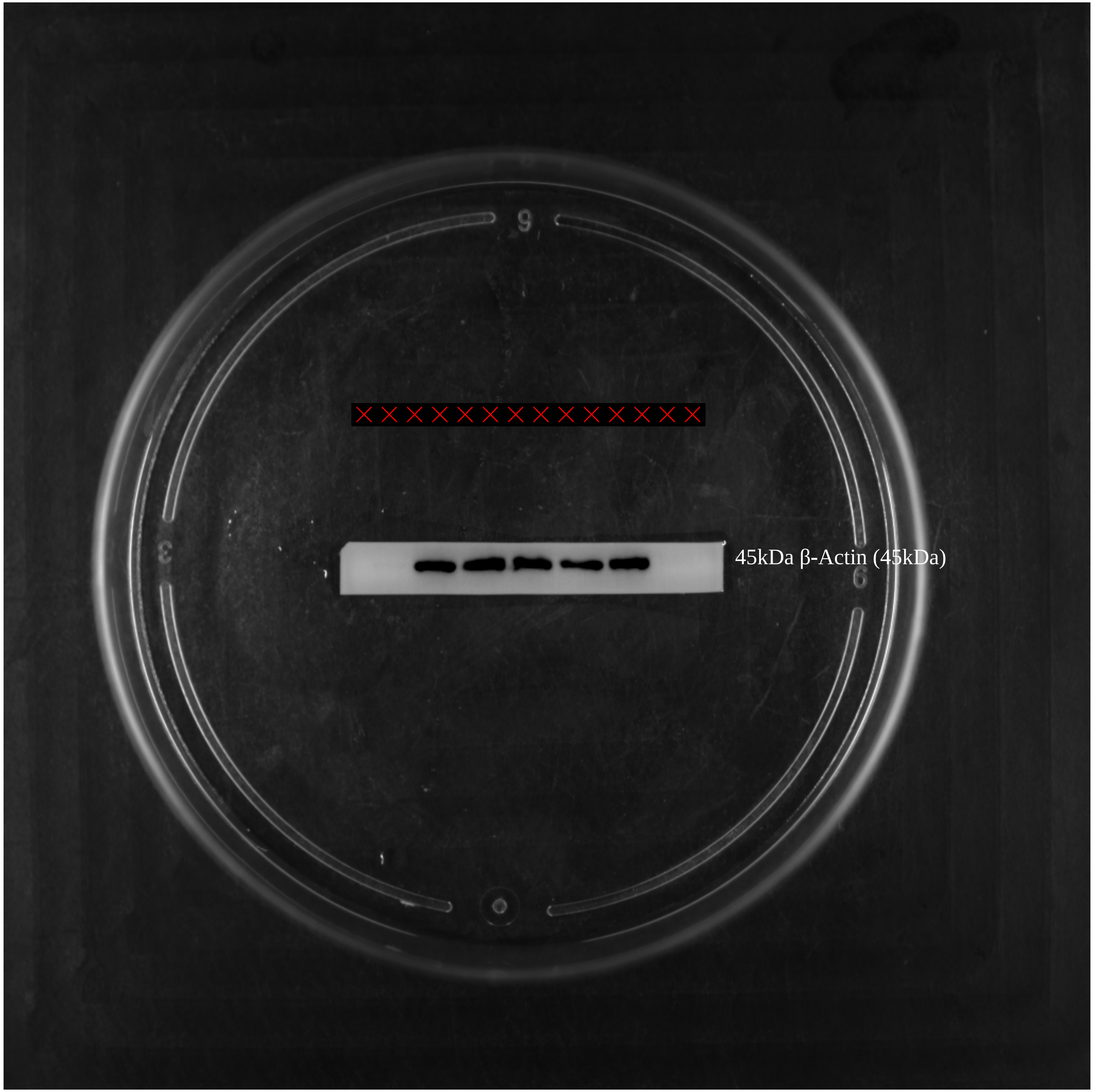

Con Mod NS NS+Ra NS+3-MA

BAX-3-full

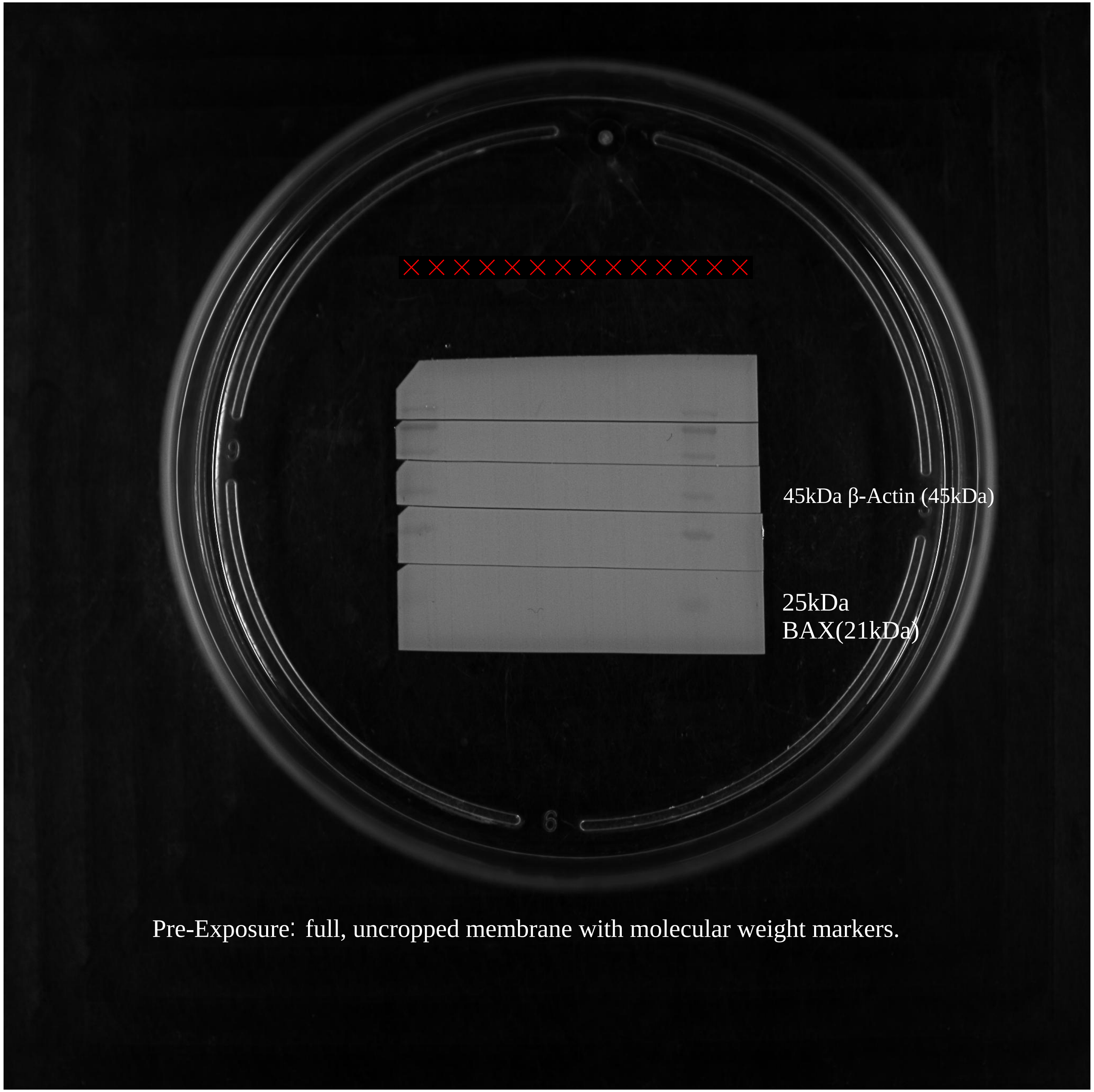

Con Mod NS NS+Ra NS+3-MA

BAX-3

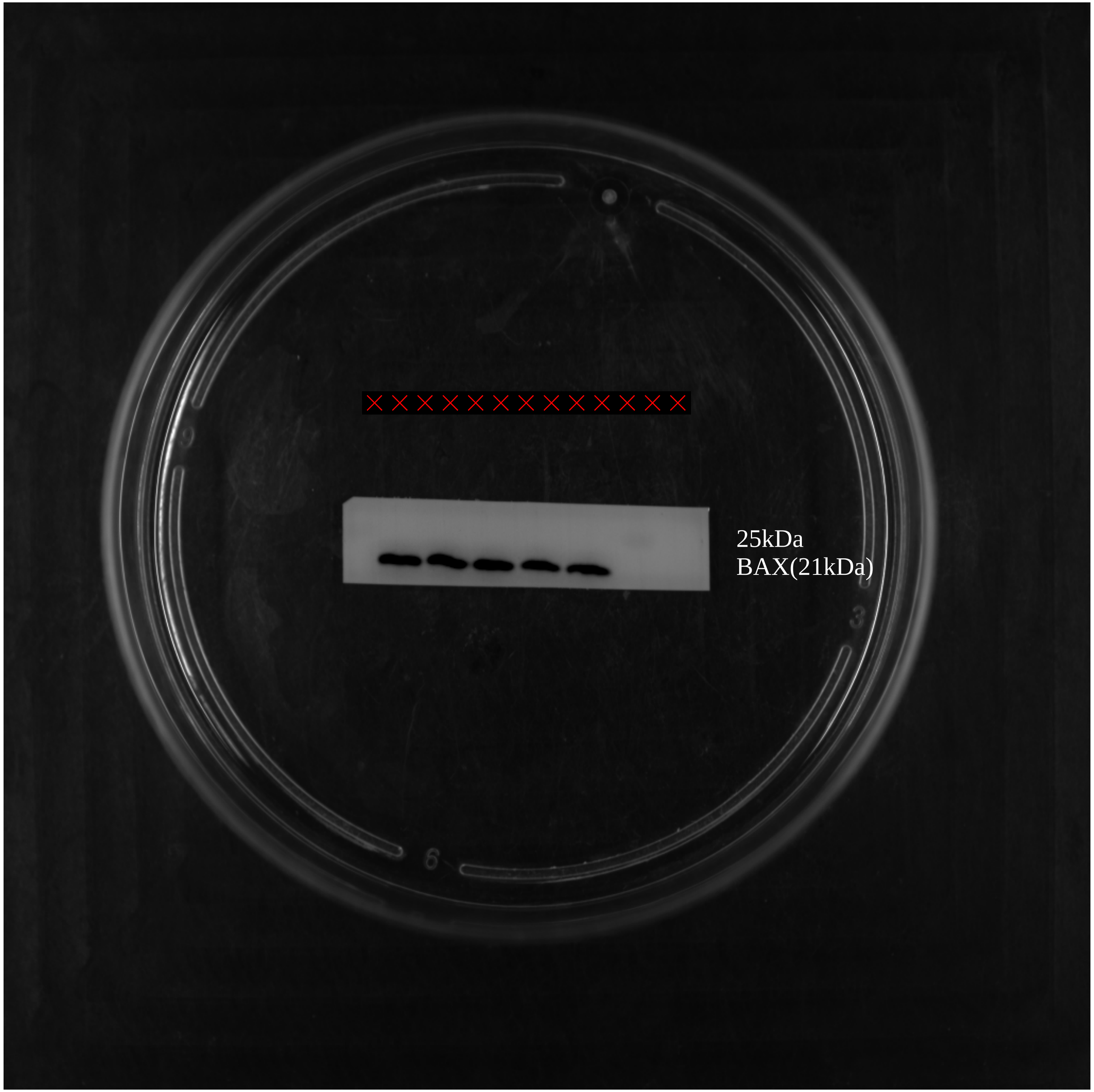

Con Mod NS NS+Ra NS+3-MA

BAX-3-β-Actin

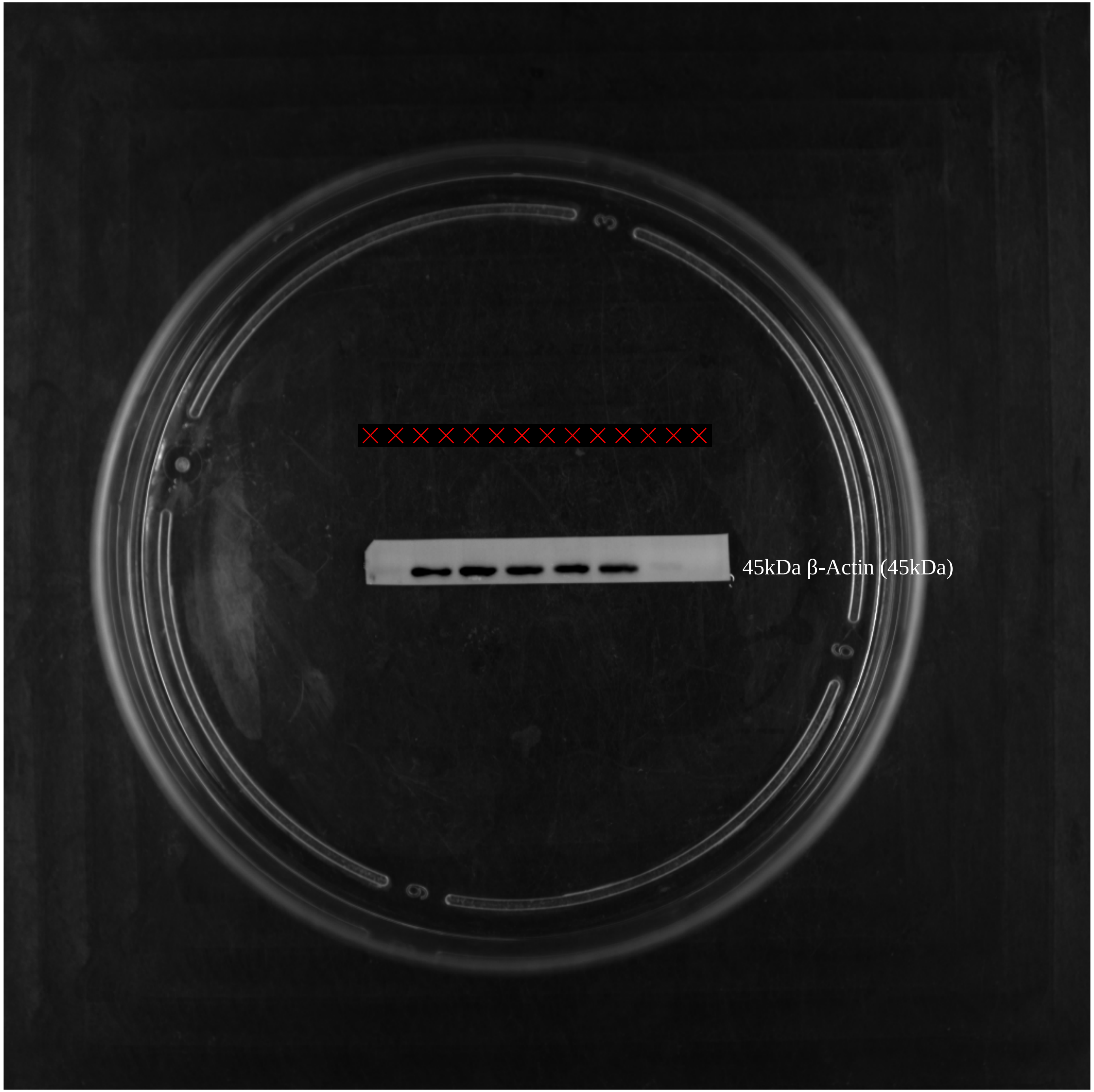

Con Mod NS NS+Ra NS+3-MA

BCL2-2-full

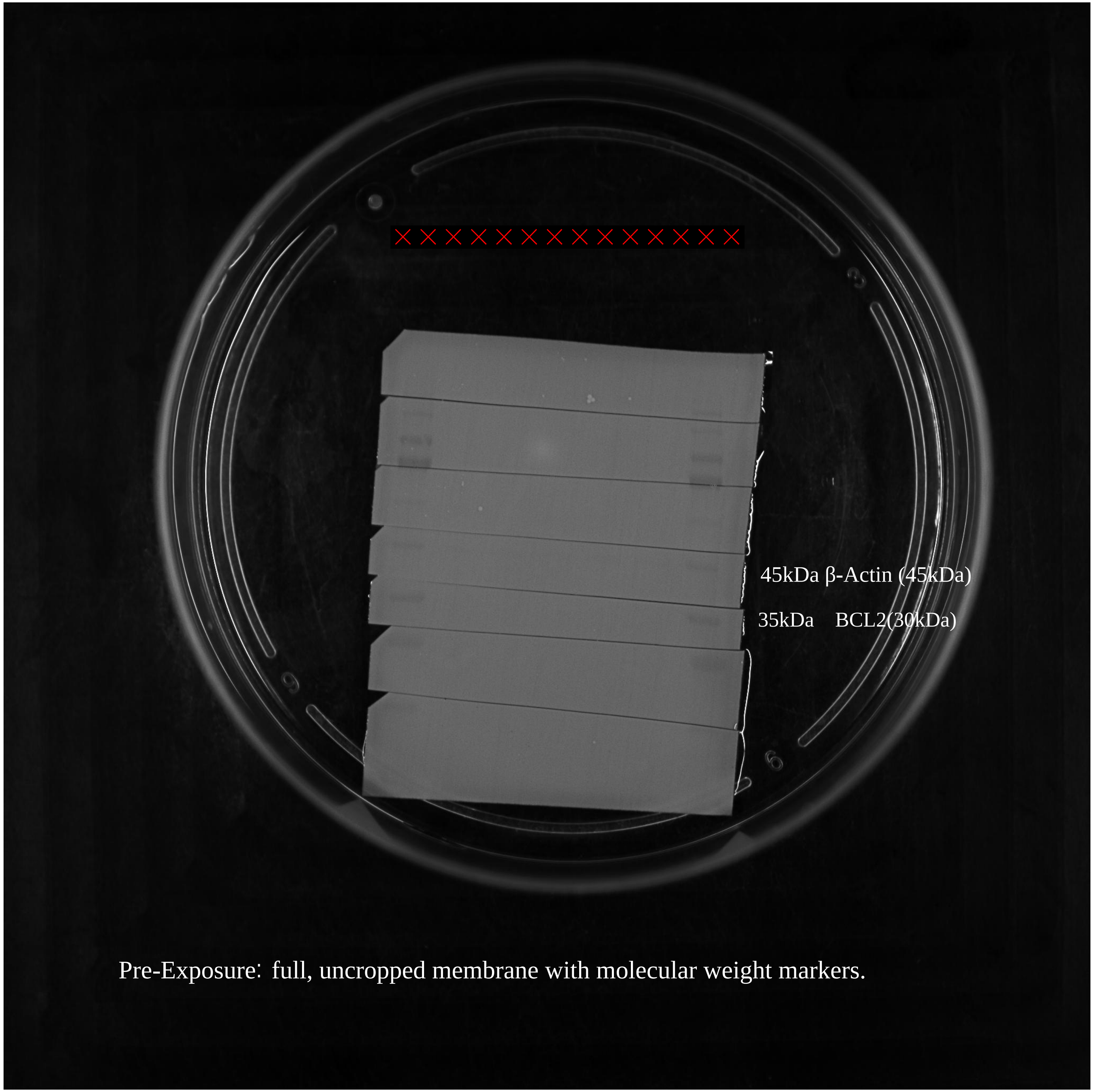

Con Mod NS NS+Ra NS+3-MA

BCL2-2

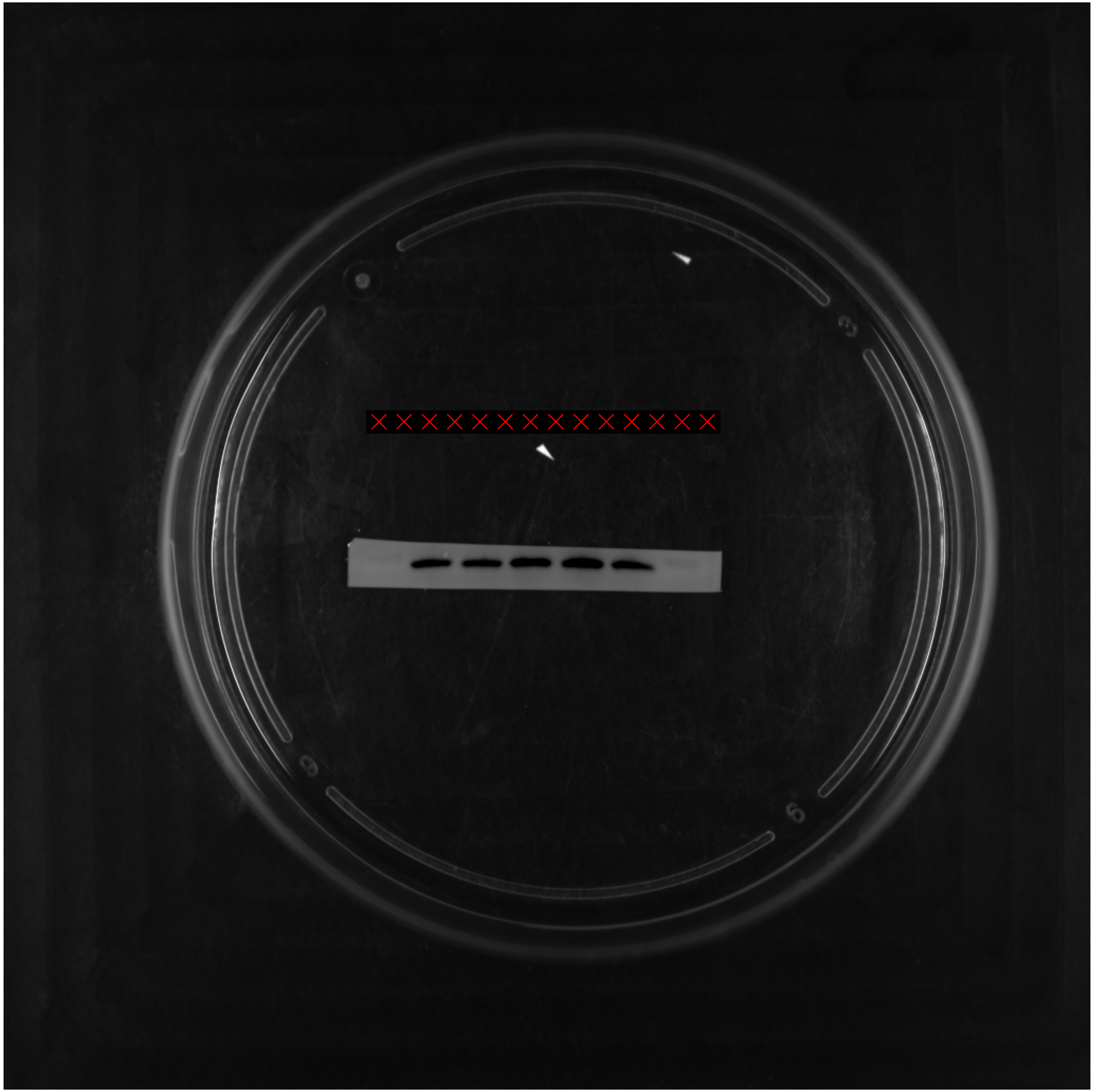

Con Mod NS NS+Ra NS+3-MA

BCL2-2-β-Actin

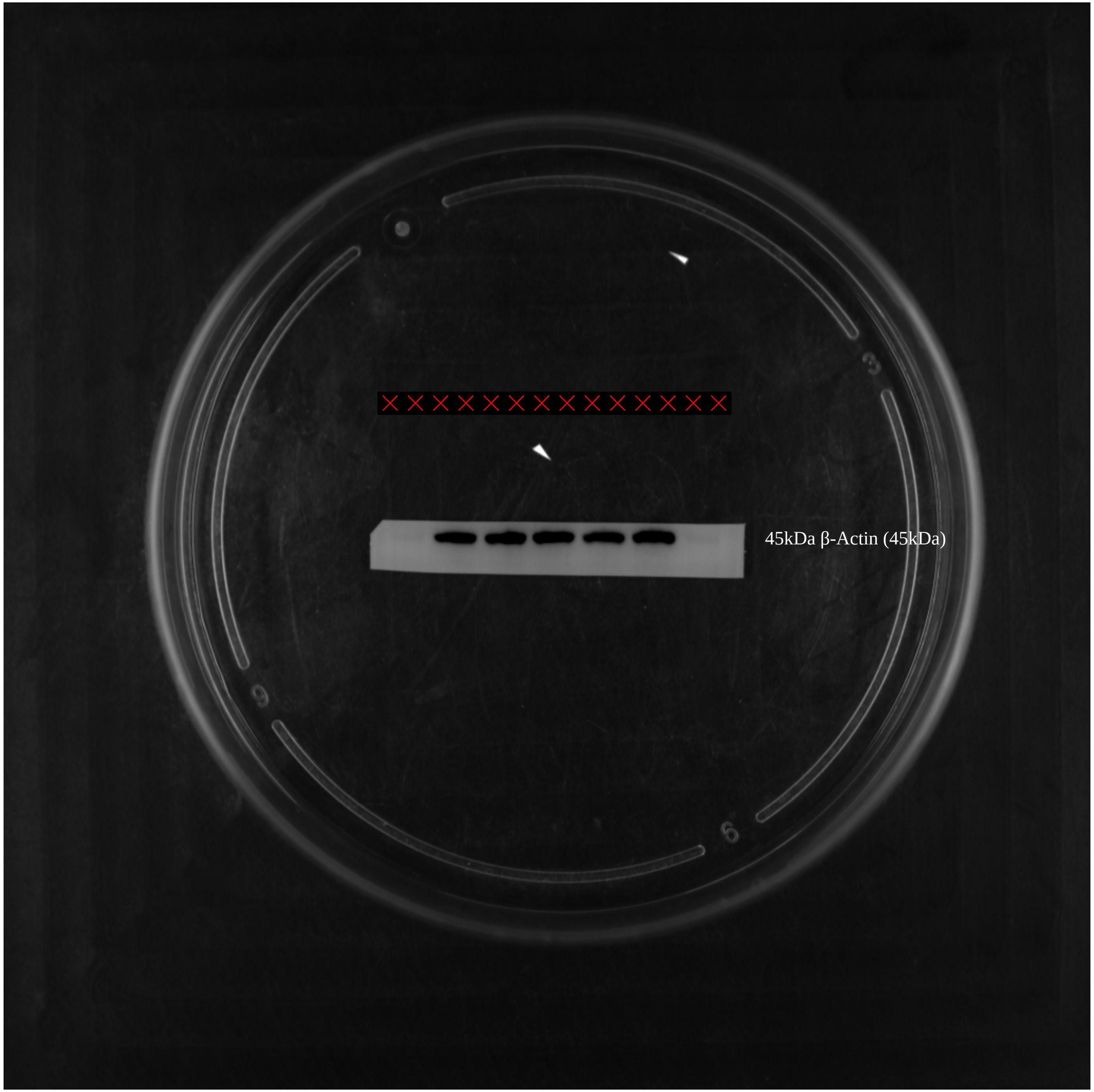

Con Mod NS NS+Ra NS+3-MA

BCL2-3-full

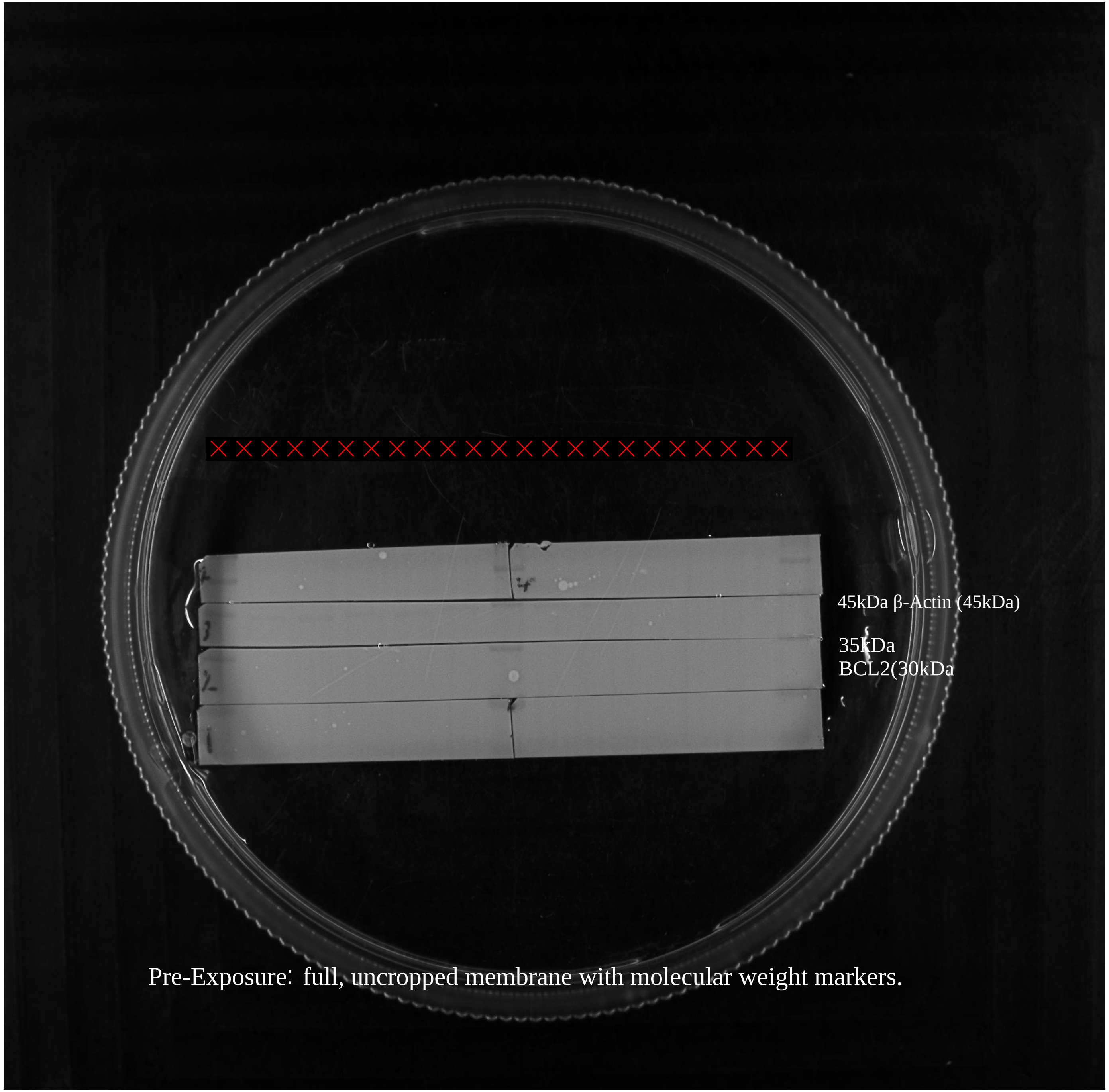

Con Mod NS NS+Ra NS+3-MA Con Mod NS NS+Ra NS+3-MA

BCL2-3

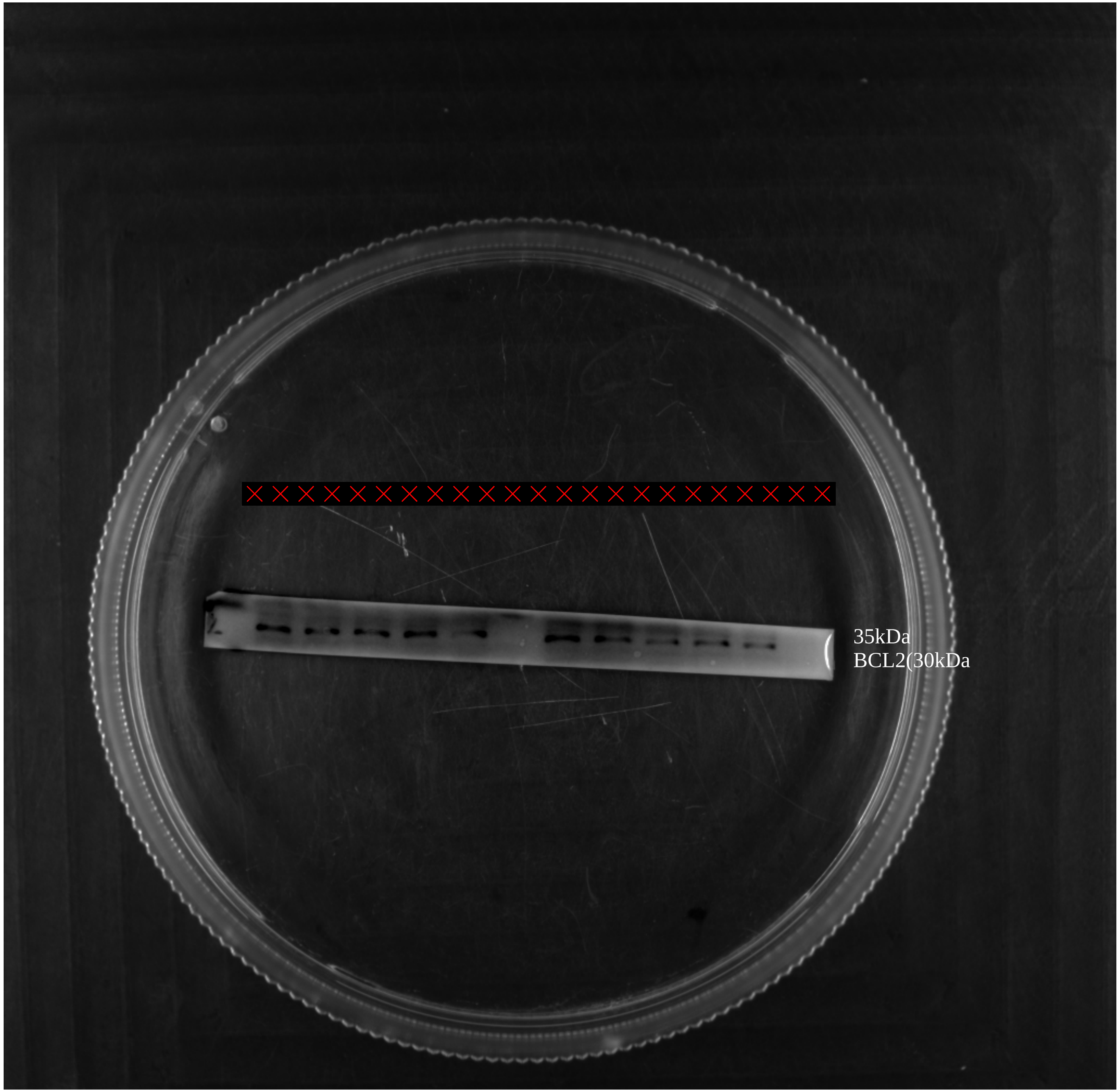

Con Mod NS NS+Ra NS+3-MA Con Mod NS NS+Ra NS+3-MA

BCL2-3-β-Actin

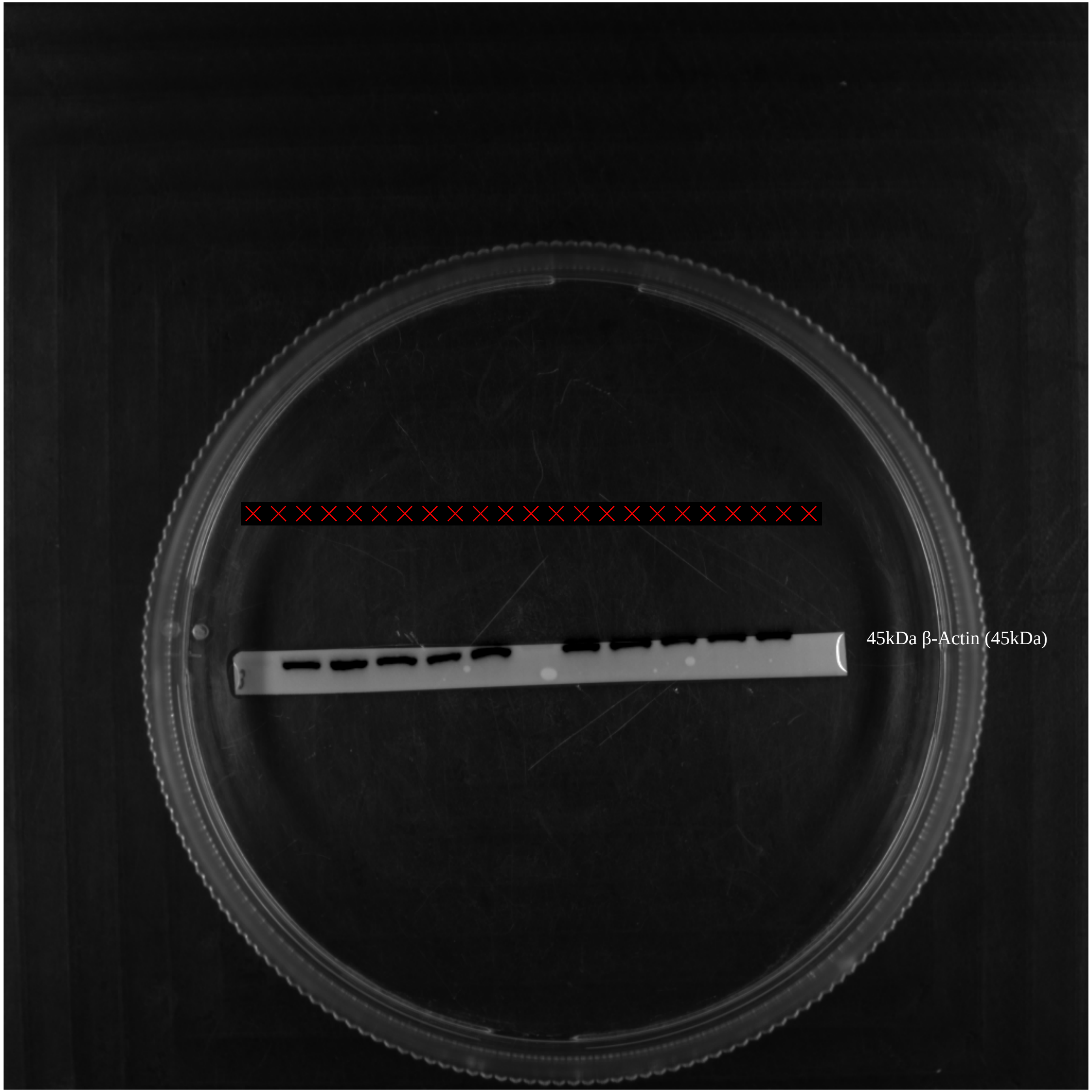

Con Mod NS NS+Ra NS+3-MA Con Mod NS NS+Ra NS+3-MA

Caspase-3-3-full

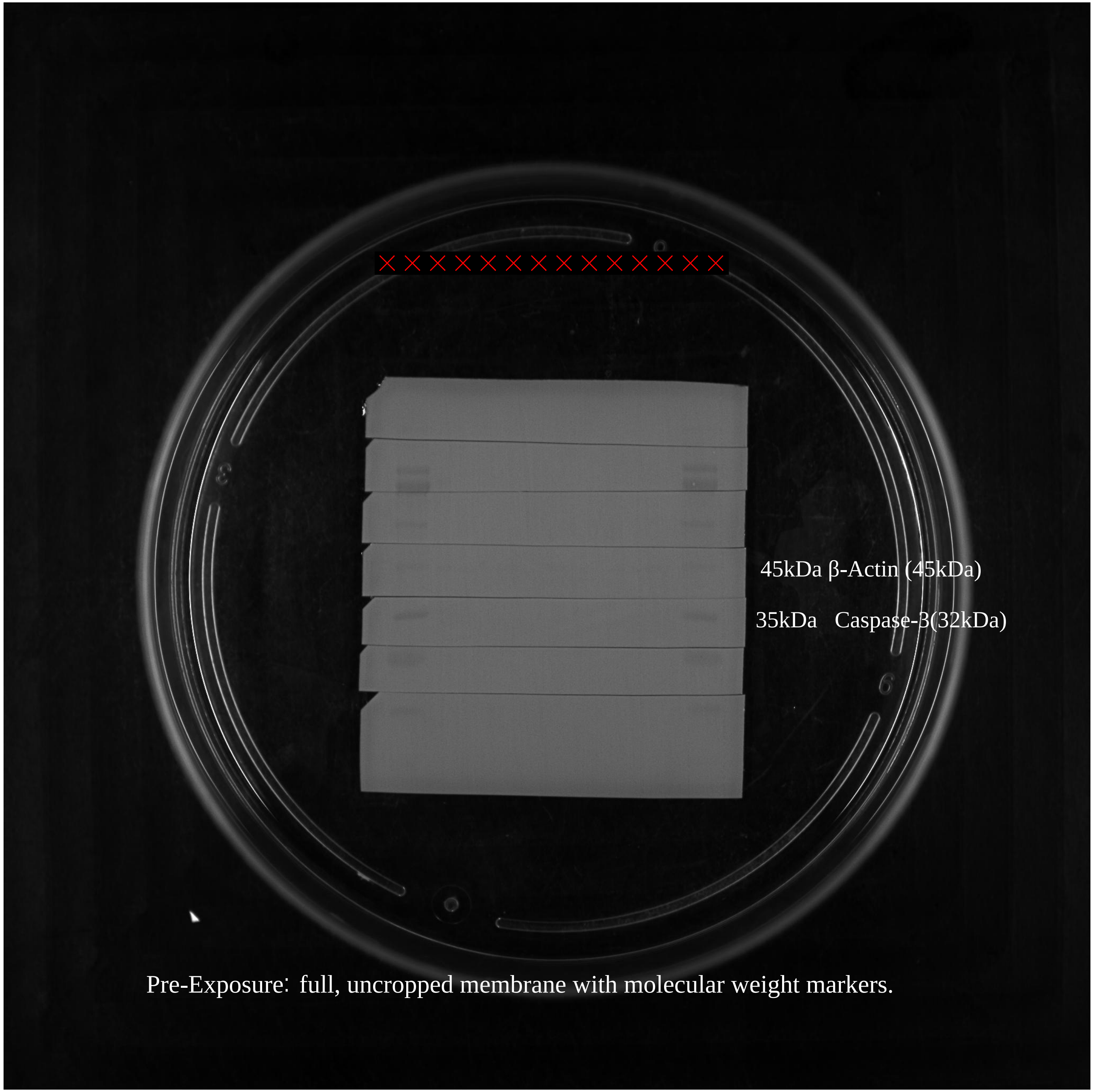

Con Mod NS NS+Ra NS+3-MA

Caspase-3-3

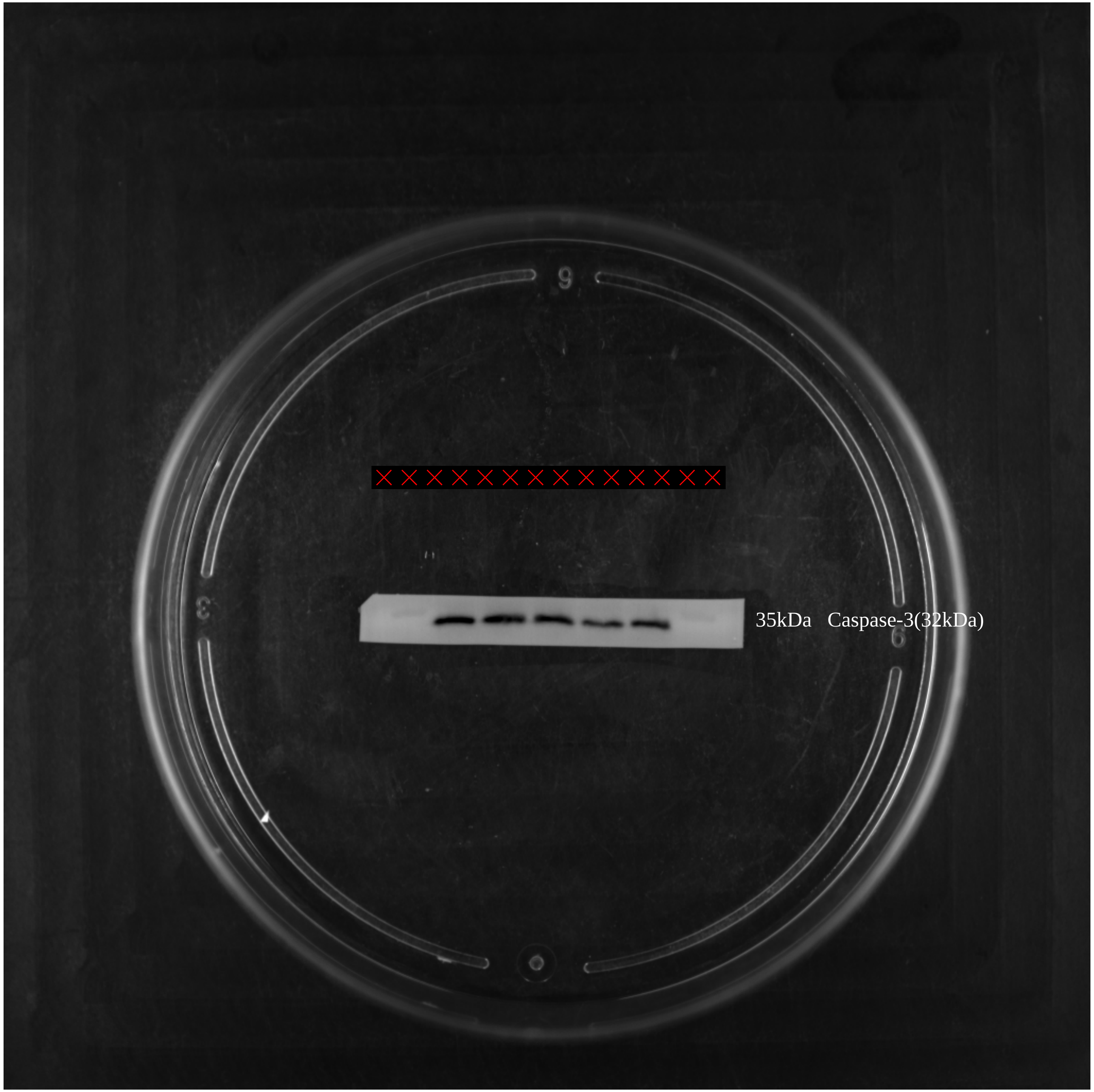

Con Mod NS NS+Ra NS+3-MA

Caspase-3-3-β-Actin

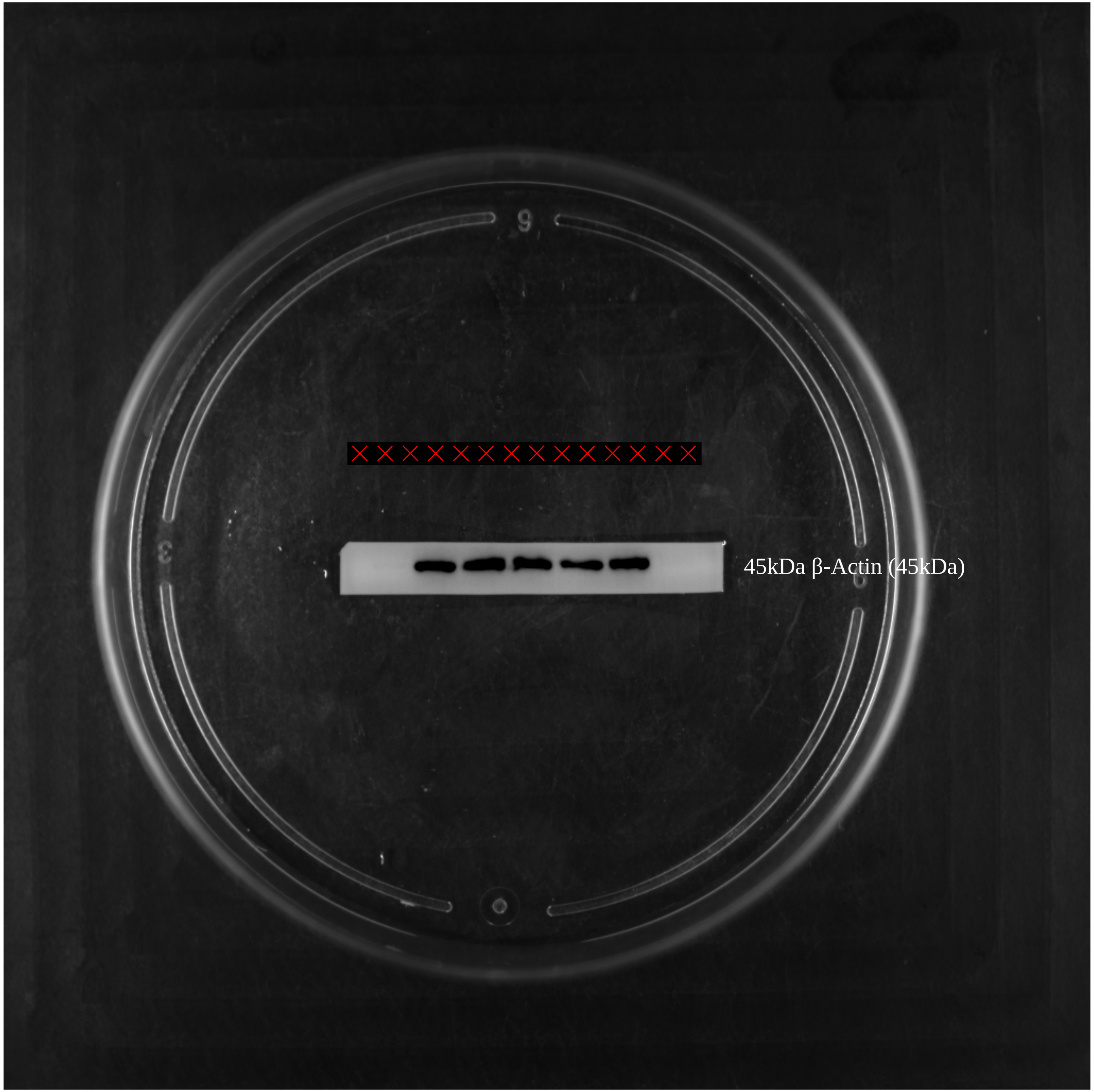

Con Mod NS NS+Ra NS+3-MA

Caspase-3-4-full

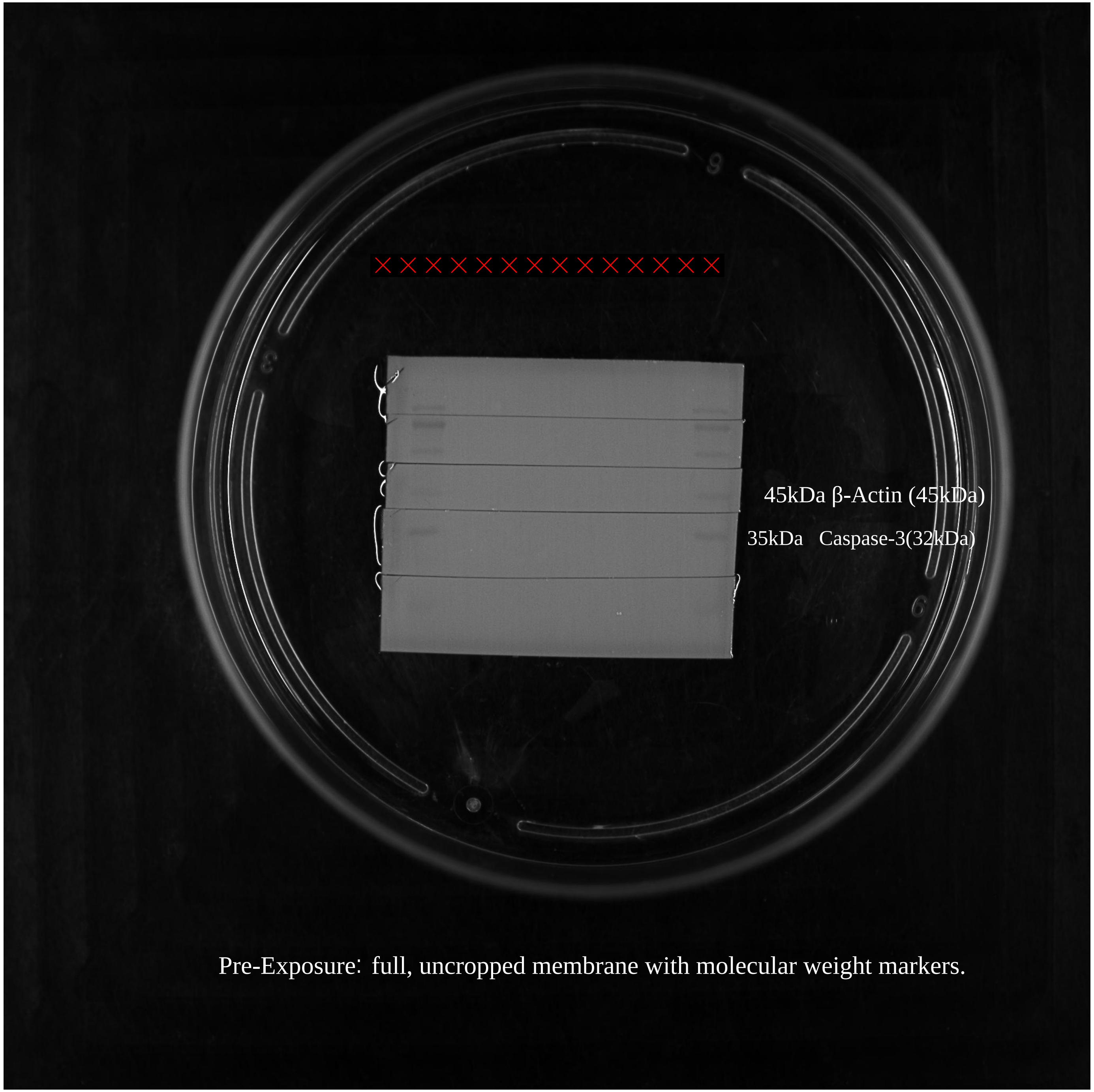

Con Mod NS NS+Ra NS+3-MA

Caspase-3-4

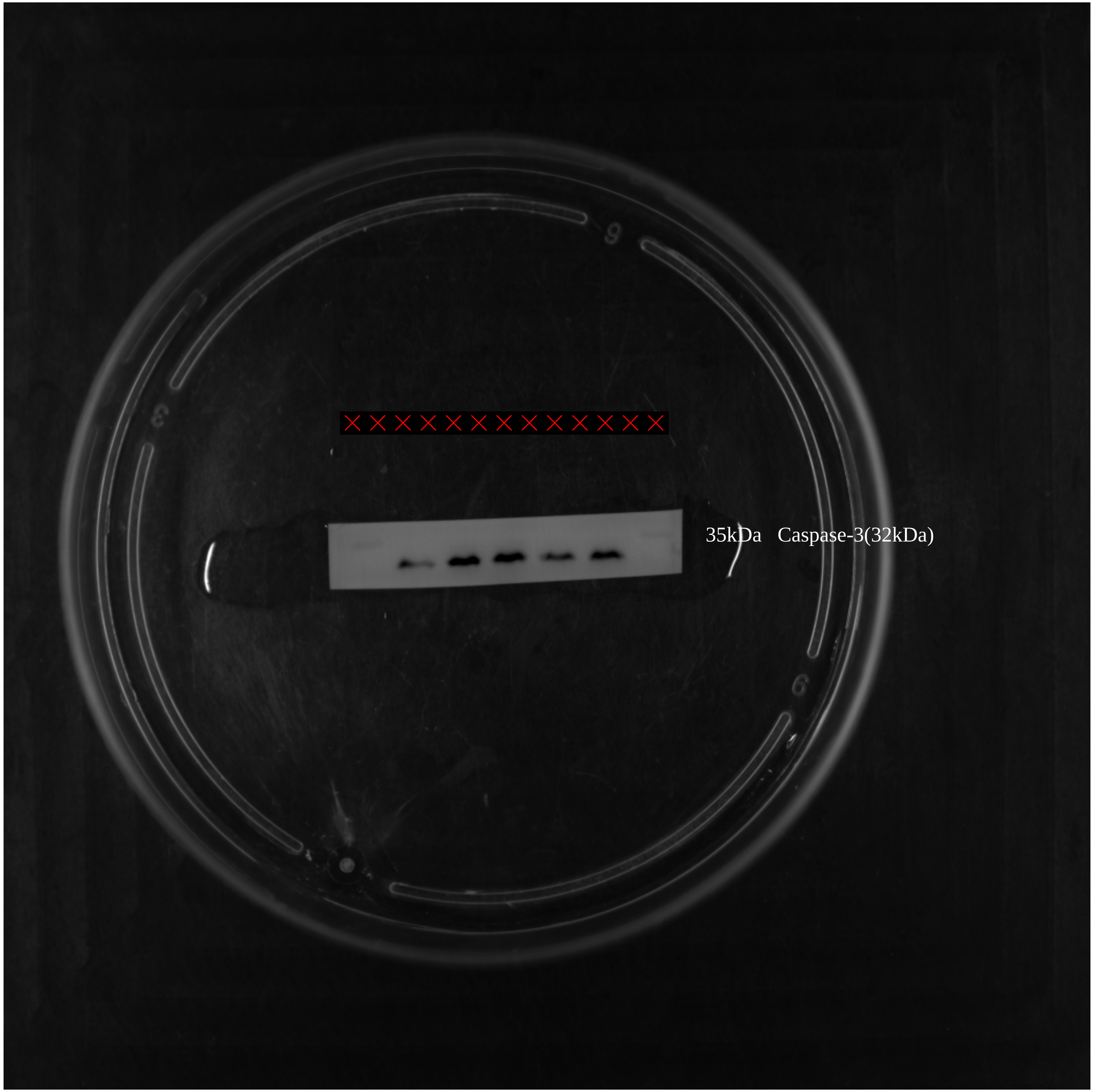

Con Mod NS NS+Ra NS+3-MA

Caspase-3-4-β-Actin

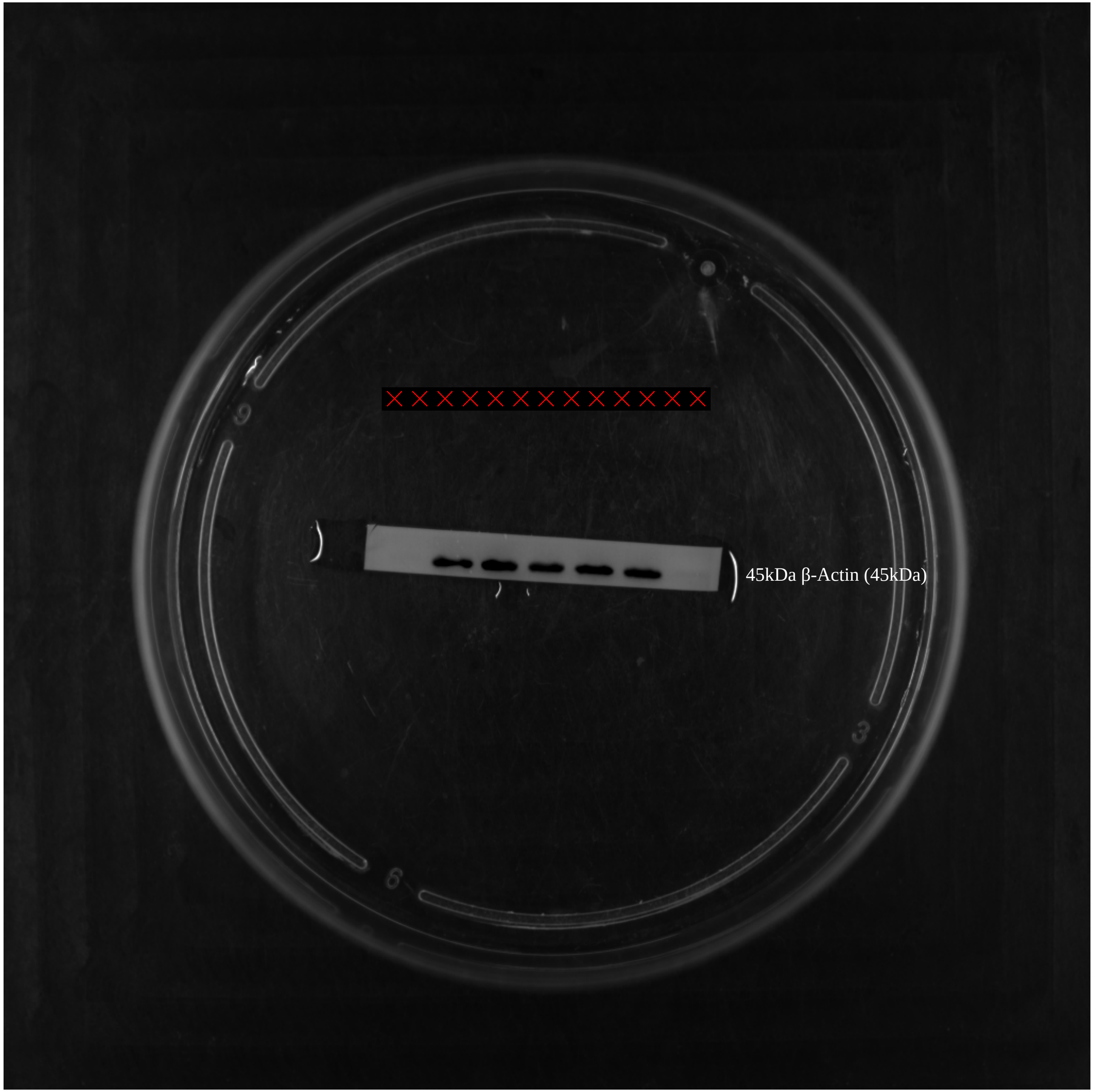

Con Mod NS NS+Ra NS+3-MA

P62-3&4-full

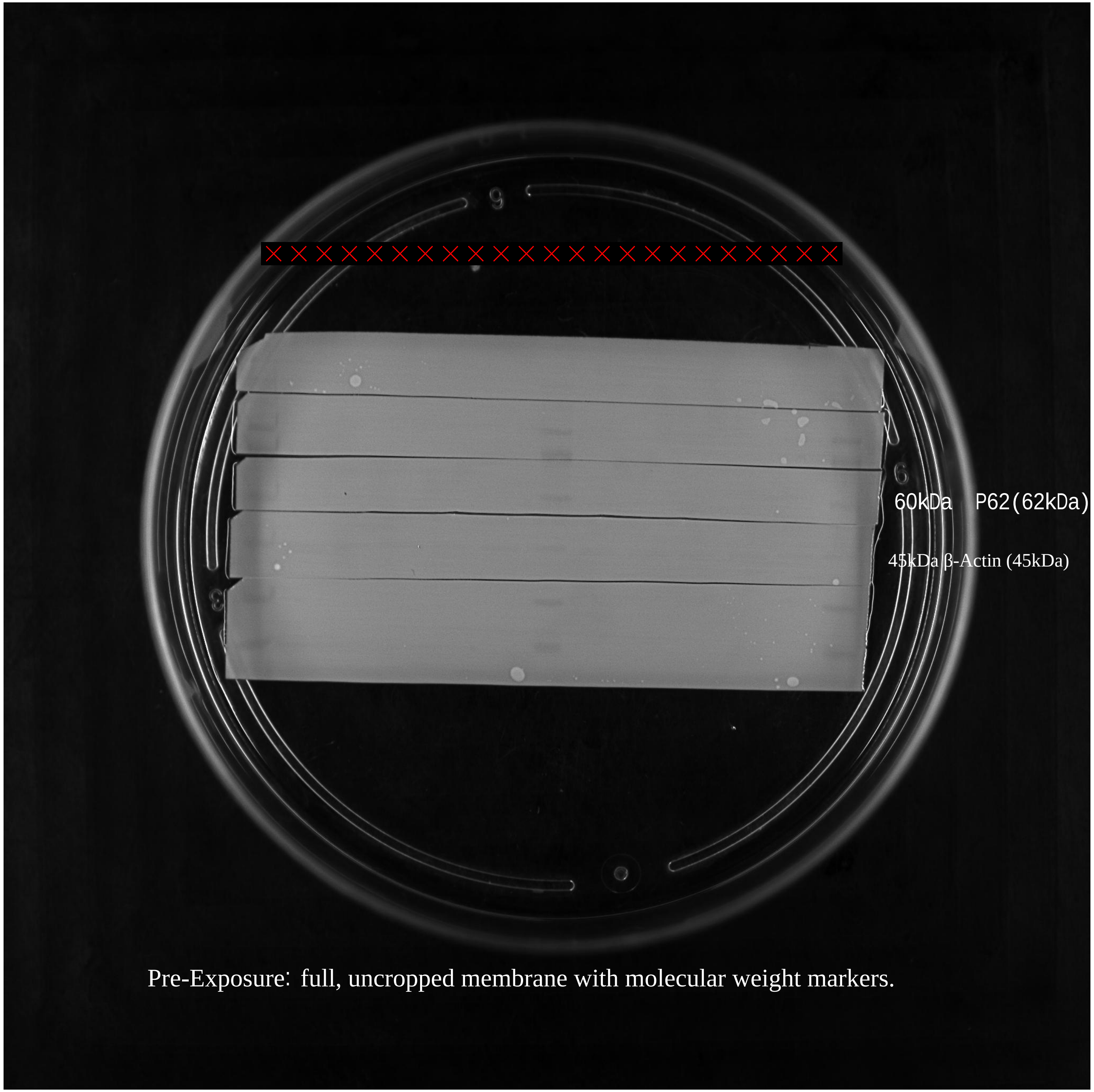

Con Mod NS NS+Ra NS+3-MA Con Mod NS NS+Ra NS+3-MA

P62-3&4

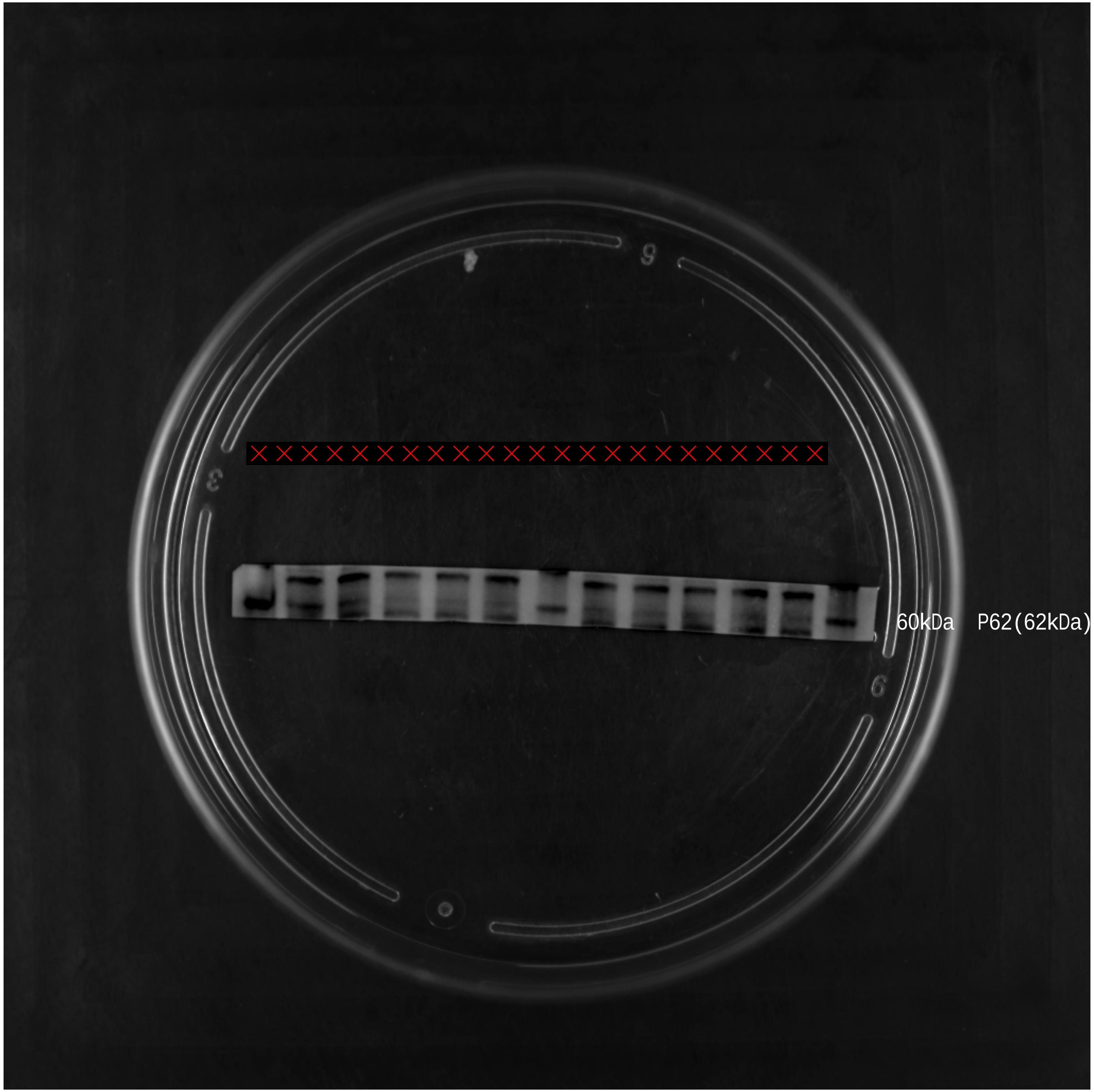

Con Mod NS NS+Ra NS+3-MA Con Mod NS NS+Ra NS+3-MA

P62-3&4-β-Actin

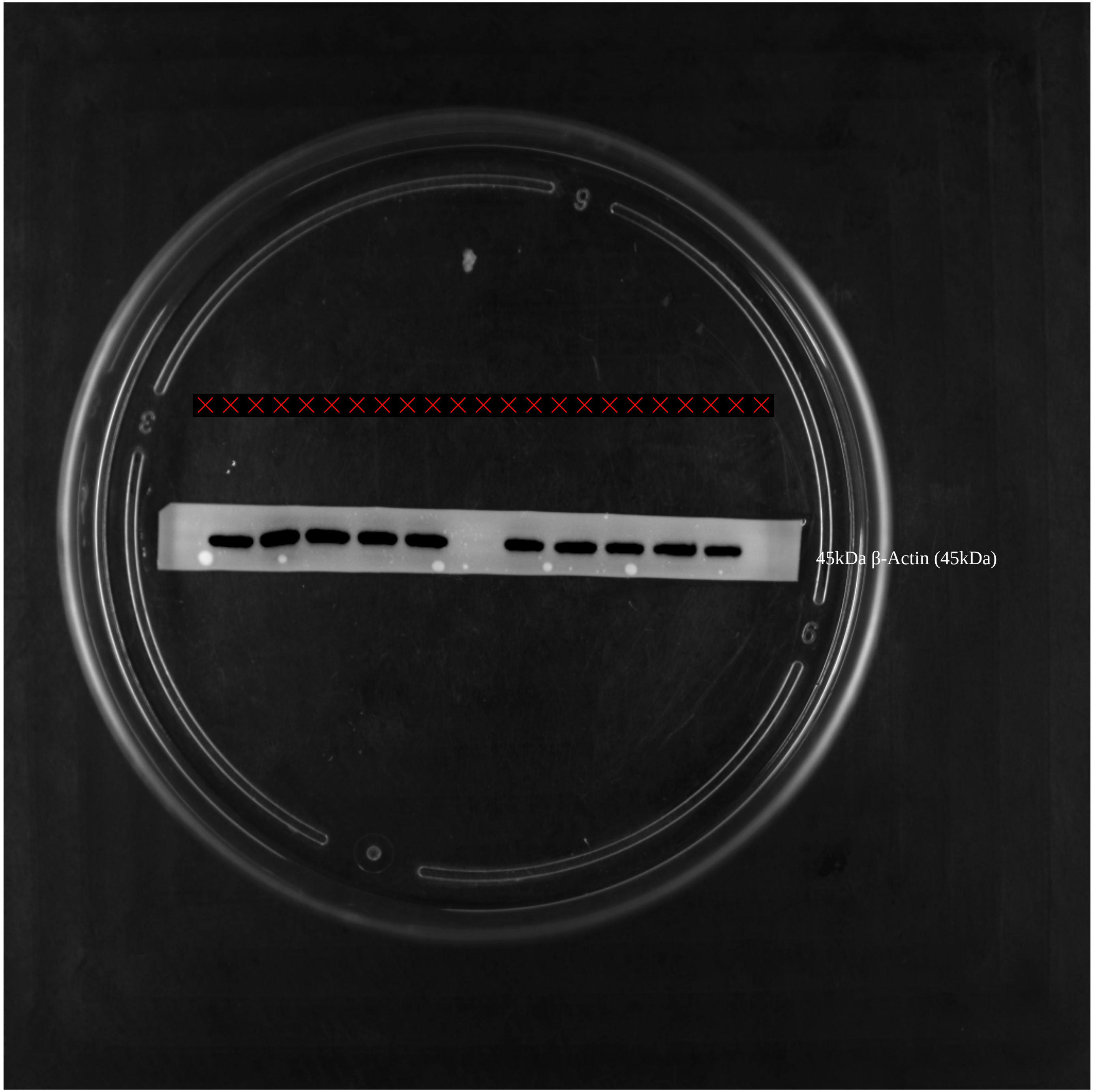

Con Mod NS NS+Ra NS+3-MA Con Mod NS NS+Ra NS+3-MA

Beclin1-2-full

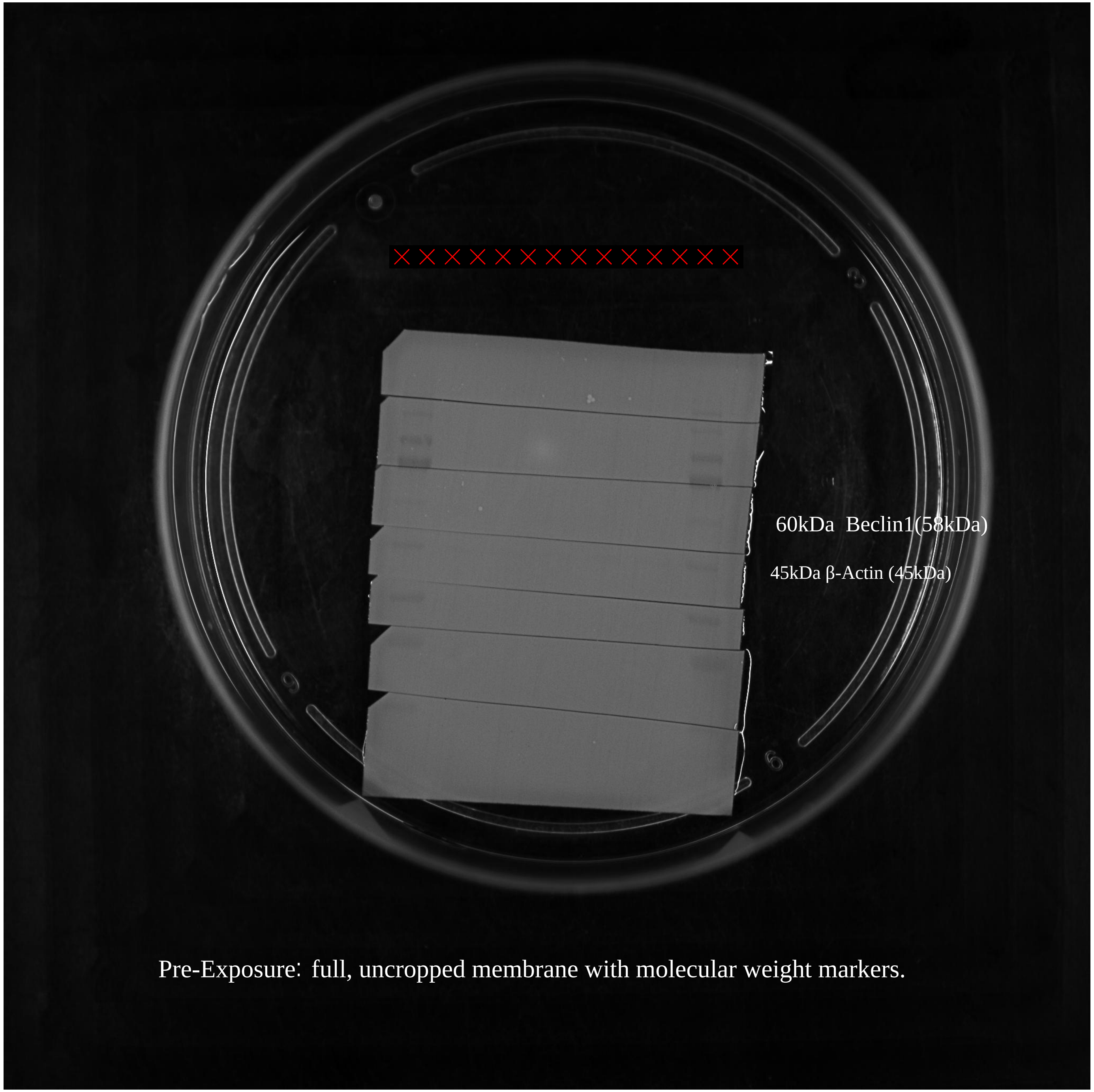

Con Mod NS NS+Ra NS+3-MA

Beclin1-2

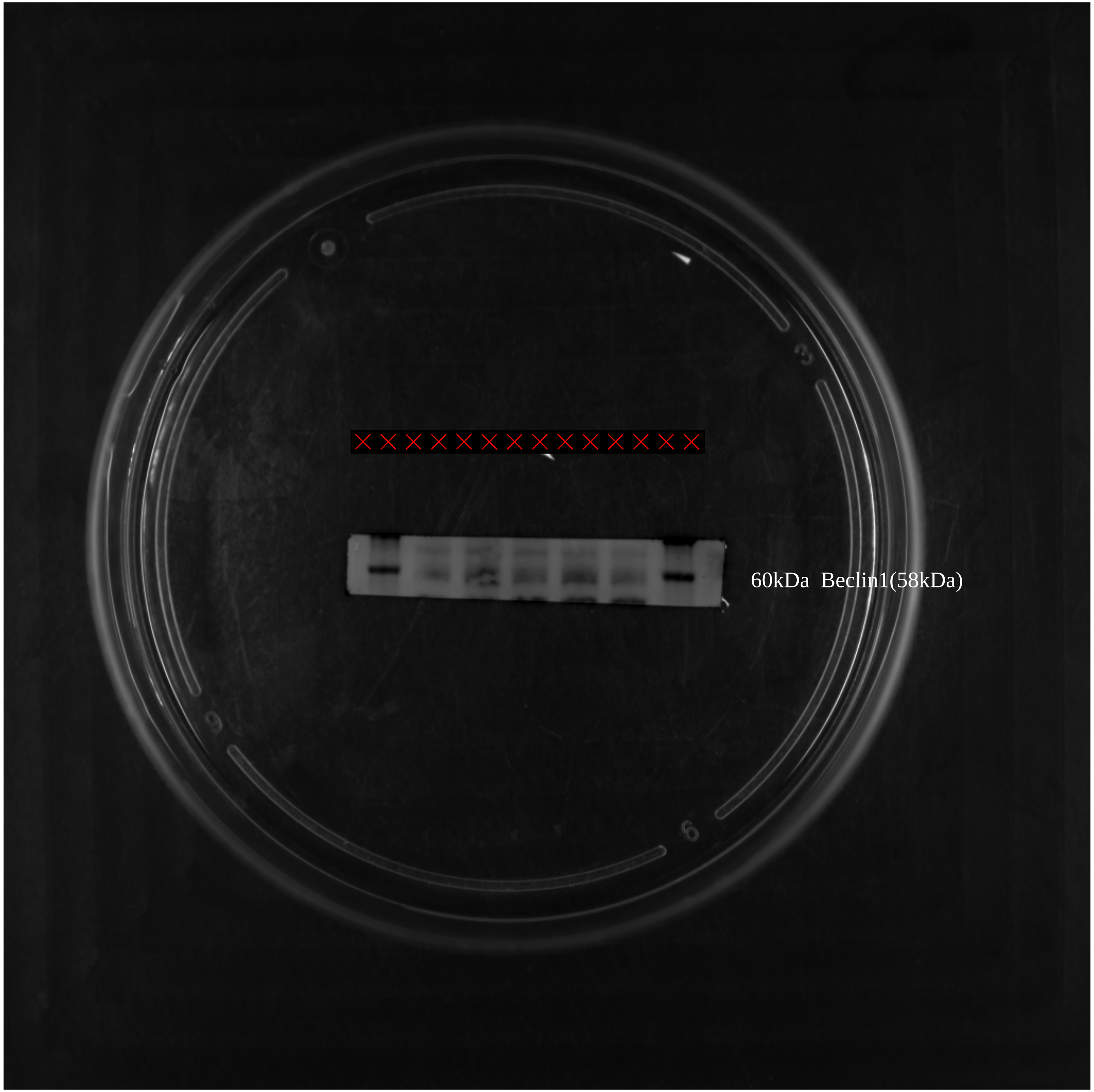

Con Mod NS NS+Ra NS+3-MA

Beclin1-2-β-Actin

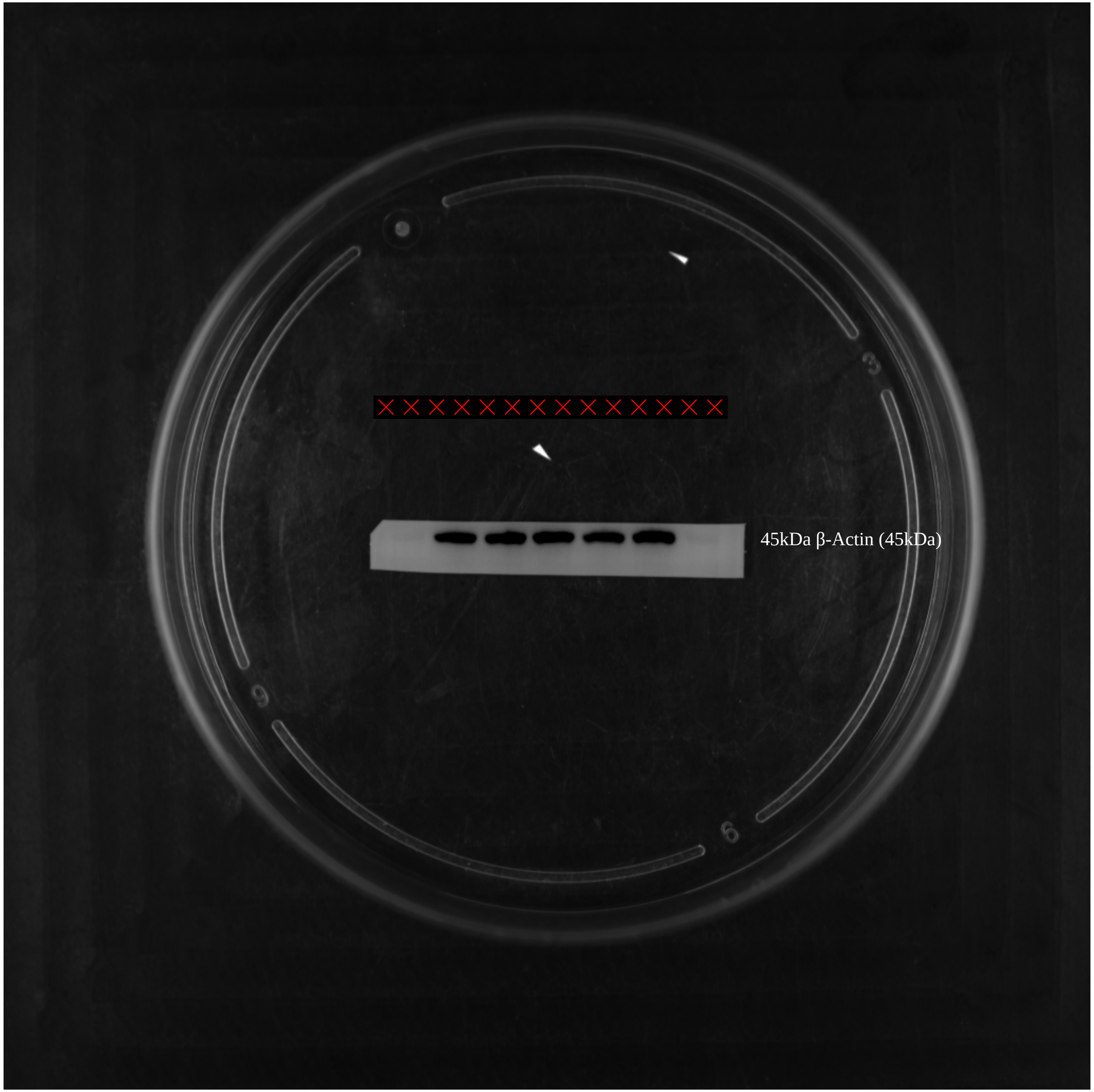

Con Mod NS NS+Ra NS+3-MA

Beclin1-3-full

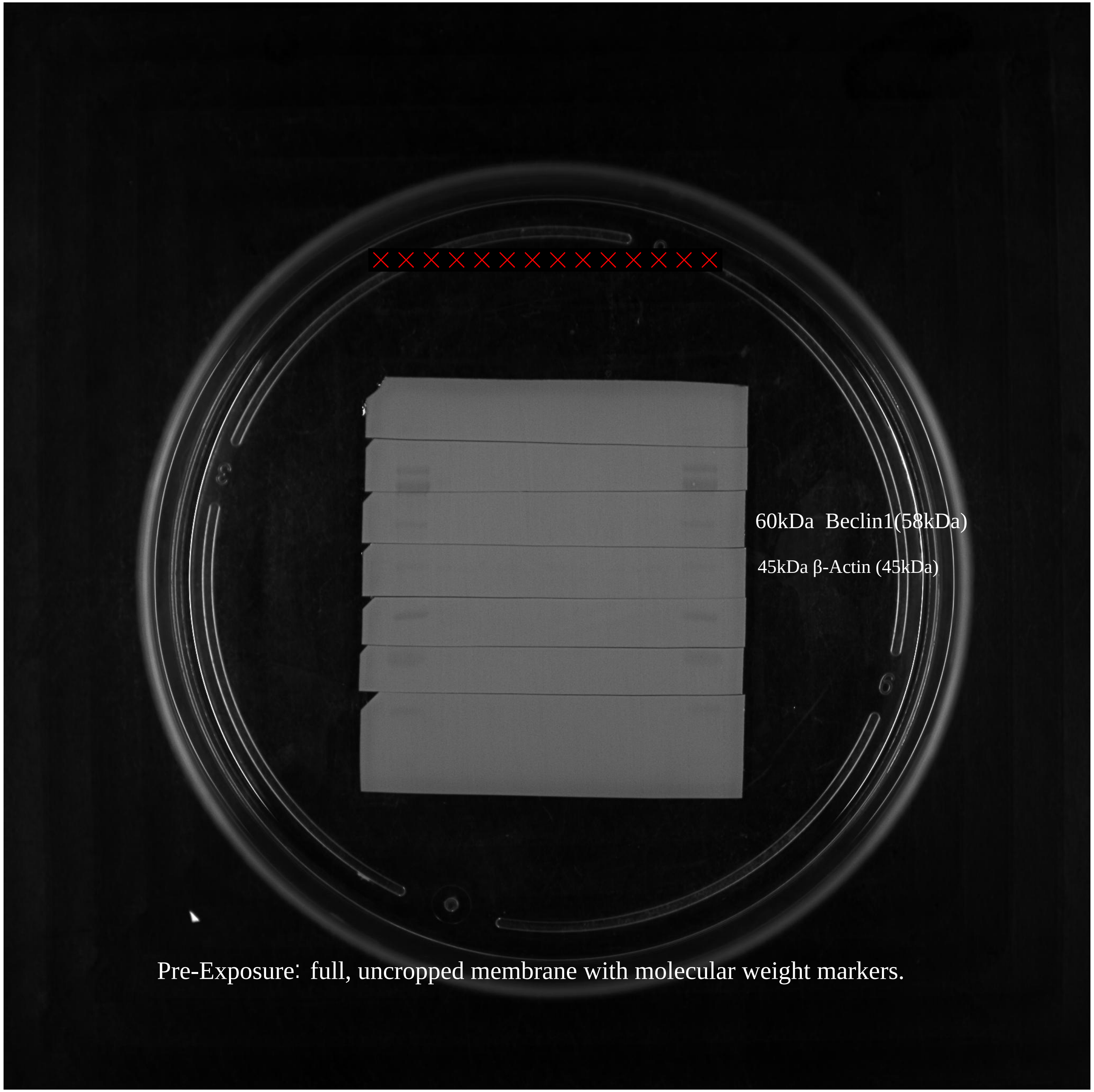

Con Mod NS NS+Ra NS+3-MA

Beclin1-3

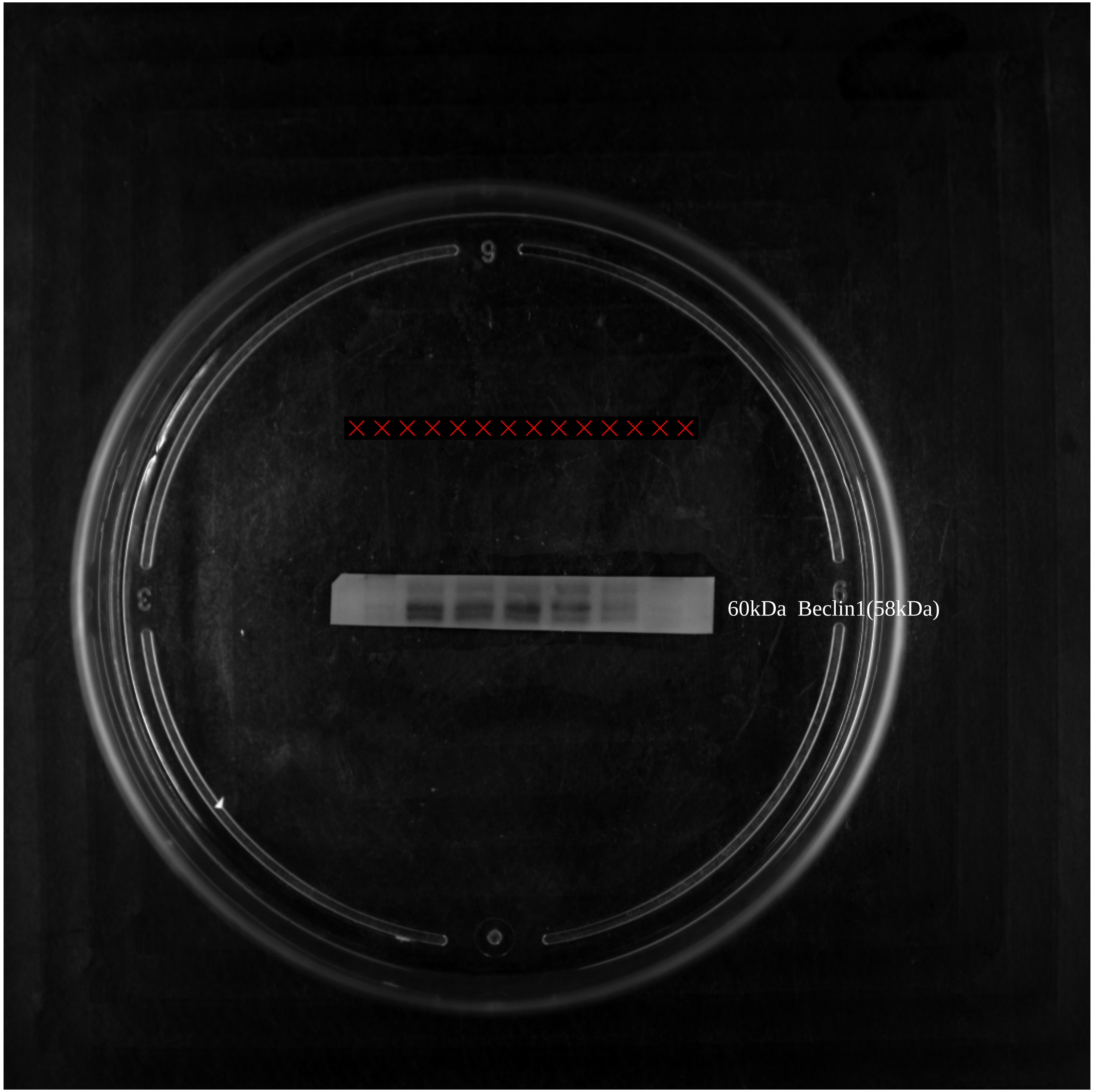

Con Mod NS NS+Ra NS+3-MA

Beclin1-3-β-Actin

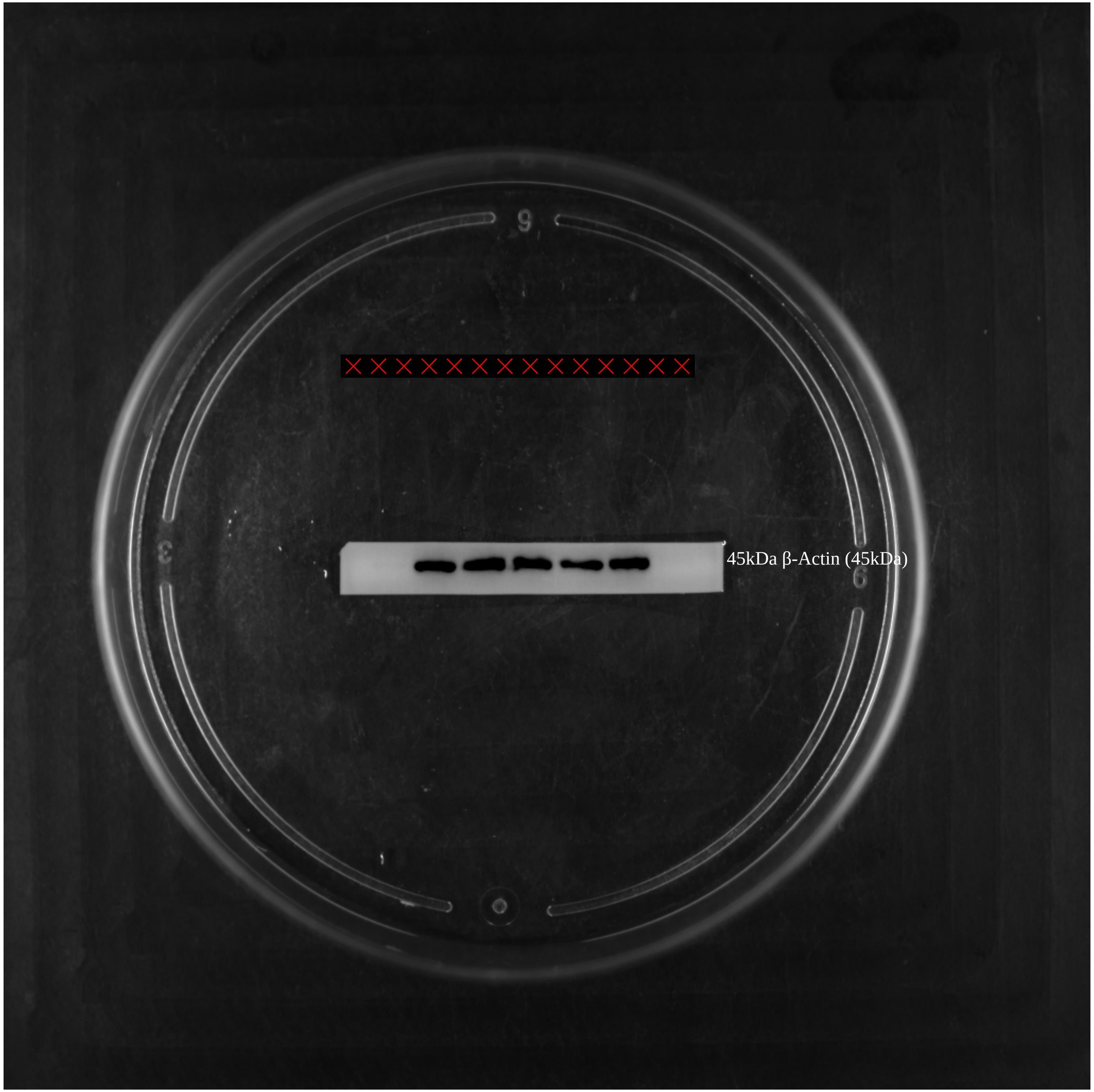

Con Mod NS NS+Ra NS+3-MA

LC3-2-full

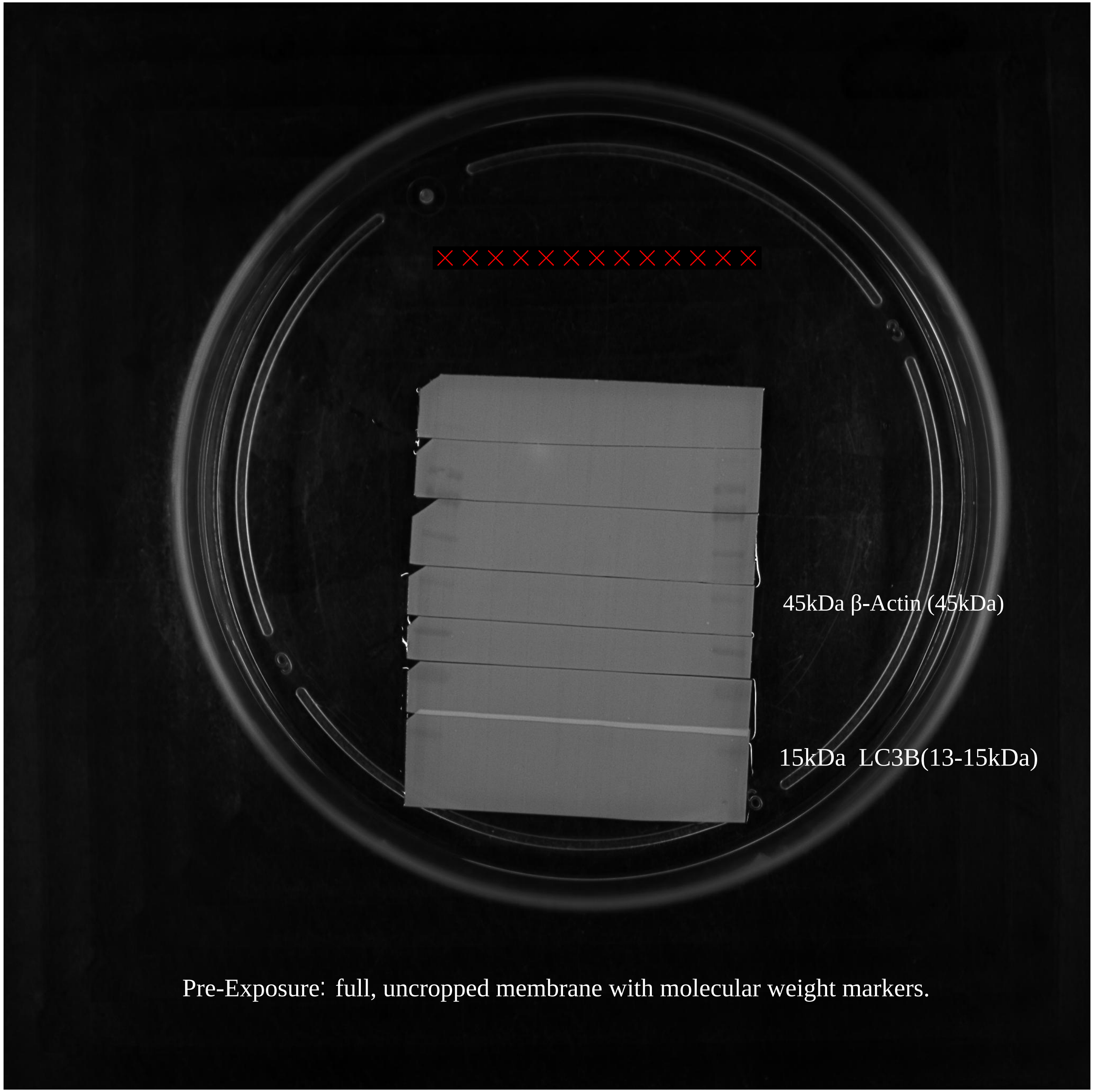

Con Mod NS NS+Ra NS+3-MA

LC3-2

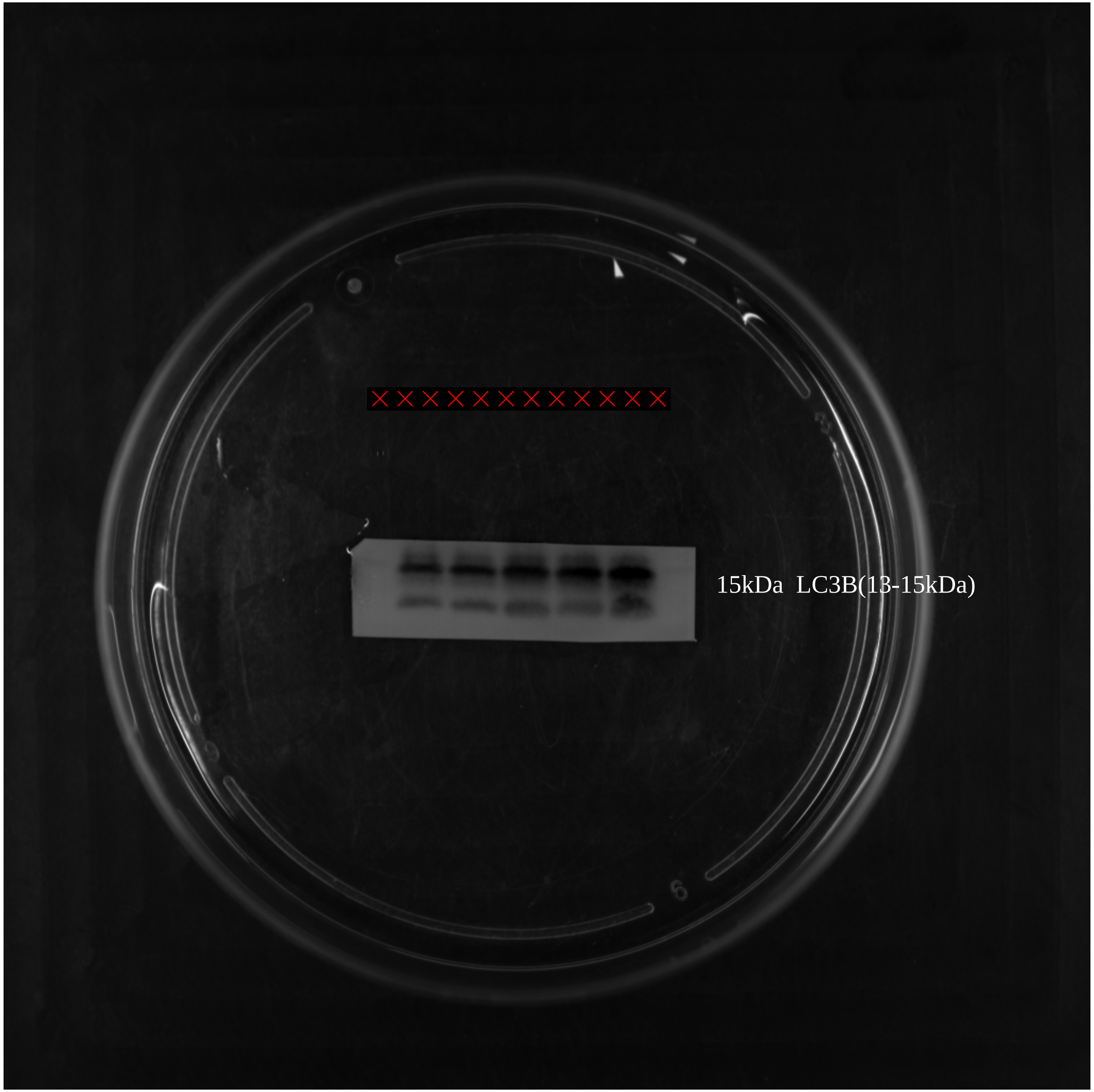

Con Mod NS NS+Ra NS+3-MA

LC3-2-β-Actin

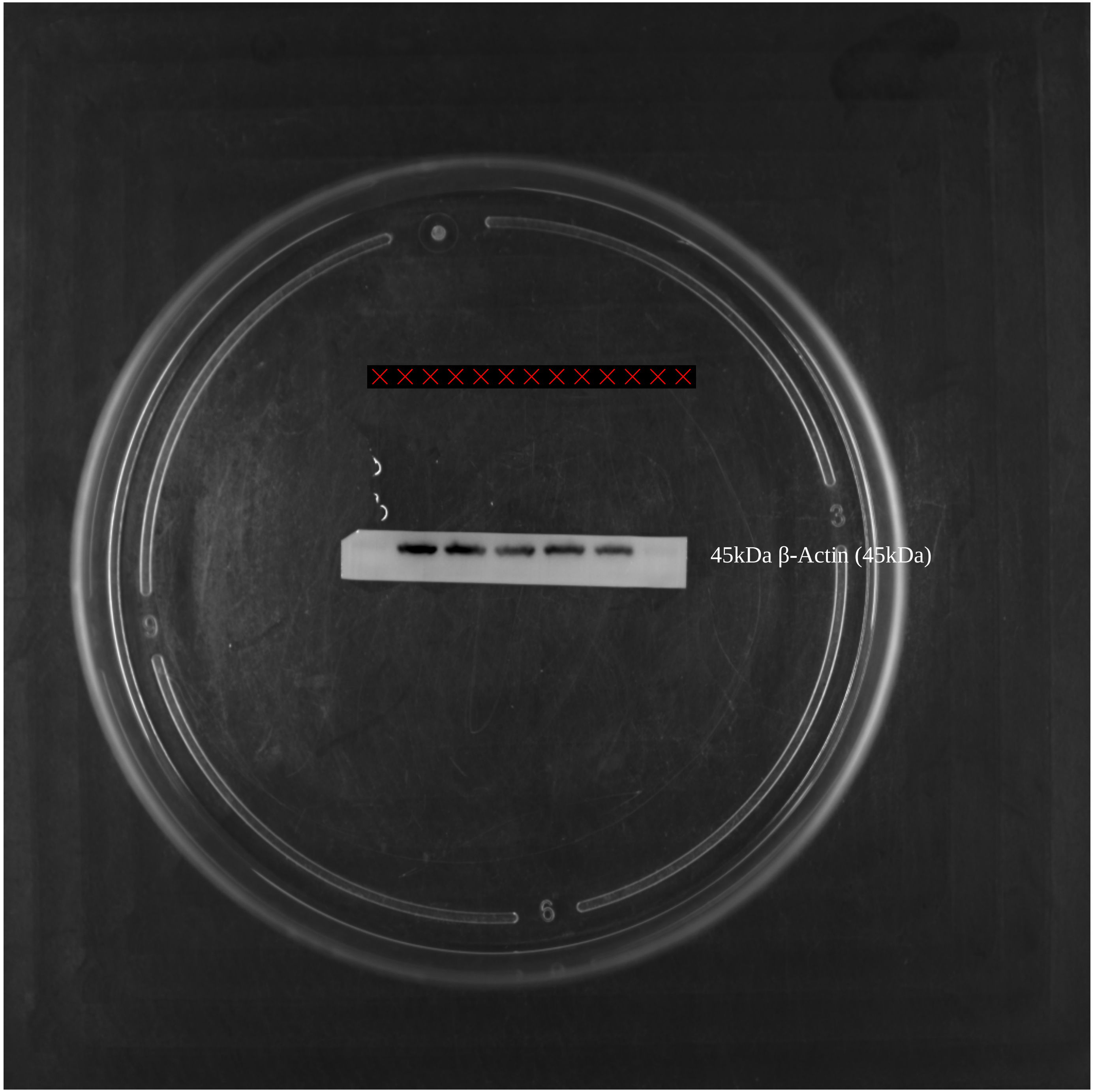

Con Mod NS NS+Ra NS+3-MA

LC3-3-full

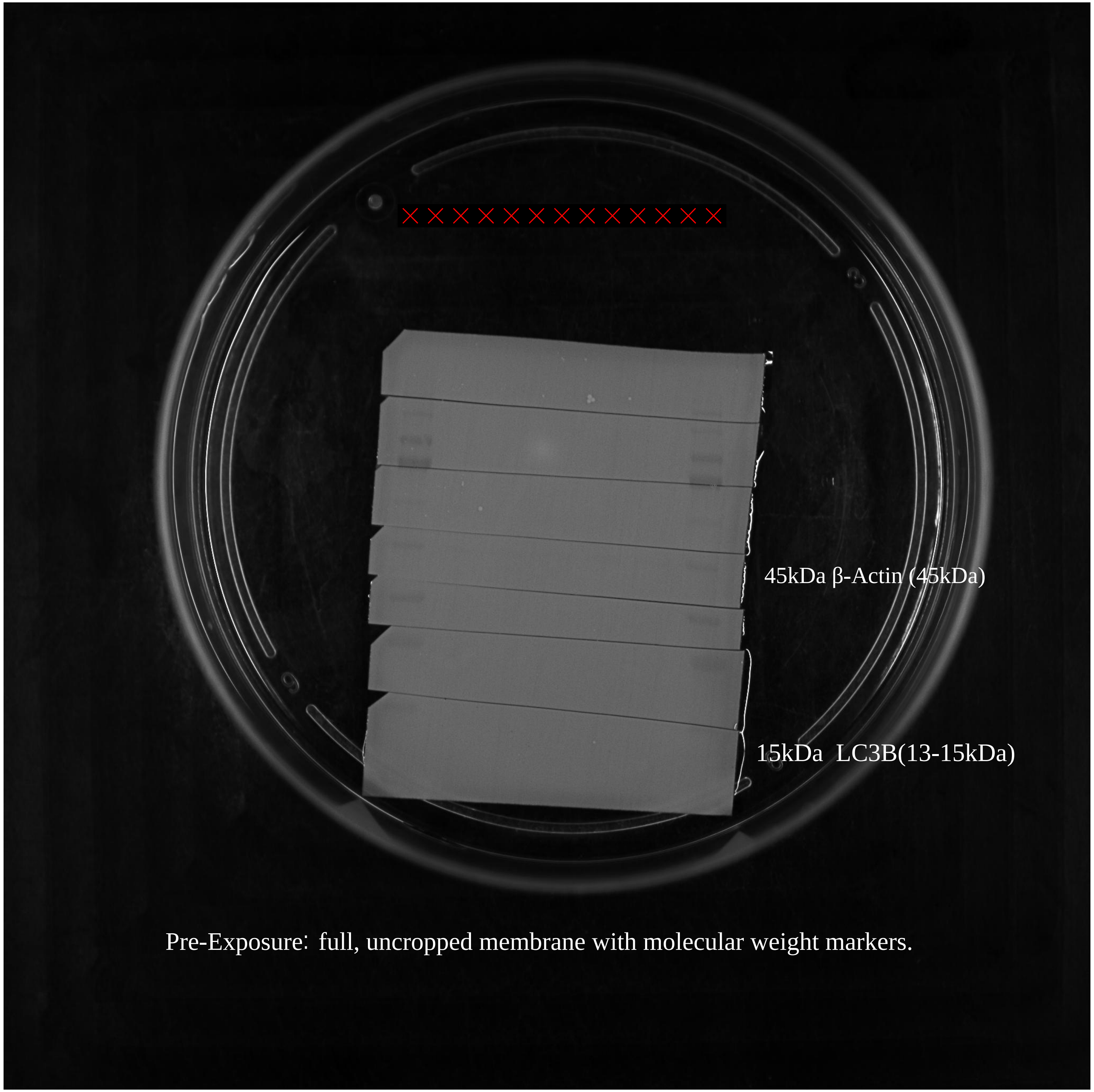

Con Mod NS NS+Ra NS+3-MA

LC3-3

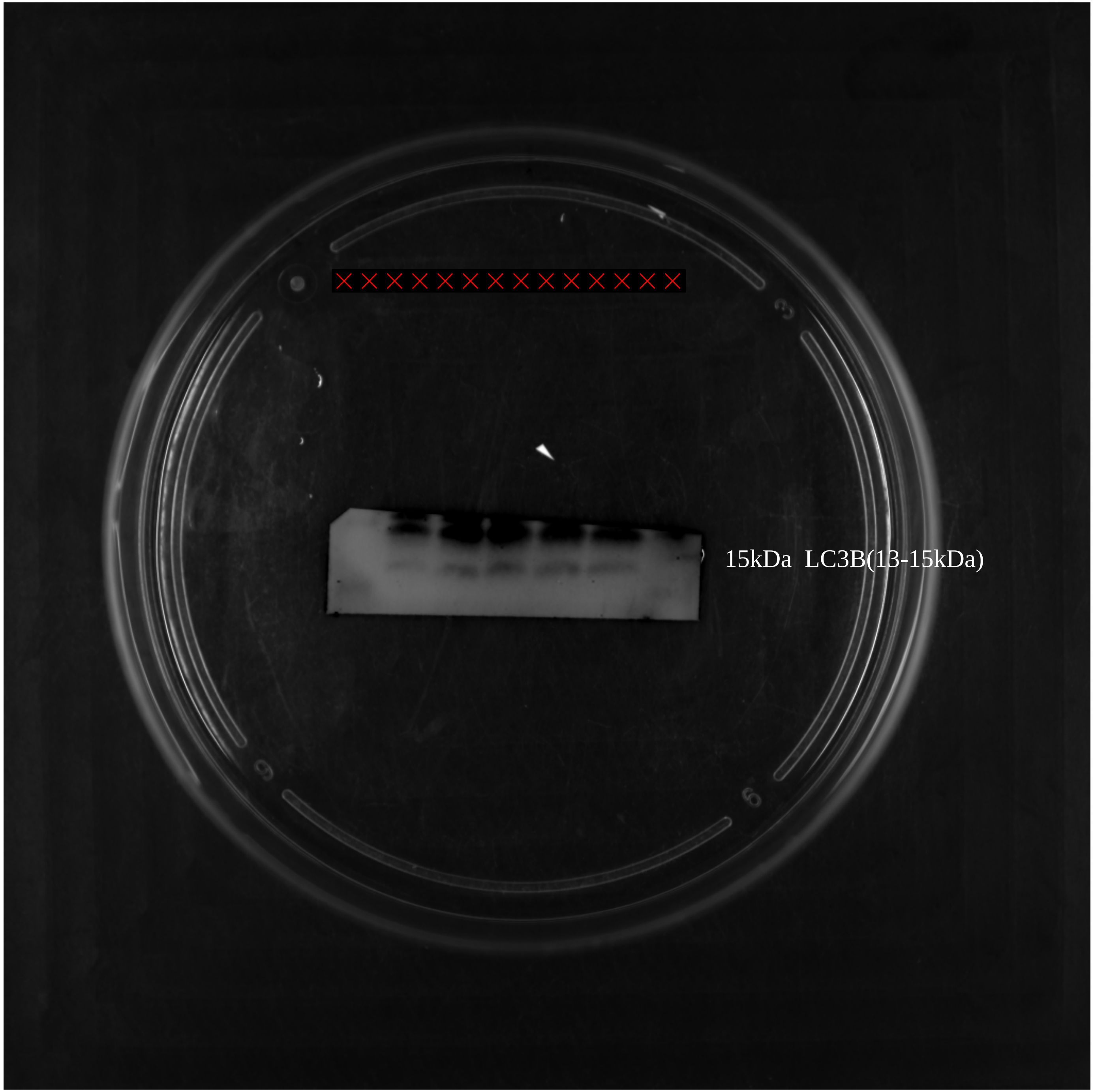

Con Mod NS NS+Ra NS+3-MA

LC3-3-β-Actin

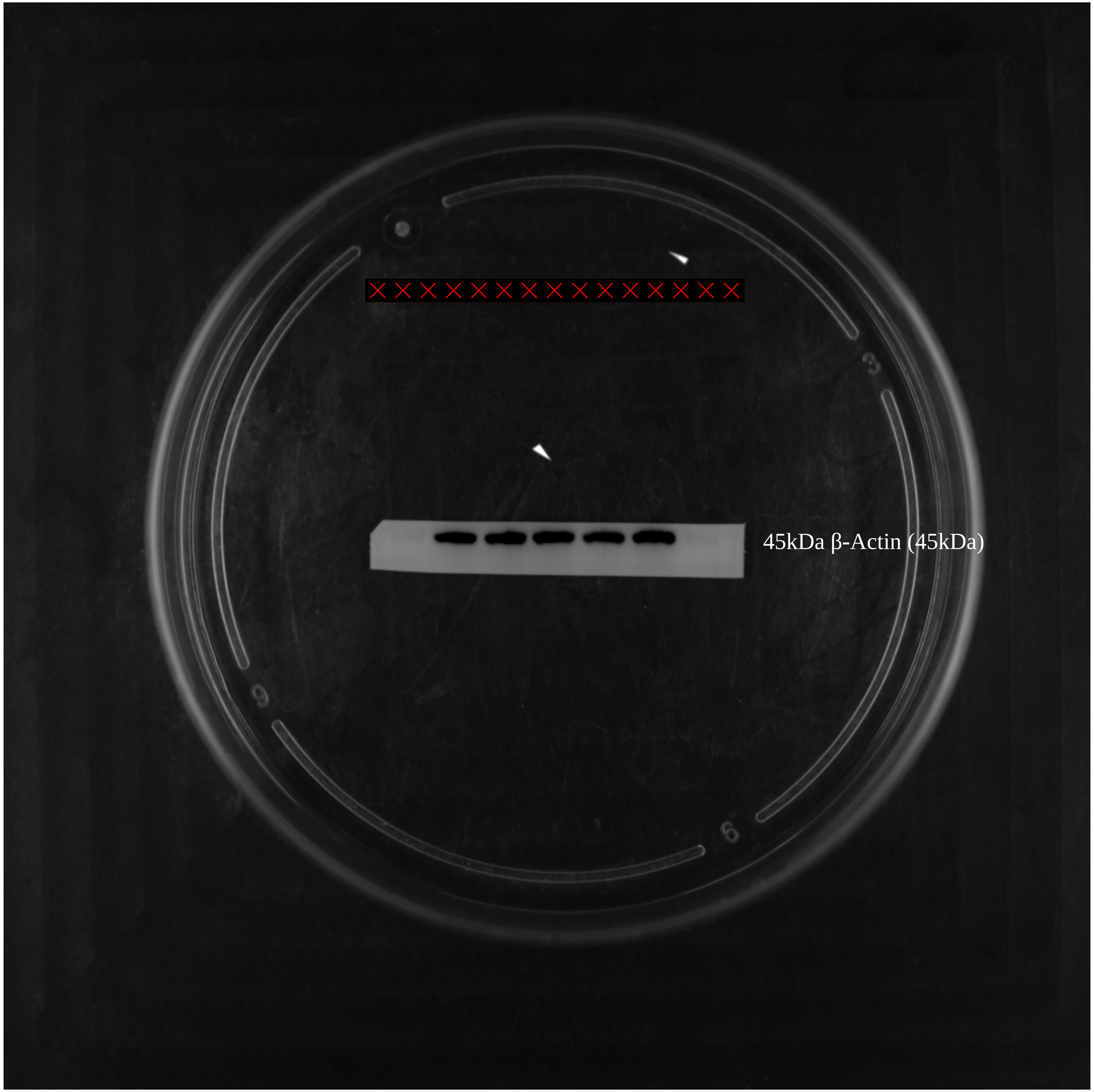

Con Mod NS NS+Ra NS+3-MA
