# Supplementary material for: Synergistic effects of notoginsenoside R1 and saikosaponin B2 in atherosclerosis: A novel approach targeting PI3K/AKT/mTOR pathway and macrophage autophagy
Source: PLoS One. 2025 Jun 27;20(6):e0326687. doi: 10.1371/journal.pone.0326687 (PMC12204583; doi:10.1371/journal.pone.0326687)
Supplement: Attachment — Submitted filename: PONE-D-24-45738_reviewer.pdf [file pone.0326687.s003.pdf]

PLOS ONE

Synergistic Effects of Notoginsenoside R1 and Saikosaponin B2 in Atherosclerosis: A Novel Approach Targeting PI3K/AKT/mTOR Pathway and Macrophage Autophagy

--Manuscript Draft--

**Manuscript Number:**	PONE-D-24-45738
**Article Type:**	Research Article
**Full Title:**	Synergistic Effects of Notoginsenoside R1 and Saikosaponin B2 in Atherosclerosis: A Novel Approach Targeting PI3K/AKT/mTOR Pathway and Macrophage Autophagy
**Short Title:**	Synergistic Effects of Notoginsenoside R1 and Saikosaponin B2 in Atherosclerosis
**Corresponding Author:**	chunyang zhou<br>North Sichuan Medical College [Search North Sichuan Medical College]: North Sichuan Medical University<br>nanchong, CHINA
**Keywords:**	atherosclerosis; notoginsenoside R1; Saikosaponin B2; autophagy; PI3K/AKT/mTOR Signaling Pathway
**Abstract:**	Atherosclerosis (AS) is a significant global contributor to cardiovascular diseases and related mortalities. Although traditional treatments are effective, they often come with side effects. Recent studies have highlighted the potential protective properties of saponin compounds derived from traditional herbal sources, such as notoginsenoside R1 (NGR1) and saikosaponin B2 (SSB2), in combating AS. However, the comprehensive effects and underlying mechanisms of these compounds remain inadequately understood. This study aims to characterize the impact and mechanisms of the NGR1 and SSB2 combination (NS) on atherosclerosis. Our results demonstrated that the NS combination ameliorates lipid levels, lipid transport, and unstable plaque formation in ApoE-/- mice on a western high-fat diet. Using network pharmacology, we found that the NS combination markedly influences inflammation, apoptosis, and autophagy, implicating crucial proteins such as caspase-3 and mTOR.. In vitro assays demonstrated that NS effectively inhibits macrophage lipid uptake while downregulating NLRP3 inflammasome-related proteins and genes. Notably, NS also mitigated oxidative stress and apoptosis markers, including Bax, Bcl2, and caspase-3, in macrophages. Both in vitro and in vivo studies revealed that NS inhibited the PI3K/AKT/mTOR signaling pathway, thereby activating macrophage autophagy. Collectively, these findings suggest that the NS combination enhances protection against atherosclerosis by inhibiting macrophage inflammation and apoptosis, regulating the PI3K/AKT/mTOR signaling pathway, and activating macrophage autophagy. This work paves the way for innovative therapeutic strategies against atherosclerosis, potentially transforming current treatment paradigms.
**Order of Authors:**	chunyang zhou
	Yihua Wang
	Qing Liao
	Xue Mei
	Han Xu
	Lijun Luo
	Rong Huang
	Yue Tang
**Additional Information:**	
**Question**	**Response**
**Financial Disclosure**<br><br>Enter a financial disclosure statement that	Yes

Powered by Editorial Manager® and ProduXion Manager® from Aries Systems Corporation

describes the sources of funding for the work included in this submission. Review the submission guidelines for detailed requirements. View published research articles from *PLOS ONE* for specific examples.<br><br>This statement is required for submission and **will appear in the published article** if the submission is accepted. Please make sure it is accurate.<br><br>**Funded studies**<br>Enter a statement with the following details:<br>• Initials of the authors who received each award<br>• Grant numbers awarded to each author<br>• The full name of each funder<br>• URL of each funder website<br>• Did the sponsors or funders play any role in the study design, data collection and analysis, decision to publish, or preparation of the manuscript?<br><br>Did you receive funding for this work?	
Please add funding details.<br>as follow-up to "**Financial Disclosure**<br><br>Enter a financial disclosure statement that describes the sources of funding for the work included in this submission. Review the submission guidelines for detailed requirements. View published research articles from *PLOS ONE* for specific examples.<br><br>This statement is required for submission and **will appear in the published article** if the submission is accepted. Please make sure it is accurate.	1.The Scientific Startup Foundation for Doctors of North Sichuan Medical College(CBY23-QDA11) to XM;<br>2.Key Research and Development Projects of the Sichuan Provincial Department of Science and Technology (2020YFS0528) to CZ.<br>The funders had no role in study design, data collection and analysis, decision to publish and preparation of the manuscript.

**Funded studies**<br>Enter a statement with the following details:<br>• Initials of the authors who received each award<br>• Grant numbers awarded to each author<br>• The full name of each funder<br>• URL of each funder website<br>• Did the sponsors or funders play any role in the study design, data collection and analysis, decision to publish, or preparation of the manuscript?<br><br>Did you receive funding for this work?"	
Please select the country of your main research funder (please select carefully as in some cases this is used in fee calculation).<br>as follow-up to "**Financial Disclosure**<br><br>Enter a financial disclosure statement that describes the sources of funding for the work included in this submission. Review the submission guidelines for detailed requirements. View published research articles from *PLOS ONE* for specific examples.<br><br>This statement is required for submission and **will appear in the published article** if the submission is accepted. Please make sure it is accurate.<br><br>**Funded studies**<br>Enter a statement with the following details:<br>• Initials of the authors who received each award<br>• Grant numbers awarded to each author<br>• The full name of each funder<br>• URL of each funder website<br>• Did the sponsors or funders play any role in the study design, data collection and analysis, decision to publish, or preparation of the manuscript?<br><br>Did you receive funding for this work?"	CHINA - CN
**Competing Interests**	The authors have declared that no competing interests exist.

Use the instructions below to enter a competing interest statement for this submission. On behalf of all authors, disclose any competing interests that could be perceived to bias this work—acknowledging all financial support and any other relevant financial or non-financial competing interests.<br><br>This statement is required for submission and **will appear in the published article** if the submission is accepted. Please make sure it is accurate and that any funding sources listed in your Funding Information later in the submission form are also declared in your Financial Disclosure statement.<br><br>View published research articles from *PLOS ONE* for specific examples.<br><br>**NO authors have competing interests**<br><br>Enter: *The authors have declared that no competing interests exist.*<br><br>**Authors with competing interests**<br><br>Enter competing interest details beginning with this statement:<br><br>*I have read the journal's policy and the authors of this manuscript have the following competing interests: [insert competing interests here]*<br><br>* typeset	
**Ethics Statement**<br><br>Enter an ethics statement for this submission. This statement is required if the study involved:<br><br>• Human participants<br>• Human specimens or tissue<br>• Vertebrate animals or cephalopods<br>• Vertebrate embryos or tissues	All intervention measures and animal care protocols were conducted in accordance with the Animal Ethics Committee guidelines provided by the North Sichuan Medical College (Sichuan, China). At the end of the experiments, all mice were anesthetized with an intraperitoneal injection of sodium pentobarbital (50 mg/kg body weight) and then euthanized bycervical dislocation.

• Field research<br><br>Write "N/A" if the submission does not require an ethics statement.<br><br>General guidance is provided below. Consult the submission guidelines for detailed instructions. **Make sure that all information entered here is included in the Methods section of the manuscript.**	

Powered by Editorial Manager® and ProduXion Manager® from Aries Systems Corporation

**Format for specific study types**<br><br>**Human Subject Research (involving human participants and/or tissue)**<br>• Give the name of the institutional review board or ethics committee that approved the study<br>• Include the approval number and/or a statement indicating approval of this research<br>• Indicate the form of consent obtained (written/oral) or the reason that consent was not obtained (e.g. the data were analyzed anonymously)<br><br>**Animal Research (involving vertebrate animals, embryos or tissues)**<br>• Provide the name of the Institutional Animal Care and Use Committee (IACUC) or other relevant ethics board that reviewed the study protocol, and indicate whether they approved this research or granted a formal waiver of ethical approval<br>• Include an approval number if one was obtained<br>• If the study involved *non-human primates*, add *additional details* about animal welfare and steps taken to ameliorate suffering<br>• If anesthesia, euthanasia, or any kind of animal sacrifice is part of the study, include briefly which substances and/or methods were applied<br><br>**Field Research**<br><br>Include the following details if this study involves the collection of plant, animal, or other materials from a natural setting:<br>• Field permit number<br>• Name of the institution or relevant body that granted permission	
**Data Availability**<br><br>Authors are required to make all data underlying the findings described fully available, without restriction, and from the time of publication. PLOS allows rare exceptions to address legal and ethical concerns. See the PLOS Data Policy and FAQ for detailed information.	Yes - all data are fully available without restriction

A Data Availability Statement describing where the data can be found is required at submission. Your answers to this question constitute the Data Availability Statement and **will be published in the article**, if accepted.<br><br>**Important:** Stating 'data available on request from the author' is not sufficient. If your data are only available upon request, select 'No' for the first question and explain your exceptional situation in the text box.<br><br>Do the authors confirm that all data underlying the findings described in their manuscript are fully available without restriction?	
**Describe where the data may be found in full sentences. If you are copying our sample text, replace any instances of XXX with the appropriate details.**<br><br>• If the data are **held or will be held in a public repository**, include URLs, accession numbers or DOIs. If this information will only be available after acceptance, indicate this by ticking the box below. For example: *All XXX files are available from the XXX database (accession number(s) XXX, XXX.).*<br>• If the data are all contained **within the manuscript and/or Supporting Information files**, enter the following: *All relevant data are within the manuscript and its Supporting Information files.*<br>• If neither of these applies but you are able to provide **details of access elsewhere**, with or without limitations, please do so. For example:<br><br>*Data cannot be shared publicly because of [XXX]. Data are available from the XXX Institutional Data Access / Ethics Committee (contact via XXX) for researchers who meet the criteria for access to confidential data.*<br><br>*The data underlying the results presented in the study are available from (include the name of the third party*	All relevant data are within the manuscript and its Supporting Information files.

Powered by Editorial Manager® and ProduXion Manager® from Aries Systems Corporation

and contact information or URL).<br>• This text is appropriate if the data are owned by a third party and authors do not have permission to share the data.<br><br>* typeset	
Additional data availability information:	

Manuscript Click here to access/download;Manuscript;Manuscript.docx

Synergistic Effects of Notoginsenoside R1 and Saikosaponin B2 in Atherosclerosis: A Novel Approach Targeting PI3K/AKT/mTOR Pathway and Macrophage Autophagy

Yihua Wang[1,2], Qing Liao[1],Xue Mei[1],Han Xu[1,3], Lijun Luo[1], Rong Huang[1], Yue Tang[1],Chunyang Zhou[1*]

1.Institute of Materia Medica, School of Pharmacy, North Sichuan Medical College, No. 234 Fujiang Road, Nanchong, 637000, Sichuan, China

2.Department of Pharmacy, Nanbu People’s Hospital, Nanbu 637300, Sichuan, China

3.Southwest Medical University Zigong Affiliated Hospital, Zigong Mental Health Center, Zigong, Sichuan , China

*Correspondence: zhouchunyang@nsmc.edu.cn

Abstract

Atherosclerosis (AS) is a significant global contributor to cardiovascular diseases and

this sentence is vague. Make a sharp entence

related mortalities. Although traditional treatments are effective, they often come with side effects. Recent studies have highlighted the potential protective properties of saponin compounds derived from traditional herbal sources, such as notoginsenoside R1 (NGR1) and saikosaponin B2 (SSB2), in combating AS. However, the comprehensive effects and underlying mechanisms of these compounds remain

in which disease?. complete the sentence to make sense the sense strong.

inadequately understood. This study aims to characterize the impact and mechanisms

Methodology is missing in abstract

of the NGR1 and SSB2 combination (NS) on atherosclerosis. Our results demonstrated that the NS combination ameliorates lipid levels, lipid transport, and unstable plaque formation in ApoE$^{-/-}$ mice on a western high-fat diet. Using network pharmacology, we found that the NS combination markedly influences inflammation, apoptosis, and autophagy, implicating crucial proteins such as caspase-3 and mTOR.*In vitro* assays demonstrated that NS effectively inhibits macrophage lipid uptake while downregulating NLRP3 inflammasome-related proteins and genes. Notably, NS also mitigated oxidative stress and apoptosis markers, including Bax, Bcl2, and caspase-3, in macrophages. Both *in vitro* and *in vivo* studies revealed that NS inhibited the PI3K/AKT/mTOR signaling pathway, thereby activating macrophage autophagy. Collectively, these findings suggest that the NS combination enhances protection against atherosclerosis by inhibiting macrophage inflammation and apoptosis, regulating the PI3K/AKT/mTOR signaling pathway, and activating macrophage autophagy. This work paves the way for innovative therapeutic strategies against atherosclerosis, potentially transforming current treatment paradigms.

1. Introduction

Arteriosclerosis(AS) is one of the leading causes of cardiovascular diseases and related deaths globally, including conditions such as myocardial infarction and stroke [1, 2]. With the aging population and the prevalence of unhealthy lifestyles, the incidence of arteriosclerosis has been steadily increasing. Globally, millions of people die each year from arteriosclerosis-related diseases, particularly in developing countries, where the mortality rate is on the rise [3]. Current therapeutic strategies for AS are primarily centered around pharmacological interventions, including lipid-lowering agents, antiplatelet therapies, and invasive procedures like angioplasty or bypass surgery. However, these treatments often come with significant challenges in clinical practice, including issues such as drug resistance, hepatotoxicity, and an increased risk of severe hemorrhagic complications [4-6]. Given these limitations, there is a pressing need for the continuous development of novel therapeutic strategies and drug candidates aimed at minimizing adverse effects while improving clinical outcomes.

Recent studies have shown that phytochemicals derived from traditional herbal plants exhibit a wide range of biological activities, contributing to significant preventive and therapeutic effects against various diseases[7]. Among them, saponins from traditional Chinese herb medicine are particularly notable due to their multifaceted roles in preventing and treating atherosclerotic diseases through mechanisms such as anti-inflammatory, antioxidant, cholesterol-lowering, immunomodulatory, and antiplatelet aggregation effects [8]. Notably, Panax notoginseng and Bupleurum, have demonstrated prominent efficacy in cardiovascular disease management .

Notoginsenoside R1 (NGR1) is a unique component of Panax notoginseng. Previous studies have shown that NGR1 exhibits a variety of biological activities and provides protective effects across multiple organs, including neuroprotection, cardiovascular protection, pulmonary protection, and hepatic protection [9]. Jia and his

team [10]were the first to demonstrate that NR1 may counteract atherosclerosis by modulating inflammation, oxidative stress, and lipid metabolism. More recent studies have further highlighted its role in ameliorating atherosclerosis by alleviating inflammatory responses and inhibiting endothelial dysfunction [11].

Saikosaponin is the active component in Bupleurum, account for up to 7% of the plant's dry root weight and possess a wide array of pharmacological activities. Among them, saikosaponin A (SSA) and saikosaponin D (SSD) have received the most attention in pharmacological research. Of particular interest is saikosaponin B2 (SSB2), a compound formed when SSD is exposed to high temperatures or low pH, reflecting its significance in the traditional preparation of TCM decoctions.[12,13] . Previous studies have indicated that SSB2 exerts anti-inflammatory effects in LPS-induced macrophages, and influence the lipid metabolism process in HepG2 cells, demonstrating anti-atherosclerotic activity potential[14,15]. These findings suggest SSB2 may regulate the occurrence and development of arteriosclerosis, potentially serving as a valuable new drug for treating atherosclerosis. Although studies have suggested that NGR1 and SSB2 may have protective effects in cardiovascular diseases, the precise molecular mechanisms by which these compounds exert their effects on atherosclerosis, either individually or in combination, remain incompletely understood.

Current research attributes the development of atherosclerosis (AS) to lipid accumulation, chronic inflammation, and immune dysregulation[16]. However, the pathogenesis of this disease is not fully understood. A growing body of evidence links AS progression to foam cell formation, which derived from monocyte differentiation and matured macrophages[17,18].When exposed to excessive ox-LDL, macrophages acquired a foam cell-like phenotype through scavenger receptors, leading to necrotic cores formation within atherosclerotic plaques[19]. Persistent exposure to atherogenic lipoproteins, the necrosis and apoptosis of these foam cells result in lipid core necrosis, activating vascular inflammatory responses that lead to cell apoptosis and abnormal lipid metabolism. In contrast, macrophage autophagy has been shown to inhibit the progression of atherosclerosis and stabilize plaques by promoting intracellular lipid

degradation[20-22]. Enhancing autophagic activity in macrophages facilitates intracellular lipid degradation; while inhibition of autophagy predisposes cells to necrosis and inflammation following foam cell death, contributing to plaque formation and rupture[23].Given these insights, inhibiting foam cell formation derived from macrophages and enhancing macrophage autophagy represent effective therapeutic strategies for treating atherosclerosis.

In this context, network pharmacology has become an invaluable tool for systematically evaluating drug-disease interactions, offering significant potential in identifying novel therapeutic targets and pathways. As a tool widely used in traditional Chinese herb medicine research, network pharmacology provides significant insights into the treatment of various diseases. Here, we integrate network pharmacology with *in vitro* cell and *in vivo* experiments to assess whether NGR1 and SSB2 might improve the occurrence and progression of atherosclerosis and to further validate their underlying molecular mechanisms.

In last paragraph make a strong bridging between previous work done and what is left undone.
Make your hypothesis and set the objectives of the study.
All these statements should be loud and clear with strong construction of sentence. revise the paragarph as instructed. This paragraph is not catchy.

2.Materials and methods

2.1 Drugs and reagents

Notoginsenoside R1 (NGR1, chemical structure $C_{47}H_{80}O_{18}$, molecular weight = 933 g/mol, purity = 99.5%) and saikosaponin B2 (SSB2, chemical structure $C_{42}H_{68}O_{13}$, molecular weight = 781 g/mol, purity = 99.5%) were purchased from bidepharm(Shanghai,China). Additionally, the following reagents were purchased:Oxidized low-density lipoprotein (ox-LDL, YB-002, Yiyuan Biotech, China), Rapamycin (HY-10219, MCE, USA), Ultra-sensitive ECL chemiluminescence kit (M2301, HaiGene, China), Reactive Oxygen Species Assay Kit(S0033S, Beyotime, China),Oil Red O (O8010, Solarbio, China), total cholesterol assay kit (A111-1-1, njjcnio, China), triglyceride assay kit (A110-1-1, njjcbio, China), high-density lipoprotein cholesterol assay kit (A112-1-1, njjcbio, China), low-density lipoprotein cholesterol assay kit (A113-1-1, njjcbio, China), creatinine assay kit (C011-2-1,

njjcbio, China), aspartate aminotransferase assay kit (C010-2-1, njjcbio, China), alanine aminotransferase assay kit (C009-2-1, njjcbio, China).

2.2 Animal programs

mention total number of mice?

Male ApoE$^{-/-}$ mice at 7 weeks of age were obtained from the Animal Experimental Center of North Sichuan Medical College (Sichuan, China). All intervention measures and animal care protocols were conducted in accordance with the Animal Ethics Committee guidelines provided by the North Sichuan Medical College (Sichuan, China). The mice were housed in temperature-controlled facilities (temperature: **approval number and date?** 25±2°C, humidity: 55±5%), with a 12-hour light-dark cycle. They were acclimatized for 1 week with ad libitum access to standard maintenance feed and sterile drinking water before experimental procedures commenced.

At 8 weeks of age, 30 ApoE$^{-/-}$ mice were randomly divided into 5 groups (n=6 per group). Due to the poor water solubility of the administered compounds,, a vehicle was prepared with the following composition: 10% DMSO (HY-Y0873, MCE, USA), 40% PEG300 (HY-Y0873, MCE, USA), 5% Tween-80 (HY-Y1891, MCE, USA), and 45% saline solution. The groups included control group (Con,vehicle,ip.), model group (Mod,vehicle,ip.), NGR1 group (NGR1,30 mg/kg/day, ip.), SSB2 group (SSB2,30 mg/kg/day, ip.), and NGR1+SSB2 group (NS,NGR1 30 mg/kg/day + SSB2 30 mg/kg/day, ip.). The dosage of 30 mg/kg/day was selected based on previous pharmacological studies indicating its effectiveness in mitigating atherosclerotic changes while minimizing potential toxicity[24,25]. All groups, except the control group, were fed a Western diet containing 21% fat and 0.15% cholesterol for 12 weeks. Starting from the 9th week, mice received intraperitoneal injections daily for 4 weeks.

2.3 Histological Analysis

Liver tissues were fixed in 10% formalin for 24 hours, then embedded in paraffin or OTC, and consecutively sectioned at a thickness of 7μm. Standard hematoxylin and eosin (H&E) staining was performed on paraffin-embedded tissue sections, while frozen sections of liver embedded in OTC were stained with Oil Red O to assess hepatic lipid accumulation and tissue damage. **must include reference of the protocol.**

The aorta was dissected from the heart base of the mice and fixed in 4% paraformaldehyde. The aortic tissues were embedded in OCT and sectioned at a thickness of 7 μm. Subsequently, sections of the aortic sinus were stained with hematoxylin and eosin (H&E) and Oil Red O (ORO). Additionally, the entire aorta was longitudinally sectioned and stained with Oil Red O to visualize lipid deposition. The areas of lesion in the aorta and aortic sinus were quantified using ImageJ software.

2.4 Biochemical Analysis

Blood was collected from fasting mice via retro-orbital puncture into Eppendorf tubes pre-coated with heparin, and centrifuged at 5000 rpm for 10 minutes at 4°C to isolate plasma. Hepatic and renal indicators, as well as lipid markers, were measured in the plasma samples according to the manufacturer's instructions. These markers included alanine aminotransferase (ALT), aspartate aminotransferase (AST), creatinine (Cr), total cholesterol (TC), triglycerides (TG), low-density lipoprotein cholesterol (LDL-C), and high-density lipoprotein cholesterol (HDL-C).

2.5 Network Pharmacology

Potential targets of NGR1 and SSB2 were identified through SwissTargetPrediction (http://www.swisstargetprediction.ch/), Encyclopedia of Traditional Chinese Medicine (http://www.tcmip.cn/ETCM/index.php/Home/Index/All), Traditional Chinese Medicine Systems Pharmacology Database and Analysis Platform (https://old.tcmsp-e.com/tcmsp.php), and Herb (http://herb.ac.cn/). Molecular targets related to atherosclerosis were screened using GeneCards (https://www.genecards.org/), PharmGKB (https://www.pharmgkb.org/), and OMIM (https://omim.org/) databases. A protein-protein interaction (PPI) network was constructed using STRING 11.0 database (https://string-db.org) and visualized using Cytoscape 3.9.1 to identify key targets based on degree and Maximum Clique Centrality (MCC). Subsequently, key shared targets in the PPI network for NGR1-SSB2-AS were imported into R software for pathways enrichment analysis of Gene Ontology (GO) and Kyoto Encyclopedia of Genes and Genomes (KEGG) using

'enrichplot' and 'clusterProfiler' R packages.

2.6 Cell Culture

RAW264.7 cells (CL-0190) were purchased from Procell(china,wuhan). The cells were cultured in Dulbecco's Modified Eagle Medium (DMEM; KGL1206-500, KeyGEN bioTECH, China), supplemented with 10% fetal bovine serum from South America (FBS,10270106, Gibco, USA)at 37 °C in a humidified atmosphere containing 5% CO_2.

2.7 CCK8 Assay

Cell proliferation was assessed using the CCK-8 kit (KGA9305-500, KeyGEN BioTECH, China). A total of 6 × 10^3 cells per well were seeded in a 96-well plate. The cells were stimulated with varying concentrations of ox-LDL, NGR1, and SSB2, and then incubated under appropriate conditions for 24 hours. Afterward, the culture medium was removed and DMEM containing 10% CCK-8 solution was added to each well. The plates were incubated for an additional 2 hours Absorbance was measured at 450nm using a microplate reader (Bio-Tek, Winooski, VT) after shaking for 30 seconds.

2.8 Oil Red O Staining and Dil Labeling

Oil Red O staining was employed to observe changes in lipid droplet phagocytosis in RAW264.7 cells. A total of 4 × 10^43 cells per well were seeded in a 24-well plate with coverslips. The RAW264.7 cells were treated with ox-LDL, NGR1, and SSB2 for 24 hours. Then, the cells were gently washed with PBS and fixed with 4% paraformaldehyde solution for 3 minutes. They were then stained with freshly prepared Oil Red O working solution in a 37°C water bath for 15 minutes. Followed by counterstain with hematoxylin for 5 minutes. Staining and cell morphology were observed under an optical microscope (OLYMPUS, Japan),where lipid droplets within the cells appear as red granules, and cell nuclei as blue and elliptical.

Similarly, after fixing RAW264.7 cells, DiI labeling was performed by adding the Dil working solution directly to the cells. The cells were stained for 10 minutes and

then observed under an inverted fluorescence microscope. Lipid droplets exhibited red fluorescence, while cell nuclei showed blue fluorescence.

2.9 Analysis of Apoptotic Cells by Flow Cytometry

Cell apoptosis was detected using the Annexin V-FITC/PI double staining apoptosis detection kit (KGA1102-50, KeyGEN BioTECH, China). After 24 hours of treatment, RAW264.7 cells were stained with Annexin V-FITC and propidium iodide (PI) in the dark at room temperature. The extent of apoptosis was then analyzed using a flow cytometer (Agilent, USA).

2.10 Measurement of Cellular Reactive Oxygen Species

RAW264.7 cells were seeded in a 24-well sterile culture plate and treated with the appropriate drug. DCFH-DA (S0033S, Beyotime, China) was diluted with serum-free medium to a final concentration of 10 μmol/L at a ratio of 1:1000. After removing the cell culture medium, the diluted DCFH-DA was added, and the cells were incubated at 37°C for 20 minutes.. The cells were then washed three times with serum-free medium to completely remove any excess DCFH-DA that had not entered the cells. Then, reactive oxygen species were observed using a fluorescence microscope.

2.11 RT-qPCR

Total RNA was extracted from macrophages using Trizol reagent (15596018CN, Invitrogen, USA). The extracted RNA was then reverse transcribed into cDNA using a reverse transcription kit (A502, ExonGen, China). qPCR is performed using Fast SYBR Green qPCR Master Mix UDG (A402, ExonGen, China) on the LightCycler® 480 system (Roche LifeScience, USA). Specific primers for tumor necrosis factor alpha (*Tnf-α),* interleukin-1 Beta *(Il-1β),* interleukin-18 (*Il-18*), and NOD-like receptor family pyrin domain containing 3(*Nlrp3*) were designed using Primer 3 software (Table 1). Expression levels of target gene expression is normalized to β-actin. The relative fold changes in target gene expression were calculated using the $2^{-\Delta\Delta Ct}$ method. Each sample was tested in at least three replicates.

Table 1. Primers used for RT-qPCR

Primer Name	Forward Primer (5'-3')	Reverse primer (5'-3')

β-actin	CTGGCTCCTAGCACCATGAAGA	ACAGTCCGCCTAGAAGCACTTG
TNF-α	CCCTCCAGAAAAGACACCATG	CACCCCGAAGTTCAGTAGACAG
IL-1β	GAAATGCCACCTTTTGACAGTG	TGGATGCTCTCATCAGGACAG
IL-18	GTGAACCCCAGACCAGACTG	CCTGGAACACGTTTCTGAAAGA
Nlrp3	GATCTTCGCTGCGATCAACA	GGGATTCGAAACACGTGCATTA

2.12 Western blot analysis

Total protein was extracted from cells or tissue using RIPA buffer (BL504A, Biosharp, China) supplemented with protease inhibitors (P1045, Beyotime, China), and phosphatase inhibitors (P1045, Beyotime, China). Protein concentration was measured using a BCA protein assay kit (Bio-Rad, China).The protein samples were then mixed with 5× SDS sample buffer (P0015, Beyotime, China) and heated at 95°C for 15 minutes. Subsequently, 30 μg of protein from each sample was separated by 7%, 10%, or 12.5% SDS-PAGE (60V for 15 minutes; 110V for 75 minutes).The proteins were transferredl to a 0.22μm PVDF membrane (Millipore, USA) (200mA for 80 minutes). The membrane was blockedwith 5% non-fat milk in TBST and incubated with diluted primary antibodies at 4°C overnight on a shaker. The antibodies included: AKT Monoclonal antibody (60203-2-Ig, Proteintech, China), Phospho-AKT (Ser473) Monoclonal antibody (66444-1-Ig, Proteintech, China), mTOR Monoclonal antibody (66888-1-Ig), Phospho-mTOR (Ser2448) Monoclonal antibody (67778-1-Ig, Proteintech, China), PI3 Kinase p110 Beta Polyclonal antibody (20584-1-AP, Proteintech, China), Phospho-PI3 Kinase p85 (Tyr458)/p55 (Tyr199) Antibody (4228T, CST, USA), IL-1 Beta Polyclonal antibody (16806-1-AP, Proteintech, China), IL-18 Polyclonal antibody (10663-1-AP, Proteintech, China), Nlrp3 Polyclonal antibody (30109-1-AP, Proteintech, China), Caspase 3/p17/p19 Polyclonal antibody (19677-1-AP, Proteintech, China), BAX Polyclonal antibody (50599-2-Ig), Bcl2 Polyclonal antibody (26593-1-AP, Boster, China), Anti-LC3B/MAP1LC3B Antibody (BM4827, Boster, China), Beclin-1 Antibody (3738T, CST, USA), Anti-P62/SQSTM1 Antibody (BA2849, Boster, China).After incubation with secondary antibodies (Boster, Wuhan, China) for 1 hour, protein bands were visualized using an enhanced ECL chemiluminescence reagent (P0018AM, Beyotime, China) and an Odyssey infrared

imaging scanner (LI-COR Biosciences). The intensity of the bands was analyzed using Image-J software (NIH, Bethesda, MD) and and protein expression levels were normalized to β-actin as a loading control.

2.13 Statistical Analysis

All data were presented as the mean ± standard deviation (SD). Statistical analysis were performed using GraphPad Prism 8.0 (GraphPad Software, Inc.). Comparisons between multiple groups were made using one-way analysis of variance (ANOVA), with p-values < 0.05 considered statistically significant.

3.Results

3.1 Network pharmacology analysis reveals a close association between NS and AS

To explore the potential mechanisms of NS in combating AS, we employed network pharmacology approaches A total of 241 NGR1-related targets, 222 SSB2-related targets, and 4619 AS-related targets were identified, with113 shared targets (Fig 1A). Enrichment analysis using KEGG and GO indicated that NS may modulate processes involved in inflammation, apoptosis, autophagy, and the PI3K/AKT signaling pathway to mediate the progression of atherosclerosis (Fig 1B-C). The protein-protein interaction network comprised 110 nodes and 1732 edges (Fig 1D). Through target contribution (degree) and maximum clique centrality (MCC), 10 key proteins including CASP3 and MTOR were identified as core targets (Fig 1E, 1F).

3.2 NS promotes lipid transport in the ApoE$^{-/-}$ mouse model

The atherosclerosis model was induced in ApoE$^{-/-}$ mice by feeding Western diet for 12 weeks. During this period, the control group showed a declining trend in body weight from weeks 8 to 12, possibly due to stress from intraperitoneal injections (Fig 1A). In contrast, the other groups did not show significant differences in weight change during this time.To investigate the effect of the NS combination on lipid metabolism *in*

vivo, we measured four blood lipid parameters in these ApoE$^{-/-}$ mice fed the Western diet. Compared to the control group, the model group showed significantly elevated levels of TC, TG, and LDL-C, along with a significant reduction in HDL-C. Treatment with NGR1, SSB2, or the NS combinationreduceed the levels of TC, TG, and LDL-C levels,while increaseing HDL-C levels in ApoE$^{-/-}$ mice. Among them, the NS combination group showed the most significant effects (Figs 1B–E). Furthermore, we conducted biochemical and morphological analyses of the liver and kidneys in these mice. The Western diet increased serum ALT and AST levels in the liver. Compared to the model group, the NS combination therapy group showed reduced serum ALT and AST levels (Figs 1F-G). H&E staining of liver sections revealed significant hepatocellular steatosis induced by the Western diet, which was mitigated under NS treatment, as evidenced by reduced lipid vacuoles and inflammatory changes. Oil Red O staining of the liver confirmed a notable decrease in lipid droplets in the NS-treated group (Fig 1I). There were no significant differences observed among groups in kidney morphology (Supplementary Fig 1).

3.3 NS delays the formation of atherosclerotic plaques in the ApoE$^{-/-}$ mouse model

To investigate the effects of NS on atherosclerotic plaques, we assessed the changes in the entire aortic tissues of high-fat-fed ApoE$^{-/-}$ mouse models. Oil Red O staining revealed that the stained area in the NS-treated group was smaller compared to both the high-fat diet group and the monotherapy groups (Fig 3C). Histological examination of aortic root sections under optical microscopy showed severe lipid accumulation, and the formation of unstable plaques with necrotic cores in the model group (Figs 3A-B). In contrast, the plaque area was reduced in the NGR1 and SSB2 treatment groups. Notably, the NS combination intervention significantly limited necrotic area and reduced lipid deposition to stabilize aortic plaques.Macrophage accumulation, a hallmark of atherosclerotic plaques, was assessed using CD68 immunofluorescence analysis. The results indicated that NS treatment significantly

reduced the accumulation of macrophages in atherosclerotic lesions.

3.4 NS combination attenuates ox-LDL-induced foam cell formation

Before assessing the *in vitro* effects of two traditional Chinese medicine monomers, RAW 264.7 macrophage cells were exposed for 24 hours to various concentrations of NGR1 and SSB2, and their cytotoxicity was evaluated using the CCK-8 assay. As shown in Figure 4A, SSB2 did not demonstrate significant cytotoxic effects at concentrations ranging from 12.5 to 50 μM. However, at 100 μM (cell viability 84.09%) and 200 μM (cell viability 61.94%), it exhibited notable cytotoxicity, leading to the selection of 50 μM for further experiments. Meanwhile, NGR1 did not display significant cytotoxicity across concentrations from 12.5 to 200 μM, with a trend of increased cell viability at lower concentrations, peaking at 50 μM (Fig 4B). Therefore, the concentration of 50 μM was chosenas the optimal concentration for NGR1 as well.When over-loaded with ox-LDL, macrophages uptake excessive lipids, inducing the formation of foam cells, and ultimately leading to the instability and rupture of atherosclerotic plaques.To explore the effects of NS on foam cell formation induced by ox-LDL, we established a foam cell model by treating macrophages with 50 μg/ml ox-LDL.We found that after incubation with ox-LDL, RAW 264.7 macrophages transitioned from a round shape to an irregular morphology with pseudopodia, accompanied by significant cytoplasmic lipid droplet accumulation. In contrast, treatment with NGR1 or SSB2 corrected cell morphology and lipid droplet burden. Compared to the individual treatment groups, combination therapy markedly reduced lipid droplet accumulation (Figs 4C-D). These results suggest that NS may inhibit foam cell formation induced by ox-LDL in RAW 264.7 macrophages.

3.5 NS inhibits inflammation induced by Ox-LDL in RAW264.7 cells

Based on *in vivo* experiments and network pharmacology results, we further investigated whether NS could inhibit ox-LDL-induced macrophage inflammatory responses. Results from Figure 5(A-D) revealed that NR1 and SSB2 significantly reduced the gene expression levels of inflammatory cytokines (TNF-α, IL-1β, IL-18) as well as NLRP3. Visual and quantitative results from Western blotting indicated that ox-LDL stimulation markedly increased the expression of NLRP3 inflammasome-related proteins, including NLRP3, IL-1β, and IL-18.Treatment with NGR1 or SSB2 reversed this upregulation, with the combination therapy showing a more pronounced inhibitory effect (Figs 5E-H).

3.6 NS inhibits apoptosis induced by Ox-LDL in RAW264.7 cells

Network pharmacology results indicate that the anti-atherosclerotic effects of NS are closely associated with apoptosis, with CASP3 playing a crucial role in apoptosis and identified as a potential key target of NS in its anti-atherosclerotic action. To understand the role of NS in ox-LDL-induced apoptosis in RAW264.7 cells, we measured intracellular ROS levels and assessed apoptosis using flow cytometry and western blotting .Compared to the model group, the NS combination treatment significantly reduced the ROS levels in RAW264.7 cells (Fig 6A). As shown in Figure 6B, compared to the control group, ox-LDL-treated RAW264.7 cells exhibited a significantly increased number of apoptotic cells, this increase was reversed by NS treatment. Consistent with the changes in apoptosis data, ox-LDL induction in the model group led to the downregulation of the anti-apoptotic protein Bcl-2, while upregulating the pro-apoptotic proteins Bax and cleaved caspase-3. Treatment with either NGR1 or SSB2 attenuated the apoptotic effects of ox-LDL, and compared to single-drug treatment, the combination therapy further inhibited apoptosis in macrophage cells (Figs 6C-F). In summary, these data indicate that NS effectively suppresses ox-LDL-induced apoptosis in RAW264.7 cells.

3.7 NS affects the PI3K/AKT/mTOR pathway in RAW264.7 cells

The above results indicate that the PI3K/AKT signaling pathway plays a significant role in the anti-atherosclerotic effect of NS. mTOR is a potential core target for the anti-atherosclerotic effect of NS, acting as a downstream target of the PI3K/AKT pathway and exerting pleiotropic cellular effects.Therefore, we further explored the changes in the PI3K/AKT/mTOR pathway *in vivo* and in ox-LDL-induced macrophages treated with NS. We observed the expression of proteins involved in the PI3K/AKT/mTOR signaling pathway. In ApoE$^{-/-}$ mice, as shown by western blot results, the ratio of p-PI3K/PI3K in ox-LDL-exposed RAW264.7 cells was significantly higher than in the control group. NS treatment markedly attenuated this increase. Similarly, the ratio of p-Akt/Akt and p-mTOR/mTOR in ox-LDL-exposed RAW264.7 cells was significantly enhanced compared to the control group. In contrast, NS treatment significantly reduced these ratios (Figs 7A-D). Immunofluorescence analysis further supported these findings. Compared to the control group, the staining intensity of P-mTOR gradually increased in the ox-LDL group. Additionally, P-mTOR expression was reduced in the NS treatment group (Figs 7E-F). These results indicates that NS partially inhibits the activation of the PI3K/Akt/mTOR pathway.

3.8 NS inhibits AS by enhancing macrophage autophagic flux

Autophagy is deactivated in foam cells, and reactivating the autophagic process can reverse foam cell formation, thereby improving atherosclerotic plaques. Within the autophagy regulatory pathway, mTOR acts as a negative regulator of autophagy. Thus, inhibiting the PI3K/AKT/mTOR pathway can regulate macrophage autophagy and significantly impact the progression of atherosclerosis.Using Western blotting, we evaluated the expression of key autophagy proteins Beclin-1, P62, and LC3 in

ox-LDL-exposed RAW264.7 cells.Our findings indicated that NS treatment significantly enhances Beclin-1 expression in ox-LDL-exposed RAW264.7 cells. Conversely, NS treatment markedly attenuates P62 expression in ox-LDL-exposed RAW264.7 cells. Additionally, the LC3 II/I protein ratio, which was significantly reduced in ox-LDL-exposed RAW264.7 cells, was significantly reversed by NS treatment. Immunofluorescence analysis of P62 to assess autophagic activity in lesions revealed decreased autophagic activity in atherosclerotic lesions. However, following administration of NS, autophagy significantly recovered (Fig 8E-F). In summary, these results indicate that NS can alleviate atherosclerosis by activating macrophage autophagy.

3.9 NS restores autophagy via the PI3K/AKT/mTOR pathway, inhibiting foam cell formation and suppressing inflammation and apoptosis in ox-LDL-stimulated macrophages

To futher determine whether the NS improves atherosclerosis by affecting the PI3K/Akt/mTOR signaling pathway and autophagy, we evaluated the effects of rapamycin (Ra, an mTOR inhibitor) and 3-MA (an autophagy inhibitor) on ox-LDL-stimulated macrophages. We observed that rapamycin further reduced the accumulation of lipid droplets and apoptosis rate induced by ox-LDL. In contrast, the beneficial effects of NS treatment were partially blocked by 3-MA (Figs 9A-B). Next, we measured the levels of key proteins using Western blot, including NLRP3 (Fig 9D), caspase-3 (Fig 9G),the P-mTOR/mTOR ratio (Fig 9H), and p62 (Fig 9I). Compared to the control group, the Mod group showed a significant increase in these markers. In contrast, BCL2 (Fig 9F) and Beclin1 (Fig 9J) were significantly reduced in the ox-LDL group, indicating that ox-LDL treatment activates the PI3K/Akt/mTOR pathway and apoptosis while inhibiting autophagic flux and inflammation in RAW264.7 cells. As expected, compared to cells treated with ox-LDL alone, NS treatment significantly reduced the expression of NLRP3, p-mTOR, BAX, and p62. The NS treatment group showed significantly higher levels of BCL2, Beclin1, and LC3-II compared to the Mod group, with rapamycin further enhancing the effects of NS. However, these effects of

NS could be partially eliminated by the autophagy inhibitor 3-MA. These results suggested that NS mitigates the activation of the PI3K/Akt/mTOR signaling pathway induced by ox-LDL, thereby restoring autophagic flux in ox-LDL-treated macrophages and inhibiting foam cell formation and apoptosis.

First paragraph of the discussion should restate objectives of the study with novel findings.

4.Discussion

Highlighted part is good for introduction or can be put as logic after discussion of your data.

Atherosclerosis is an arterial vascular disease characterized by the accumulation of lipid substances within the blood vessel wall, forming plaques that can ultimately lead to vascular narrowing or occlusion. NGR1 and SSB2 are active compounds derived from *Panax notoginseng* and *Bupleurum*, respectively. In previous studies, both compounds have shown an anti-atherosclerotic tendency [15, 24]; however, the

Mention your result then argue it with reference or your logic

specific mechanism of their combined therapy in treating AS remains unclear. In this study, we employed the well-established ApoE$^{-/-}$ mouse model as an *in vivo* model of atherosclerosis. The results showed that serum levels of TC, TG, and LDL-C were significantly elevated in ApoE$^{-/-}$ mice fed a high-fat diet compared to the control group, while HDL-C levels were significantly lower than in the control group. Additionally, histopathological staining revealed prominent plaques in the aortic sinuses of the model group. On the other hand, NS combination treatment reduced serum levels of TC, TG, and LDL-C in ApoE$^{-/-}$ mice, increased HDL-C and lipid transport levels, and decreased the formation of AS plaques and hepatic lipid vacuoles. *In vitro*, ox-LDL induced substantial lipid engulfment by RAW 264.7 macrophages was significantly reduced by NS combination therapy compared to either individual drug treatment, suggesting that NS markedly improves lipid metabolism in AS.

Always start new variable of your result in new paragraph by citing your figure or table and then support it with references or previously work done.

Atherosclerosis is a complex chronic vascular disease involving intertwined and cooperative pathological processes and factors, primarily including inflammation, oxidative stress, apoptosis, lipid accumulation, and autophagy [16]. Macrophages play a crucial role in the development of atherosclerosis [25], influencing both plaque formation and stability, and therefore the risk of potential complications. Therefore,

Again always start with yur result by citing your data. Highlighted paragraph is not a good start in the discussion. Always start with your result.

investigating the various mechanisms by which macrophages act in atherosclerosis is crucial for elucidating their key role in disease progression. Inflammatory biomarkers are independent risk factors for cardiovascular events [27]. Uptake of ox-LDL induces macrophage transformation into foam cells, which are rich in lipids and cholesterol. These cells also release inflammatory mediators such as chemokines and cell adhesion molecules, promoting the development of inflammatory responses [26].The NLRP3 inflammasome is a multiprotein complex located in the cytoplasm that is responsible for the maturation of pro-inflammatory cytokines such as interleukin-1β (IL-1β) and IL-18 [27]. Our study showed that NS significantly inhibited ox-LDL-induced inflammation by downregulating pro-inflammatory factors, including TNF-α, IL-1β, and IL-18, indicating that NS treatment can suppress macrophage-driven inflammatory responses in AS. **Cite your data? like as shown in Figure….**

The formation and apoptosis of macrophage foam cells are considered critical processes in the development of atherosclerosis [30]. Liao and her team suggested that macrophage apoptosis can destabilize the interior of plaques, leading to inflammation and necrosis, increasing the risk of plaque rupture and thrombus formation, causing acute cardiovascular events such as myocardial infarction or stroke [31].Therefore, **This is theory part for introduction. You can support it for your result but you have to show your result first then give reason.** protecting macrophages from apoptosis is considered an effective strategy to mitigate plaque instability and counteract acute vascular events. Proteins such as Caspase-3, Bax, and Bcl2 are critical markers associated with apoptosis, and their expression directly reflects the extent of cellular apoptosis [32]. Additionally, oxidative stress can accelerate macrophage apoptosis, leading to an increased risk of acute vascular events[33]. In this study, we observed that NS significantly reduces the apoptosis rate and oxidative stress induced by ox-LDL . This effect is evidenced by the downregulation of the pro-apoptotic protein Bax and the upregulation of the anti-apoptotic protein Bcl-2 following NS treatment. **Cite your data here?**

Autophagy is a cellular degradation process that breaks down and recycles damaged organelles and molecules [34]. In recent years, studies have highlighted the beneficial role of autophagy in AS[35-37]. To further explore the mechanism of NS in

atherosclerosis, we established an ox-LDL-induced macrophage model *in vitro* and evaluated the effects of NS on macrophage autophagy.The upregulation of the autophagy key protein Beclin-1 serves as an indicator of autophagosome activation [38], and together with LC3-II, it constitutes critical components involved in the formation of autophagosomes in mammals [39].The autophagy-related protein light chain 3 (LC3) is a key biomarker for autophagy. During autophagy activation, LC3-II is abundantly produced, making it an important marker for detecting autophagy initiation. P62 serves as an adaptor protein critical for recognizing and clearing cellular cargo [40]. Our study demonstrated that NS treatment significantly upregulated the expression of autophagy markers LC3-II and Beclin-1, while reducing p62 levels, indicating restored autophagic flux in ox-LDL-stimulated macrophages. This enhancement of autophagy suggests that NS treatment prevents foam cell formation and apoptosis, contributing to improved plaque stability.

This is theory part for introduction. You can support it for your result but you have to show your result first then give reason.

There is a complex interplay between autophagy, inflammation, and apoptosis. Studies have shown that inducing autophagy in macrophages can effectively reduce foam cell formation and accumulation in atherosclerotic plaques, and promote plaque stability by protecting cells from oxidative stress, reducing apoptosis, and decreasing the secretion of inflammatory factors [39, 40]. Furthermore, autophagy can also act as an apoptosis inhibitor by reducing the activation and formation of inflammasomes [41]. In the current study, we first evaluated the effects of NS on foam cell formation and apoptosis, intracellular ox-LDL accumulation, and the activity of inflammatory factors. Our data support the hypothesis that NS promotes autophagy, which in turn reduces foam cell formation, inhibits inflammation, and prevents apoptosis. Notably, the addition of an autophagy inhibitor (3-MA) abrogated the protective effects of NS, highlighting the central role of autophagy in mediating the therapeutic effects of the NS combination. **Cite your data here.**

The PI3K/Akt/mTOR signaling pathway is associated with various cellular functions, including inflammation, apoptosis, and autophagy, thereby influencing cell survival and death [42, 43]. Selective inhibition of the PI3K/Akt/mTOR signaling

pathway has been demonstrated to regulate macrophage autophagy and plays a crucial role in the formation and progression of atherosclerotic plaques [39]. This study indicates that ox-LDL overload activates the PI3K/Akt/mTOR signaling pathway through enhanced phosphorylation of PI3K, AKT, and mTOR, leading to the accumulation of the autophagy marker P62 and a significant reduction in LC3B and Beclin1. This is consistent with previous research showing that ox-LDL inhibits

Reference is missing here?

autophagy in macrophages by activating the PI3K/Akt/mTOR pathway[]. In contrast, NS blocks the phosphorylation of PI3K, Akt, and mTOR, thereby restoring the activity of LC3B and Beclin1.Furthermore, adding the mTOR inhibitor rapamycin to NS treatment showed an enhanced positive effect.

In summary, our study provides novel insights into the combined effects of NGR1 and SSB2 on atherosclerosis. By modulating the PI3K/Akt/mTOR signaling pathway, NS treatment enhances autophagy, inhibits inflammation, and reduces macrophage apoptosis, offering a promising therapeutic strategy for combating atherosclerosis. These findings underscore the potential of the NS combination as an effective treatment for atherosclerosis, particularly through its capacity to restore autophagic balance and improve plaque stability (Fig 10).

Dont you think, it is already in conclusion part. This information is useless in highlighted part. Same thing is mentioned in conclusion

5.Conclusion

This pioneering study provides compelling evidence that the combination of NGR1 and SSB2 (NS) effectively mitigates inflammation and inhibits the activation of the PI3K/Akt/mTOR signaling pathway in atherosclerosis. Our findings reveal that NS treatment promotes autophagy, reduces the formation of inflammatory foam cells, and decreases macrophage apoptosis. This multifaceted approach underscores NS's potential as a therapeutic strategy for preventing the onset and progression of atherosclerosis. Importantly, our research elucidates the molecular mechanisms underlying the synergistic effects of NGR1 and SSB2, highlighting their therapeutic promise in atherosclerosis management. By restoring autophagic flux and modulating inflammatory responses, NS not only addresses key pathological features of AS but

also opens avenues for innovative treatment strategies targeting lipid metabolism and macrophage function.

In conclusion, this study lays a solid foundation for future investigations into the clinical applications of NS in atherosclerosis and emphasizes the need for further research to explore its potential in combination therapies. The insights gained from this research contribute significantly to our understanding of atherosclerosis pathophysiology and may inform the development of novel interventions aimed at improving cardiovascular health.

Acknowledgment:

Funding from the Scientific Startup Foundation for Doctors of North Sichuan Medical College to Xue Mei (CBY23-QDA11) and Key Research and Development Projects of the Sichuan Provincial Department of Science and Technology (2020YFS0528).

Author Contributions

Conceptualization: Chunyang Zhou

Data curation: Yihua Wang,Qing Liao.

Formal analysis: Yihua Wang,Xue Mei.

Funding acquisition: Chunyang Zhou，Xue Mei.

Investigation: Chunyang Zhou.

Methodology: Yihua Wang,Qing Liao,Han Xu.

Project administration: Chunyang Zhou.

Software: Yihua Wang.

Validation: Yihua Wang.

Visualization: Rong Huang, Yue Tang.

Writing – original draft: Yihua Wang.

Writing – review & editing: Chunyang Zhou,Xue Mei,Lijun Luo.

References:

1. Virani, S.S., et al., Heart Disease and Stroke Statistics-2021 Update: A Report From the American Heart Association. Circulation, 2021. 143(8): p. e254-e743.

2. Frostegård, J., Immunity, atherosclerosis and cardiovascular disease. BMC medicine, 2013. 11: p. 117.

3. Global Health Estimates 2020: Deaths by Cause, Age, Sex, by Country and by Region, 2000 – 2019. Geneva, World Health Organization, 2020.

4. Bułdak, Ł., Cardiovascular Diseases-A Focus on Atherosclerosis, Its Prophylaxis, Complications and Recent Advancements in Therapies. International journal of molecular sciences, 2022. 23(9).

5. Libby, P., The changing landscape of atherosclerosis. Nature, 2021. 592(7855): p. 524-533.

6. Xu, S., J. Pelisek and Z.G. Jin, Atherosclerosis Is an Epigenetic Disease. Trends in endocrinology and metabolism: TEM, 2018. 29(11): p. 739-742.

7. Soltani, S., et al., Effects of phytochemicals on macrophage cholesterol efflux capacity: Impact on atherosclerosis. Phytotherapy research : PTR, 2021. 35(6): p. 2854-2878.

[8. Luo, H., et al., Advances in the Bioactivities of Phytochemical Saponins in the Prevention and Treatment of Atherosclerosis. Nutrients, 2022. 14(23).

9. Liu, H., et al., Focus on Notoginsenoside R1 in Metabolism and Prevention Against Human Diseases. Drug design, development and therapy, 2020. 14: p. 551-565.

10. Jia, C., et al., Notoginsenoside R1 attenuates atherosclerotic lesions in ApoE deficient mouse model. PloS one, 2014. 9(6): p. e99849.

11. Ma, L., et al., Notoginsenoside R1 Ameliorate High-Fat-Diet and Vitamin D3-Induced Atherosclerosis via Alleviating Inflammatory Response, Inhibiting Endothelial Dysfunction, and Regulating Gut Microbiota. Drug design, development and therapy, 2024. 18: p. 1821-1832.

12. Ochi, A., et al., Highly sensitive indirect competitive enzyme-linked immunosorbent assay based on a monoclonal antibody against saikosaponin b2 for quality control of Kampo medicines containing Bupleuri radix. Journal of natural medicines, 2024. 78(1): p. 160-168.

13. Shimizu, K., S. Amagaya and Y. Ogihara, Structural transformation of saikosaponins by gastric juice and intestinal flora. Journal of pharmacobio-dynamics, 1985. 8(9): p. 718-25.

14. Shin, J., H. Im and K. Lee, Saikosaponin B2 Suppresses Inflammatory Responses Through IKK/IκBα/NF-κB Signaling Inactivation in LPS-Induced RAW 264.7 Macrophages. Inflammation, 2019. 42(1): p. 342-353.

15. Liu, S., et al., HepG2 cells biospecific extraction and HPLC-ESI-MS analysis for screening potential antiatherosclerotic active components in Bupeuri radix. Journal of pharmaceutical and biomedical analysis, 2016. 121: p. 56-62.

16. Libby, P., et al., Atherosclerosis. Nature reviews. Disease primers, 2019. 5(1): p. 56.

17. Gui, Y., H. Zheng and R.Y. Cao, Foam Cells in Atherosclerosis: Novel Insights Into Its Origins, Consequences, and Molecular Mechanisms. Frontiers in cardiovascular medicine, 2022. 9: p. 845942.

18. Wan, Q., et al., Atmospheric fine particulate matter exposure exacerbates atherosclerosis in apolipoprotein E knockout mice by inhibiting autophagy in macrophages via the PI3K/Akt/mTOR signaling pathway. Ecotoxicology and environmental safety, 2021. 208: p. 111440.

19. Liu, Y., et al., Retinol-Binding Protein-Dependent Cholesterol Uptake Regulates Macrophage Foam Cell Formation and Promotes Atherosclerosis. Circulation, 2017. 135(14): p. 1339-1354.

20. Pi, S., et al., The P2RY12 receptor promotes VSMC-derived foam cell formation by inhibiting

autophagy in advanced atherosclerosis. Autophagy, 2021. 17(4): p. 980-1000.
21. Ouimet, M., et al., microRNA-33 Regulates Macrophage Autophagy in Atherosclerosis. Arteriosclerosis, thrombosis, and vascular biology, 2017. 37(6): p. 1058-1067.
22. Ouimet, M., et al., Autophagy regulates cholesterol efflux from macrophage foam cells via lysosomal acid lipase. Cell metabolism, 2011. 13(6): p. 655-67.
23. Liao, X., et al., Macrophage autophagy plays a protective role in advanced atherosclerosis. Cell metabolism, 2012. 15(4): p. 545-53.
24. Hou, D., et al., Notoginsenoside R1 improves intestinal microvascular functioning in sepsis by targeting Drp1-mediated mitochondrial quality imbalance. Pharmaceutical biology, 2024. 62(1): p. 250-260.
25. Ma, Q., et al., Antitumor effects of saikosaponin b2 on breast cancer cell proliferation and migration. Molecular medicine reports, 2019. 20(2): p. 1943-1951.
26. Colin, S., G. Chinetti-Gbaguidi and B. Staels, Macrophage phenotypes in atherosclerosis. Immunological reviews, 2014. 262(1): p. 153-66.
27. Libby, P., Inflammation in atherosclerosis. Arteriosclerosis, thrombosis, and vascular biology, 2012. 32(9): p. 2045-51.
28. Hansson, G.K., A.L. Robertson and C. Söderberg-Nauclér, Inflammation and atherosclerosis. Annual review of pathology, 2006. 1: p. 297-329.
29. Batty, M., M.R. Bennett and E. Yu, The Role of Oxidative Stress in Atherosclerosis. Cells, 2022. 11(23).
30. Ghattas, A., et al., Monocytes in coronary artery disease and atherosclerosis: where are we now? Journal of the American College of Cardiology, 2013. 62(17): p. 1541-51.
31. Tabas, I., Macrophage apoptosis in atherosclerosis: consequences on plaque progression and the role of endoplasmic reticulum stress. Antioxidants & redox signaling, 2009. 11(9): p. 2333-9.
32. Xue, Q., et al., Copper metabolism in cell death and autophagy. Autophagy, 2023. 19(8): p. 2175-2195.
33. Papaharalambus, C.A. and K.K. Griendling, Basic mechanisms of oxidative stress and reactive oxygen species in cardiovascular injury. Trends in cardiovascular medicine, 2007. 17(2): p. 48-54.
34. Mizushima, N. and M. Komatsu, Autophagy: renovation of cells and tissues. Cell, 2011. 147(4): p. 728-41.
35. Cao, H., et al., Quercetin Suppresses the Progression of Atherosclerosis by Regulating MST1-Mediated Autophagy in ox-LDL-Induced RAW264.7 Macrophage Foam Cells. International journal of molecular sciences, 2019. 20(23).
36. Zhou, M., et al., Shen-Yuan-Dan Capsule Attenuates Atherosclerosis and Foam Cell Formation by Enhancing Autophagy and Inhibiting the PI3K/Akt/mTORC1 Signaling Pathway. Frontiers in pharmacology, 2019. 10: p. 603.
37. Han, X.B., et al., Upconversion nanoparticle-mediated photodynamic therapy induces autophagy and cholesterol efflux of macrophage-derived foam cells via ROS generation. Cell death & disease, 2017. 8(6): p. e2864.
38. Ouimet, M., Autophagy in obesity and atherosclerosis: Interrelationships between cholesterol homeostasis, lipoprotein metabolism and autophagy in macrophages and other systems. Biochimica et biophysica acta, 2013. 1831(6): p. 1124-33.
39. Tanida, I., T. Ueno and E. Kominami, LC3 conjugation system in mammalian autophagy. The international journal of biochemistry & cell biology, 2004. 36(12): p. 2503-18.

40. Valionyte, E., et al., The caspase-6-p62 axis modulates p62 droplets based autophagy in a dominant-negative manner. Cell death and differentiation, 2022. 29(6): p. 1211-1227.
41. Zhai, C., et al., Selective inhibition of PI3K/Akt/mTOR signaling pathway regulates autophagy of macrophage and vulnerability of atherosclerotic plaque. PloS one, 2014. 9(3): p. e90563.
42. Kiffin, R., U. Bandyopadhyay and A.M. Cuervo, Oxidative stress and autophagy. Antioxidants & redox signaling, 2006. 8(1-2): p. 152-62.
43. Li, R., et al., Interference in autophagosome fusion by rare earth nanoparticles disrupts autophagic flux and regulation of an interleukin-1β producing inflammasome. ACS nano, 2014. 8(10): p. 10280-92.
44. Saiki, S., et al., Caffeine induces apoptosis by enhancement of autophagy via PI3K/Akt/mTOR/p70S6K inhibition. Autophagy, 2011. 7(2): p. 176-87.
45. Zheng, X., et al., Sinomenine ester derivative inhibits glioblastoma by inducing mitochondria-dependent apoptosis and autophagy by PI3K/AKT/mTOR and AMPK/mTOR pathway. Acta pharmaceutica Sinica. B, 2021. 11(11): p. 3465-3480.

Figure Legends

Figure 1. Network pharmacology analysis predicted the targets of NS on AS.(A) Venn analysis of NGR1, SSB2, and AS overlapping targets.(B) GO enrichment analysis of NS-AS shared targets.(C) KEGG enrichment analysis of NS-AS shared targets.(D) PPI (Protein-Protein Interaction) network of NS-AS shared targets.(E) Top 10 core targets selected from a list of 113 targets based on degree contribution.(F) Identification of 10 targets from shared targets based on Maximum Clique Centrality (MCC).

Figure 2. NS affects lipid transport in $ApoE^{-/-}$mouse models.

Eight-week-old male $ApoE^{-/-}$ mice, weighing approximately 25g, were fed a Western high-fat diet for 12 weeks. They were then intraperitoneally injected with NGR1 (30 mg/kg/day) and SSB2 (30 mg/kg/day) for 4 weeks. (A) Effects of NGR1 and SSB2 on the body weight of $ApoE^{-/-}$ mice. (B) Triglycerides (TG), (C) total cholesterol (TC), (D) high-density lipoprotein cholesterol (HDL-C), (E) low-density lipoprotein cholesterol (LDL-C), (F) alanine transaminase, (G) aspartate transaminase, and (H) creatinine levels in the control group(Con), model group(Mod), NGR1 group(NGR1), SSB2 group(SSB2), and NGR1+SSB2 combination treatment group(NS). (I) Representative images of liver sections stained with H&E and Oil Red O. Tissues were examined using an optical microscope (scale bar = 50 μm).All data were presented as the mean ± SD. *p < 0.05 vs. control group, **p < 0.01 vs. control group, #p < 0.05 vs. model group, ##p < 0.01 vs. model group, $$$p$ < 0.01 vs. NGR1 group,+p < 0.05 vs. SSB2 group, ++p < 0.01 vs. SSB2 group.

Figure 3. NS delays the formation of atherosclerotic plaques in $ApoE^{-/-}$ mouse models.

(A, D) Representative images and quantitative analysis of the aortic roots from $ApoE^{-/-}$ mice using H&E staining (scale bars = 200 μm and 100 μm, respectively). (B, E) Representative images and quantitative analysis of the aortic roots from $ApoE^{-/-}$ mice using Oil Red O staining (scale bar = 200 μm). (C, F) Representative images and quantitative analysis of CD68 immunostaining in the aortic root (scale bar = 50 μm).All data were presented as the mean ± SD,(n=3). *p < 0.05 vs. control group, **p < 0.01 vs. control group, #p < 0.05 vs. model group, ##p < 0.01 vs. model group, $$$p$ < 0.01 vs. NGR1 group,+p < 0.05 vs. SSB2 group, ++p < 0.01 vs. SSB2 group.

Figure 4.NS attenuates ox-LDL-induced foam cell formation.(A) Effect of NGR1 on RAW264.7 cell viability.(B) Effect of SSB2 on RAW264.7 cell viability.(C) Representative images of Oil Red O staining showing lipid droplets accumulation in RAW264.7 cells. Cells were examined using an optical microscope (scale bar = 100 μm).(D) Representative fluorescence images of RAW264.7 cells labeled with Dil-oxLDL. Cells were visualized using inverted fluorescence microscopy (scale bar = 50 μm).All data were presented as the mean ± SD,(n=3). *$p < 0.05$ vs. control group, **$p < 0.01$ vs. control group.

Figure 5. NS suppresses inflammation induced by Ox-LDL in RAW264.7 cells. Relative mRNA levels of inflammatory genes (A) *TNF-α*,(B) *IL-1β*, (C) *IL-18*, and (D) NLRP3 measured by RT-qPCR. (E)Representative Western blot images showing protein expression of NLRP3, IL-1β, and IL-18 in the Ox-LDL-induced cell model. β-actin was used as a loading control. (F-H) Quantification of the relative protein levels of NLRP3, IL-1β, and IL-18, normalized to β-actin and presented as relative intensity based on Western blot.analysis. All data were presented as the mean ± SD,(n=3). *p < 0.05 vs. control group, **p < 0.01 vs. control group, #p < 0.05 vs. model group, ##p < 0.01 vs. model group,$p < 0.05 vs. NGR1 group, $$p < 0.01 vs. model group,$p < 0.05 vs. NGR1 group, $$p < 0.01 vs. NGR1 group,+p < 0.05 vs. SSB2 group, ++p < 0.01 vs. SSB2 group.

Figure 6. NS co-suppresses Ox-LDL-induced apoptosis in RAW264.7 cells.(A) ox-LDL induces ROS generation in RAW264.7 cells, visualized by inverted fluorescence microscopy (scale bar = 50 μm).(B) Representative images of apoptosis detected by flow cytometry.(C) Representative Western blot images showing protein expression of BAX, BCL2, and Caspase-3 in the ox-LDL-induced cell model. β-actin was used as a loading control.(D-F) Quantification of the relative protein levels of BAX, BCL2, and Caspase-3 normalized to β-actin, and presented as relative intensity measured by Western blot analysis. All data were presented as the mean ± SD,(n = 3). *p < 0.05 vs. control group, **p < 0.01 vs. control group, #p < 0.05 vs. model group, ##p < 0.01 vs. model group,$p < 0.05 vs. NGR1 group, $$p < 0.01 vs. model group,$p < 0.05 vs. NGR1 group, $$p < 0.01 vs. NGR1 group,+p < 0.05 vs. SSB2 group, ++p < 0.01 vs. SSB2 group.

Figure 7. NS affects the PI3K/AKT/mTOR pathway in RAW264.7 cells.(A) Representative western blot images showing protein expression of P-PI3K, PI3K, P-AKT, AKT, P-mTOR, and mTOR in the Ox-LDL-induced cell model. β-actin was used as a loading control.(B) Quantitative analysis of P-PI3K/PI3K ratio.(C) Quantitative analysis of P-AKT/AKT ratio.(D) Quantitative analysis of P-mTOR/mTOR ratio. The ratios are presented as relative intensities.(E) Representative fluorescence staining image of P-mTOR examined by inverted fluorescence microscopy (scale bar = 50 μm).(F)Quantitative analysis of representative fluorescent staining images of P-AKT, expressed as relative intensity.All data were presented as the mean ± SD,(n = 3). *p < 0.05 vs. control group, **p < 0.01 vs. control group, #p < 0.05 vs. model group, ##p < 0.01 vs. model group,$p < 0.05 vs. NGR1 group, $$p < 0.01 vs. model group,$p < 0.05 vs. NGR1 group, $$p < 0.01 vs. NGR1 group,+p < 0.05 vs. SSB2 group, ++p < 0.01 vs. SSB2 group.

Figure 8. NS enhances autophagy inhibits atherosclerosis AS in RAW264.7 cells.
(A)Representative Western blot images showing protein expression levels of BECLIN1, LC3, and P62 in the Ox-LDL-induced cell model. β-actin was used as a loading control.(B-D) Relative protein levels of BECLIN1, LC3, and P62, normalized to β-actin and presented as relative intensities.(E) Representative fluorescence staining images of P62 examined by inverted fluorescence microscopy (scale bar = 50 μm).(F) Quantitative analysis of the fluorescence intensity of P62 from representative staining images. All data were presented as the mean ± SD,(n = 3). *p < 0.05 vs. control group, **p < 0.01 vs. control group, #p < 0.05 vs. model group, ##p < 0.01 vs. model group,$p < 0.05 vs. NGR1 group, $$p < 0.01 vs. model group,$p < 0.05 vs. NGR1 group, $$p < 0.01 vs. NGR1 group,+p < 0.05 vs. SSB2 group, ++p < 0.01 vs. SSB2 group.

Figure 9. NS restores autophagy through the PI3K/Akt/mTOR pathway, reducing foam cell formation and suppressing inflammation and apoptosis in ox-LDL-stimulated macrophages.
(A)Representative images of Oil Red O staining showing lipid droplets in RAW264.7 cells. Cells were examined under a light microscope (scale bar = 100 μm).(B) Representative images of cell apoptosis detected by flow cytometry.(C) Representative Western blot images showing protein expression levels of P-mTOR, mTOR, NLRP3, BAX, BCL2, Caspase3, P62, BECLIN1, and LC3

in ox-LDL-induced cellular models. β-actin was used as the loading control.(D-K) Quantification of relative protein levels were normalized to β-actin and were presented as relative intensity.All data were presented as the mean ± SD,(n = 3). *p < 0.05 vs. control group, **p < 0.01 vs. control group, #p < 0.05 vs. model group, ##p < 0.01 vs. model group,$p < 0.05 vs. NS group, $$p < 0.01 vs. NS group,+p < 0.05 vs. NS group, ++p < 0.01 vs. NS group. Rapamycin: 0.5 μM, 3-MA: 3 mM.

Figure 10. NS effectively modulates the PI3K/Akt/mTOR signaling pathway and inhibits lipid transport, inflammation, and apoptosis in macrophages by enhancing autophagy.

Supplementary materials:

S1 Figure.The HE-stained section of mouse kidney, scale bar = 50 μm.

Figure 1

Click here to access/download;Figure;Figure 1.tif

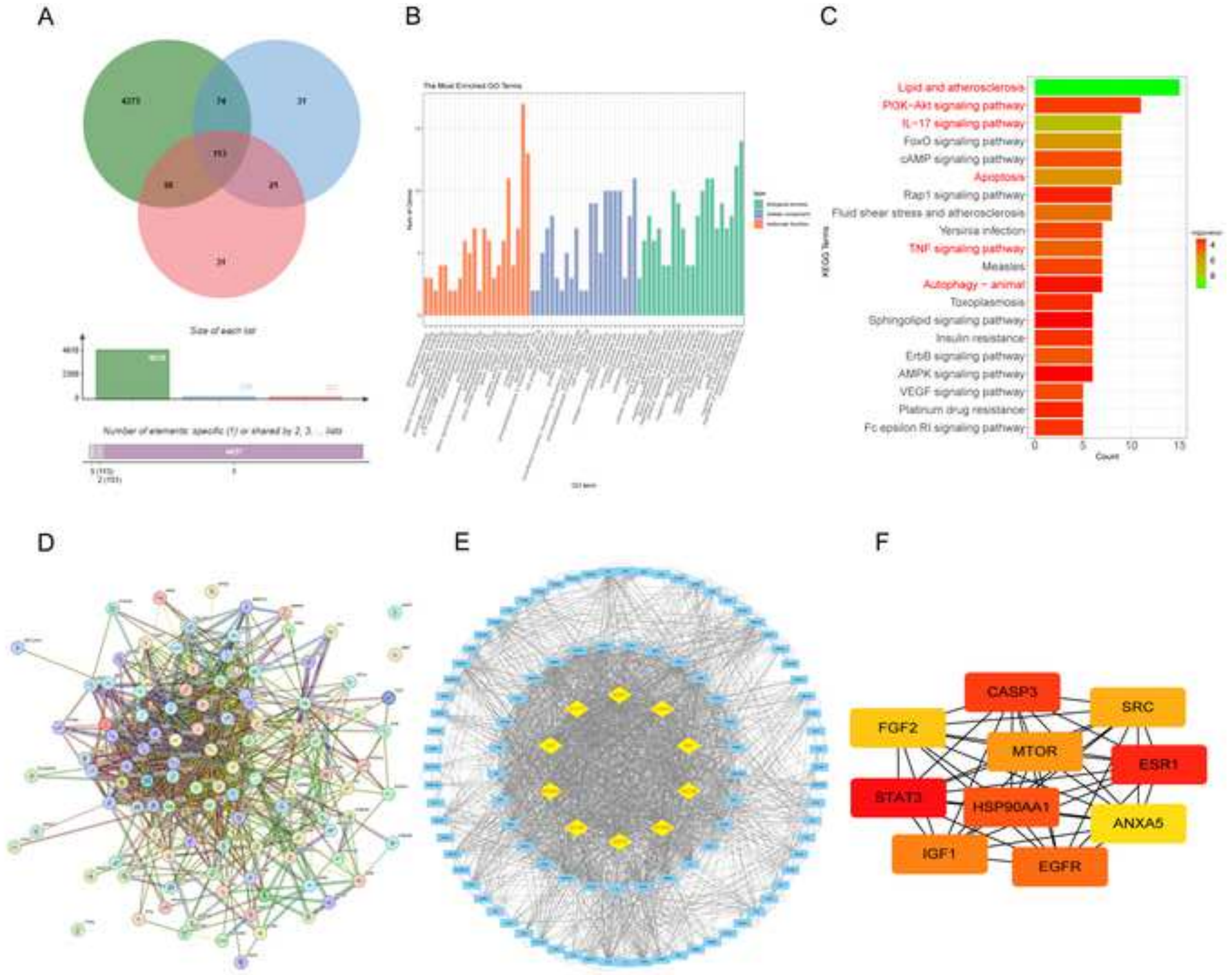

Figure 2

Click here to access/download;Figure;Figure 2.tif

Figures are blurry, should be sharp

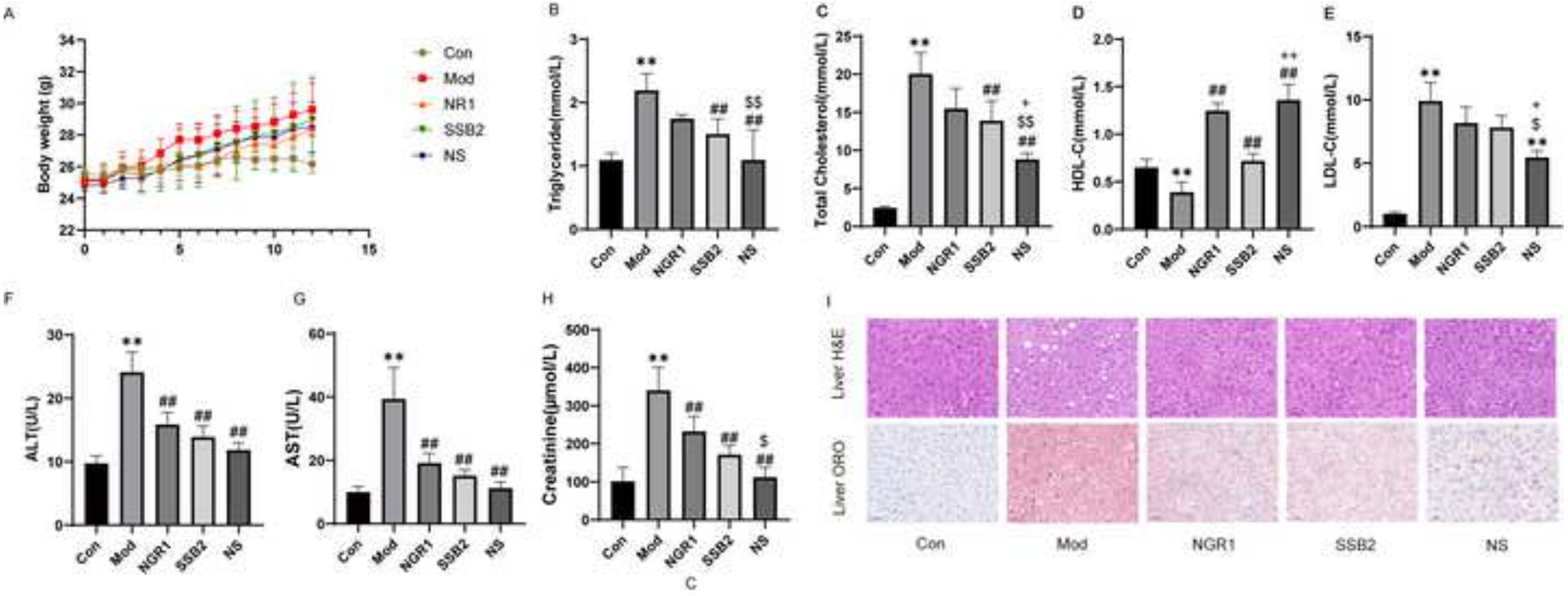

Figure 3

Click here to access/download;Figure;Figure 3.tif

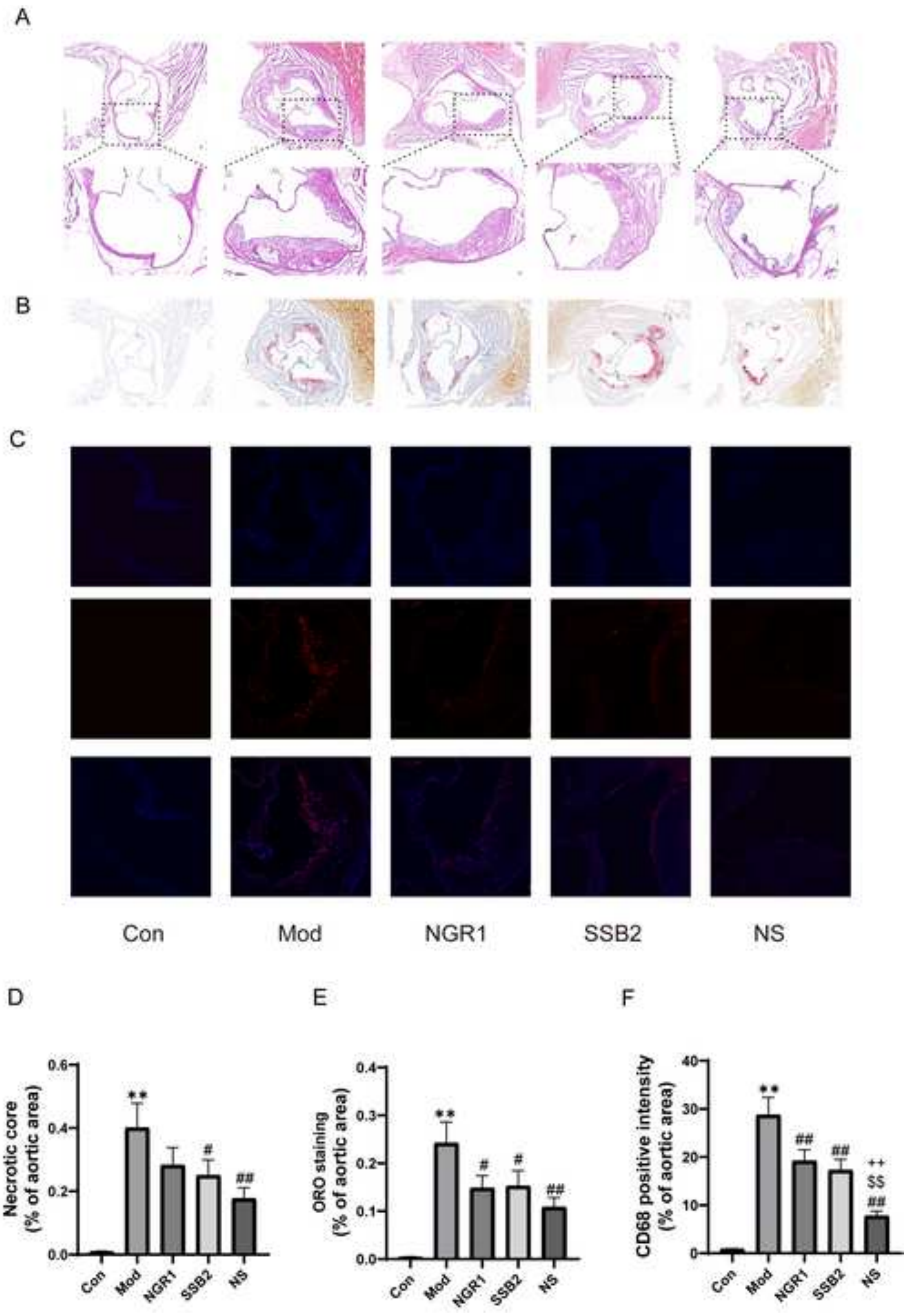

Click here to access/download;Figure;Figure 4.tif

Figures are blurry

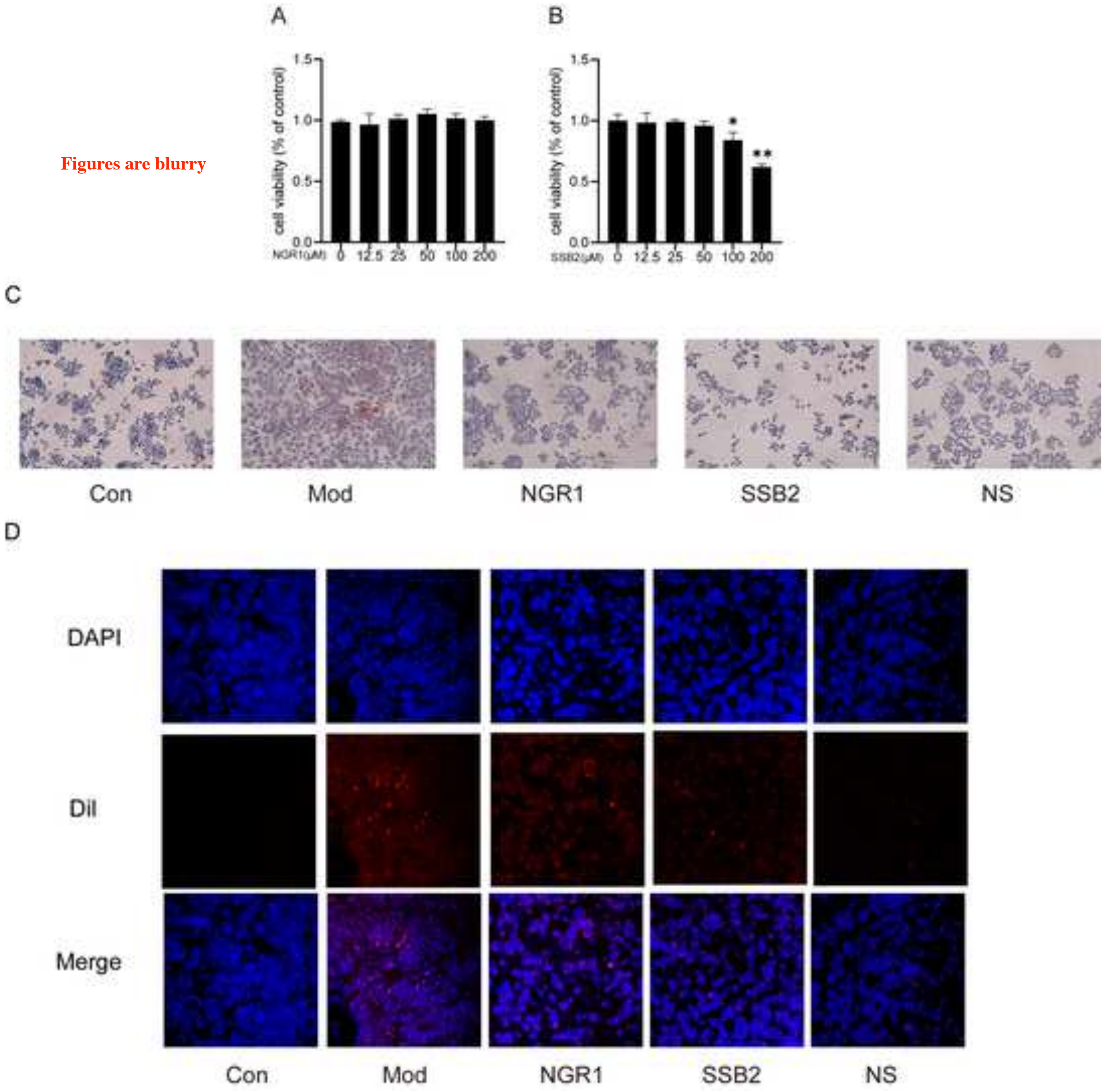

Figure 5

Click here to access/download;Figure;Figure 5.tif

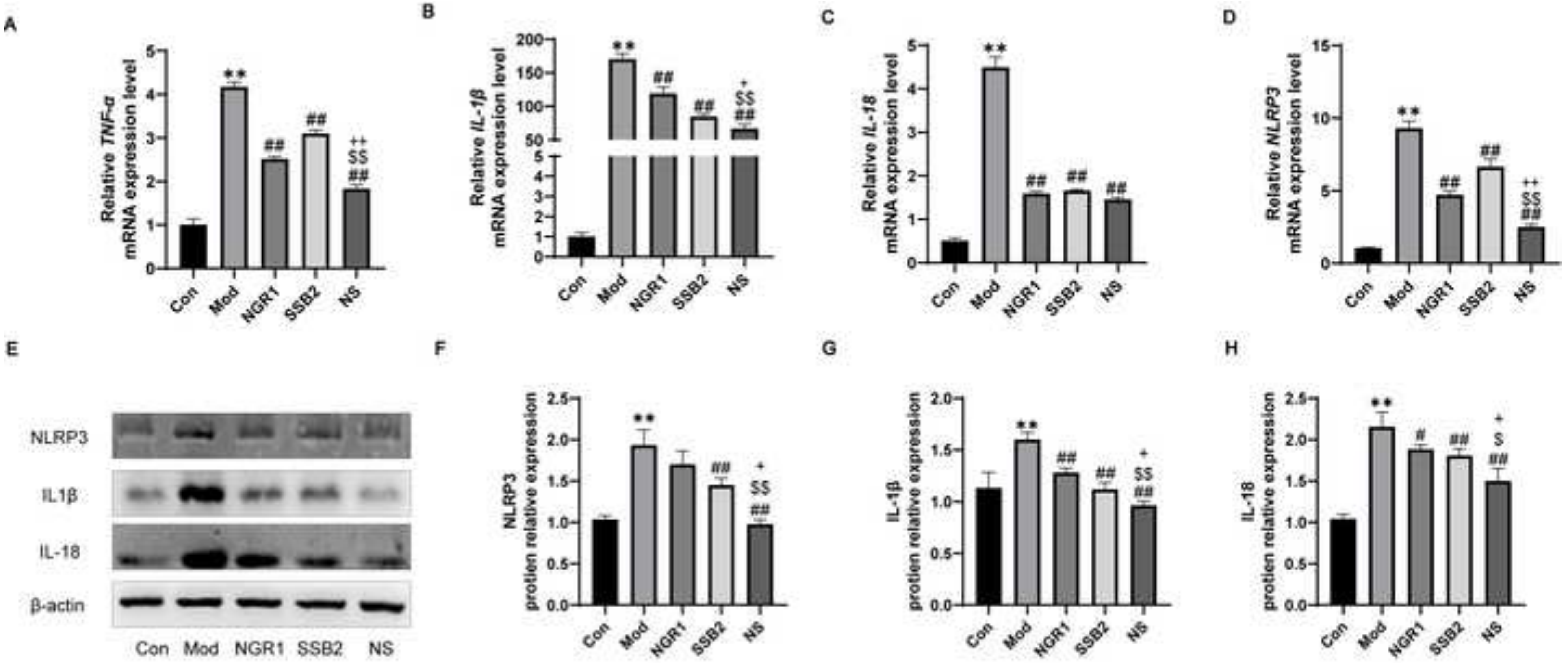

Figure 6 Click here to access/download;Figure;Figure 6.tif

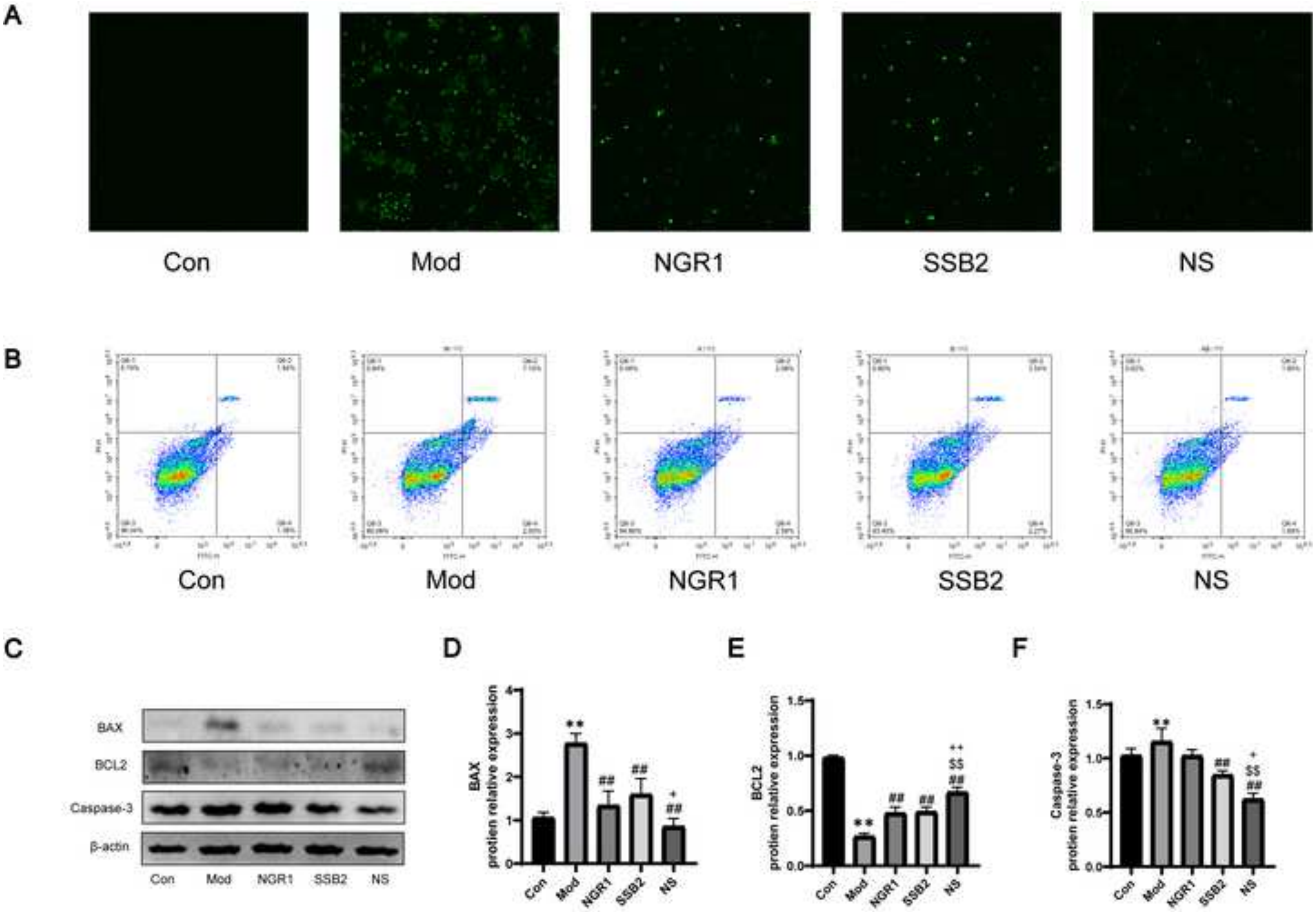

Figure 7

Click here to access/download;Figure;Figure 7.tif

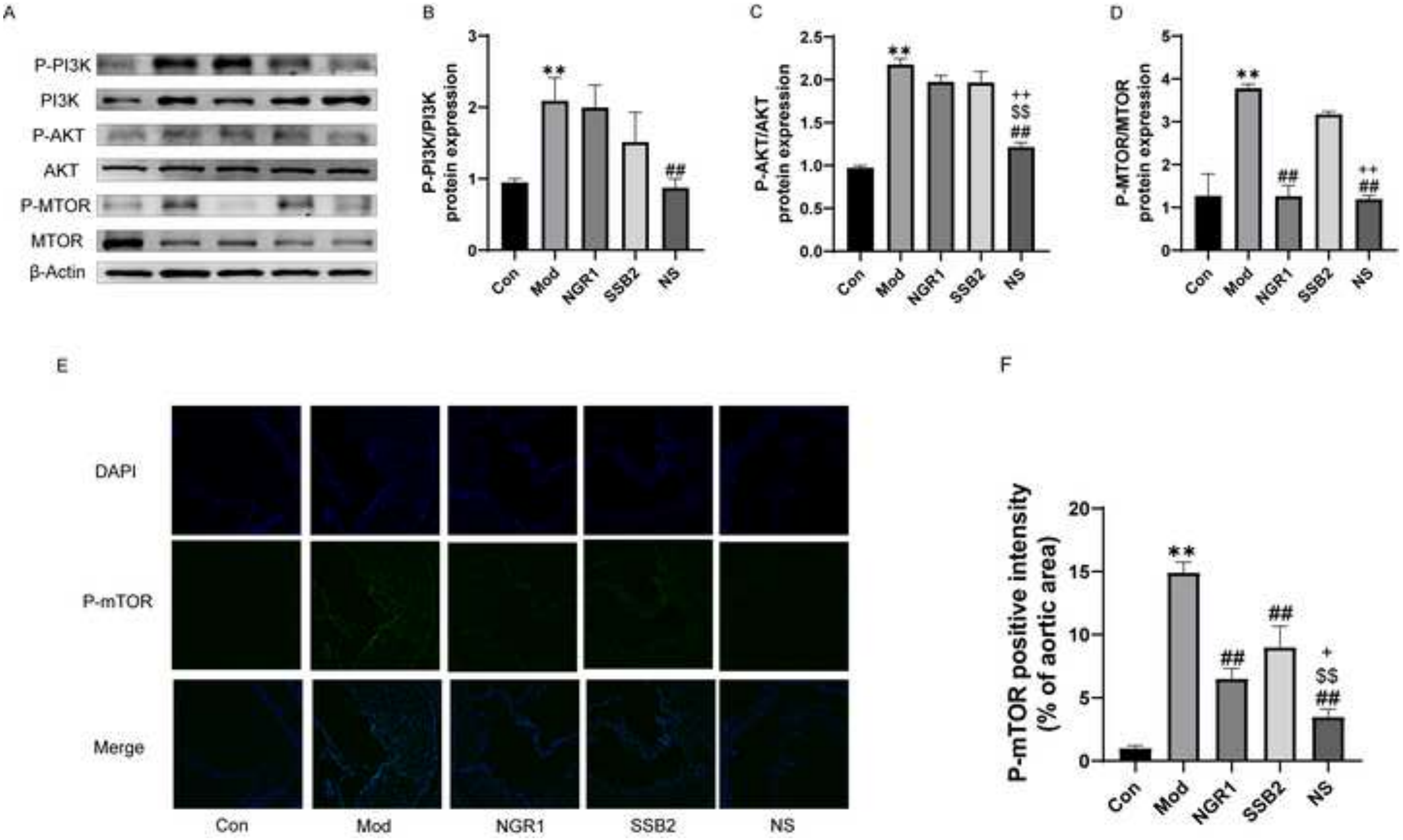

Figure 8

Click here to access/download;Figure;Figure 8.tif

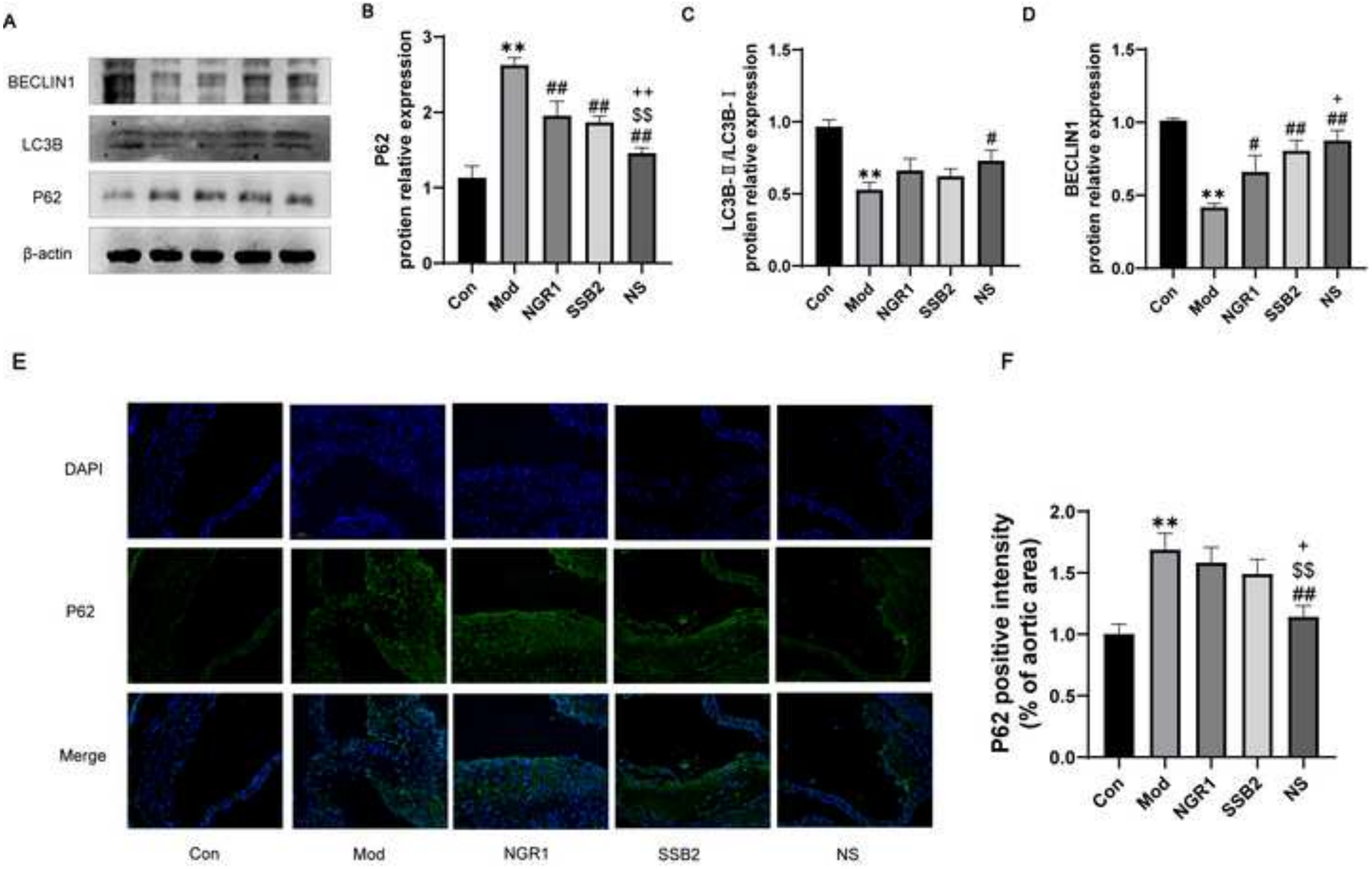

Figure 9

Click here to access/download;Figure;Figure 9.tif

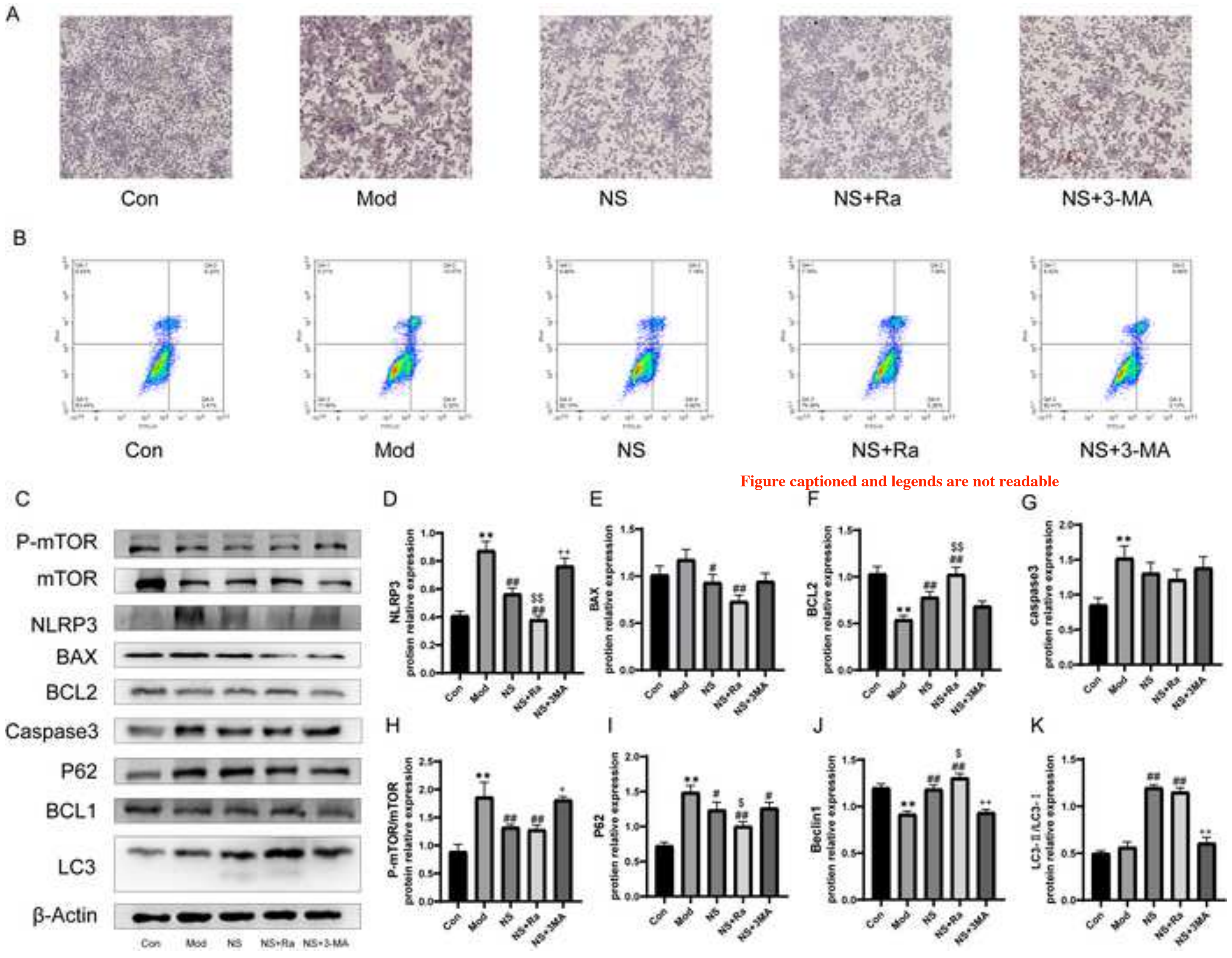

Figure 10

Click here to access/download;Figure;Figure 10.tif

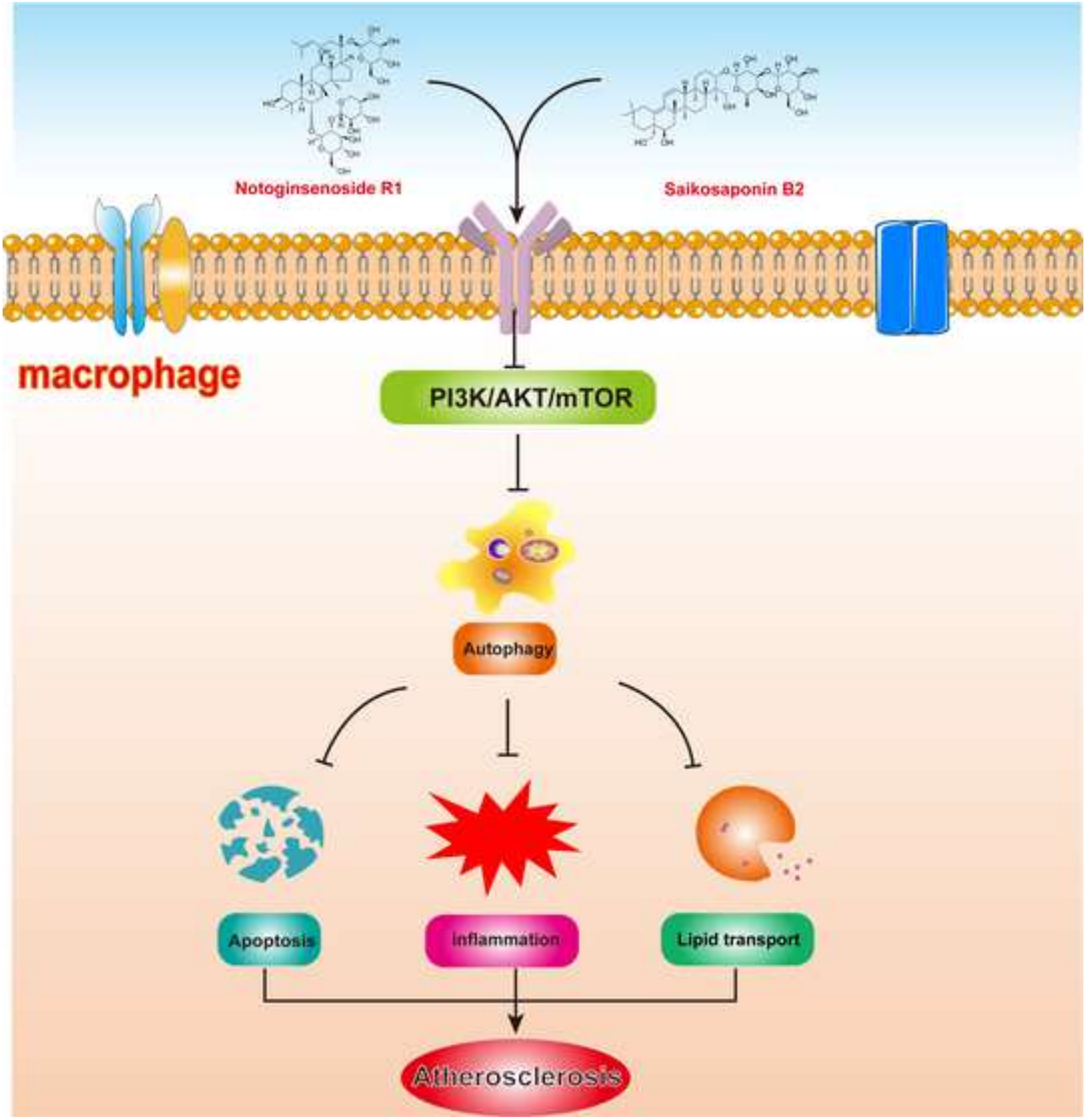

S1 Figure

Click here to access/download;Figure;S1 Figure.tif

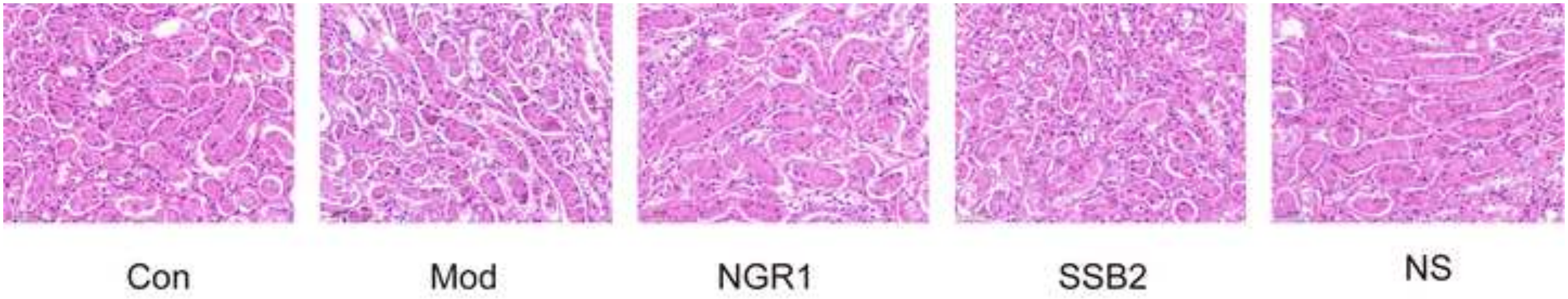

No legend, no caption, no title, no number?

Click here to access/download
Supporting Information
supplement materials.docx
