# Supplementary material for: Synergistic effects of notoginsenoside R1 and saikosaponin B2 in atherosclerosis: A novel approach targeting PI3K/AKT/mTOR pathway and macrophage autophagy
Source: PLoS One. 2025 Jun 27;20(6):e0326687. doi: 10.1371/journal.pone.0326687 (PMC12204583; doi:10.1371/journal.pone.0326687)
Supplement: Attachment — Submitted filename: Response letter.docx [file pone.0326687.s005.docx]

Dear Dr. Riyaz Ahmad and Reviewers,

Thank you for your constructive feedback and the opportunity to revise our manuscript titled "Synergistic Effects of Notoginsenoside R1 and Saikosaponin B2 in Atherosclerosis: A Novel Approach Targeting PI3K/AKT/mTOR Pathway and Macrophage Autophagy" (MS ID: PONE-D-24-45738). We have carefully addressed all comments from the reviewers and editors, and detailed responses are provided below. Revisions in the manuscript are highlighted in the "Revised Manuscript with Track Changes" file.

Journal Requirements

1. Style Requirements

The manuscript has been reformatted to comply with PLOS ONE’s style guidelines, including file naming conventions.

2. Code Sharing

No authorgenerated code was used in this study.

3. Animal Experiment Details

Methods of Sacrifice: Added to Section 2.2: "Mice were euthanized via cervical dislocation under deep anesthesia induced by intraperitoneal injection of sodium pentobarbital (50 mg/kg)."

Anesthesia/Analgesia: Added in Section 2.2: "For intraperitoneal injections, mice were briefly anesthetized with 2% isoflurane to minimize distress."

Efforts to Alleviate Suffering: Added in Section 2.2: "Mice were monitored daily for signs of pain or distress, and body weight was recorded weekly. No unexpected adverse events occurred."

4. Funding Statement

The Acknowledgments section has been revised to remove funding details. The updated Funding Statement in the online submission form reads:

"This work was supported by the Scientific Startup Foundation for Doctors of North Sichuan Medical College (CBY23QDA11 to XM) and the Key Research and Development Projects of the Sichuan Provincial Department of Science and Technology (2020YFS0528 to CZ). The funders had no role in study design, data collection, analysis, decision to publish, or manuscript preparation."

5. Original Blot/Gel Images

All uncropped, unadjusted Western blot images have been uploaded to the public repository Figshare (http://datadryad.org/share/4GvB7LlOnZ0wtL8Og2WIplaC9ZctRnAeJkkWt7K1FnI). These are cited in the figure legends.

Due to the high resolution and large file size of the figures, we were unable to upload them directly through the submission system. To ensure that the images are accessible to reviewers and readers, we have deposited all high-resolution figures in the [dryad/<https://datadryad.org/>] repository. The figures can be accessed via the following link: [http://datadryad.org/share/4GvB7LlOnZ0wtL8Og2WIplaC9ZctRnAeJkkWt7K1FnI].

Reviewer #1 Comments

1. Abstract Improvement

Abstract: Revised to emphasize novel findings: "This study demonstrates, for the first time, that the NS combination synergistically activates macrophage autophagy by suppressing the PI3K/AKT/mTOR pathway, thereby attenuating lipid accumulation, inflammation, and apoptosis in atherosclerotic models."

Atherosclerosis (AS) is a major global contributor to cardiovascular diseases and associated mortality. The traditional treatment primarily relies on statins; however, these drugs are often linked to side effects such as liver dysfunction and muscle impairment. Recent studies have highlighted the potential protective properties of saponin compounds derived from traditional herbal sources, such as notoginsenoside R1 (NGR1) and saikosaponin B2 (SSB2), in combating AS. However, the comprehensive effects of these compounds against atherosclerosis and their underlying mechanisms remain inadequately understood. Firstly, we employed network pharmacology analysis to identify 113 common targets, including mTOR and CASP3, for NGR1, SSB2, and atherosclerosis from databases such as TCMSP. We constructed a protein-protein interaction (PPI) network and performed GO and KEGG enrichment analyses, whcih revealed key signaling pathways involved in PI3K/Akt, inflammation, and autophagy. The atherosclerosis model was established using ApoE^-/-^ mice fed with a "Western diet," followed by treatment with NGR1, SSB2, or NS combination. Histological examinations, including hematoxylin-eosin (HE) staining, oil red O (ORO) staining, and CD68 immunofluorescence, were conducted to evaluate the pathological conditions of the aortic root, liver and kidneys in ApoE^-/-^ mice. Our findings demonstrate that the NS combination improves lipid levels, lipid transport, and unstable plaque formation in ApoE^-/-^ mice without adverse effects on liver or kidney function. Finally, oxidized low-density lipoprotein (ox-LDL) was used to culture RAW264.7 macrophages to establish an *in vitro* foam cell model. The effects of NS combination on lipid uptake, inflammatory response, apoptosis, the PI3K/Akt/mTOR signaling pathway, and autophagy were evaluated using methods such as CCK-8 assay, Oil Red O staining, immunofluorescence analysis, flow cytometry, RT-PCR, and Western blot analysis. The results indicated that NS combination promoted autophagy by inhibiting the PI3K/Akt/mTOR pathway. This significantly alleviated inflammation, reduced apoptosis, and decreasing lipid accumulation, thereby improving the pathological progression of atherosclerosis. Collectively, this study demonstrates, for the first time, that the NS combination synergistically activates macrophage autophagy by suppressing the PI3K/AKT/mTOR pathway, thereby attenuating lipid accumulation, inflammation, and apoptosis in atherosclerotic models.

2. Introduction Improvement

The revised paragraph is now more logical and engaging, providing a strong foundation for the study. Below is the revised paragraph for the reviewer’s reference:

“Despite some existing studies revealing the biological activity and potential therapeutic effects of NGR1 and SSB2 in atherosclerosis (AS), there is currently no definitive conclusion regarding their efficacy when applied individually. More importantly, whether NGR1 and SSB2 can synergistically enhance therapeutic effects remains underexplored. Therefore, the combined use of these two substances with unique pharmacological properties not only inherits traditional Chinese medicine compatibility theories but also represents an innovative exploration in modern medical translation. This study aims to delve into the potential of combining NGR1 and SSB2 for AS treatment based on contemporary understanding of the mechanisms underlying AS development. Specifically, through a comprehensive approach incorporating network pharmacology, in vitro cellular experiments, and in vivo animal studies, we will systematically evaluate their mechanisms of action in AS. The focus will be on how both agents may exert a synergistic effect by regulating the formation of macrophage-derived foam cells and influencing macrophage autophagy processes to improve therapeutic outcomes for AS.We anticipate that this combination therapy strategy will not only effectively alleviate the pathological progression of atherosclerosis but also reduce common side effects associated with existing treatments, ultimately providing a safer and more effective new treatment option for clinical practice. Consequently, this research not only theoretically expands new avenues for AS treatment but is also expected to offer more scientific evidence and practical guidance for the application of traditional Chinese medicine formulations.”

3. Materials and Methods Improvement

Regarding your request for references related to the protocols, we have revised the manuscript to include appropriate citations and references pertinent to the protocols utilized in our study. The specific protocol followed in this research has been meticulously outlined and relevant sources have been duly cited.

4. Discussion Improvement

The first paragraph now restates objectives and highlights key discoveries: "Our study aimed to elucidate the synergistic mechanisms of NGR1 and SSB2 in atherosclerosis. We identified that NS combination therapy uniquely restores autophagic flux in macrophages, a finding not previously reported for either compound alone."

In each paragraph of the Discussion, we now begin by presenting our key results (e.g., “As shown in Figure X” or “As indicated in Table Y”) and then discuss their implications in the context of existing literature.

We have revised the Discussion to consistently reference our data using phrases such as “As shown in Figure X” or “As indicated in Table Y”. This ensures that readers can easily locate the corresponding data in the manuscript.

5. Figure Quality

All figures have been reexported in high resolution (600 dpi). Scale bars and labels are now consistent across panels.

Reviewer #2 Comments

1. PPARγ, LXRα, and ABC Transporters

We sincerely thank the reviewer for their insightful comment regarding the importance of PPAR-γ, LXR-α, and ABC transporters in reverse cholesterol transport (RCT) in macrophages. We agree that these markers play a crucial role in lipid metabolism and atherosclerosis progression, and their investigation is essential to fully understand the mechanisms of our study.

In response to the reviewer’s suggestion, we are pleased to inform you that we have already performed gene expression studies (qPCR) to evaluate the effects of NS on PPAR-γ, LXR-α, and ABC transporters (including ABCA1 and ABCG1) in RAW264.7 macrophages. Our results demonstrate that NS treatment significantly upregulates the expression of these key markers, further supporting its role in promoting reverse cholesterol transport and attenuating lipid accumulation in macrophages.We have now included these findings in the revised manuscript (see Figure 4 and Figure 9)

2. mTOR and Lysosomal Markers (LAMP1/RAB7)

In response to the reviewer’s comment, we are pleased to inform you that we have already performed immunofluorescence experiments to evaluate the expression and localization of p-mTOR and LXR-α in RAW264.7 macrophages. These results have been included in the revised manuscript (see Figures 8-9). Our data demonstrate that NS treatment significantly modulates the expression and distribution of these markers, further supporting its role in regulating autophagy.

3. Catalog Numbers

All reagents now include catalog numbers (e.g., "AntiLC3B/MAP1LC3B Antibody (BM4827, Boster, China)").

4. Proofreading

The manuscript has undergone professional English editing. Typos (e.g., "Arteriosclerosis" → "atherosclerosis ") are corrected.

We appreciate the reviewers’ insights, which have strengthened the manuscript. Please contact us for further clarifications.

Sincerely,

Chunyang Zhou, PhD

Corresponding Author

Institute of Materia Medica, North Sichuan Medical College

Email: zhouchunyang@nsmc.edu.cn

Attachments:

1. Revised Manuscript with Track Changes

2. Manuscript

3. Response to Reviewers

4. Data Review URL
