# Supplementary material for: Synergistic effects of notoginsenoside R1 and saikosaponin B2 in atherosclerosis: A novel approach targeting PI3K/AKT/mTOR pathway and macrophage autophagy
Source: PLoS One. 2025 Jun 27;20(6):e0326687. doi: 10.1371/journal.pone.0326687 (PMC12204583; doi:10.1371/journal.pone.0326687)
Supplement: Attachment — Submitted filename: Response_letter_auresp_2.docx [file pone.0326687.s006.docx]

Dear Dr. Riyaz Ahmad and Reviewers,

Thank you for your constructive feedback and the opportunity to revise our manuscript titled "Synergistic Effects of Notoginsenoside R1 and Saikosaponin B2 in Atherosclerosis: A Novel Approach Targeting PI3K/AKT/mTOR Pathway and Macrophage Autophagy" (MS ID: PONE-D-24-45738R1). We have carefully addressed all comments from the reviewers and editors, and detailed responses are provided below. Revisions in the manuscript are highlighted in the "Revised Manuscript with Track Changes" file.

**Journal Requirement：**

1. Reference List Review

We have carefully reviewed the reference list. None of the cited articles have been retracted. All references have been checked for completeness, and any missing DOIs (References: 15, 27, 63, 65, 66, 70, 74) have been updated in accordance with the journal’s formatting guidelines.

**Additional Editor Comments:**

1. Concern regarding lack of colocalization analysis between mTOR and LAMP-1 in Figures 8 and 9.

We sincerely appreciate your insightful comment regarding the mTOR-LAMP-1 colocalization analysis. Accordingly, we have now performed dual-color confocal colocalization experiments as suggested, with the results incorporated in the revised Figure 8E and Figure 9P.

The new data are demonstrated in section 3.8 and 3.9 as follows:

“*Compared with the control group, which displayfewer yellow puncta,ox-LDL-induced RAW264.7 cells exhibited significantly enhanced co-localization of p-mTOR (green) with LAMP-1 (red), with a marked increase in the area of yellow merged puncta in confocal images. Notably, NS treatment significantly suppressed the ox-LDL-induced lysosomal localization of p-mTOR, as evidenced by a substantial reduction in co-localized yellow puncta”* (Fig.8E)

*“In comparison to the NS group, rapamycin-treated RAW264.7 cells exhibited further reduced co-localization of p-mTOR with LAMP-1. Conversely, 3-MA-treated RAW264.7 cells demonstrated significantly enhanced p-mTOR/LAMP-1 co-localization, with a marked increase in the area of yellow merged puncta observed in the confocal microscopy images (Fig. 9P)*.”

The revised Discussion (section 4, page 23 and 24) is outlined as follows:“*Our study revealed a significant increase in colocalization between p-mTOR and LAMP-1 in ox-LDL-stimulated RAW264.7 macrophages (Figure 8E). This enhanced colocalization suggests that lipid overload leads to hyperactivation of the PI3K/AKT/mTOR signaling pathway, promoting mTORC1 recruitment and anchoring to the lysosomal membrane. Previous studies have demonstrated that lysosomal localization of mTORC1 is essential for its biological functions in promoting cell growth and inhibiting autophagy(63). As a highly glycosylated lysosomal membrane marker, LAMP-1 not only serves as a key indicator of lysosomal biogenesis(64), but also plays a crucial role as a mediator in autophagosome-lysosome fusion, which is critical for completing autophagic flux (65). However, when mTOR becomes overactivated and accumulates on LAMP-1-positive lysosomal membranes, it phosphorylates and inhibits ULK1 (a key kinase involved in autophagy initiation), thereby blocking autophagic progression(66). This inhibitory effect may impair the clearance of damaged lipids and protein aggregates, exacerbating intracellular lipid accumulation and ultimately promoting macrophage transformation into foam cells. This discovery reveals the significant role of NS combination therapy in regulating autophagy to prevent atherosclerosis.”“As shown in Figure 9P, our immunofluorescence co-localization analysis revealed that rapamycin-treated RAW264.7 cells exhibited significantly reduced co-localization of p-mTOR with LAMP-1 compared to the normal control group. This reduction islikely attributable to rapamycin-mediated inhibition of mTOR activity which consequently impaired autophagosome-lysosome fusion. Conversely, 3-MA treatment markedly enhanced the p-mTOR/LAMP-1 co-localization, potentially through its inhibitory effect on the initial stages of autophagy, which secondarily modulates mTOR activity, thereby promoting the association between p-mTOR and LAMP-1.”*

2. Use of “recent studies” with only a single reference

Thank you for pointing out this inconsistency. We have thoroughly reviewed the manuscript and:

1) Added additional relevant citations where appropriate to justify the plural usage ( references 7, 8, and 9 on page 3).

2) Replaced plural expressions like “recent studies” with “a recent study” where only one reference is cited (reference 13 on page 4).

3. Use of abbreviation “AS” for atherosclerosis

We have revised the manuscript to consistently use the full term "atherosclerosis" throughout the text, all instances of "AS" have been replaced accordingly.

Due to the high resolution and large file size of the figures, we were unable to upload them directly through the submission system. To ensure that the images are accessible to reviewers and readers, we have deposited all high-resolution figures in the [dryad/<https://datadryad.org/>] repository. The figures can be accessed via the following link: [http://datadryad.org/share/4GvB7LlOnZ0wtL8Og2WIplaC9ZctRnAeJkkWt7K1FnI].

**Reviewer #2 Comments**

1. mTOR-LAMP-1 colocalization analysis

We sincerely appreciate your insightful comment regarding the mTOR-LAMP-1 colocalization analysis. As mentioned earlier, we have now performed dual-color confocal colocalization experiments, and the results have been incorporated in the revised Figure 8E and Figure 9P.

The new data demonstrate:

Compared with the control group, which displayfewer yellow puncta,ox-LDL-induced RAW264.7 cells exhibited significantly enhanced co-localization of p-mTOR (green) with LAMP-1 (red), with a marked increase in the area of yellow merged puncta in confocal images. Notably, NS treatment significantly suppressed the ox-LDL-induced lysosomal localization of p-mTOR, as evidenced by a substantial reduction in co-localized yellow puncta (Fig.8E).

In comparison to the NS group, rapamycin-treated RAW264.7 cells exhibited further reduced co-localization of p-mTOR with LAMP-1. Conversely, 3-MA-treated RAW264.7 cells demonstrated significantly enhanced p-mTOR/LAMP-1 co-localization, with a marked increase in the area of yellow merged puncta observed in the confocal microscopy images (Fig. 9P)

We believe this comprehensive approach strengthens the study's mechanistic insights. We hope that the revised manuscript and the new data fully address the concerns raised. Thank you for prompting this important improvement. We look forward to your favorable consideration.

Sincerely,

Chunyang Zhou, PhD

Corresponding Author

Institute of Materia Medica, North Sichuan Medical College

Email: zhouchunyang@nsmc.edu.cn
